# Two Distinct Diazo–Diazo Cross-Coupling Reactions between α-Aryldiazo Ketones and Vinyldiazo Esters Using Gold Catalyst and Tetrabutylammonium Fluoride

**DOI:** 10.1021/acs.orglett.5c00068

**Published:** 2025-02-19

**Authors:** Debashis Barik, Rai-Shung Liu

**Affiliations:** † Department of Chemistry, National Tsing Hua University, Hsinchu 30013, Taiwan; ‡ Department of Chemistry and College of Semiconductor Research Institute, National Tsing-Hua University, Hsinchu 30013, Taiwan

## Abstract

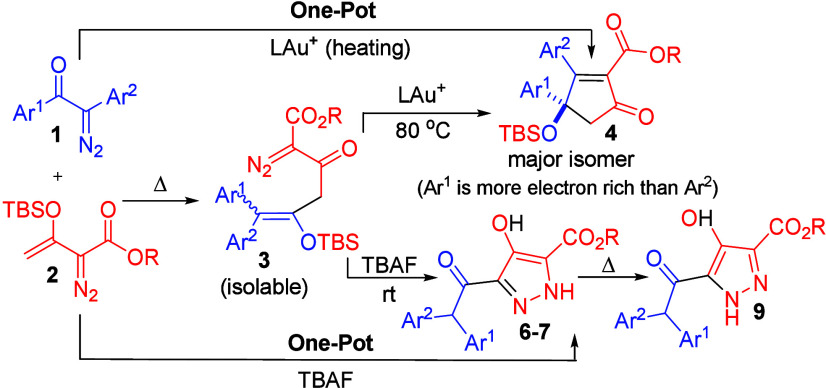

Two distinct coupling reactions of α-aryldiazo ketones with vinyldiazo esters are described using gold catalyst (5 mol %) and Bu_4_NF promoter (1.5 equiv), respectively. This new synthetic scheme involves a prior rearrangement of α-aryldiazo ketones to generate diarylketenes under thermal conditions whereas vinyldiazo esters serve as nucleophiles to afford isolable 4-siloxy-1-diazo-pent-4-en-2-ones. This diazo–diazo cross-coupling reaction yields highly substituted cyclopentenones using gold catalyst and substituted pyrazole derivatives with Bu_4_NF promoter.

Catalytic diazo–diazo cross-couplings are a topic of growing interest.^1−3^ The initial target of such reactions focuses on olefin synthesis^2^ with a release of N_2_ as the only byproduct. This olefin synthesis meets atom economy compared to the well-known Wittig reactions and olefin metathesis, which produced Ph_3_PO or ethylene gas instead. In a general protocol, the donor–acceptor diazo species are instantly transformed into Rh-, Ru-, and Cu-carbenes that are subsequently trapped with highly nucleophilic α-diazo esters to yield trisubstituted olefins (eq 1, Scheme 1). This diazo–diazo cross-coupling is further applicable to the synthesis of acyclic dienes using donor–acceptor diazo species, vinyldiazo esters, and gold catalyst as shown in eq 2, Scheme 1; again, the former generates gold carbenes, whereas the latter serves as nucleophiles. α-Aryldiazo ketones are also prone to the Wolff reactions to form diarylketenes in a hot solution (60–80 °C).^3c,4^ Hence, we plan to explore a new diazo–diazo cross-coupling involving α-vinyldiazo esters as nucleophiles and α-aryldiazo arylketones as diarylketene precursors to seek new chemoselectivity beyond olefin synthesis (eq 4, Scheme 1). This work reports one-pot synthesis of cyclopentenone scaffolds from α-vinyldiazo esters and α-aryldiazo ketones in the presence of a gold catalyst (eq 3, Scheme 1). Notably, the chemoselectivity is switched to the regio-controlled synthesis of pyrazole derivatives (**6**–**7**) when Bu_4_NF promoter was employed (eq 3, Scheme 1). Interestingly, this pyrazole regioisomer **6** or **7** is convertible to the other regioisomer **9** under suitable conditions. With our efforts, initial thermal reactions between α-aryldiazo ketones (**1**) and α-vinyldiazo esters (**2**) efficiently yielded 4-siloxy-1-diazo-pent-4-en-2-ones (**3**), which turns out to be viable intermediates for the formation of highly substituted cyclopentenone derivatives **4**–**5** and pyrazole products **6**–**7**.

Table 1 shows the optimized yields for the diazo–diazo cross-coupling between 2-diazo-1,2-diphenylethan-1-one **1a** and ethyl 3-((tert-butyldimethylsilyl)oxy)-2-diazobut-3-enoate **2a** (**2a**/**1a** = 1.3) using gold catalysts. Initially, the reaction mixture was placed in hot DCE to ensure a complete Wolff reaction of species **1a**.^3^ Subsequently, JohnphosAuCl/AgNTf_2_ (5 mol %) was added to this solution at room temperature with stirring for 12 h. In this instance, we obtained only species **3a** in a 64% yield (entry 1). We thus operated the reaction at an elevated temperature (80 °C, 6 h), further yielding the desired product **4a** in 58% yield (entry 2). Accordingly, species **3a** was the precursor for our target **4a**. With this new condition, various gold catalysts LAuCl/AgNTf_2_ (L= PPh_3_, P(OPh)_3_ and IPr) were tested to give our target **4a** in 44–49% yields (entries 3–5), showing no improvement. We altered the silver salt as in JohnPhosAuCl/AgSbF_6_, increasing the yield of compound **4a** to 65% (entry 6). Silver-free catalysts such as JohnPhosAuCl/NaBArF (BArF = B[3,5-(CF_3_)_2_C_6_H_3_]_4_) gave our target **4a** in only 36% yield (entry 7). JohnPhosAuCl/AgSbF_6_, in different solvents, gave the following yields DCM (55%), THF (52%), and toluene (43%); DCE proves to be the most effective solvent (entries 8–10). AgSbF_6_ was catalytically inactive (entry 11).

**Scheme 1:**
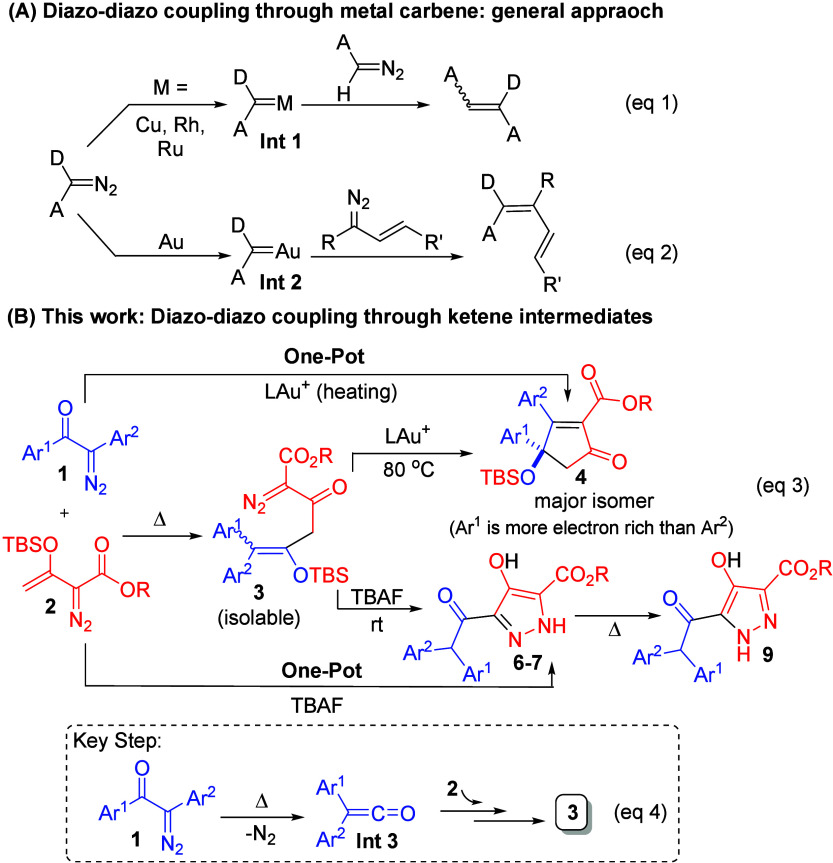
Scope for Diazo–Diazo Cross-Coupling Reactions

**Table 1:**
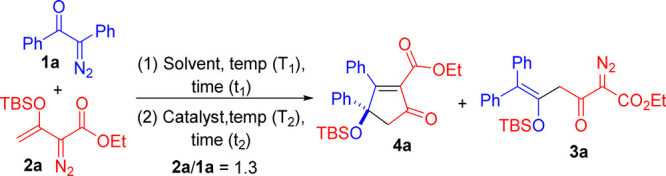
Catalyst Screening and Optimization of One-Pot Reaction Conditions^a^

entries	catalyst (5 mol %)	solvent	temp (°C) (*T*_1_/*T*_2_)	time (h) (*t*_1_/*t*_2_)	**4a** (yield %)^b^
1^c^	LAuCl/AgNTf_2_	DCE	80/rt	1.5/12	0
2	LAuCl/AgNTf_2_	DCE	80/80	1.5/6	58
3	PPh_3_AuCl/AgNTf_2_	DCE	80/80	1.5/6	49
4	P(OPh)_3_AuCl/AgNTf_2_	DCE	80/80	1.5/6	45
5	IPrAuCl/AgNTf_2_	DCE	80/80	1.5/6	44
**6**	**LAuCl/AgSbF**_**6**_	**DCE**	**80/80**	**1.5/4**	**65**
7	LAuCl/NaBArF	DCE	80/80	1.5/6	36
8	LAuCl/AgSbF_6_	DCM	50/50	2/8	55
9	LAuCl/AgSbF_6_	THF	65/65	2/7	52
10	LAuCl/AgSbF_6_	PhMe	110/110	2.5/6	43
11	AgSbF_6_	DCE	80/80	2/12	0

a Reaction condition: **1a** (0.4 mmol) and **2a** (0.52 mmol) in solvent (5 mL) under N_2_.

b The product yields were calculated after column chromatography on silica gel.

c L = P(*t*-Bu)2(o-biphenyl), and compound **3a** was isolated.

**Scheme 2:**
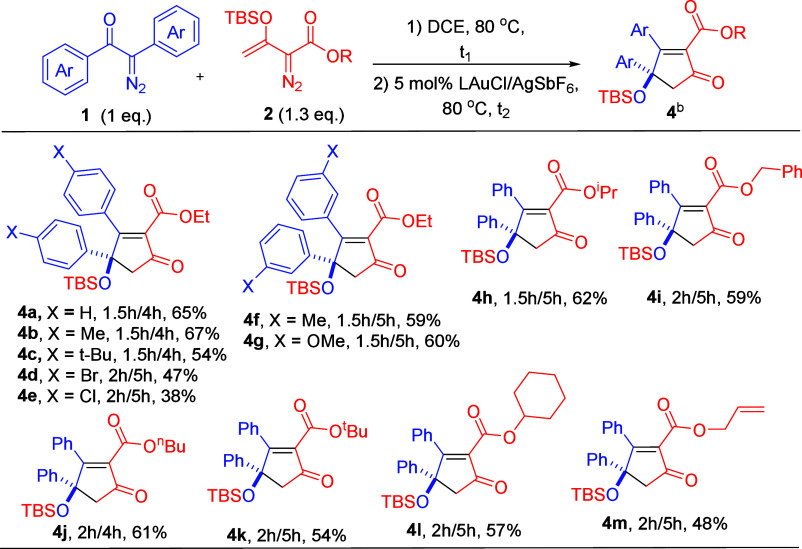
Scope for α-aryl Diazo Substrates Bearing the Same Aryl Groups and Vinyldiazo Esters Reaction condition: **1a** (0.4 mmol), **2a** (0.52 mmol), and 5 mol % LAuCl/AgSbF_6_ in DCE (5 mL) under N_2_ atmosphere. L = P(*t*-Bu)_2_(o-biphenyl). The product yields were calculated after column chromatography on silica gel.

**Scheme 3:**
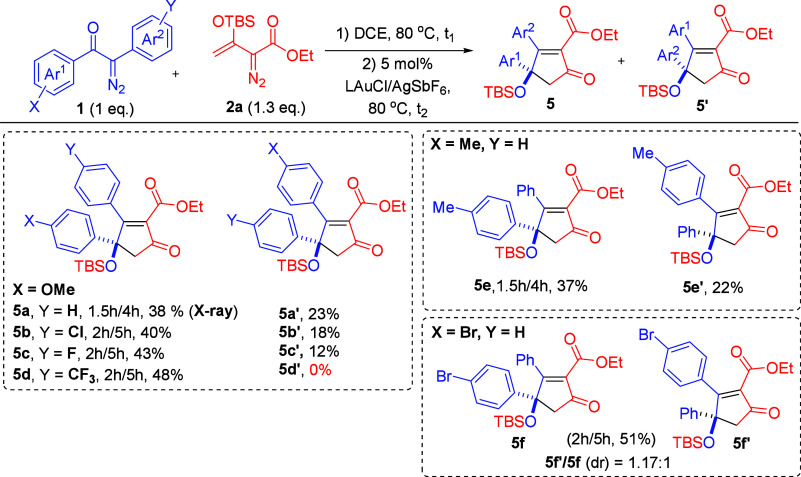
Scope for α-Aryldiazo Ketones Bearing Two Different Aryl Groups Reaction condition: **1a** (0.4 mmol), **2a** (0.52 mmol), and 5 mol % LAuCl/AgSbF_6_ in DCE (5 mL) under N_2_ atmosphere. The product yields were calculated after column chromatography on silica gel. L = P(*t*-Bu)_2_(o-biphenyl).

**Scheme 4:**
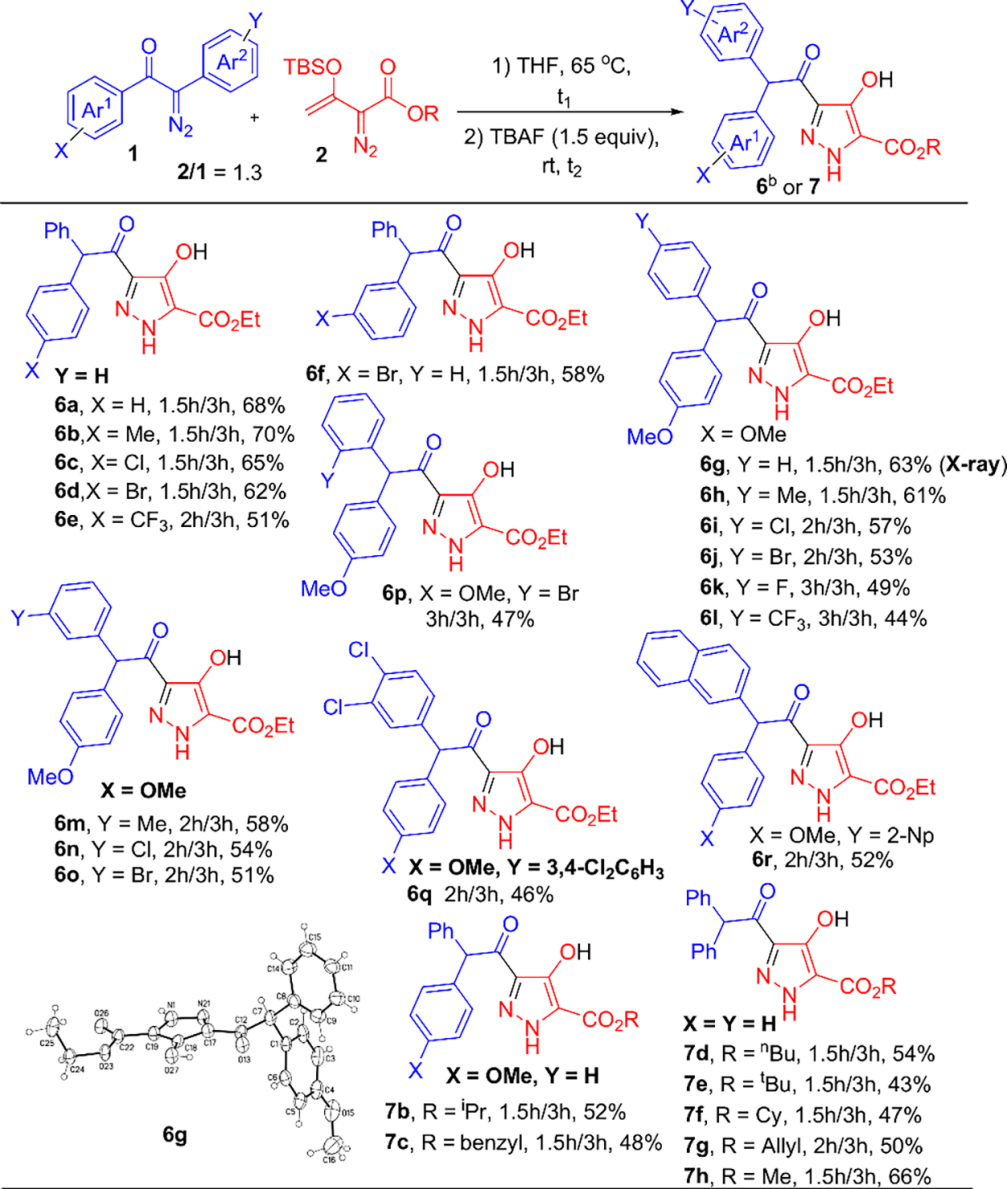
TBAF-Promoted Synthesis of Pyrazole Derivatives Reaction condition: **1a** (0.4 mmol), **2a** (0.52 mmol), and TBAF (1.5 equiv) in THF (5 mL) under N_2_ atmosphere. TBAF = Bu_4_NF. The product yields were calculated after column chromatography on silica gel.

**Scheme 5:**
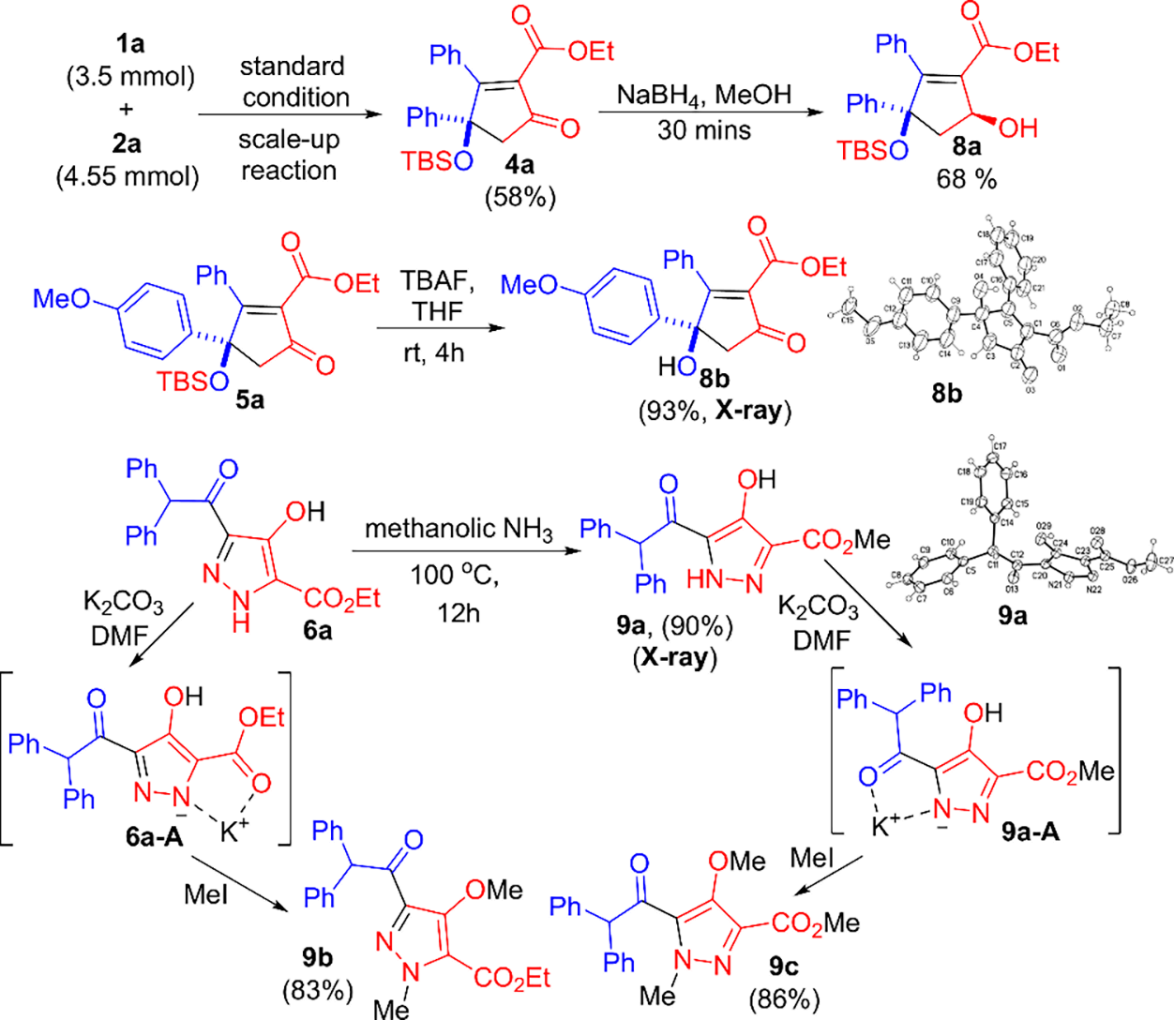
Scale-Up Reaction and Chemical Functionalizations

**Scheme 6:**
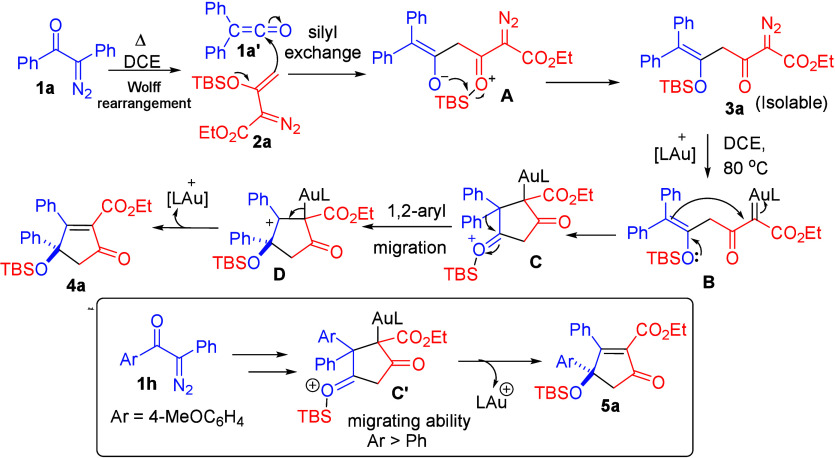
Plausible Mechanism for Gold-Catalyzed One-Pot Reaction

**Scheme 7:**
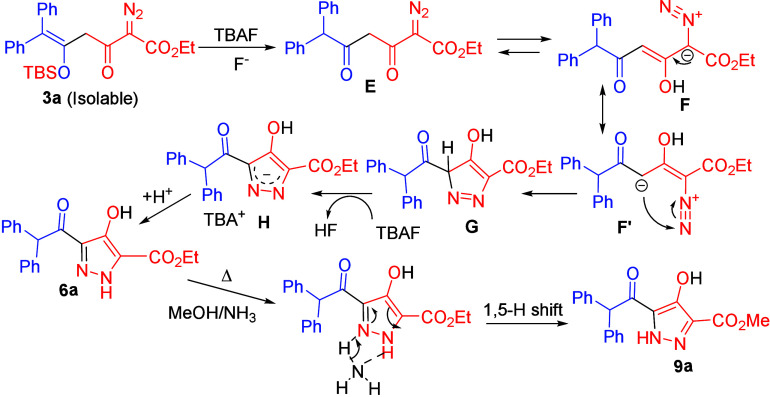
Plausible Mechanism for Selective Pyrazole Synthesis

We assessed the substrate scope of α-aryldiazo ketones **1** bearing two identical aryl groups; their Au-catalyzed couplings with various vinyldiazo esters **2** are summarized in Scheme 2. For α-diazo ketones, **1a**–**1e** bearing para-substituents (X = H, Me, ^t^Bu, Br, and Cl) at the aryl groups afforded the desired substituted cyclopentenones **4a**–**4e** in 38–67% yields. Herein, α-aryldiazo ketones with electron-rich aryl rings (X = H, Me, and ^*t*^Bu) are more efficient than their electron-withdrawing analogues (X = Br and Cl) because the former has better migration ability in the Wolff rearrangement. This cross-coupling reaction works well with their meta-substituted phenyl analogues **1f**–**1g** (X = Me and OMe), resulting in the expected products **4f**–**4g** in 59% and 60% yields, respectively. Several α-vinyldiazo species **2b**–**2g**, bearing different ester groups including *i*-propyl, benzyl, *n*-butyl, *t*-butyl, cyclohexyl, and allyl, could deliver the corresponding products **4h**–**4m** in 48–62% yields.

Scheme 3 shows the substrate scope of α-aryldiazo ketones (**1**) bearing two different aryl groups; the other partner is species **2a**. There are two possible products **5** and **5′** due to their competing 1,2-aryl migration rates in a key intermediate. The two products were separable using an HPLC instrument equipped with a silica column (Cosmosil 5SL-II, 15 μm particle size). For α-aryldiazo ketones **1h**–**1j** bearing Ar^1^ = 4-MeOC_6_H_4_ and Ar^2^ = 4-YC_6_H_4_ (Y = H, Cl and F), the major isomers **5a**–**5c** were delivered in 38–43% yields together with minor isomers **5a′**–**5c′** in 12–23% yields. Notably, a strong electron-withdrawing *para*-substituent **1k** (X = CF_3_) yielded only one product **5d** in 48% yield. We next tested the reaction on *para*-methylphenyl ketone **1l** (X = Me), giving the major isomer **5e** and its minor isomer **5e′** in 37% and 22% yields, respectively, whereas the *para*-bromophenyl ketone **1m** produces the desired product **5f**/**5f′** in a 51% overall yield with a 1.17:1 ratio. The molecular structure was elucidated by X-ray diffraction of major isomer **5a**(5) and also its derivative **8b**(5) which was obtained from a desilylation reaction (vide infra), whereas the structure of the minor isomer was confirmed by ^1^H, ^13^C, and ^1^H NOE spectra. We also tested this gold catalysis on α-methyldiazo phenylketone (**1″**), which failed to give the desired product (Scheme S1, see SI). This daizo species **1″** is not an applicable substrate for the next Bu_4_NF-promoted pyrazoles.

We performed several experiments to ascertain the intermediacy of species (**3**). Treatment of α-aryldiazo substrates (**1a**) with vinyldiazo ester (**2a**) under thermal conditions (80 °C, 1 h, eq 5) yielded single product **3a** in good yield. In contrast, the α-aryldiazo substrate (**1h**) produced two separable regioisomers, **3n** and **3n′**, with respective yields of 48% and 29%, and the E/Z selectivity was affected by the aryl group size, where the more bulky aryl (Ar) is preferable in the trans position to the siloxy group. The structures of compounds **3n** and **3n′** were confirmed using ^1^H NMR, 2D NMR (HMBC, HSQC), and IR spectroscopy (see Supporting Information). The two isomers **3n** and **3n′** were separately treated with JohnPhosAuCl/AgSbF_6_ (5 mol %) and Bu_4_NF (1.5 equiv), respectively. Despite their isomeric structures, the two species **3n** and **3n′** yielded **5a** and **5a′** nearly in the same amount with respective 50–53% and 30–31% yields (eqs 6–7), whereas the treatment of Bu_4_NF (1.5 equiv) with either isomer **3n** or isomer **3n′** delivered pyrazole product (**6g**) exclusively in 90–92% yields (eqs 6–7). The molecular structure of **6g** was inferred from X-ray diffraction study;^5^ its ORTEP image is shown in Scheme 4.
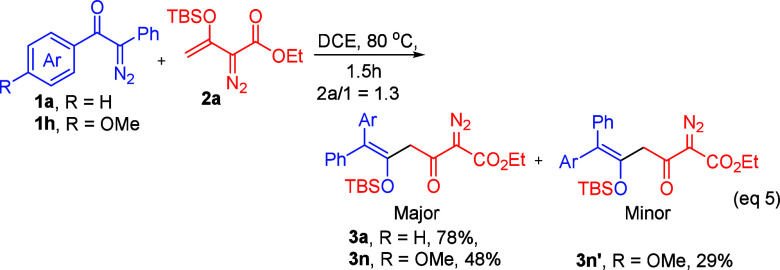

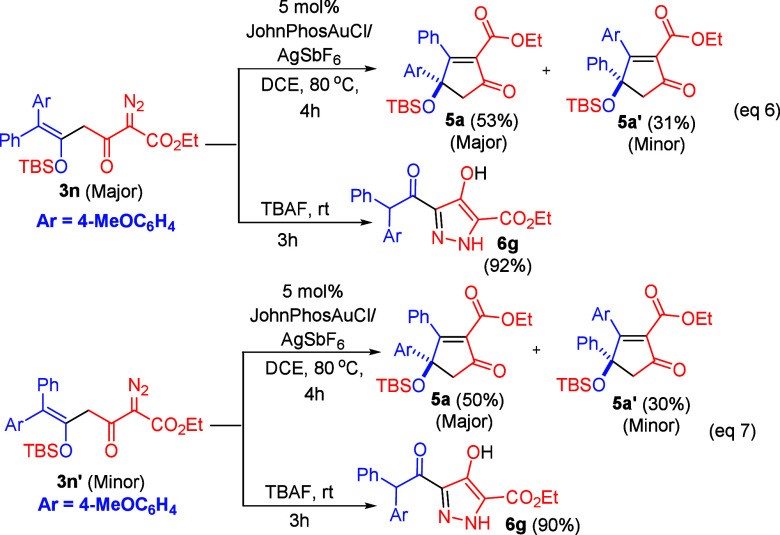


We turned our attention to the synthesis of substituted pyrazoles such as **6** and **7** as Bu_4_NF (1.5 equiv) altered the chemoselectivity; the significance of this synthesis is the excellent selectivity toward one specific pyrazole product with its NH group neighboring the CO_2_R group. We did not isolate the other pyrazole isomer with NH adjacent to the ketone group. To optimize the condition (Table S1, see SI), we examined the reaction efficiency in different solvents, with THF being the best solvent.

Scheme 4 summarizes the results for the diazo–diazo cross-coupling reactions using TBAF as the promoter. The initial two reactants were heated in THF (65 °C, 1.5–3 h) before treatment with TBAF (1.5 equiv). For both *para*-substituents (X = H, Me, Cl, Br, and CF_3_) and *meta*-substituents (X = Br) on the aryl ketones (Ar^1^), their annulations with standard vinyldiazo ester **2a** delivered the products **6a**–**6f** in 51–70% yields. Similarly, we altered the *para*-substituents (Y = H, Me, Cl, Br, F, and CF_3_), *meta*-substituents (Y= Me, Cl, and Br), and *ortho*-substituents (Y= Br) of the α-aryldiazo moieties (Ar^2^ = YC_6_H_4_); the expected products **6g**–**6p** were obtained in 44–63% yields. We prepared 3,4-dichlorophenyl and 2-naphthyl substituents on the phenyldiazo ketones (Ar^2^ = YAr), affording the desired products **6q** and **6r** in 46% and 52% yields, respectively. For vinyldiazo esters **2** bearing various ester groups (R= ^*i*^Pr, benzyl, ^*n*^Bu, ^*t*^Bu, Cy, allyl, and Me), this TBAF-promoted reaction afforded the expected pyrazole derivatives **7b**–**7h** in 43–66% yields.

Chemical functionalizations of the two reactions were demonstrated by the gram-scale reactions shown in Scheme 5. Treatment of **1a** (0.78 g, 3.5 mmol) with **2a** (1.3 g, 4.55 mmol) under standard conditions yielded **4a** (o.89 g) in 58% yield. Subsequently, selective reduction of **4a** using NaBH_4_ afforded **8a** with a 68% yield, and its stereochemistry was confirmed by ^1^H NOE spectroscopy. Compound **5a** was treated with TBAF, resulting in a desilylation to yield **8b** in 93% yield; its molecular structure was confirmed by X-ray diffraction analysis as well. Another representative **6a** was heated in a methanolic NH_3_ solution in an attempt to produce an amide but led to a different isomer **9a** with 90% yield; its structure was also confirmed by X-ray analysis.^5^ Accordingly, initial isomer **6a** was kinetically favorable whereas its regioisomer **9a** was thermodynamically favorable. The availability of two distinct pyrazole isomers **6a** and **9a** enables two respective methylations using MeI to give two distinct alkylation products **9b** and **9c**. This outcome indicates that their resulting amide anions from the K_2_CO_3_ treatment are different from each other instead of being equivalent. In other words, the pyrazole ring of species **6a** and **9a** will not generate a delocalized anion; instead, chelating potassium salts **6a-A** and **9a-A** are viable intermediates to react with MeI with two distinct regioselectivities.

In Scheme 6, we propose a mechanism in which α-phenyldiazo ketone **1a** first undergoes a thermal Wolff rearrangement to generate ketene intermediate **1a′** that was trapped by ethyl 3-siloxy-2-diazobut-3-enoate **2a** to form the zwitter ionic intermediate **A**. This type of zwitter ionic intermediate was previously reported by the Raynolds’ group,^6^ resulting from a reaction between ketene and silyl enol ether. A silyl exchange within intermediate **A** affords isolable intermediate **3a**. Notably, the gold catalyst enables the diazo decomposition of compound **3a** to generate a gold carbene intermediate **B**, which subsequently undergoes an intramolecular cyclization through an enol/carbene reaction, leading to cyclic oxonium intermediate **C**. Cationic intermediate **C** facilitates 1,2-aryl migration, ultimately leading to the formation of observed product **4a**. In the case of α-aryldiazo ketones **1h** bearing two different aryl groups, the more electron-rich group Ar = 4-MeOC_6_H_4_ has a better migration ability, and the observed cyclopentenone is the preferred product.

In the TBAF-promoted pyrazole synthesis, two distinct regioisomers, **6a** and **9a**, can be synthesized with complete chemoselectivity. We postulate a siloxy deprotection of isolable intermediate **3a**, resulting in the formation of 1,3-diketone intermediate **E**. This intermediate undergoes keto–enol tautomerization to form its enol species **F**. Another resonating structure, **F′** can undergo 5-endo-dig cyclization to form species **G**. TBAF serves as a base for deprotonation of species **G**, forming diazenylmethanide **H**, in which the negative charge is located at the 1-C and 3-N atom positions. Reprotonation at species **H** is expected to give only one regioisomer **6a**. In a hot MeOH/NH_3_ solution, a 1,5-hydrogen shift is assisted by NH_3_ via coordination by the N-NH group; the rate is slow but irreversible to afford the other isomer **9a** due to its thermodynamic stability (Scheme 7).

## Data Availability

The data underlying this study are available in the published article and its online Supporting Information.

## Supporting Information Available

Scheme of substrate preparation, single-crystal X-ray data, and ^1^H NMR and ^13^C NMR spectra for representative compounds (PDF)
