# Supplementary material for: Two Distinct Diazo–Diazo Cross-Coupling Reactions between α-Aryldiazo Ketones and Vinyldiazo Esters Using Gold Catalyst and Tetrabutylammonium Fluoride
Source: Org Lett. 2025 Feb 19;27(8):1871–7. doi: 10.1021/acs.orglett.5c00068 (PMC11877532; doi:10.1021/acs.orglett.5c00068)

Supporting Information

Two Distinct Diazo-Diazo Cross-Coupling Reactions between α-Aryldiazo Ketones and Vinyldiazo Esters Using Gold Catalyst and Tetrabutylammonium Fluoride

Debashis Barik[a] and Rai-Shung Liu[a,b,*]

[a]Department of Chemistry, National Tsing-Hua University, Hsinchu 30013, Taiwan

[b]Department of Chemistry and College of Semiconductor Research Institute, National Tsing-Hua University, Hsinchu 30013, Taiwan; E-mail: rsliu@mx.nthu.edu.tw

Table of Contents:

Experimental Section

(1) **General Methods ……………………………………………………………………………S2**

(2) **General synthetic procedures for preparation of diazo ketones ……………………………S2**

(3) **General procedure for Synthesis of vinyl-diazo ester …………………………………………S3**

(4) **Standard Catalytic Reaction Procedure ……………………………………………………..S3**

4.1. Reaction Procedure for the Synthesis of **3a**…………………………………………**S4**

4.2. Reaction Procedure for the Synthesis of **5a and 5a'** from **3n and 3n'** ……………………....**S4**

4.3. Reaction Procedure for the synthesis of **6g** from **3n and 3n'** ………………………………**S5**

4.4. Standard Procedure of One-Pot Reaction for the Synthesis of **4** and **5**……………………...**S5**

4.5. Optimization Table of One-Pot Reaction for the Synthesis of Pyrazole **6a** (Table s1)……...**S6**

4.6. Standard Procedure of One-Pot Reaction for the Synthesis of **6** and **7**……………………...**S6**

4.7. Unsuccessful attempts with alkyl substituted diazo (**1''**) Substrate (Scheme s1)…………....**S7**

(5) **Procedure for Chemical Functionalizations ………………………………………………......S7**

(6) **References …………………………………………………………………………………....S9**

(7) **Spectral data for key compounds ……………………………………………………….........S9**

(8) **^{1}H-NOE data of compound (3n, 3n', 5c' and 8a)……………………………………………S32**

(9) **X-ray Crystallographic Structure and data for 5a, 6g, 8b and 9a…………………..............S33**

(10) **^{1}H and ^{13}C spectra of key compounds ………………………………………………………S38**

Experimental Section.

(1) General methods:

Unless otherwise noted, all the reactions to prepare the substrates were performed in oven-dried glassware under nitrogen atmosphere with freshly distilled solvents. The catalytic reactions were performed under nitrogen atmosphere. Toluene and DCE were distilled from CaH_2 under nitrogen. THF was distilled from Na metal under nitrogen. All other commercial reagents were used without further purification unless otherwise indicated. ^{1}H NMR and ^{13}C NMR spectra were recorded on a Varian 700 MHz and Bruker 400 MHz Spectrometers using chloroform-d ($CDCl_3$: δ_H = 7.24 ppm, δ_C = 77.00, CD_2Cl_2: δ_H = 5.32 ppm, δ_C = 54.00) as a solvent and Me_4Si as an internal standard. Chemical Shift (δ) and Spin-Spin coupling constant (J). The following abbreviations were used to show the multiplicities: s: singlet, bs: broad singlet, d: doublet, t: triplet, q: quadruplet, dd: doublet of doublet, quin: quintet, m: multiplet. Mass spectrometry was performed in the positive and negative electrospray ionization (ESI+ and ESI-) mode, positive electron ionization (EI+) mode, and positive field ionization (FI+) mode. The IR spectra were recorded using a Bruker Tensor 27 FT/IR spectrometer equipped with a KBr beam splitter and a DTGS detector. All heating reactions were carried out with an oil bath as the heat source. Reactions were magnetically stirred and monitored by thin layer chromatography carried out on 0.25 mm E. Merck silica gel plate (60f - 254) using UV light as visualizing agents. Single-crystal X-ray diffraction intensity data were collected on a Bruker X8 APEX diffractometer equipped with a CCD area detector and Mo Kα radiation (λ = 0.71073 Å) at 100 K; all data calculations were performed by using the PC version of the APEX2 program package. All the substrates α-diazo ketones and α-vinyldiazo esters were prepared according to the literature procedures, which are described below. Structural assignments were made with additional information from NOE, gHSQC, and gHMBC experiments.

(2) General synthetic procedures for preparation of symmetrical and non-symmetrical diazo ketone substrates:

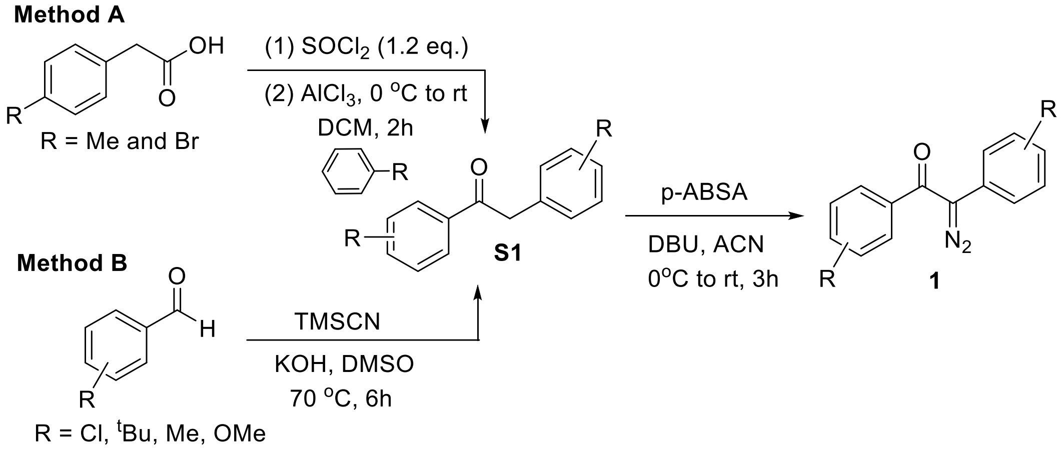

Method A:

To a DCM (60 ml) solution of substituted phenylacetic acid (**S1**) (1.0 equiv.) was added $SOCl_2$ (1.2 equiv.) dropwise at 0 °C. The mixture was warm to room temperature and stirred for 2 h. The resulting solution was cooled to 0 °C, followed by the addition of $AlCl_3$ (1.1 equiv.) and substituted benzene (1 equiv.). The mixture was stirred at room temperature for 3h. After completion of the reaction. The reaction was quenched with H_2O extracted with ethyl acetate (2 x 50 mL), and washed with brine (25 mL). The combined organic

layers were dried over $MgSO_4$, concentrated under reduced pressure, and purified by a silica column to afford symmetrically substituted 1,2-diphenylethan-1-one (**S1**) as a white solid.
S1b and S**1d** were prepared according to the method **A**.

Method B:
Using a nitrogen-filled glove box, an oven-dried sealed tube was charged with a magnetic stirring bar, KOH (1 mmol, 1.0 equiv.), substituted aldehyde (1.0 equiv.), and DMSO (6 ml). Then, the sealed tube was closed tightly with a rubber stopper, removed from the glove box and immersed in a preheated metal bath (60 °C). TMSCN (0.5 equiv.) was added to the mixture in two batches. After the reaction for 4 h, the solvent was evaporated under reduced pressure and the residue was purified by flash column chromatography (petroleum ether/ethyl acetate=100:1–10:1) on silica gel to give the substituted 1,2-diphenylethan-1-one (**S1**) as white solid **S1**. The spectral data were in agreement with those previously reported.[1]
S1c, S1e, S1f and S**1g** were prepared according to the method **B**.

To an acetonitrile solution of **S1** (1 equiv.) was added *p*-ABSA (1.1 equiv). The solution was cooled to 0 °C and DBU (1.3 equiv) was added dropwise to the above mixture and stirred at room temperature for 2 h. The reaction was quenched with H_2O, followed by extraction with ether (2 x 50 mL), and washed with brine (25 mL). The combined organic layers were dried over $MgSO_4$, concentrated under reduced pressure, and purified by a silica column (EA/Hexane = 15/85) to afford **1**.
All the symmetrically substituted and non-symmetrically substituted diazo-ketones **1** were synthesized according to the reported literatures. [2-3]

(3) General synthetic procedures for preparation of vinyl-diazo ester:

All α-diazo esters (**2b-2i**) were prepared from the reported procedure in the literature. [4]

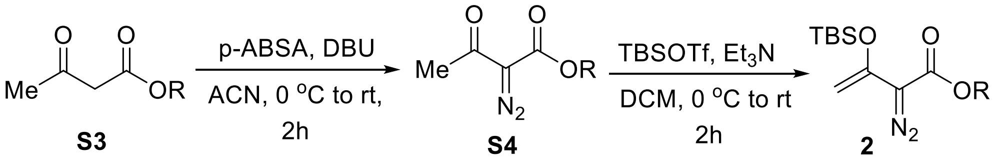

Under an argon atmosphere, in a 100 mL oven-dried round bottom flask containing a magnetic stirring bar, **S3** (1.0 equiv) and *p*-ABSA (1.1 equiv) in MeCN were added DBU (1.3 equiv) slowly at 0 ºC. After the reaction mixture was stirred for 2h, H_2O and DCM were added. The organic phase was separated and washed with saturated aqueous NaCl and dried over $MgSO_4$. After evaporating the solvents, diazo compounds **S4** were purified by flash chromatography (petroleum ether/ethyl acetate =20:1).
Under an argon atmosphere, in a 100 mL oven-dried round bottom flask containing a magnetic stirring bar, **S4** (1.0 equiv) and Et_3N (1.3 equiv) in 10 mL DCM was added TBSOTf (1.2 equiv) slowly at 0º C. After the reaction mixture was stirred for 1h under these conditions, saturated aqueous $NaHCO_3$ was added. The organic phase was separated and washed with brine and dried with anhydrous $MgSO_4$. After evaporating the solvents, vinyldiazo compounds **2** were directly used without further purification. ^{1}H and ^{13}C NMR spectra were recorded after the workup.

(4) Standard Catalytic Reaction Procedure:

4.1. General procedure Synthesis of ethyl 5-((tert-butyldimethylsilyl)oxy)-2-diazo-3-oxo-6,6-diphenylhex-5-enoate (3a).

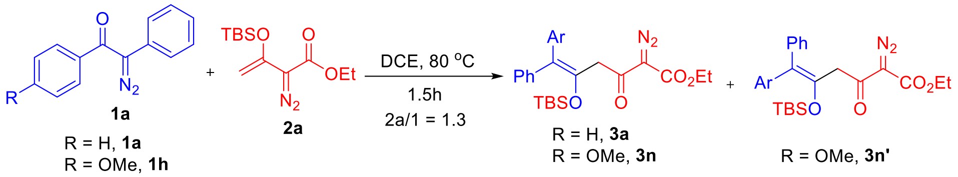

A Schlenk tube charged with freshly dry DCE (2 mL) and heated up to the 80°C in oil bath, then to that pre-heated solution of DCE, a solution of **1a** (88.9 mg, 0.4 mmol) and **2a** (140.6 mg, 0.52 mmol, 1.3 equiv.) in DCE (1.5 mL) was added dropwise at 80°C and resultant reaction mixture continue for 1.5h at same temperature. Reaction monitor by using TLC and after completion of reaction, solvent was removed under reduced pressure and eluted through a silica gel column with ethyl acetate/ hexane (3/97) to afford ethyl 5-((tert-butyldimethylsilyl)oxy)-2-diazo-3-oxo-6,6-diphenylhex-5-enoate **3a** (145.4 mg, 0.313 mmol, 78%) as a colorless oil.

The compounds **3n and 3n'** were synthesized using the same reaction procedure.

4.2. General Procedure Synthesis of ethyl 3-((tert-butyldimethylsilyl)oxy)-3-(4-methoxyphenyl)-5-oxo-2-phenylcyclopent-1-ene-1-carboxylate (5a) and ethyl 3-((tert-butyldimethylsilyl)oxy)-2-(4-methoxyphenyl)-5-oxo-3-phenylcyclopent-1-ene-1-carboxylate (5a') from 3n and 3n':

4.2.a. Synthesis of 5a and 5a' from 3n (Major isomer):

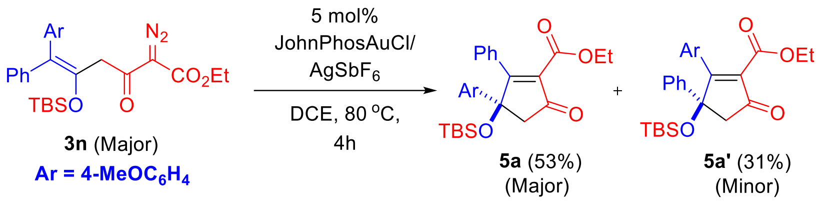

A schlenk tube was charged with JohnPhosAuCl (2.65 mg, 0.005 mmol) and $AgSbF_6$ (1.72 mg, 0.005 mmol) and to this mixture was added dry DCE (1.5 mL). The resulting mixture was stirred at room temperature for 5 minutes, and to this mixture was added a dry DCE solution (2.5 mL) of the major isomer **3n** (50.0 mg, 0.1 mmol) dropwise. After stirring at 80 °C temperature for 4 hours, the reaction mixture was filtered through a short celite bed and concentrated into crude products. The crude was purified by column chromatography on a silica column (5 % EA/hexane) and further separation with an HPLC instrument equipped with a silica column (Cosmosil 5SL-II, 15µm particle size) afforded the **5a** (25.1 mg, 0.054 mmol, 53%) and **5a'** (14.8 mg, 0.032 mmol, 31%) as sticky solid and colorless liquid respectively.

4.2.b. Synthesis of 5a and 5a' from 3n' (Minor isomer):

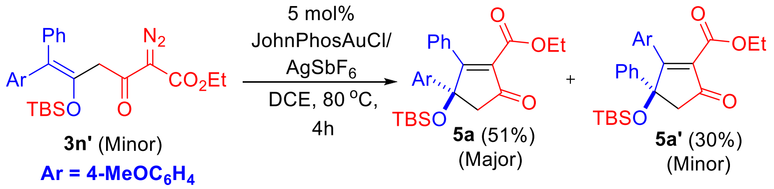

The minor isomer **3n'** (50.0 mg, 0.1 mmol) was treated with JohnPhosAuCl/$AgSbF_6$ under similar conditions as described in the case of major isomer **3n**. Two isomers **5a** (24.2 mg, 0.052 mmol, 51%) and **5a'** (14.2 mg, 0.03 mmol, 30%)**,** were isolated, respectively.

4.3. General procedure Synthesis of ethyl 3-(2,2-diphenylacetyl)-4-hydroxy-1H-pyrazole-5-carboxylate (6a) from 3n and 3n':

4.3.a. Synthesis of 6g from 3n (Major isomer):

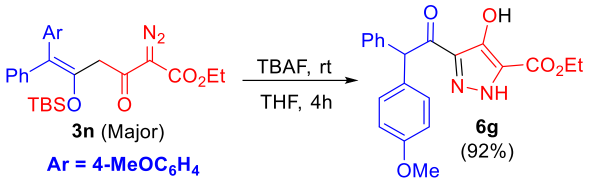

A Schlenk tube charged with freshly dry THF (2 mL) solution of the major isomer **3n** (50 mg, 0.1 mmol). To this solution was added a tetrabutylammonium fluoride (39.7 mg, 0.15 mmol, 1.5 equiv.) and the reaction was stirred at room temperature for 4 hours and was monitored by TLC. The reaction mixture was filtered through a short celite bed and concentrated to crude products of **6a**. The crude was purified by column chromatography on a silica column (20 % EA/hexane) afforded the desired ethyl 4-hydroxy-3-(2-(4-methoxyphenyl)-2-phenylacetyl)-1H-pyrazole-5-carboxylate **6g** (35.5 mg, 0.09 mmol, 92%) as white solid.

4.3.b. Synthesis of 6g from 3n' (Minor isomer):

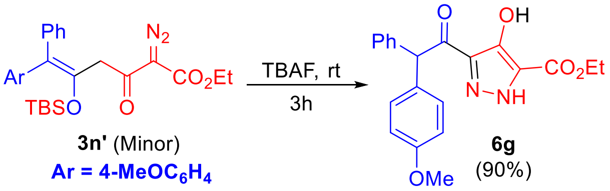

The minor isomer **3n'** (50.0 mg, 0.1 mmol) was treated with TBAF under similar conditions as described in the case of major isomer **3n**. Compound **6g** was afforded in 90% yield.

4.4. General procedure for the one-pot synthesis of ethyl-3-((tert-butyldimethylsilyl)oxy)-5-oxo-2,3-diphenylcyclopent-1-ene-1-carboxylate (4a).

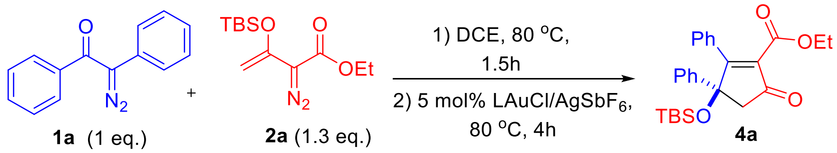

A Schlenk tube charged with freshly dry DCE (2 mL) and heated up to the 80°C in oil bath, then to that pre-heated solution of DCE, a solution of **1a** (88.9 mg, 0.4 mmol) and **2a** (140.6 mg, 0.52 mmol, 1.3 equiv.) in DCE (1.5 mL) was added dropwise at 80°C and resultant reaction mixture continue for 1.5h at same temperature. The reaction was monitored by TLC. After completion of starting material **1a**, it cooled down to room temperature and was added JohnPhosAuCl (10.6 mg, 0.02 mmol) and $AgSbF_6$ (6.9 mg, 0.02 mmol) to the reaction mixture under the N_2 atmosphere and further stirred for 4h at 80 °C. The reaction mixture was filtered through a short celite bed and concentrated into crude products **4a**. The crude was purified by column chromatography on a silica column (5 % EA/hexane) afforded the desired ethyl-3-((tert-butyldimethylsilyl)oxy)-5-oxo-2,3-diphenylcyclopent-1-ene-1-carboxylate **4a** (114.0 mg, 0.26 mmol, 65%) as a colorless liquid. All the other products **4** and **5** were prepared similarly.

4.5. Table s1: Optimization Table of One-Pot Reaction for the synthesis of Pyrazole (6a).

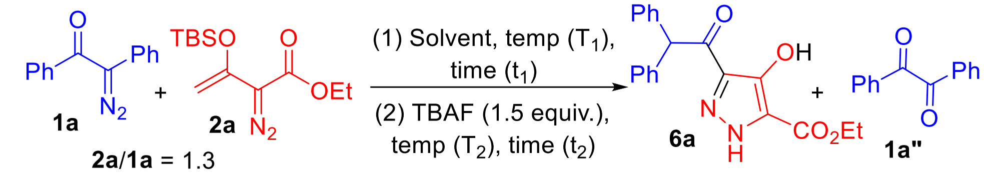

entry	Catalyst	solvent	temp (°C) T_1/T_2	time (t_1/t_2)	yield[b] 6a	1a"
1)	None	DCE	80/rt	2h/4h	65	--
2)	None	toluene	100/rt	1.5h/4h	61	--
3)	None	$C_6H_5CF_3$	80/rt	2h/4h	54	--
**4)**	**None**	**THF**	**65/rt**	**1.5h/3h**	**68**	--
5)	None	C_6H_6	80/rt	2.0h/4h	55	--
6)	None	DCM	50/rt	2.5h/3h	58	--
7)	None	CH_3CN	80/rt	4h/4h	50	--
8)[c]	LAuCl/AgNTf$_2$ (5 mol%)	DCE	80/rt	10h/6h	--	62
9)	AgNTf$_2$ (5 mol%)	DCE	80/rt	10h/6h	--	57

[a] **1a** (0.4 mmol) **2a** (0.52 mmol) in solvent (5 mL) under N_2 atmosphere. [b] The product yields were calculated after column chromatography on silica gel. [c] L= P(*t*-Bu)$_2$(*o*-biphenyl). TBAF = Tetra-n-butylammonium fluoride.

4.6. General procedure for the one-pot synthesis of ethyl 3-(2,2-diphenylacetyl)-4-hydroxy-1H-pyrazole-5-carboxylate (6a).

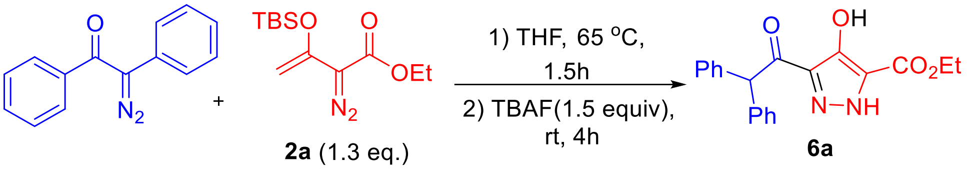

A Schlenk tube charged with freshly dry THF (2 mL) and heated up to the 65 °C in oil bath, then to that pre-heated solution of THF, a solution of **1a** (88.9 mg, 0.4 mmol) and **2a** (140.6 mg, 0.52 mmol, 1.3 equiv.) in THF (1.5 mL) was added dropwise at 65 °C and resultant reaction mixture continue for 1.5h at same temperature. The reaction was monitored by TLC. After completion of starting material **1a**, it cooled down to room temperature and was added tetrabutylammonium fluoride (157.0 mg, 1.5 equiv.) to the reaction mixture under the N_2 atmosphere and further stirred for 4h at room temperature. The reaction mixture was filtered through a short celite bed and concentrated into crude products **6a**. The crude was purified by column chromatography on a silica column (20 % EA/hexane) afforded the desired ethyl 3-(2,2-diphenylacetyl)-4-hydroxy-1H-pyrazole-5-carboxylate **6a** (95.5 mg, 0.28 mmol, 68%) as white solid. All the other products **6** and **7** were prepared similarly.

4.7. Scheme s1: Unsuccessful attempts with alkyl substituted diazo (1") Substrate:

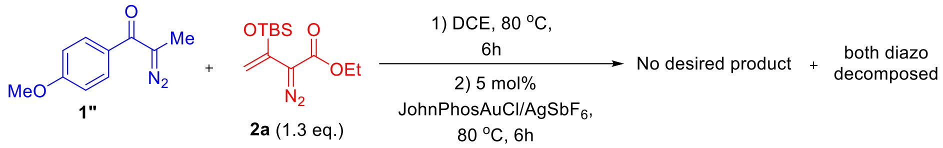

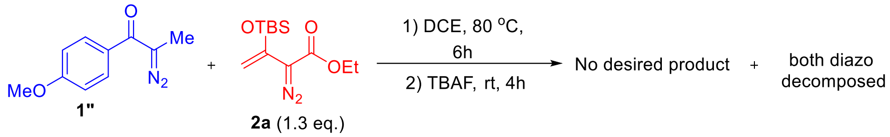

(5) Standard Procedure for Chemical Functionalization:

5.1. Standard Procedure for the Scale-up Reaction:

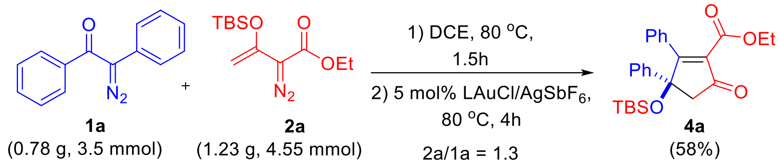

A scale-up reaction was performed between 2-diazo-1,2-diphenylethan-1-one **1a** (0.78 g, 3.5 mmol) and ethyl 3-((tert-butyldimethylsilyl)oxy)-2-diazobut-3-enoate **2a** (1.23 g, 4.55 mmol) in dichloroethane (DCE, 15.0 mL) under the standard conditions to afford **4a** (0.89 g, 58 %) as colorless liquid.

5.2. Synthesis of ethyl-3-((tert-butyldimethylsilyl)oxy)-5-hydroxy-2,3-diphenylcyclopent-1-ene-1-carboxylate (8a).

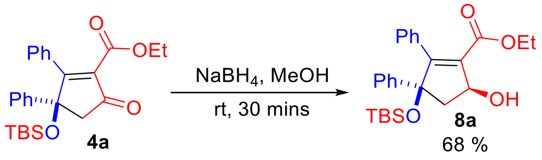

To a MeOH solution of **4a** (50 mg, 0.115 mmol) was added $NaBH_4$ (5.5 mg, 0.137 mmol). The reaction mixture was stirred at rt for 30 mins, after which time it was quenched with H_2O. Then, the solvent was removed under reduced pressure and was extracted with EtOAc. The layers were separated and the organic layer was dried over $MgSO_4$ and concentrated under reduced pressure. The crude was purified by column chromatography over silica gel with 10% EtOAc/Hexane to afford **8a** (34 mg, 0.078 mmol, 68%) as a colorless liquid.

5.3. Synthesis of ethyl 3-hydroxy-3-(4-methoxyphenyl)-5-oxo-2-phenylcyclopent-1-ene-1-carboxylate (8b).

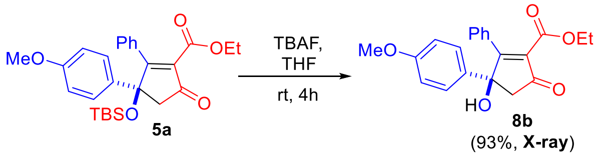

In an oven-dried catalytic tube equipped with a magnetic stirring bar, to a THF (3 mL) solution of **5a** (50 mg, 0.107 mmol) was added tetrabutylammonium fluoride (42.0 mg, 0.16 mmol, 1.5 equiv.) and the

reaction was stirred at room temperature for 4 hours. Upon completion of the reaction, the reaction mixture was extracted with DCM and brine solution. The organic layer was dried with $MgSO_4$ and concentrated under reduced pressure and purified by column chromatography over silica gel with 15% EtOAc/Hexane to afford **8b** (35 mg, 0.1 mmol, 93%) as a white solid.

5.4. Synthesis of methyl 5-(2,2-diphenylacetyl)-4-hydroxy-1H-pyrazole-3-carboxylate (9a).

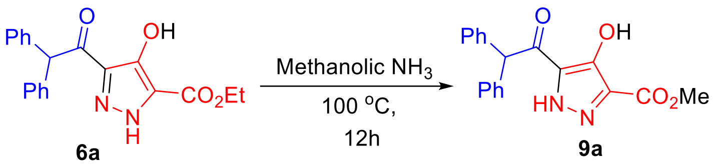

In an oven-dried catalytic tube equipped with a magnetic stirring bar, the compound **6a** (50.0 mg, 0.143 mmol) was treated with ammonia ca. 7N solution in methanol (4 ml). The reaction mixture was stirred at 100 °C for 12 hours. Upon completion of the reaction, the solvent was removed under reduced pressure the reaction mixture was extracted with DCM and brine solution. The organic layer was dried with $MgSO_4$ and concentrated under reduced pressure and purified by column chromatography over silica gel with 25% EtOAc/Hexane to afford **9a** (43.3 mg, 0.128 mmol, 90%) as a white solid.

5.5. Synthesis of ethyl 3-(2,2-diphenylacetyl)-4-methoxy-1-methyl-1H-pyrazole-5-carboxylate (9b).

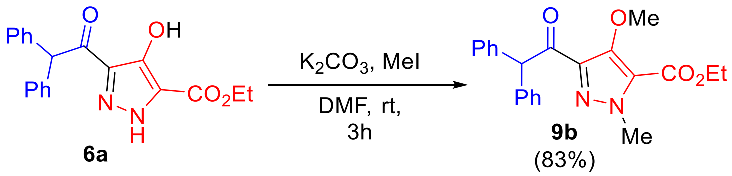

A catalytic tube was charged with **6a** (35 mg, 0.1 mmol, 1.0 equiv.), K_2CO_3 (27.6 mg, 0.8 mmol, 2.0 equiv.) and DMF (5 mL) stirred for 5 mins. Methyl iodide (28.4 mg, 0.2 mmol, 2.0 equiv.) was added and the reaction was stirred at room temperature for 3 hours. Then the reaction mixture was extracted with EtOAc and dried over anhydrous $MgSO_4$ and concentrated under reduced pressure and purified by column chromatography over silica gel with 10% EtOAc/Hexane to afford **9b** (31.3 mg, 0.083 mmol, 83%) as a colorless liquid.

5.6. Synthesis of methyl 5-(2,2-diphenylacetyl)-4-methoxy-1-methyl-1H-pyrazole-3-carboxylate (9c).

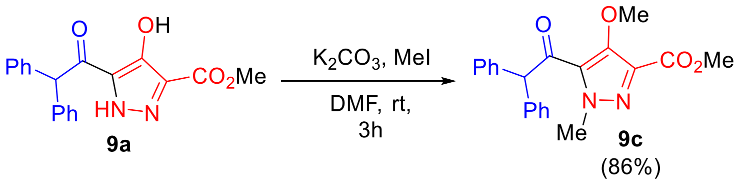

A catalytic tube was charged with **9a** (35 mg, 0.104 mmol, 1.0 equiv.), K_2CO_3 (29 mg, 0.21 mmol, 2 equiv.) and DMF (5 mL) stirred for 5 mins. Methyl iodide (30 mg, 0.21 mmol, 2.0 equiv.) was added and the reaction was stirred at room temperature for 3 hours. Then the reaction mixture was extracted with EtOAc and dried over anhydrous $MgSO_4$ and concentrated under reduced pressure and purified by column chromatography over silica gel with 10% EtOAc/Hexane to afford **9c** (32.6 mg, 0.089 mmol, 86%) as a colorless liquid.

(6) References:

(1) Zhang, G.; Liang, Q.; Yang, W.; Jiang, S.; Wang, Z.; Zhang, C.; Zhang, G. *Adv. Synth. Catal.* **2022**, *364*, 2951 – 2956.
(2) (a) Sasane, A. V.; Liu, R.-S. *Chem. Commun.* **2023**, *59*, 1086– 1089. (b) More, S. A.; Sadaphal, V. A.; Kuo, T.-C.; Cheng, M.-J.; Liu, R.-S. *Chem. Commun.* **2022**, *58*, 10064– 10067.
(3) (a) Xu, B.; Zhu, S. F.; Zuo, X. D.; Zhang, Z. C.; Zhou, Q. L. *Angew. Chem., Int. Ed.* **2014**, *53*, 3913– 3916. (b) Chen, C. N.; Cheng, W. M.; Wang, J. K.; Chao, T. H.; Cheng, M. J.; Liu, R. S. *Angew. Chem., Int. Ed.* **2021**, *60*, 4479– 4484.
(4) (a) Shved, A.; Tabolin, A.; Novikov, R.; Nelyubina, Y.; Timofeev, V.; Toffe, S. *Eur. J. Org. Chem.* **2016**, *2016*, 5569– 5578. (b) Zhu, C.; Xu, G.; Sun, J. *Angew. Chem., Int. Ed. Engl.* **2016**, *55*, 11867– 11871 (c) Szczepanik, P.M.; Mikhaylov, A. A.; Hylse, O.; Kučera, R.; Daďová, P.; Nečas, M.; Kubala, L.; Paruch, K.; Švenda, J. *Angew. Chem. Int. Ed.* **2023**, *62*, e202213183.

(7) Spectral data for key compounds:

Spectral data for 2-diazo-1,2-diphenylethan-1-one (1a):

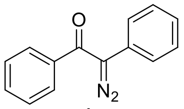

1a

Prepared from **S1a** (1.96 g, 10 mmol, 1 equiv.) by the general procedure for diazo preparation. Purified by silica gel column, hexane/ethyl acetate (90:10) as eluent; yellow solid (1.25 g, 5.62 mmol, 70%); ^{1}H NMR (400 MHz, $CDCl_3$): δ 7.61 – 7.58 (m, 2H), 7.50 – 7.44 (m, 3H), 7.42 – 7.36 (m, 4H), 7.26 – 7.22 (m, 1H); ^{13}C NMR (100 MHz, $CDCl_3$): δ 188.2, 137.8, 131.6, 128.9, 128.4, 127.6, 126.9, 126.0, 125.9, 72.9; HRMS (ESI-TOF)$^+$ calcd for $C_{14}H_{11}N_2O$ [M+H]$^+$: 223.0871, found: 223.0878.

Spectral data for 2-diazo-1,2-di-p-tolylethan-1-one (1b):

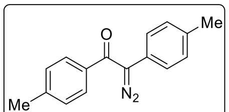

1b

Prepared from **S1b** (1.12 g, 5.0 mmol, 1 equiv.) by the general procedure for diazo preparation. Purified by silica gel column, hexane/ethyl acetate (90:10) as eluent; yellow solid (0.65 g, 2.60 mmol, 52%); ^{1}H NMR (400 MHz, $CDCl_3$): δ 7.51 – 7.49 (m, 2H), 7.34 – 7.31 (m, 2H), 7.20 – 7.17 (m, 4H), 2.37 (s, 3H), 2.34 (s, 3H); ^{13}C NMR (100 MHz, $CDCl_3$): δ 188.2, 142.1, 136.8, 135.2, 129.6, 128.9, 127.8, 126.1, 123.0, 72.4, 21.4, 21.0; HRMS (ESI-TOF)$^+$ calcd for $C_{16}H_{14}N_2ONa$ [M+Na]$^+$: 273.1003, found: 273.1008.

Spectral data for 1,2-bis(4-(tert-butyl)phenyl)-2-diazoethan-1-one (1c):

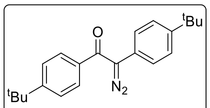

1c

Prepared from **S1c** (1.54 g, 5.0 mmol, 1 equiv.) by the general procedure for diazo preparation. Purified by silica gel column, hexane/ethyl acetate (90:10) as eluent; yellow liquid (0.6 g, 1.8 mmol, 35%); ^{1}H NMR

(400 MHz, $CDCl_3$): δ 7.89 – 7.87 (m, 4H), 7.50 – 7.48 (m, 4H), 1.32 (s, 18H); ^{13}C NMR (100 MHz, $CDCl_3$): δ 194.5, 158.8, 130.6, 129.9, 125.9, 35.3, 30.9 (diazo carbon is missing due to dilute sample); Mass didn't found in ESI/EI HRMS.

Spectral data for 1,2-bis(4-bromophenyl)-2-diazoethan-1-one (1d):

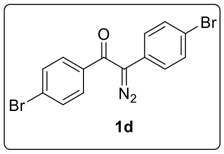

Prepared from **S1d** (1.77 g, 5.0 mmol, 1 equiv.) by the general procedure for diazo preparation. Purified by silica gel column, hexane/ethyl acetate (85:15) as eluent; yellow solid (1.05 g, 2.76 mmol, 55%); ^1H NMR (400 MHz, $CDCl_3$): δ 7.56 – 7.44 (m, 6H), 7.32 (d, J = 8.4 Hz, 2H); ^{13}C NMR (100 MHz, $CDCl_3$): δ 186.7, 136.3, 132.2, 131.9, 129.3, 127.4, 126.6, 124.9, 121.0 (diazo carbon is missing due to dilute sample); Mass didn't found in ESI/EI HRMS.

Spectral data for 1,2-bis(4-chlorophenyl)-2-diazoethan-1-one (1e):

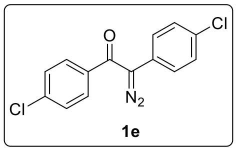

Prepared from **S1e** (1.33 g, 5.0 mmol, 1 equiv.) by the general procedure for diazo preparation. Purified by silica gel column, hexane/ethyl acetate (90:10) as eluent; yellow solid (0.74 g, 2.52 mmol, 50%); ^1H NMR (400 MHz, $CDCl_3$): δ 7.54 – 7.51 (m, 2H), 7.39 – 7.36 (m, 6H); ^{13}C NMR (100 MHz, $CDCl_3$): δ 186.7, 138.1, 135.9, 133.0, 129.3, 129.2, 128.9, 127.2, 124.4, 72.6; Mass didn't found in ESI/EI HRMS.

Spectral data for 2-diazo-1,2-bis(3-methoxyphenyl)ethan-1-one (1g):

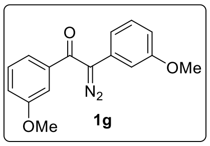

Prepared from **S1g** (1.28 g, 5.0 mmol, 1 equiv.) by the general procedure for diazo preparation. Purified by silica gel column, hexane/ethyl acetate (90:10) as eluent; yellow solid (0.64 g, 2.27 mmol, 45%); ^1H NMR (400 MHz, $CDCl_3$): δ 7.32 – 7.27 (m, 2H), 7.17 – 7.12(m, 3H), 7.03 – 6.96 (m, 2H), 6.80 – 6.77 (m, 1H), 3.79 – 3.78 (m, 6H); ^{13}C NMR (100 MHz, $CDCl_3$): δ 188.0, 160.0, 159.6, 139.2, 129.9, 129.5, 127.4, 119.9, 118.0, 117.9, 112.7, 112.5, 111.5, 73.1, 55.3, 55.2; HRMS (ESI-TOF)$^+$ calcd for $C_{16}H_{14}N_2O_3Na$ $[M+Na]^+$: 305.0902, found: 305.0899.

Spectral data for ethyl 3-((tert-butyldimethylsilyl)oxy)-2-diazobut-3-enoate (2a):

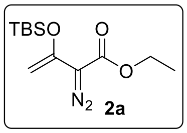

Prepared from **S4a** (1.56 g, 10.0 mmol, 1 equiv.) by the general procedure for diazo preparation. Yellow liquid (2.47 g, 9.13 mmol, 91%); ^1H NMR (400 MHz, $CDCl_3$): δ 4.96 (d, J = 2.0 Hz, 1H), 4.24 – 4.18 (m, 3H), 1.25 (t, J = 7.2 Hz, 3H), 0.87 (s, 9H), 0.18 (s, 6H); ^{13}C NMR (100 MHz, $CDCl_3$): δ 164.2, 140.8, 90.3,

66.1, 60.6, 25.5, 18.0, 14.3, -4.8; HRMS (ESI-TOF)$^-$ calcd for $C_{12}H_{21}N_2O_3Si$ [M-H]$^-$: 269.1321, found: 269.1322.

Spectral data for isopropyl 3-((tert-butyldimethylsilyl)oxy)-2-diazobut-3-enoate (2b):

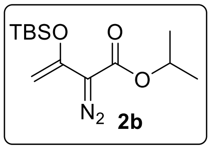

Prepared from **S4b** (0.34 g, 2.0 mmol, 1 equiv.) by the general procedure for diazo preparation. Yellow liquid (0.51 mg, 1.79 mmol, 89%); ^{1}H NMR (400 MHz, $CDCl_3$): δ 5.14 – 5.05 (m, 1H), 4.97 (d, *J* = 2.0 Hz, 1H), 4.20 (d, *J* = 2.0 Hz, 1H), 1.25 (d, *J* = 6.0 Hz, 6H), 0.89 (s, 9H), 0.19 (s, 6H); ^{13}C NMR (100 MHz, $CDCl_3$): δ 163.8, 140.9, 90.2, 68.3, 66.3, 25.5, 21.9, 18.0, -4.8; HRMS (ESI-TOF)$^+$ calcd for $C_{13}H_{24}N_2O_3SiNa$ [M+Na]$^+$: 307.1454, found: 307.1455.

Spectral data for benzyl 3-((tert-butyldimethylsilyl)oxy)-2-diazobut-3-enoate (2c):

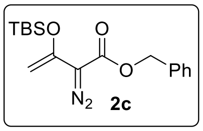

Prepared from **S4c** (0.44 g, 2.0 mmol, 1 equiv.) by the general procedure for diazo preparation. Yellow liquid (0.57 g, 1.7 mmol, 85%); ^{1}H NMR (400 MHz, $CDCl_3$): δ 7.35 – 7.30 (m, 5H), 5.22 (s, 2H), 4.99 (d, *J* = 2.0 Hz, 1H), 4.23 (d, *J* = 2.0 Hz, 1H), 0.89 (s, 9H), 0.19 (s, 6H); ^{13}C NMR (100 MHz, $CDCl_3$): δ 164.1, 140.6, 135.8, 128.5, 128.2, 128.0, 90.4, 66.2, 25.5, 18.0, (Si-C carbon at negative δ ppm couldn‘t be recorded and diazo carbon is missing due to dilute sample); HRMS (ESI-TOF)$^+$ calcd for $C_{17}H_{24}N_2O_3SiNa$ [M+Na]$^+$: 353.1297, found: 353.1301.

Spectral data for butyl 3-((tert-butyldimethylsilyl)oxy)-2-diazobut-3-enoate (2d):

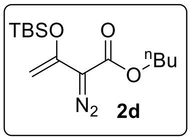

Prepared from **S4d** (0.37 g, 2.0 mmol, 1 equiv.) by the general procedure for diazo preparation. Yellow liquid (0.49 g, 1.64 mmol, 82%); ^{1}H NMR (400 MHz, $CDCl_3$): δ 4.97 (d, *J* = 2.0 Hz, 1H), 4.20 – 4.15 (m, 3H), 1.64 – 1.57 (m, 2H), 1.40 – 1.32 (m, 2H), 0.92 – 0.87 (m, 12H), 0.19 (s, 6H); ^{13}C NMR (175 MHz, $CDCl_3$): δ 164.2, 140.7, 90.2, 65.1, 64.5, 30.7, 25.4, 18.9, 17.9, 13.5, -4.9; HRMS (ESI-TOF)$^+$ calcd for $C_{14}H_{26}N_2O_3SiNa$ [M+Na]$^+$: 321.1610, found: 321.1609.

Spectral data for tert-butyl 3-((tert-butyldimethylsilyl)oxy)-2-diazobut-3-enoate (2e):

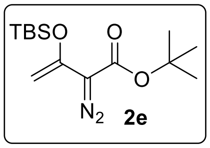

Prepared from **S4e** (0.37 g, 2.0 mmol, 1 equiv.) by the general procedure for diazo preparation. Yellow liquid (0.48 g, 1.59 mmol, 79%); ^{1}H NMR (400 MHz, $CDCl_3$): δ 4.93 (d, *J* = 2.0 Hz, 1H), 4.18 (d, *J* = 2.0 Hz, 1H), 1.46 (s, 9H), 0.88 (s, 9H), 0.18 (s, 6H); ^{13}C NMR (100 MHz, $CDCl_3$): δ 163.6, 141.3, 90.0, 81.7,

66.7, 28.3, 25.5, 18.0, -4.8; HRMS (ESI-TOF)$^+$ calcd for $C_{14}H_{26}N_2O_3SiNa$ [M+Na]$^+$: 321.1610, found: 321.1611.

Spectral data for cyclohexyl 3-((tert-butyldimethylsilyl)oxy)-2-diazobut-3-enoate (2f):

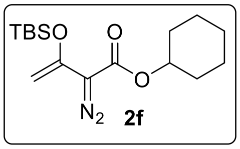

Prepared from **S4f** (0.42 g, 2.0 mmol, 1 equiv.) by the general procedure for diazo preparation. Yellow liquid (0.49 g, 1.51 mmol, 75%); ^{1}H NMR (400 MHz, $CDCl_3$): δ 4.97 (d, J = 2.0 Hz, 1H), 4.90 – 4.84 (m, 1H), 4.20 (d, J = 2.0 Hz, 1H), 1.84 – 1.80 (m, 2H), 1.70 – 1.65 (s, 2H), 1.50 – 1.40 (m, 3H), 1.39 – 1.22 (m, 3H), 0.88 (s, 9H), 0.19 (s, 6H); ^{13}C NMR (100 MHz, $CDCl_3$): δ 163.7, 140.9, 90.1, 72.9, 31.6, 25.6, 25.5, 25.2, 23.4, 18.0, (Si-C carbon at negative δ ppm couldn't be recorded and diazo carbon is missing due to dilute sample); HRMS (ESI-TOF)$^+$ calcd for $C_{16}H_{28}N_2O_3SiNa$ [M+Na]$^+$: 347.1767, found: 347.1764.

Spectral data for allyl 3-((tert-butyldimethylsilyl)oxy)-2-diazobut-3-enoate (2g):

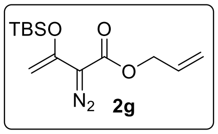

Prepared from **S4g** (0.34 g, 2.0 mmol, 1 equiv.) by the general procedure for diazo preparation. Yellow liquid (0.44 g, 1.58 mmol, 78%); ^{1}H NMR (400 MHz, $CDCl_3$): δ 5.95 – 5.85 (m, 1H), 5.34 – 5.26 (m, 1H), 5.23 – 5.20 (m, 1H), 4.97 (d, J = 2.0 Hz, 1H), 4.69 – 4.66 (m, 2H), 4.22 (d, J = 2.0 Hz, 1H), 0.88 (s, 9H), 0.19 (s, 6H); ^{13}C NMR (100 MHz, $CDCl_3$): δ 163.8, 140.6, 132.0, 118.2, 90.4, 65.8, 65.1, 25.5, 18.0, (Si-C carbon at negative δ ppm couldn't be recorded); HRMS (ESI-TOF)$^+$ calcd for $C_{13}H_{22}N_2O_3SiNa$ [M+Na]$^+$: 305.1297, found: 305.1295.

Spectral data for methyl 3-((tert-butyldimethylsilyl)oxy)-2-diazobut-3-enoate (2h):

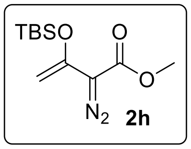

Prepared from **S4h** (0.28 g, 2.0 mmol, 1 equiv.) by the general procedure for diazo preparation. Yellow liquid (0.47 g, 1.83 mmol, 91%); ^{1}H NMR (400 MHz, $CDCl_3$): δ 4.97 (d, J = 2.0 Hz, 1H), 4.22 (d, J = 2.0 Hz, 1H), 3.76 (s, 3H), 0.88 (s, 9H), 0.19 (s, 6H); ^{13}C NMR (100 MHz, $CDCl_3$): δ 164.6, 140.7, 90.4, 51.7, 25.5, 18.0, (Si-C carbon at negative δ ppm couldn't be recorded and diazo carbon is missing due to dilute sample); HRMS (ESI-TOF)$^+$ calcd for $C_{11}H_{21}N_2O_3Si$ [M+H]$^+$: 257.1321, found: 257.1322.

Spectral data for ethyl 5-((tert-butyldimethylsilyl)oxy)-2-diazo-3-oxo-6,6-diphenylhex-5-enoate (3a):

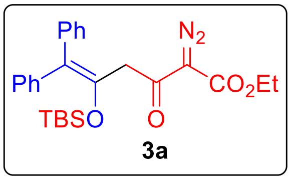

Purified by silica gel column, hexane/ethyl acetate (95:5) as eluent; Colorless liquid (145.4 mg, 78%); ^{1}H NMR (700 MHz, CDCl$_3$): δ 7.28 – 7.27 (m, 2H), 7.24 – 7.19 (m, 4H), 7.17 – 7.14 (m, 3H), 7.11 (t, *J* = 7.0 Hz, 1H), 4.23 (q, *J* = 7.0 Hz, 2H), 3.77 (s, 2H), 1.26 (t, *J* = 7.0 Hz, 3H), 0.73 (s, 9H), -0.14 (s, 6H); ^{13}C NMR (175 MHz, CDCl$_3$): δ 188.6, 161.1, 142.6, 141.6, 140.5, 130.4, 130.1, 128.1, 127.6, 126.8, 126.5, 126.1, 75.9, 61.3, 45.7, 25.6, 18.1, 14.2, -4.4; HRMS (ESI-TOF)$^+$ calcd for $C_{26}H_{32}N_2O_4SiNa$ [M+Na]$^+$: 487.2029, found: 487.2024.

Spectral data for ethyl (E)-5-((tert-butyldimethylsilyl)oxy)-2-diazo-6-(4-methoxyphenyl)-3-oxo-6-phenylhex-5-enoate (3n):

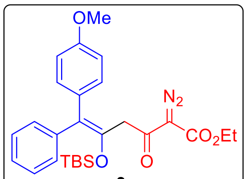

3n

Purified by silica gel column, hexane/ethyl acetate (95:5) as eluent; Colorless liquid (95.0 mg, 48%); ^{1}H NMR (700 MHz, CDCl$_3$): δ 7.27 (d, *J* = 7.7 Hz, 2H), 7.20 (t, *J* = 7.7 Hz, 2H), 7.10 (t, *J* = 7.0 Hz, 1H), 7.07 (d, *J* = 8.4 Hz, 2H), 6.77 (d, *J* = 8.4 Hz, 2H), 4.24 (q, *J* = 7.0 Hz, 2H), 3.77 (s, 2H), 3.75 (s, 3H), 1.27 (t, *J* = 7.0 Hz, 3H), 0.73 (s, 9H), -0.15 (s, 6H); ^{13}C NMR (175 MHz, CDCl$_3$): δ 188.7, 161.1, 158.3, 142.4, 140.7, 134.0, 131.2, 130.4, 127.6, 126.2, 126.0, 113.5, 75.9, 61.4, 55.1, 45.8, 25.6, 18.1, 14.2, -4.5; HRMS (ESI-TOF)$^+$ calcd for $C_{27}H_{34}N_2O_5SiNa$ [M+Na]$^+$: 517.2135, found: 517.2134.

Spectral data for ethyl (Z)-5-((tert-butyldimethylsilyl)oxy)-2-diazo-6-(4-methoxyphenyl)-3-oxo-6-phenylhex-5-enoate (3n'):

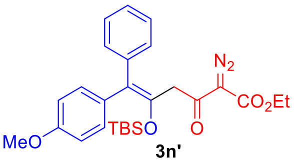

3n'

Purified by silica gel column, hexane/ethyl acetate (95:5) as eluent; Colorless liquid (58.0 mg, 29%); ^{1}H NMR (700 MHz, CDCl$_3$): δ 7.24 – 7.19 (m, 4H), 7.16 (t, *J* = 7.0 Hz, 2H), 7.14 – 7.13 (m, 2H), 6.76 (d, *J* = 8.4 Hz, 2H), 4.23 (q, *J* = 7.0 Hz, 2H), 3.75 (s, 5H), 1.26 (t, *J* = 7.0 Hz, 3H), 0.76 (s, 9H), -0.12 (s, 6H); ^{13}C NMR (175 MHz, CDCl$_3$): δ 188.7, 161.1, 157.9, 142.0, 141.9, 133.0, 131.5, 130.1, 128.1, 126.4, 126.3, 113.0, 61.3, 55.2, 45.7, 25.7, 18.1, 14.3, -4.4 (diazo carbon is missing due to dilute sample); HRMS (ESI-TOF)$^+$ calcd for $C_{27}H_{34}N_2O_5SiNa$ [M+Na]$^+$: 517.2135, found: 517.2127.

Spectral data for ethyl 3-((tert-butyldimethylsilyl)oxy)-5-oxo-2,3-diphenylcyclopent-1-ene-1-carboxylate (4a):

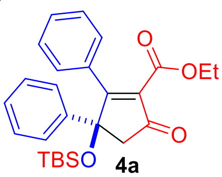

4a

Purified by silica gel column, hexane/ethyl acetate (95:5) as eluent; Colorless liquid (114.0 mg, 65%); ^{1}H NMR (700 MHz, CDCl$_3$): δ 7.41 (d, *J* = 7.7 Hz, 2H), 7.33 – 7.29 (m, 3H), 7.25 – 7.24 (m, 1H), 7.20 (s, 4H),

4.29 – 4.24 (m, 2H), 3.15 (d, *J* = 18.2 Hz, 1H), 2.96 (d, *J* = 18.2 Hz, 1H), 1.28 (t, *J* = 7.0 Hz, 3H), 0.82 (s, 9H), 0.12 (s, 3H), -0.17 (s, 3H); ^{13}C NMR (175 MHz, $CDCl_3$): δ 199.6, 173.6, 164.2, 144.7, 133.9, 131.5, 130.6, 129.7, 128.7, 128.0, 127.44, 124.49, 82.5, 61.5, 55.1, 25.7, 18.4, 13.8, -2.5, -3.3; HRMS (ESI-TOF)$^+$ calcd for $C_{26}H_{33}O_4Si$ [M+H]$^+$: 437.2149, found: 437.2148.

Spectral data for ethyl 3-((tert-butyldimethylsilyl)oxy)-5-oxo-2,3-di-p-tolylcyclopent-1-ene-1-carboxylate (4b):

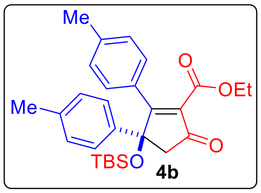

Purified by silica gel column, hexane/ethyl acetate (95:5) as eluent; Colorless liquid (125.0 mg, 67%); ^{1}H NMR (700 MHz, $CDCl_3$): δ 7.28 (d, *J* = 8.4 Hz, 2H), 7.18 (d, *J* = 8.4 Hz, 2H), 7.11 (d, *J* = 8.4 Hz, 2H), 7.01 (d, *J* = 8.4 Hz, 2H), 4.31 – 4.26 (m, 2H), 3.11 (d, *J* = 18.2 Hz, 1H), 2.89 (d, *J* = 18.2 Hz, 1H), 2.30 (s, 3H), 2.26 (s, 3H), 1.22 (t, *J* = 7.0 Hz, 3H), 0.83 (s, 9H), 0.11 (s, 3H), -0.20 (s, 3H); ^{13}C NMR (175 MHz, $CDCl_3$): δ 199.8, 173.2, 164.7, 142.2, 141.2, 136.9, 133.2, 130.0, 129.4, 128.7, 128.6, 124.3, 82.6, 61.5, 55.3, 25.7, 21.4, 20.9, 18.4, 13.9, -2.6, -3.3; HRMS (ESI-TOF)$^+$ calcd for $C_{28}H_{36}NaO_4Si$ [M+Na]$^+$: 487.2280, found: 487.2282.

Spectral data for ethyl 2,3-bis(4-(tert-butyl)phenyl)-3-((tert-butyldimethylsilyl)oxy)-5-oxocyclopent-1-ene-1-carboxylate (4c):

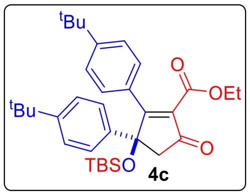

Purified by silica gel column, hexane/ethyl acetate (95:5) as eluent; Colorless liquid (118.7 mg, 54%); ^{1}H NMR (700 MHz, $CDCl_3$): δ 7.37 – 7.34 (m, 4H), 7.23 (d, *J* = 8.4 Hz, 2H), 7.17 (d, *J* = 8.4 Hz, 2H), 4.32 – 4.26 (m, 2H), 3.11 (d, *J* = 18.2 Hz, 1H), 2.89 (d, *J* = 18.2 Hz, 1H), 1.29 (s, 9H), 1.23 – 1.21 (m, 12H), 0.75 (s, 9H), 0.11 (s, 3H), -0.27 (s, 3H); ^{13}C NMR (175 MHz, $CDCl_3$): δ 199.6, 173.7, 164.6, 154.4, 150.2, 142.2, 133.1, 130.0, 128.5, 125.7, 125.0, 123.9, 82.4, 61.5, 55.5, 34.8, 34.4, 31.3, 30.9, 25.6, 18.4, 13.9, -2.64, -3.58; HRMS (ESI-TOF)$^+$ calcd for $C_{34}H_{48}NaO_4Si$ [M+Na]$^+$: 571.3219, found: 571.3214.

Spectral data for ethyl 2,3-bis(4-bromophenyl)-3-((tert-butyldimethylsilyl)oxy)-5-oxocyclopent-1-ene-1-carboxylate (4d):

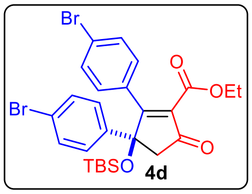

Purified by silica gel column, hexane/ethyl acetate (95:5) as eluent; Colorless liquid (112.0 mg, 47%); ^{1}H NMR (700 MHz, $CDCl_3$): δ 7.44 (d, *J* = 8.4 Hz, 2H), 7.37 (d, *J* = 8.4 Hz, 2H), 7.22 (d, *J* = 8.4 Hz, 2H), 7.11 (d, *J* = 8.4 Hz, 2H), 4.29 – 4.25 (m, 2H), 3.13 (d, *J* = 18.2 Hz, 1H), 2.91 (d, *J* = 18.2 Hz, 1H), 1.21 (t,

J = 7.0 Hz, 3H), 0.84 (s, 9H), 0.12 (s, 3H), -0.12 (s, 3H); ^{13}C NMR (175 MHz, $CDCl_3$): δ 198.9, 171.3, 163.8, 143.5, 134.2, 132.0, 131.4, 131.0, 130.0, 126.3, 125.5, 121.7, 82.3, 61.8, 54.6, 25.7, 18.4, 13.9, -2.5, -3.1; HRMS (ESI-TOF)$^+$ calcd for $C_{26}H_{30}Br_2NaO_4Si$ [M+Na]$^+$: 615.0178, found: 615.0184.

Spectral data for ethyl 3-((tert-butyldimethylsilyl)oxy)-2,3-bis(4-chlorophenyl)-5-oxocyclopent-1-ene-1-carboxylate (4e):

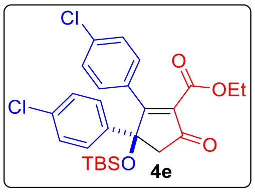

Purified by silica gel column, hexane/ethyl acetate (95:5) as eluent; Colorless liquid (77.0 mg, 38%); ^{1}H NMR (700 MHz, $CDCl_3$): δ 7.28 (s, 4H), 7.21 (d, J = 8.4 Hz, 2H), 7.18 (d, J = 8.4 Hz, 2H), 4.30 – 4.25 (m, 2H), 3.14 (d, J = 18.2 Hz, 1H), 2.91 (d, J = 18.2 Hz, 1H), 1.21 (t, J = 7.0 Hz, 3H), 0.84 (s, 9H), 0.12 (s, 3H), -0.12 (s, 3H); ^{13}C NMR (175 MHz, $CDCl_3$): δ 198.9, 171.3, 163.9, 143.0, 137.0, 134.2, 133.5, 130.9, 129.6, 129.0, 128.5, 125.9, 82.3, 61.8, 54.6, 25.7, 18.4, 13.9, -2.5, -3.2; HRMS (ESI-TOF)$^+$ calcd for $C_{26}H_{30}Cl_2NaO_4Si$ [M+Na]$^+$: 527.1188, found: 527.1189.

Spectral data for ethyl 3-((tert-butyldimethylsilyl)oxy)-5-oxo-2,3-di-m-tolylcyclopent-1-ene-1-carboxylate (4f):

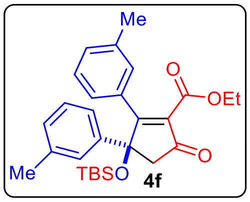

Purified by silica gel column, hexane/ethyl acetate (95:5) as eluent; Colorless liquid (110.0 mg, 59%); ^{1}H NMR (700 MHz, $CDCl_3$): δ 7.20 – 7.18 (m, 2H), 7.16 (s, 1H), 7.10 – 7.06 (m, 3H), 7.04 (d, J = 4.9 Hz, 1H), 6.99 (d, J = 7.0 Hz, 1H), 4.29 – 4.24 (m, 2H), 3.12 (d, J = 18.2 Hz, 1H), 2.95 (d, J = 18.2 Hz, 1H), 2.29 (s, 3H), 2.19 (s, 3H), 1.19 (t, J = 7.0 Hz, 3H), 0.83 (s, 9H), 0.11 (s, 3H), -0.17 (s, 3H); ^{13}C NMR (175 MHz, $CDCl_3$): δ 199.8, 173.9, 164.4, 144.7, 138.3, 137.4, 133.5, 131.4, 131.3, 130.3, 128.5, 128.1, 127.8, 126.9, 125.1, 121.6, 82.5, 61.4, 55.1, 25.7, 21.6, 21.2, 18.4, 13.8, -2.5, -3.3; HRMS (ESI-TOF)$^+$ calcd for $C_{28}H_{36}NaO_4Si$ [M+Na]$^+$: 487.2280, found: 487.2283.

Spectral data for ethyl 3-((tert-butyldimethylsilyl)oxy)-2,3-bis(3-methoxyphenyl)-5-oxocyclopent-1-ene-1-carboxylate (4g):

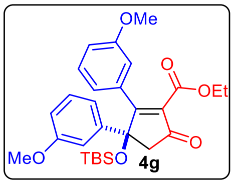

Purified by silica gel column, hexane/ethyl acetate (94:6) as eluent; Colorless liquid (120.5 mg, 60%); ^{1}H NMR (700 MHz, $CDCl_3$): δ 7.26 (d, J = 6.3 Hz, 1H), 7.14 (t, J = 8.4 Hz, 1H), 7.08 (s, 1H), 6.95 (d, J = 7.7 Hz, 1H), 6.89 – 6.85 (m, 2H), 6.80 (m, 1H), 6.76 (s, 1H), 4.31 – 4.27 (m, 2H), 3.76 (s, 3H), 3.59 (s, 3H), 3.14 (d, J = 18.2 Hz, 1H), 2.97 (d, J = 18.2 Hz, 1H), 1.12 (t, J = 7.0 Hz, 3H), 0.85 (s, 9H), 0.15 (s, 3H), -

0.13 (s, 3H); ^{13}C NMR (175 MHz, $CDCl_3$): δ 199.6, 172.8, 164.3, 159.9, 159.0, 146.7, 134.2, 132.6, 129.9, 129.0, 122.4, 117.1, 116.6, 114.3, 112.9, 110.2, 82.5, 61.6, 55.1, 55.08, 55.02, 25.7, 18.4, 13.9, -2.6, -3.3; HRMS (ESI-TOF)$^+$ calcd for $C_{28}H_{36}NaO_6Si$ $[M+Na]^+$: 519.2179, found: 519.2179.

Spectral data for ethyl 3-((tert-butyldimethylsilyl)oxy)-2,3-bis(3-methoxyphenyl)-5-oxocyclopent-1-ene-1-carboxylate (4h):

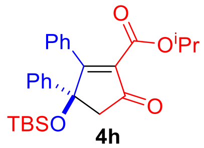

4h

Purified by silica gel column, hexane/ethyl acetate (95:5) as eluent; Colorless liquid (112.0 mg, 62%); ^{1}H NMR (700 MHz, $CDCl_3$): δ 7.40 (d, J = 7.7 Hz, 2H), 7.31 (t, J = 7.0 Hz, 2H), 7.28 – 7.26 (m, 1H), 7.25 – 7.23 (m, 1H), 7.21 – 7.17 (m, 4H), 5.17 – 5.14 (m, 1H), 3.14 (d, J = 18.2 Hz, 1H), 2.95 (d, J = 18.2 Hz, 1H), 1.19 (d, J = 5.6 Hz, 3H), 1.14 (d, J = 6.3 Hz, 3H), 0.82 (s, 9H), 0.12 (s, 3H), -0.16 (s, 3H); ^{13}C NMR (175 MHz, $CDCl_3$): δ 199.7, 173.0, 163.8, 144.7, 134.2, 131.6, 130.4, 129.7, 128.7, 127.9, 127.3, 124.5, 82.5, 69.3, 55.1, 25.7, 21.5, 21.3, 18.4, -2.6, -3.3; HRMS (ESI-TOF)$^+$ calcd for $C_{27}H_{34}NaO_4Si$ $[M+Na]^+$: 473.2124, found: 473.2124.

Spectral data for benzyl 3-((tert-butyldimethylsilyl)oxy)-5-oxo-2,3-diphenylcyclopent-1-ene-1-carboxylate (4i):

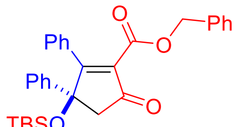

4i

Purified by silica gel column, hexane/ethyl acetate (95:5) as eluent; Colorless liquid (118.0 mg, 59%); ^{1}H NMR (700 MHz, $CDCl_3$): δ 7.39 (d, J = 7.7 Hz, 2H), 7.32 – 7.23 (m, 7H), 7.19 – 7.18 (m, 2H), 7.09 – 7.06 (m, 4H), 5.27 – 5.19 (m, 2H), 3.16 (d, J = 18.2 Hz, 1H), 2.97 (d, J = 18.2 Hz, 1H), 0.80 (s, 9H), 0.11 (s, 3H), -0.19 (s, 3H); ^{13}C NMR (175 MHz, $CDCl_3$): δ 199.4, 174.3, 164.0, 144.6, 134.9, 133.5, 131.3, 130.5, 129.6, 128.8, 128.49, 128.45, 128.2, 128.0, 127.4, 124.5, 82.5, 67.1, 55.1, 25.7, 18.4, -2.6, -3.3; HRMS (ESI-TOF)$^+$ calcd for $C_{31}H_{34}NaO_4Si$ $[M+Na]^+$: 521.2124, found: 521.2124.

Spectral data for butyl 3-((tert-butyldimethylsilyl)oxy)-5-oxo-2,3-diphenylcyclopent-1-ene-1-carboxylate (4j):

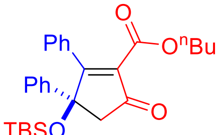

4j

Purified by silica gel column, hexane/ethyl acetate (95:5) as eluent; Colorless liquid (113.5 mg, 61%); ^{1}H NMR (700 MHz, $CDCl_3$): δ 7.40 (d, J = 7.7 Hz, 2H), 7.32 (t, J = 7.7 Hz, 2H), 7.28 (d, J = 1.4 Hz, 1H), 7.25 – 7.24 (m, 1H), 7.19 – 7.17 (m, 4H), 4.21 – 4.18 (m, 2H), 3.15 (d, J = 18.2 Hz, 1H), 2.95 (d, J = 18.2 Hz, 1H), 1.53 – 1.49 (m, 2H), 1.20 – 1.15 (m, 2H), 0.82 – 0.79 (s, 12H), 0.12 (s, 3H), -0.17 (s, 3H); ^{13}C NMR (175 MHz, $CDCl_3$): δ 199.6, 173.6, 164.3, 144.7, 133.9, 131.6, 130.5, 129.6, 128.7, 128.0, 127.4, 124.5,

82.6, 65.3, 55.1, 30.2, 25.7, 18.8, 18.4, 13.5, -2.6, -3.3; HRMS (ESI-TOF)$^+$ calcd for $C_{28}H_{36}NaO_4Si$ $[M+Na]^+$: 487.2280, found: 487.2287.

Spectral data for butyl 3-((tert-butyldimethylsilyl)oxy)-5-oxo-2,3-diphenylcyclopent-1-ene-1-carboxylate (4k):

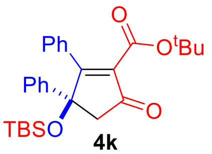

4k

Purified by silica gel column, hexane/ethyl acetate (95:5) as eluent; Colorless liquid (101.0 mg, 54%); ^1H NMR (700 MHz, $CDCl_3$): δ 7.38 (d, J = 7.7 Hz, 2H), 7.32 – 7.26 (m, 3H), 7.24 – 7.23 (m, 3H), 7.20 – 7.18 (m, 2H), 3.12 (d, J = 18.2 Hz, 1H), 2.93 (d, J = 18.2 Hz, 1H), 1.41 (s, 9H), 0.83 (s, 9H), 0.11 (s, 3H), -0.16 (s, 3H); ^{13}C NMR (175 MHz, $CDCl_3$): δ 199.9, 172.0, 163.4, 144.8, 135.0, 131.8, 130.0, 129.7, 128.6, 127.8, 127.3, 124.5, 82.8, 82.5, 55.1, 27.8, 25.7, 18.4, -2.6, -3.3; HRMS (ESI-TOF)$^+$ calcd for $C_{28}H_{36}NaO_4Si$ $[M+Na]^+$: 487.2280, found: 487.2281.

Spectral data for cyclohexyl 3-((tert-butyldimethylsilyl)oxy)-5-oxo-2,3-diphenylcyclopent-1-ene-1-carboxylate (4l):

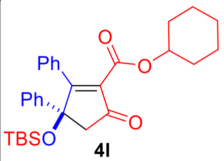

4l

Purified by silica gel column, hexane/ethyl acetate (95:5) as eluent; Colorless liquid (112.7 mg, 57%); ^1H NMR (700 MHz, $CDCl_3$): δ 7.39 (d, J = 7.7 Hz, 2H), 7.32 – 7.29 (m, 2H), 7.27 (t, J = 7.7 Hz, 1H), 7.24 – 7.22 (m, 3H), 7.17 (t, J = 7.0 Hz, 2H), 4.97 – 4.94 (m, 1H), 3.13 (d, J = 18.2 Hz, 1H), 2.94 (d, J = 18.2 Hz, 1H), 1.79 – 1.74 (m, 2H), 1.53 – 1.16 (m, 7H), 0.83 (s, 9H), 0.12 (s, 3H), -0.16 (s, 3H); ^{13}C NMR (175 MHz, $CDCl_3$): δ 199.7, 172.8, 163.7, 144.8, 134.4 131.6, 130.4, 129.7, 128.7, 127.9, 127.3, 124.5, 82.6, 73.9, 55.1, 31.1, 31.0, 25.7, 25.2, 23.3, 23.2, 18.4, -2.6, -3.3; HRMS (ESI-TOF)$^+$ calcd for $C_{30}H_{38}NaO_4Si$ $[M+Na]^+$: 513.2437, found: 513.2445.

Spectral data for allyl 3-((tert-butyldimethylsilyl)oxy)-5-oxo-2,3-diphenylcyclopent-1-ene-1-carboxylate (4m):

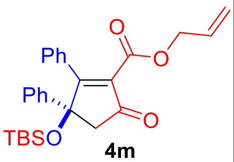

4m

Purified by silica gel column, hexane/ethyl acetate (95:5) as eluent; Colorless liquid (86.5 mg, 48%); ^1H NMR (700 MHz, $CDCl_3$): δ 7.41 (d, J = 7.7 Hz, 2H), 7.32 (t, J = 7.7 Hz, 2H), 7.30 – 7.27 (m, 1H), 7.26 (d, J = 7.0 Hz, 1H), 7.20 – 7.18 (m, 4H), 5.84 – 5.78 (m, 1H), 5.24 (d, J = 16.8 Hz, 1H), 5.18 (d, J = 16.8 Hz, 1H), 4.73 – 4.68 (m, 2H), 3.16 (d, J = 18.2 Hz, 1H), 2.96 (d, J = 18.2 Hz, 1H), 0.81 (s, 9H), 0.12 (s, 3H), -0.17 (s, 3H); ^{13}C NMR (175 MHz, $CDCl_3$): δ 199.4, 174.1, 163.9, 144.7, 133.5, 131.4, 131.1, 130.6, 129.7,

128.8, 128.0, 127.4, 124.4, 118.9, 82.6, 66.0, 55.1, 25.7, 18.4, -2.5, -3.3; HRMS (ESI-TOF)$^+$ calcd for $C_{27}H_{32}NaO_4Si$ [M+Na]$^+$: 471.1967, found: 471.1965.

Spectral data for ethyl 3-((tert-butyldimethylsilyl)oxy)-3-(4-methoxyphenyl)-5-oxo-2-phenylcyclopent-1-ene-1-carboxylate (5a):

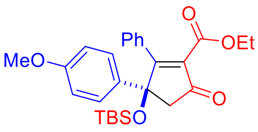

5a (Major)

Purified by silica gel column, hexane/ethyl acetate (95:5) as eluent; Colorless liquid (71.0 mg, 38%); ^{1}H NMR (700 MHz, $CDCl_3$): δ 7.30 (d, *J* = 7.7 Hz, 3H), 7.20 – 7.19 (m, 4H), 6.85 (d, *J* = 8.4 Hz, 2H), 4.26 – 4.21 (m, 2H), 3.77 (s, 3H), 3.15 (d, *J* = 18.2 Hz, 1H), 2.95 (d, *J* = 18.2 Hz, 1H), 1.16 (t, *J* = 7.0 Hz, 3H), 0.80 (s, 9H), 0.10 (s, 3H), -0.17 (s, 3H); ^{13}C NMR (175 MHz, $CDCl_3$): δ 199.6, 174.0, 164.1, 158.8, 136.5, 133.5, 131.6, 130.4, 129.6, 127.9, 125.7, 114.0, 82.2, 61.4, 55.2, 55.1, 25.7, 18.4, 13.8, -2.63, -2.64, -3.3; HRMS (ESI-TOF)$^+$ calcd for $C_{27}H_{34}NaO_5Si$ [M+Na]$^+$: 489.2073, found: 489.2076.

Spectral data for ethyl 3-((tert-butyldimethylsilyl)oxy)-2-(4-methoxyphenyl)-5-oxo-3-phenylcyclopent-1-ene-1-carboxylate (5a'):

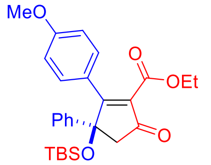

5a' (Minor)

Purified by silica gel column, hexane/ethyl acetate (95:5) as eluent; Colorless liquid (43.0 mg, 23%); ^{1}H NMR (700 MHz, $CDCl_3$): δ 7.42 (d, *J* = 7.7 Hz, 2H), 7.32 – 7.29 (m, 4H), 7.23 (d, *J* = 7.0 Hz, 1H), 6.72 (d, *J* = 8.4 Hz, 2H) 4.34 – 4.30 (m, 2H), 3.74 (s, 3H), 3.10 (d, *J* = 18.9 Hz, 1H), 2.87 (d, *J* = 18.9 Hz, 1H), 1.26 (t, *J* = 7.0 Hz, 3H), 0.85 (s, 9H), 0.14 (s, 3H), -0.20 (s, 3H); ^{13}C NMR (175 MHz, $CDCl_3$): δ 199.5, 171.8, 165.0, 161.6, 145.6, 132.5, 132.2, 128.7, 127.2, 124.2, 123.6, 113.6, 82.7, 61.6, 55.3, 55.2, 25.8, 18.4, 14.0, -2.6, -3.4; HRMS (ESI-TOF)$^+$ calcd for $C_{27}H_{35}O_5Si$ [M+H]$^+$: 467.2254, found: 467.2249.

Spectral data for ethyl 3-((tert-butyldimethylsilyl)oxy)-2-(4-chlorophenyl)-3-(4-methoxyphenyl)-5-oxocyclopent-1-ene-1-carboxylate (5b):

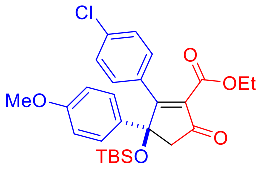

5b (Major)

Purified by silica gel column, hexane/ethyl acetate (95:5) as eluent; Colorless liquid (80.0 mg, 40%); ^{1}H NMR (700 MHz, $CDCl_3$): δ 7.25 (d, *J* = 8.4 Hz, 2H), 7.20 (d, *J* = 8.4 Hz, 2H), 7.16 (d, *J* = 8.4 Hz, 2H), 6.84 (d, *J* = 8.4 Hz, 2H), 4.27 – 4.23 (m, 2H), 3.77 (s, 3H), 3.14 (d, *J* = 18.2 Hz, 1H), 2.96 (d, *J* = 18.2 Hz, 1H), 1.19 (t, *J* = 7.0 Hz, 3H), 0.83 (s, 9H), 0.10 (s, 3H), -0.13 (s, 3H); ^{13}C NMR (175 MHz, $CDCl_3$): δ 199.4,

172.5, 163.9, 158.9, 136.6, 136.1, 133.5, 130.9, 130.0, 128.2, 125.8, 114.1, 82.2, 61.6, 55.2, 54.8, 25.7, 18.4, 13.9, -2.5, -3.1; HRMS (ESI-TOF)$^+$ calcd for $C_{27}H_{33}ClNaO_5Si$ [M+Na]$^+$: 523.1683, found: 523.1682.

Spectral data for ethyl 3-((tert-butyldimethylsilyl)oxy)-3-(4-chlorophenyl)-2-(4-methoxyphenyl)-5-oxocyclopent-1-ene-1-carboxylate (5b'):

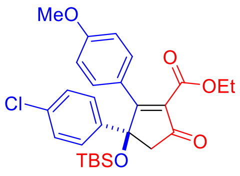

5b' (Minor)

Purified by silica gel column, hexane/ethyl acetate (95:5) as eluent; Colorless liquid (36.5 mg, 18%); ^{1}H NMR (700 MHz, $CDCl_3$): δ 7.34 (d, J = 8.4 Hz, 2H), 7.29 – 7.27 (m, 4H), 6.73 (d, J = 9.1 Hz, 2H), 4.35 – 4.30 (m, 2H), 3.75 (s, 3H), 3.09 (d, J = 18.2 Hz, 1H), 2.82 (d, J = 18.2 Hz, 1H), 1.26 (t, J = 7.0 Hz, 3H), 0.85 (s, 9H), 0.13 (s, 3H), -0.20 (s, 3H); ^{13}C NMR (175 MHz, $CDCl_3$): δ 199.1, 171.1, 164.8, 161.8, 144.3, 133.1, 132.7, 132.2, 128.9, 125.7, 123.3, 113.7, 82.4, 61.7, 55.3, 55.1, 25.8, 18.4, 14.0, -2.6, -3.4; HRMS (ESI-TOF)$^+$ calcd for $C_{27}H_{33}ClNaO_5Si$ [M+Na]$^+$: 523.1683, found: 523.1683.

Spectral data for ethyl 3-((tert-butyldimethylsilyl)oxy)-2-(4-fluorophenyl)-3-(4-methoxyphenyl)-5-oxocyclopent-1-ene-1-carboxylate (5c):

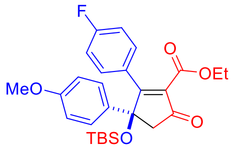

5c (Major)

Purified by silica gel column, hexane/ethyl acetate (94:6) as eluent; Colorless liquid (84.0 mg, 43%); ^{1}H NMR (700 MHz, $CDCl_3$): δ 7.26 – 7.22 (m, 4H), 6.90 (t, J = 8.4 Hz, 2H), 6.84 (d, J = 8.4 Hz, 2H), 4.28 – 4.23 (m, 2H), 3.77 (s, 3H), 3.13 (d, J = 18.2 Hz, 1H), 2.94 (d, J = 18.2 Hz, 1H), 1.19 (t, J = 7.0 Hz, 3H), 0.81 (s, 9H), 0.10 (s, 3H), -0.16 (s, 3H); ^{13}C NMR (175 MHz, $CDCl_3$): δ 199.4, 172.5, 164.5 (d, J = 250.2 Hz), 164.1, 158.9, 136.3, 133.4, 131.9 (d, J = 8.75 Hz), 127.7 (d, J = 3.5 Hz), 115.3 (d, J = 22.7 Hz), 114.1, 82.2, 61.6, 55.2, 54.9, 25.7, 18.4, 13.9, -2.5, -3.2; ^{19}F NMR (471 MHz, $CDCl_3$): δ -108.6; HRMS (ESI-TOF)$^+$ calcd for $C_{27}H_{33}FNaO_5Si$ [M+Na]$^+$: 507.1979, found: 507.1978.

Spectral data for ethyl 3-((tert-butyldimethylsilyl)oxy)-3-(4-fluorophenyl)-2-(4-methoxyphenyl)-5-oxocyclopent-1-ene-1-carboxylate (5c'):

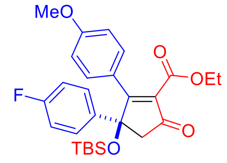

5c' (Minor)

Purified by silica gel column, hexane/ethyl acetate (94:6) as eluent; Colorless liquid (23.5 mg, 12%); ^{1}H NMR (700 MHz, $CDCl_3$): δ 7.38 (m, 2H), 7.29 (d, J = 8.4 Hz, 2H), 6.99 (t, J = 8.4 Hz, 2H), 6.73 (d, J = 8.4 Hz, 2H), 4.34 – 4.30 (m, 2H), 3.75 (s, 3H), 3.10 (d, J = 18.2 Hz, 1H), 2.83 (d, J = 18.2 Hz, 1H), 1.26 (t, J = 7.0 Hz, 3H), 0.85 (s, 9H), 0.13 (s, 3H), -0.20 (s, 3H); ^{13}C NMR (175 MHz, $CDCl_3$): δ 199.3, 171.4,

164.9, 162.6 (d, J = 245 Hz), 161.7, 141.4 (d, J = 3.5 Hz), 132.6, 132.2, 126.0 (d, J = 7.0 Hz), 123.4, 115.6 (d, J = 21 Hz), 113.6, 82.4, 61.6, 55.3, 55.2, 25.8, 18.4, 14.0, -2.6, -3.4; ^{19}F NMR (471 MHz, $CDCl_3$): δ -115.3; HRMS (ESI-TOF)$^+$ calcd for $C_{27}H_{33}FNaO_5Si$ [M+Na]$^+$: 507.1979, found: 509.1977.

Spectral data for ethyl 3-((tert-butyldimethylsilyl)oxy)-3-(4-methoxyphenyl)-5-oxo-2-(4-(trifluoromethyl)phenyl)cyclopent-1-ene-1-carboxylate (5d):

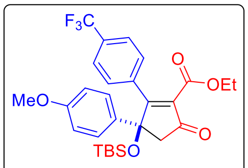

5d

Purified by silica gel column, hexane/ethyl acetate (93:7) as eluent; Colorless liquid (103.0 mg, 48%); ^{1}H NMR (700 MHz, $CDCl_3$): δ 7.47 (d, J = 8.4 Hz, 2H), 7.24 – 7.20 (m, 4H), 6.83 (d, J = 9.1 Hz, 2H), 4.22 – 4.17 (m, 2H), 3.77 (s, 3H), 3.18 (d, J = 18.2 Hz, 1H), 3.03 (d, J = 18.2 Hz, 1H), 1.12 (t, J = 7.0 Hz, 3H), 0.80 (s, 9H), 0.07 (s, 3H), -0.09 (s, 3H); ^{13}C NMR (175 MHz, $CDCl_3$): δ 199.3, 173.4, 163.3, 159.1, 135.4, 135.3, 133.9, 131.7 (q, J = 31.5 Hz), 129.5, 126.0, 124.7 (q, J = 3.5 Hz), 124.4 (d, J = 271.2 Hz), 114.1, 82.1, 61.6, 55.2, 54.4, 25.7, 18.4, 13.8, -2.54, -2.96; ^{19}F NMR (471 MHz, $CDCl_3$): δ -63.0; HRMS (ESI-TOF)$^+$ calcd for $C_{28}H_{33}F_3NaO_5Si$ [M+Na]$^+$: 557.1947, found: 557.1946.

Spectral data for ethyl 3-((tert-butyldimethylsilyl)oxy)-5-oxo-2-phenyl-3-(p-tolyl)cyclopent-1-ene-1-carboxylate (5e):

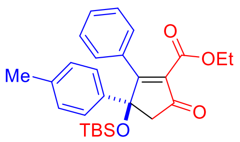

5e (Major)

Purified by silica gel column, hexane/ethyl acetate (95:5) as eluent; Colorless liquid (67.0 mg, 37%); ^{1}H NMR (700 MHz, $CDCl_3$): δ 7.28 (d, J = 7.7 Hz, 3H), 7.20 – 7.18 (m, 4H), 7.13 (d, J = 7.7 Hz, 2H), 4.27 – 4.22 (m, 2H), 3.14 (d, J = 18.2 Hz, 1H), 2.94 (d, J = 18.2 Hz, 1H), 2.31 (s, 3H), 1.17 (t, J = 7.0 Hz, 3H), 0.80 (s, 9H), 0.11 (s, 3H), -0.18 (s, 3H); ^{13}C NMR (175 MHz, $CDCl_3$): δ 199.6, 173.9, 164.2, 141.7, 137.1, 133.6, 131.6, 130.5, 129.6, 129.4, 127.9, 124.4, 82.4, 61.5, 55.2, 25.7, 20.9, 18.4, 13.8, -2.6, -3.3; HRMS (ESI-TOF)$^+$ calcd for $C_{27}H_{34}NaO_4Si$ [M+Na]$^+$: 473.2124, found: 473.2125.

Spectral data for ethyl 3-((tert-butyldimethylsilyl)oxy)-5-oxo-3-phenyl-2-(p-tolyl)cyclopent-1-ene-1-carboxylate (5e'):

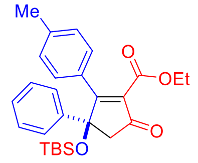

5e' (Minor)

Purified by silica gel column, hexane/ethyl acetate (95:5) as eluent; Colorless liquid (40.2 mg, 22%); ^{1}H NMR (700 MHz, $CDCl_3$): δ 7.41 (d, J = 7.7 Hz, 2H), 7.31 (t, J = 7.0 Hz, 2H), 7.23 (d, J = 7.0 Hz, 1H), 7.17 (d, J = 7.7 Hz, 2H), 7.00 (d, J = 7.7 Hz, 2H), 4.31 – 4.28 (m, 2H), 3.12 (d, J = 18.2 Hz, 1H), 2.91 (d, J = 18.2 Hz, 1H), 2.26 (s, 3H), 1.23 (t, J = 7.0 Hz, 3H), 0.84 (s, 9H), 0.13 (s, 3H), -0.19 (s, 3H); ^{13}C NMR (175

MHz, $CDCl_3$): δ 199.6, 173.9, 164.2, 141.7, 137.1, 133.6, 131.6, 130.5, 129.6, 129.4, 127.9, 124.4, 82.4, 61.5, 55.2, 25.7, 20.9, 18.4, 13.8, -2.6, -3.3; HRMS (ESI-TOF)$^+$ calcd for $C_{27}H_{34}NaO_4Si$ [M+Na]$^+$: 473.2124, found: 473.2126.

Spectral data for ethyl 3-((tert-butyldimethylsilyl)oxy)-5-oxo-2-phenyl-3-(p-tolyl)cyclopent-1-ene-1-carboxylate (5f/5f'):

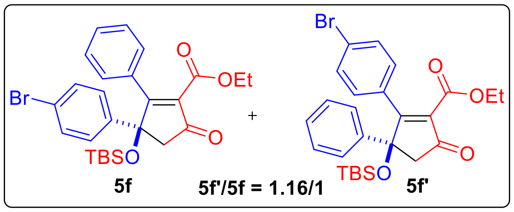

Purified by silica gel column, hexane/ethyl acetate (95:5) as eluent; Colorless liquid (105.0 mg, 51%); ^{1}H NMR (700 MHz, $CDCl_3$) (**5f**, Minor isomer): δ 7.34 – 7.30 (m, 4H, merged with major isomer), 7.24 – 7.21 (m, 5H, merged with major isomer), 4.28 – 4.26 (m, 2H, merged with major isomer), 3.14 (d, *J* = 18.2 Hz, 1H, merged with major isomer), 2.90 (d, *J* = 18.2 Hz, 1H), 1.22 – 1.17 (m, 3H, merged with major isomer), 0.83 (s, 9H), 0.12 (s, 3H, merged with major isomer), -0.17 (s, 3H); ^{13}C NMR (175 MHz, $CDCl_3$): δ 199.1, 172.1, 164.0, 144.0, 134.0, 131.9, 131.2, 130.8, 130.4, 127.6, 125.2, 121.5, 82.3, 61.7, 54.8, 25.7, 18.4, 13.8, -2.6, -3.3;

^{1}H NMR (700 MHz, $CDCl_3$) (**5f'**, Major isomer): δ 7.45 (d, *J* = 8.4 Hz, 2H), 7.34 – 7.30 (m, 3H, merged with minor isomer), 7.27 (d, *J* = 8.4 Hz, 2H), 7.10 (d, *J* = 8.4 Hz, 2H), 4.28 – 4.26 (m, 2H, merged with minor isomer), 3.15 (d, *J* = 18.2 Hz, 1H, merged with minor isomer), 2.97 (d, *J* = 18.2 Hz, 1H), 1.22 – 1.17 (m, 3H, merged with minor isomer), 0.84 (s, 9H), 0.12 (s, 3H, merged with minor isomer), -0.12 (s, 3H); ^{13}C NMR (175 MHz, $CDCl_3$): δ 199.4, 172.8, 164.1, 144.2, 134.1, 131.3, 131.0, 129.6, 128.8, 128.1, 126.3, 124.4, 82.5, 61.7, 54.9, 25.7, 18.4, 13.9, -2.5, -3.1; HRMS (ESI-TOF)$^+$ calcd for $C_{26}H_{31}NaBrO_4Si$ [M+Na]$^+$: 537.1073, found: 537.1072.

Spectral data for ethyl 3-(2,2-diphenylacetyl)-4-hydroxy-1H-pyrazole-5-carboxylate (6a):

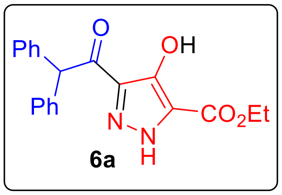

Purified by silica gel column, hexane/ethyl acetate (80:20) as eluent; White solid (95.5 mg, 68%); ^{1}H NMR (700 MHz, $CDCl_3$): δ 8.65 (s, 1H), 7.36 (d, *J* = 7.7 Hz, 4H), 7.30 (t, *J* = 7.0 Hz, 4H), 7.26 (d, *J* = 7.7 Hz, 2H), 6.16 (s, 1H), 4.44 – 4.41 (m, 2H), 1.39 (t, *J* = 7.0 Hz, 3H); ^{13}C NMR (175 MHz, $CDCl_3$): δ 137.8, 129.1, 128.6, 127.3, 61.7, 14.2 (Fewer signals than expected (C=O and CO_2Et carbonyl carbons, CH, and three pyrazole carbons are missing) due to dilute sample and slow relaxation of these signals); HRMS (ESI-TOF)$^+$ calcd for $C_{20}H_{18}N_2NaO_4$ [M+Na]$^+$: 373.1164, found: 373.1164.

Spectral data for ethyl 4-hydroxy-3-(2-phenyl-2-(p-tolyl)acetyl)-1H-pyrazole-5-carboxylate (6b):

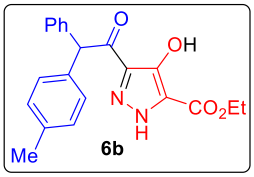

Purified by silica gel column, hexane/ethyl acetate (80:20) as eluent; White solid (102.0 mg, 70%); ^{1}H NMR (700 MHz, $CDCl_3$): δ 11.8 (s, 1H), 8.68 (s, 1H), 7.36 (d, J = 7.7 Hz, 2H), 7.30 (t, J = 7.7 Hz, 2H), 7.25 (d, J = 8.4 Hz, 3H), 7.12 (d, J = 7.7 Hz, 2H), 6.14 (s, 1H), 4.45 – 4.42 (m, 2H), 2.31 (s, 3H), 1.40 (t, J = 7.0 Hz, 3H); ^{13}C NMR (175 MHz, $CDCl_3$): δ 147.8, 138.0, 137.0, 134.8, 129.3, 129.0, 128.9, 128.5, 127.2, 61.7, 58.3, 21.0, 14.2 (Fewer signals than expected (C=O and three pyrazole carbons are missing) due to dilute sample and slow relaxation of these signals); HRMS (ESI-TOF)$^+$ calcd for $C_{21}H_{20}N_2NaO_4$ $[M+Na]^+$: 387.1321, found: 387.1319.

Spectral data for ethyl 3-(2-(4-chlorophenyl)-2-phenylacetyl)-4-hydroxy-1H-pyrazole-5-carboxylate (6c):

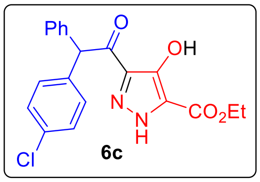

Purified by silica gel column, hexane/ethyl acetate (80:20) as eluent; White solid (100.3 mg, 65%); ^{1}H NMR (700 MHz, $CDCl_3$): δ 11.2 (s, 1H), 8.55 (s, 1H), 7.31 – 7.29 (m, 4H), 7.26 – 7.25 (m, 5H), 6.07 (s, 1H), 4.43 – 4.40 (m, 2H), 1.38 (t, J = 7.7 Hz, 3H); ^{13}C NMR (175 MHz, $CDCl_3$): δ 147.7, 137.4, 136.3, 133.3, 130.5, 129.0, 128.78, 128.77, 127.5, 61.8, 58.0, 14.2 (Fewer signals than expected (C=O and three pyrazole carbons are missing) due to dilute sample and slow relaxation of these signals); HRMS (ESI-TOF)$^+$ calcd for $C_{20}H_{17}ClN_2NaO_4$ $[M+Na]^+$: 407.0774, found: 407.0770.

Spectral data for ethyl 3-(2-(4-bromophenyl)-2-phenylacetyl)-4-hydroxy-1H-pyrazole-5-carboxylate (6d):

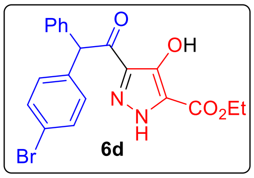

Purified by silica gel column, hexane/ethyl acetate (80:20) as eluent; White solid (106.6 mg, 62%); ^{1}H NMR (700 MHz, $CDCl_3$): δ 11.7 (s, 1H), 8.60 (s, 1H), 7.41 (d, J = 8.4 Hz, 2H), 7.32 – 7.28 (m, 4H), 7.26 – 7.24 (m, 1H), 7.22 (d, J = 8.4 Hz, 2H), 6.09 (s, 1H), 4.44 – 4.41 (m, 2H), 1.38 (t, J = 7.0 Hz, 3H); ^{13}C NMR (175 MHz, $CDCl_3$): δ 147.8, 137.3, 136.9, 131.7, 130.8, 129.0, 128.7, 127.5, 121.5, 61.8, 58.0, 14.2 (Fewer signals than expected (C=O and three pyrazole carbons are missing) due to dilute sample and slow relaxation of these signals); HRMS (ESI-TOF)$^+$ calcd for $C_{20}H_{17}BrN_2NaO_4$ $[M+Na]^+$: 451.0269, found: 451.0273.

Spectral data for ethyl 4-hydroxy-3-(2-phenyl-2-(4-(trifluoromethyl)phenyl)acetyl)-1H-pyrazole-5-carboxylate (6e):

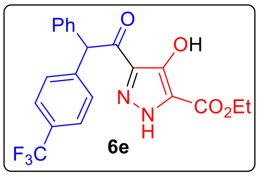

Purified by silica gel column, hexane/ethyl acetate (75:25) as eluent; White solid (85.9 mg, 51%); ^{1}H NMR (700 MHz, $CDCl_3$): δ 11.5 (s, 1H), 8.56 (s, 1H), 7.55 (d, J = 8.4 Hz, 2H), 7.46 (d, J = 8.4 Hz, 2H), 7.34 – 7.30 (m, 4H), 7.27 – 7.25 (m, 1H), 6.18 (s, 1H), 4.44 – 4.41 (m, 2H), 1.38 (t, J = 7.0 Hz, 3H); ^{13}C NMR (175 MHz, $CDCl_3$): δ 147.8, 141.9, 136.9, 129.6 (q, J = 33.25 Hz), 129.5, 129.0, 128.8, 127.7, 125.5 (q, J = 3.5 Hz), 124.0 (q, J = 271.25 Hz), 61.8, 58.4, 14.2 (Fewer signals than expected (C=O and three pyrazole carbons are missing) due to dilute sample and slow relaxation of these signals); ^{19}F NMR (471 MHz, $CDCl_3$): δ -62.6; HRMS (ESI-TOF)$^+$ calcd for $C_{21}H_{17}N_2F_3NaO_4$ $[M+H]^+$: 441.1038, found: 441.1038.

Spectral data for ethyl 3-(2-(3-bromophenyl)-2-phenylacetyl)-4-hydroxy-1H-pyrazole-5-carboxylate (6f):

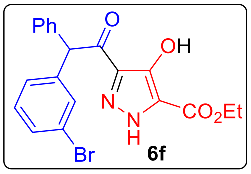

Purified by silica gel column, hexane/ethyl acetate (80:20) as eluent; White solid (100.0 mg, 58%); ^{1}H NMR (700 MHz, $CDCl_3$): δ 11.17 (s, 1H), 8.55 (s, 1H), 7.49 (s, 1H), 7.38 (d, J = 8.4 Hz, 1H), 7.33 – 7.30 (m, 4H), 7.27 – 7.24 (m, 2H), 7.17 (t, J = 7.7 Hz, 1H), 6.06 (s, 1H), 4.42 (t, J = 7.0 Hz, 2H), 1.38 (t, J = 7.7 Hz, 3H); ^{13}C NMR (175 MHz, $CDCl_3$): δ 147.7, 140.1, 137.1, 132.1, 130.5, 130.0, 129.0, 128.8, 127.8, 127.6, 122.6, 61.8, 58.2, 14.2 (Fewer signals than expected (C=O and three pyrazole carbons are missing) due to dilute sample and slow relaxation of these signals); HRMS (ESI-TOF)$^+$ calcd for $C_{20}H_{17}BrN_2NaO_4$ $[M+Na]^+$: 451.0269, found: 451.0269.

Spectral data for ethyl 4-hydroxy-3-(2-(4-methoxyphenyl)-2-phenylacetyl)-1H-pyrazole-5-carboxylate (6g):

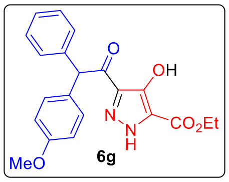

Purified by silica gel column, hexane/ethyl acetate (80:20) as eluent; White solid (96.0 mg, 63%); ^{1}H NMR (700 MHz, $CDCl_3$): δ 7.32 (d, J = 7.0 Hz, 2H), 7.29 – 7.25 (m, 4H), 7.23 – 7.21 (m, 1H), 6.83 (t, J = 9.1 Hz, 2H), 6.06 (s, 1H), 4.42 (t, J = 7.0 Hz, 2H), 3.74 (s, 3H), 1.38 (t, J = 7.0 Hz, 3H); ^{13}C NMR (175 MHz, $CDCl_3$): δ 158.8, 147.7, 138.2, 130.2, 129.8, 129.7, 129.0, 128.6, 128.5, 127.2, 126.6, 114.0, 61.7, 57.9, 55.2, 14.2 (Fewer signals than expected (C=O peak is missing) due to dilute sample and slow relaxation of these signals); HRMS (ESI-TOF)$^+$ calcd for $C_{21}H_{20}N_2NaO_5$ $[M+Na]^+$: 403.1270, found: 403.1267.

Spectral data for ethyl 4-hydroxy-3-(2-(4-methoxyphenyl)-2-(p-tolyl)acetyl)-1H-pyrazole-5-carboxylate (6h):

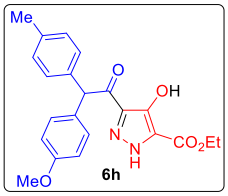

Purified by silica gel column, hexane/ethyl acetate (80:20) as eluent; White solid (97.0 mg, 61%); ^{1}H NMR (400 MHz, $CDCl_3$): δ 8.62 (s, 1H), 7.26 – 7.24 (m, 2H), 7.21 – 7.19 (m, 2H), 7.10 (d, *J* = 8.0 Hz, 2H), 6.84 – 6.81 (m, 2H), 6.02 (s, 1H), 4.42 (t, *J* = 7.2 Hz, 2H), 3.74 (s, 3H), 2.28 (s, 3H), 1.38 (t, *J* = 7.2 Hz, 3H); ^{13}C NMR (100 MHz, $CDCl_3$): δ 158.8, 147.7, 136.9, 135.2, 130.19, 130.1, 129.3, 128.9, 114.0, 61.7, 57.6, 55.2, 21.0, 14.2 (Fewer signals than expected (C=O and three pyrazole carbons are missing) due to dilute sample and slow relaxation of these signals); HRMS (ESI-TOF)$^+$ calcd for $C_{22}H_{22}N_2NaO_5$ $[M+Na]^+$: 417.1426, found: 417.1430.

Spectral data for ethyl 3-(2-(4-chlorophenyl)-2-(4-methoxyphenyl)acetyl)-4-hydroxy-1H-pyrazole-5-carboxylate (6i):

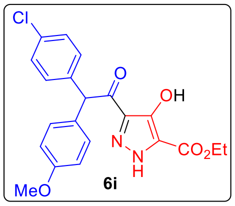

Purified by silica gel column, hexane/ethyl acetate (80:20) as eluent; White solid (95.0 mg, 57%); ^{1}H NMR (700 MHz, $CDCl_3$): δ 8.59 (s, 1H), 7.25 – 7.24 (m, 5H), 7.22 (s, 1H), 6.83 (d, *J* = 9.1 Hz, 2H) 6.03 (s, 1H), 4.42 (t, *J* = 7.0 Hz, 2H), 3.75 (s, 3H), 1.38 (t, *J* = 7.7 Hz, 3H); ^{13}C NMR (175 MHz, $CDCl_3$): δ 158.9, 147.7, 136.7, 133.2, 130.3, 130.1, 129.3, 128.7, 114.1, 61.8, 57.2, 55.2, 14.2 (Fewer signals than expected (C=O and three pyrazole carbons are missing) due to dilute sample and slow relaxation of these signals); HRMS (ESI-TOF)$^+$ calcd for $C_{21}H_{19}ClN_2NaO_5$ $[M+Na]^+$: 437.0880, found: 437.0884.

Spectral data for ethyl 3-(2-(4-bromophenyl)-2-(4-methoxyphenyl)acetyl)-4-hydroxy-1H-pyrazole-5-carboxylate (6j):

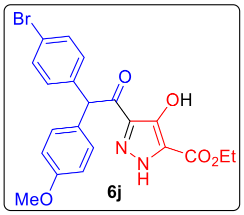

Purified by silica gel column, hexane/ethyl acetate (80:20) as eluent; White solid (97.6 mg, 53%); ^{1}H NMR (700 MHz, $CDCl_3$): δ 8.54 (s, 1H), 7.41 (d, *J* = 7.0 Hz, 2H), 7.23 (d, *J* = 9.1 Hz, 2H), 7.18 (d, *J* = 8.4 Hz, 2H), 6.84 (d, *J* = 9.1 Hz, 2H), 5.97 (s, 1H), 4.42 (t, *J* = 7.0 Hz, 2H), 3.75 (s, 3H), 1.38 (t, *J* = 7.0 Hz, 3H); ^{13}C NMR (175 MHz, $CDCl_3$): δ 159.0, 147.6, 137.3, 131.7, 131.6, 130.7, 130.2, 130.1, 129.6, 129.2, 121.4, 114.2, 61.8, 57.4, 55.2, 14.2 (Fewer signals than expected (C=O peak is missing) due to dilute sample and slow relaxation of these signals); HRMS (ESI-TOF)$^+$ calcd for $C_{21}H_{19}BrN_2NaO_5$ $[M+Na]^+$: 481.0375, found: 481.0374.

Spectral data for ethyl 3-(2-(4-fluorophenyl)-2-(4-methoxyphenyl)acetyl)-4-hydroxy-1H-pyrazole-5-carboxylate (6k):

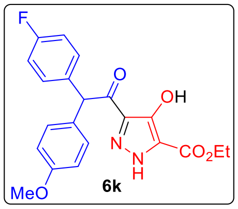

6k

Purified by silica gel column, hexane/ethyl acetate (75:25) as eluent; White solid (79.0 mg, 49%); ^{1}H NMR (700 MHz, $CDCl_3$): δ 8.56 (s, 1H), 7.28 – 7.26 (m, 2H), 7.24 – 7.22 (m, 2H), 6.97 (t, J = 9.1 Hz, 2H), 6.84 (d, J = 8.4 Hz, 2H), 6.0 (s, 1H), 4.42 (q, J = 7.0 Hz, 2H), 3.75 (s, 3H), 1.38 (t, J = 7.0 Hz, 3H); ^{13}C NMR (175 MHz, $CDCl_3$): δ 162.7 (d, J = 245 Hz), 158.9, 147.6, 133.9 (d, J = 3.5 Hz), 130.6 (d, J = 8.75 Hz), 130.1, 129.6, 115.5 (d, J = 21.0 Hz), 114.1, 61.7, 57.1, 55.2, 14.2 (Fewer signals than expected (C=O and three pyrazole carbons are missing) due to dilute sample and slow relaxation of these signals); ^{19}F NMR (471 MHz, $CDCl_3$): δ -115.3; HRMS (ESI-TOF)$^{+}$ calcd for $C_{21}H_{19}FN_2NaO_5$ [M+Na]$^{+}$: 421.1176, found: 421.1170.

Spectral data for ethyl 4-hydroxy-3-(2-(4-methoxyphenyl)-2-(4-(trifluoromethyl)phenyl)acetyl)-1H-pyrazole-5-carboxylate (6l):

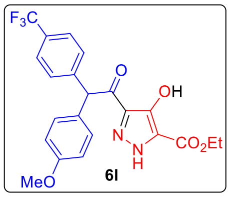

6l

Purified by silica gel column, hexane/ethyl acetate (70:30) as eluent; White solid (78.8 mg, 44%); ^{1}H NMR (700 MHz, $CDCl_3$): δ 11.19 (s, 1H), 8.54 (s, 1H), 7.55 (d, J = 8.4 Hz, 2H), 7.44 (d, J = 8.4 Hz, 2H), 7.26 – 7.24 (m, 2H), 6.85 – 6.83 (m, 2H), 6.08 (s, 1H), 4.43 (q, J = 7.2 Hz, 2H), 3.75 (s, 3H), 1.38 (t, J = 7.2 Hz, 3H); ^{13}C NMR (175 MHz, $CDCl_3$): δ 159.1, 147.7, 142.3, 131.3, 130.2, 129.5 (q, J = 32.0 Hz), 129.4, 128.8, 125.4 (q, J = 3.0 Hz), 122.7 (d, J = 270 Hz), 61.8, 57.7, 55.2, 14.2 (Fewer signals than expected (C=O and three pyrazole carbons are missing) due to dilute sample and slow relaxation of these signals); ^{19}F NMR (471 MHz, $CDCl_3$): δ -62.5; HRMS (ESI-TOF)$^{+}$ calcd for $C_{22}H_{19}F_3N_2NaO_5$ [M+Na]$^{+}$: 471.1144, found: 471.1147.

Spectral data for ethyl 4-hydroxy-3-(2-(4-methoxyphenyl)-2-(m-tolyl)acetyl)-1H-pyrazole-5-carboxylate (6m):

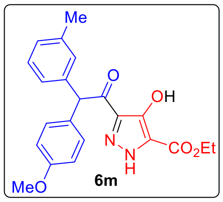

6m

Purified by silica gel column, hexane/ethyl acetate (80:20) as eluent; White solid (91.0 mg, 58%); ^{1}H NMR (400 MHz, $CDCl_3$): δ 11.06 (s, 1H), 8.61 (s, 1H), 7.26 – 7.24 (m, 2H), 7.20 – 7.16 (m, 1H), 7.11 – 7.10 (m, 2H), 7.05 (d, J = 7.6 Hz, 1H), 6.84 (d, J = 8.4 Hz, 2H), 6.00 (s, 1H), 4.42 (q, J = 6.8 Hz, 2H), 3.75 (s, 3H), 2.28 (s, 3H), 1.38 (t, J = 7.2 Hz, 3H); ^{13}C NMR (100 MHz, $CDCl_3$): δ 158.8, 147.6, 138.2, 138.0, 130.2, 129.9, 129.6, 128.4, 128.1, 126.0, 114.0, 61.7, 58.0, 55.2, 21.4, 14.2 (Fewer signals than expected (C=O

and three pyrazole carbons are missing) due to dilute sample and slow relaxation of these signals); HRMS (ESI-TOF)$^+$ calcd for $C_{22}H_{22}N_2NaO_5$ [M+Na]$^+$: 417.1426, found: 417.1432.

Spectral data for ethyl 3-(2-(3-chlorophenyl)-2-(4-methoxyphenyl)acetyl)-4-hydroxy-1H-pyrazole-5-carboxylate (6n):

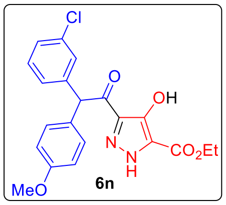

Purified by silica gel column, hexane/ethyl acetate (80:20) as eluent; White solid (90.0 mg, 54%); ^{1}H NMR (400 MHz, $CDCl_3$): δ 11.20 (s, 1H), 8.56 (s, 1H), 7.31 (s, 1H), 7.26 – 7.20 (m, 5H), 6.85 – 6.83 (m, 2H), 6.01 (s, 1H), 4.43 (q, J = 7.2 Hz, 2H), 3.75 (s, 3H), 1.38 (t, J = 7.2 Hz, 3H); ^{13}C NMR (100 MHz, $CDCl_3$): δ 159.1, 147.7, 140.3, 134.4, 130.2, 129.7, 129.1, 129.0, 127.4, 127.2, 114.2, 61.8, 57.3, 55.2, 14.2 (Fewer signals than expected (C=O and three pyrazole carbons are missing) due to dilute sample and slow relaxation of these signals); HRMS (ESI-TOF)$^+$ calcd for $C_{21}H_{19}ClN_2NaO_5$ [M+Na]$^+$: 437.0880, found: 437.0869.

Spectral data for ethyl 3-(2-(3-bromophenyl)-2-(4-methoxyphenyl)acetyl)-4-hydroxy-1H-pyrazole-5-carboxylate (6o):

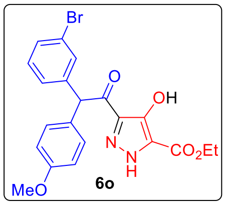

Purified by silica gel column, hexane/ethyl acetate (80:20) as eluent; White solid (94.0 mg, 51%); ^{1}H NMR (400 MHz, $CDCl_3$): δ 10.8 (s, 1H), 8.53 (s, 1H), 7.45 (s, 1H), 7.37 (d, J = 8.0 Hz, 1H), 7.25 – 7.23 (m, 3H, merged with $CDCl_3$), 7.16 (t, J = 7.6 Hz, 1H), 6.85 (d, J = 8.4 Hz, 2H), 5.97 (s, 1H), 4.42 (q, J = 7.2 Hz, 2H), 3.76 (s, 3H), 1.38 (t, J = 7.2 Hz, 3H); ^{13}C NMR (100 MHz, $CDCl_3$): δ 159.1, 147.6, 140.5, 132.0, 130.4, 130.2, 130.0, 129.0, 127.7, 122.6, 114.2, 61.8, 57.5, 55.2, 14.2 (Fewer signals than expected (C=O and three pyrazole carbons are missing) due to dilute sample and slow relaxation of these signals); HRMS (ESI-TOF)$^+$ calcd for $C_{21}H_{19}BrN_2NaO_5$ [M+Na]$^+$: 481.0375, found: 481.0382.

Spectral data for ethyl 3-(2-(2-bromophenyl)-2-(4-methoxyphenyl)acetyl)-4-hydroxy-1H-pyrazole-5-carboxylate (6p):

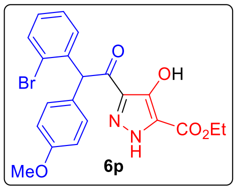

Purified by silica gel column, hexane/ethyl acetate (80:20) as eluent; White solid (86.8 mg, 47%); ^{1}H NMR (700 MHz, $CDCl_3$): δ 11.6 (s, 1H), 8.53 (s, 1H), 7.56 (d, J = 8.4 Hz, 1H), 7.22 – 7.19 (m, 3H), 7.10 (t, J = 7.7 Hz, 1H), 7.05 (d, J = 7.7 Hz, 1H), 6.85 (d, J = 8.4 Hz, 2H), 6.38 (s, 1H), 4.42 (q, J = 7.0 Hz, 2H), 3.75 (s, 3H), 1.37 (t, J = 7.0 Hz, 3H); ^{13}C NMR (175 MHz, $CDCl_3$): δ 159.1, 147.5, 138.4, 132.7, 131.0, 130.8,

128.7, 127.3, 127.2, 124.9, 114.3, 61.6, 58.4, 55.1, 14.2 (Fewer signals than expected (C=O and three pyrazole carbons are missing) due to dilute sample and slow relaxation of these signals); HRMS (ESI-TOF)$^+$ calcd for $C_{21}H_{19}BrN_2NaO_5$ $[M+Na]^+$: 481.0375, found: 481.0384.

Spectral data for ethyl 3-(2-(3,4-dichlorophenyl)-2-(4-methoxyphenyl)acetyl)-4-hydroxy-1H-pyrazole-5-carboxylate (6q):

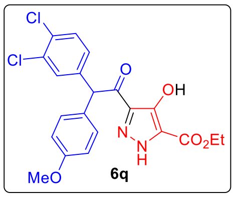

6q

Purified by silica gel column, hexane/ethyl acetate (80:20) as eluent; White solid (83.0 mg, 46%); ^{1}H NMR (700 MHz, $CDCl_3$): δ 11.2 (s, 1H), 8.54 (s, 1H), 7.41 (d, J = 1.4 Hz, 1H), 7.37 (d, J = 8.4 Hz, 1H), 7.25 – 7.24 (m, 2H), 7.17 – 7.15 (m, 1H), 6.86 (d, J = 8.4 Hz, 2H), 5.98 (s, 1H), 4.45 (q, J = 7.2 Hz, 2H), 3.77 (s, 3H), 1.40 (t, J = 7.7 Hz, 3H); ^{13}C NMR (175 MHz, $CDCl_3$): δ 159.1, 147.7, 138.5, 132.5, 131.4, 130.9, 130.4, 130.1, 128.6, 128.4, 114.3, 61.8, 56.9, 55.2, 14.2 (Fewer signals than expected (C=O and three pyrazole carbons are missing) due to dilute sample and slow relaxation of these signals); HRMS (ESI-TOF)$^+$ calcd for $C_{21}H_{18}Cl_2N_2NaO_5$ $[M+Na]^+$: 471.0490, found: 471.0495.

Spectral data for ethyl 4-hydroxy-3-(2-(4-methoxyphenyl)-2-(naphthalen-2-yl)acetyl)-1H-pyrazole-5-carboxylate (6r):

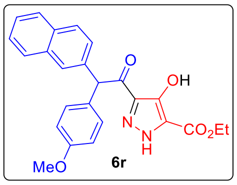

6r

Purified by silica gel column, hexane/ethyl acetate (80:20) as eluent; White solid (83.6 mg, 53%); ^{1}H NMR (700 MHz, CD_2Cl_2): δ 11.3 (s, 1H), 8.58 (s, 1H), 7.81 – 7.76 (s, 4H), 7.46 – 7.45 (s, 3H), 7.32 (d, J = 9.1 Hz, 2H), 6.87 (d, J = 9.1 Hz, 2H), 6.2 (s, 1H), 4.41 (q, J = 7.0 Hz, 2H), 3.76 (s, 3H), 1.37 (t, J = 7.0 Hz, 3H); ^{13}C NMR (175 MHz, CD_2Cl_2): δ 159.0, 147.7, 136.1, 133.4, 132.5, 130.4, 129.9, 128.2, 127.8, 127.6, 127.5, 127.3, 126.2, 126.0, 114.0, 61.7, 58.1, 55.2, 14.0 (Fewer signals than expected (C=O and three pyrazole carbons are missing) due to dilute sample and slow relaxation of these signals); HRMS (ESI-TOF)$^+$ calcd for $C_{25}H_{22}N_2NaO_5$ $[M+Na]^+$: 453.1426, found: 453.1427.

Spectral data for isopropyl 4-hydroxy-3-(2-(4-methoxyphenyl)-2-phenylacetyl)-1H-pyrazole-5-carboxylate (7b):

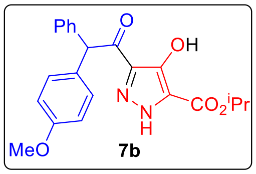

7b

Purified by silica gel column, hexane/ethyl acetate (80:20) as eluent; White solid (82.0 mg, 52%); ^{1}H NMR (700 MHz, $CDCl_3$): δ 8.62 (s, 1H), 7.33 – 7.32 (m, 2H), 7.30 – 7.26 (m, 4H), 7.24 – 7.21 (m, 1H), 6.83 (d, J = 9.1 Hz, 2H), 6.07 (s, 1H), 5.30 – 5.26 (m, 1H), 3.75 (s, 3H), 1.37 (d, J = 6.3 Hz, 6H); ^{13}C NMR (175 MHz, $CDCl_3$): δ 158.8, 147.7, 138.2, 130.2, 129.8, 129.0, 128.5, 127.2, 114.0, 69.7, 57.9, 55.2, 21.7 (Fewer

signals than expected (C=O and three pyrazole carbons are missing) due to dilute sample and slow relaxation of these signals); HRMS (ESI-TOF)$^+$ calcd for $C_{22}H_{22}N_2NaO_5$ [M+Na]$^+$: 417.1426, found: 417.1427.

Spectral data for benzyl 4-hydroxy-3-(2-(4-methoxyphenyl)-2-phenylacetyl)-1H-pyrazole-5-carboxylate (7c):

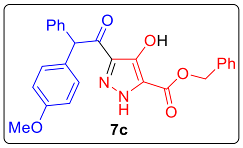

Purified by silica gel column, hexane/ethyl acetate (80:20) as eluent; White solid (85.0 mg, 48%); ^{1}H NMR (700 MHz, $CDCl_3$): δ 8.58 (s, 1H), 7.40 (d, J = 7.7 Hz, 2H), 7.34 (t, J = 7.0 Hz, 2H), 7.32 – 7.26 (m, 5H), 7.25 – 7.21 (m, 3H), 6.82 (d, J = 9.1 Hz, 2H), 6.05 (s, 1H), 5.37 (s, 2H), 3.74 (s, 3H); ^{13}C NMR (175 MHz, $CDCl_3$): δ 158.8, 148.0, 138.1, 134.9, 130.2, 129.8, 129.0, 128.66, 128.6, 128.5, 128.3, 127.2, 114.0, 67.0, 57.9, 55.2 (Fewer signals than expected (C=O and three pyrazole carbons are missing) due to dilute sample and slow relaxation of these signals); HRMS (ESI-TOF)$^+$ calcd for $C_{26}H_{22}N_2NaO_5$ [M+Na]$^+$: 465.1426, found: 465.1428.

Spectral data for butyl 3-(2,2-diphenylacetyl)-4-hydroxy-1H-pyrazole-5-carboxylate (7d):

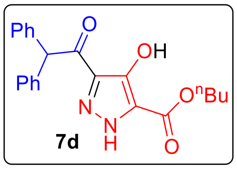

Purified by silica gel column, hexane/ethyl acetate (80:20) as eluent; White solid (79.0 mg, 54%); ^{1}H NMR (700 MHz, $CDCl_3$): δ 11.8 (s, 1H), 8.67 (s, 1H), 7.37 (d, J = 7.7 Hz, 4H), 7.30 (t, J = 7.0 Hz, 4H), 7.26 – 7.24 (m, 2H), 6.19 (s, 1H), 4.37 (t, J = 7.0 Hz, 2H), 1.76 – 1.72 (m, 2H), 1.45 – 1.42 (m, 2H), 0.94 (t, J = 7.0 Hz, 3H); ^{13}C NMR (175 MHz, $CDCl_3$): δ 147.9, 137.8, 129.1, 128.6, 127.3, 65.4, 58.6, 30.5, 18.9, 13.6 (Fewer signals than expected (C=O and three pyrazole carbons are missing) due to dilute sample and slow relaxation of these signals); HRMS (ESI-TOF)$^+$ calcd for $C_{22}H_{22}N_2NaO_4$ [M+Na]$^+$: 401.1477, found: 401.1476.

Spectral data for tert-butyl 3-(2,2-diphenylacetyl)-4-hydroxy-1H-pyrazole-5-carboxylate (7e):

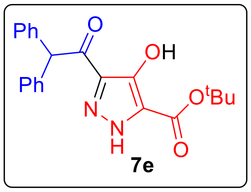

Purified by silica gel column, hexane/ethyl acetate (80:20) as eluent; White solid (65.3 mg, 43%); ^{1}H NMR (700 MHz, $CDCl_3$): δ 11.6 (s, 1H), 8.65 (s, 1H), 7.36 (d, J = 8.4 Hz, 4H), 7.30 (t, J = 7.7 Hz, 4H), 7.25 – 7.23 (m, 2H), 6.17 (s, 1H), 1.59 (s, 9H); ^{13}C NMR (175 MHz, $CDCl_3$): δ 147.8, 137.9, 129.1, 128.6, 127.2, 83.7, 58.6, 28.2 (Fewer signals than expected (C=O and three pyrazole carbons are missing) due to dilute sample and slow relaxation of these signals); HRMS (ESI-TOF)$^+$ calcd for $C_{22}H_{22}N_2NaO_4$ [M+Na]$^+$: 401.1477, found: 401.1478.

Spectral data for cyclohexyl 3-(2,2-diphenylacetyl)-4-hydroxy-1H-pyrazole-5-carboxylate (7f):

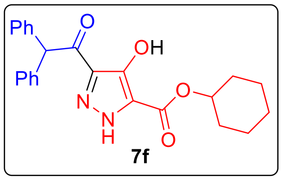

Purified by silica gel column, hexane/ethyl acetate (80:20) as eluent; White solid (76.0 mg, 47%); ^{1}H NMR (700 MHz, $CDCl_3$): δ 7.36 – 7.33 (m, 4H), 7.32 – 7.29 (m, 4H), 7.26 – 7.25 (m, 2H), 6.14 (s, 1H), 5.07 – 5.04 (m, 1H), 1.95 – 1.92 (m, 2H), 1.79 – 1.77 (m, 2H), 1.62 – 1.55 (m, 3H), 1.43 – 1.37 (m, 2H), 1.32 – 1.29 (m, 1H); ^{13}C NMR (175 MHz, $CDCl_3$): δ 147.8, 138.1, 137.8, 129.1, 128.66, 128.62, 127.4, 127.3, 74.5, 58.7, 31.4, 25.2, 23.6 (Fewer signals than expected (C=O peak is missing) due to dilute sample and slow relaxation of these signals); HRMS-ESI$^+$ calcd for $C_{24}H_{24}N_2NaO_4$ $[M+Na]^+$: 427.1634, found: 427.1629.

Spectral data for allyl 3-(2,2-diphenylacetyl)-4-hydroxy-1H-pyrazole-5-carboxylate (7g):

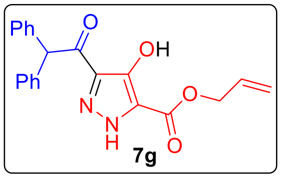

Purified by silica gel column, hexane/ethyl acetate (80:20) as eluent; White solid (72.7 mg, 50%); ^{1}H NMR (700 MHz, $CDCl_3$): δ 11.66 (s, 1H), 8.65 (s, 1H), 7.36 (d, *J* = 7.7 Hz, 4H), 7.30 (t, *J* = 7.7 Hz, 4H), 7.26 – 7.24 (m, 2H), 6.17 (s, 1H), 6.02 – 5.96 (m, 1H), 5.44 (d, *J* = 16.8 Hz, 1H), 5.30 (d, *J* = 10.5 Hz, 1H), 4.86 (d, *J* = 5.6 Hz, 2H); ^{13}C NMR (175 MHz, $CDCl_3$): δ 148.0, 137.8, 131.1, 129.1, 128.6, 127.3, 119.4, 66.5, 66.0, 65.5, 58.6 (Fewer signals than expected (C=O and three pyrazole carbons are missing) due to dilute sample and slow relaxation of these signals); HRMS (ESI-TOF)$^+$ calcd for $C_{21}H_{18}N_2NaO_4$ $[M+Na]^+$: 385.1164, found: 385.1168.

Spectral data for methyl 3-(2,2-diphenylacetyl)-4-hydroxy-1H-pyrazole-5-carboxylate (7h):

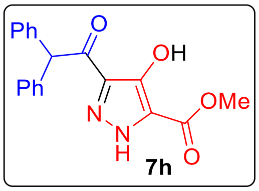

Purified by silica gel column, hexane/ethyl acetate (80:20) as eluent; White solid (89 mg, 66%); ^{1}H NMR (700 MHz, $CDCl_3$): δ 11.07 (s, 1H), 8.59 (s, 1H), 7.34 (d, *J* = 7.7 Hz, 4H), 7.30 (t, *J* = 7.0 Hz, 4H), 7.26 – 7.24 (m, 2H), 6.12 (s, 1H), 3.95 (s, 3H); ^{13}C NMR (175 MHz, $CDCl_3$): δ 137.8, 129.1, 128.6, 127.4, 52.4 (Fewer signals than expected (C=O and CO_2Et carbonyl carbons, CH, and three pyrazole carbons are missing) due to dilute sample and slow relaxation of these signals); HRMS (ESI-TOF)$^+$ calcd for $C_{19}H_{16}N_2NaO_4$ $[M+Na]^+$: 359.1008, found: 359.1009.

Spectral data for ethyl-3-((tert-butyldimethylsilyl)oxy)-5-hydroxy-2,3-diphenylcyclopent-1-ene-1-carboxylate (8a):

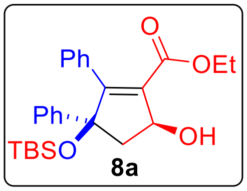

Purified by silica gel column, hexane/ethyl acetate (90:10) as eluent; Colorless liquid (34 mg, 68%); ^{1}H NMR (700 MHz, $CDCl_3$): δ 7.28 – 7.26 (m, 2H), 7.25 (s, 1H), 7.23 – 7.21 (m, 3H), 7.15 (t, *J* = 7.7 Hz, 2H), 6.90 (d, *J* = 7.0 Hz, 2H), 5.22 – 5.20 (m, 1H), 4.08 – 4.01 (m, 2H), 3.55 (d, *J* = 1.4 Hz, 1H), 3.00 – 2.97 (m, 1H), 2.49 – 2.46 (m, 1H), 0.98 (t, *J* = 7.0 Hz, 3H), 0.77 (s, 9H), 0.005 (s, 3H), -0.02 (s, 3H); ^{13}C NMR (175 MHz, $CDCl_3$): δ 166.1, 160.0, 145.5, 133.5, 130.5, 129.5, 128.1, 128.0, 127.2, 126.8, 125.1, 86.6, 73.4, 60.6, 49.9, 25.8, 18.3, 13.5, -2.4, -2.6; HRMS (ESI-TOF)$^+$ calcd for $C_{26}H_{34}NaO_4Si$ $[M+Na]^+$: 461.2124, found: 461.2125.

Spectral data for ethyl-3-hydroxy-3-(4-methoxyphenyl)-5-oxo-2-phenylcyclopent-1-ene-1-carboxylate (8b):

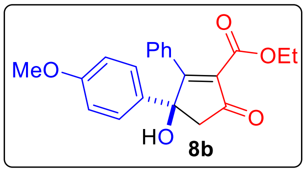

Purified by silica gel column, hexane/ethyl acetate (85:15) as eluent; White solid (35.0 mg, 93%); ^{1}H NMR (400 MHz, $CDCl_3$): δ 7.35 – 7.28 (m, 3H), 7.26 – 7.23 (m, 2H), 7.16 – 7.13 (m, 2H), 6.89 – 6.86 (m, 2H), 4.23 – 4.16 (m, 2H), 3.78 (s, 3H), 3.10 (d, *J* = 18.4 Hz, 1H), 2.99 (d, *J* = 18.4 Hz, 1H), 2.59 (s, 1H), 1.13 (t, *J* = 7.2 Hz, 3H); ^{13}C NMR (100 MHz, $CDCl_3$): δ 199.6, 173.7, 163.7, 159.1, 134.5, 134.2, 131.3, 130.4, 128.6, 128.4, 125.8, 114.3, 80.7, 61.5, 55.2, 55.0, 13.8; HRMS (ESI-TOF)$^+$ calcd for $C_{21}H_{20}NaO_5$ $[M+Na]^+$: 375.1208, found: 375.1202.

Spectral data for methyl 5-(2,2-diphenylacetyl)-4-hydroxy-1H-pyrazole-3-carboxylate (9a):

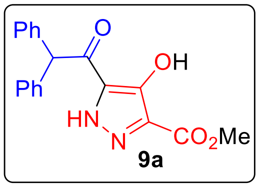

Purified by silica gel column, hexane/ethyl acetate (80:20) as eluent; White solid (43.3 mg, 90%); ^{1}H NMR (400 MHz, $CDCl_3$): δ 11.3 (s, 1H), 8.6 (s, 1H), 7.35 – 7.33 (m, 4H), 7.31 – 7.28 (m, 4H), 7.25 – 7.21 (m, 2H), 6.12 (s, 1H), 3.94 (s, 3H); ^{13}C NMR (100 MHz, $CDCl_3$): δ 147.7, 137.8, 129.1, 128.6, 127.3, 58.7, 52.4 (Fewer signals than expected (C=O and three pyrazole carbons are missing) due to dilute sample and slow relaxation of these signals); HRMS (ESI-TOF)$^+$ calcd for $C_{19}H_{16}N_2NaO_4$ $[M+Na]^+$: 359.1008, found: 359.1005.

Spectral data for ethyl 3-(2,2-diphenylacetyl)-4-methoxy-1-methyl-1H-pyrazole-5-carboxylate (9b):

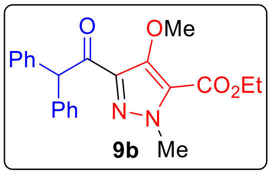

Purified by silica gel column, hexane/ethyl acetate (95:5) as eluent; Colorless liquid (31.3 mg, 83%); ^{1}H NMR (700 MHz, $CDCl_3$): δ 7.34 (d, *J* = 7.7 Hz, 4H), 7.28 (t, *J* = 7.7 Hz, 4H), 7.20 (t, *J* = 7.0 Hz, 2H), 6.3 (s, 1H), 4.36 – 4.33 (m, 2H), 4.09 (s, 3H), 3.87 (s, 3H), 1.36 (t, *J* = 7.0 Hz, 3H); ^{13}C NMR (175 MHz, $CDCl_3$): δ 192.6, 159.1, 149.1, 139.7, 139.0, 129.2, 128.4, 126.9, 125.2, 62.8, 61.2, 58.8, 41.6, 14.1; HRMS $(ESI\text{-}TOF)^+$ calcd for $C_{22}H_{22}N_2NaO_4$ $[M+Na]^+$: 401.1477, found: 401.1478.

Spectral data for methyl 5-(2,2-diphenylacetyl)-4-methoxy-1-methyl-1H-pyrazole-3-carboxylate (9c):

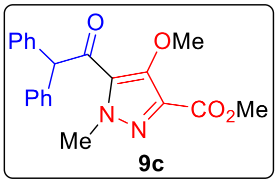

Purified by silica gel column, hexane/ethyl acetate (95:5) as eluent; Colorless liquid (32.6 mg, 86%); ^{1}H NMR (700 MHz, $CDCl_3$): δ 7.34 (d, *J* = 7.7 Hz, 4H), 7.28 (t, *J* = 7.7 Hz, 4H), 7.20 (t, *J* = 7.7 Hz, 2H), 6.3 (s, 1H), 4.10 (s, 3H), 3.89 (d, *J* = 4.2 Hz, 6H); ^{13}C NMR (175 MHz, $CDCl_3$): δ 192.6, 159.7, 149.2, 139.6, 139.0, 129.2, 128.4, 126.9, 124.8, 62.9, 58.8, 52.1, 41.7; HRMS $(ESI\text{-}TOF)^+$ calcd for $C_{21}H_{20}N_2O_4Na$ $[M+Na]^+$: 387.1321, found: 387.1320.

(8) ^{1}H-NOE data for compound 3n, 3n', 5c' and 8a:

8.1. ^{1}H-NOE data of 3n (Major isomer):

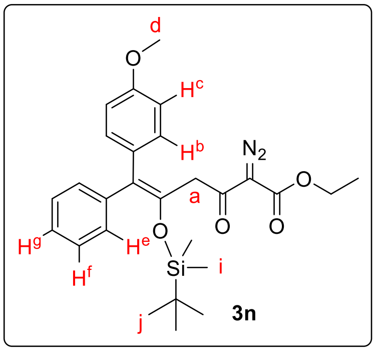

No.	Irradiate	Intensity Enhancement
1.	H^{a}, (δ: 3.77)	H^{b}, (δ: 7.07, 1.30 %), H^{i}, (δ: 0.73, 0.77 %)
2.	H^{a}, (δ: 3.77)	H^{a}, (δ: 3.77, 0.77 %), H^{c}, (δ: 6.77, 2.65 %)
3.	H^{c}, (δ: 6.77)	H^{b}, (δ: 7.07, 3.60 %), H^{d}, (δ: 3.75, 1.97 %)
4.	H^{d}, (δ: 3.75)	H^{c}, (δ: 6.77, 1.50 %)
5.	H^{j}, (δ: 0.73)	H^{i}, (δ: -0.15, 0.50 %), H^{a}, (δ: 3.77,0.12 %), H^{e}, (δ: 7.20, 0.07 %), H^{f}, (δ: 7.27, 0.11 %), H^{g}, (δ: 7.10, 0.05 %),

8.2. ^{1}H-NOE data of 3n' (Minor isomer):

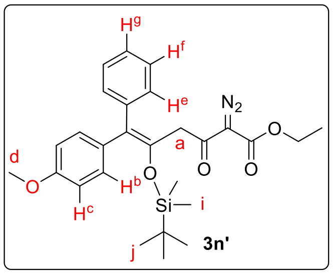

No.	Irradiate	Intensity Enhancement
1.	H^{i}, (δ: -0.12)	H^{j} (δ: 0.76, 2.16%), H^{a} (δ: 3.75, 1.55%), H^{c} (δ: 6.76, 0.95%), H^{d} (δ: 7.16, 0.90%)
2.	H^{j}, (δ: 0.76)	H^{a} (δ: -0.12, 1.28%), H^{a} (δ: 3.75, 0.69%), H^{c} (δ: 6.76, 0.63%), H^{d} (δ: 7.16, 0.70%)

8.3. ^{1}H-NOE data of 5c' (Minor isomer):

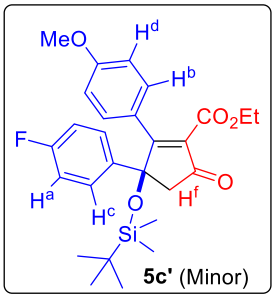

No.	Irradiate	Intensity Enhancement
1.	H^{a}, (δ: 7.38)	H^{b} (δ: 7.29, 1.02%), H^{c} (δ: 6.99, 2.89%), H^{f} (δ: 2.83, 0.98%)
2.	H^{b}, (δ: 7.29)	H^{d} (δ: 6.73, 2.70%), No H^{f} proton affected
3.	H^{c}, (δ: 6.99)	H^{a} (δ: 7.38, 2.69%), H^{f} (δ: 3.10, 0.17%), H^{f} (δ: 2.83, 0.30%)

8.3. ^{1}H-NOE data of 8a:

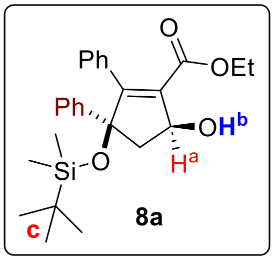

No.	Irradiate	Intensity Enhancement
1.	H^{b} (δ: 3.55)	H^{a} (δ: 5.22, 5.75%), H^{c} (δ: 0.77, 2.50%), No phenyl proton affected
2.	H^{a} (δ: 5.22)	H^{b} (δ: 3.55, 3.67%), H^{c} (δ: 0.77, 1.06%), phenyl proton affected

(9) X-ray Crystallographic Structure and data for compound 5a, 6g, 8b and 9a:

9.1. X-ray Crystallographic data for compound 5a:

Ellipsoid contour % probability level = 50%

Sample Preparation for Crystal Growth: The compound **5a** was dissolved in 0.4 mL of $CDCl_3$ in a NMR tube, and was kept inside the refrigerator for 3 months.

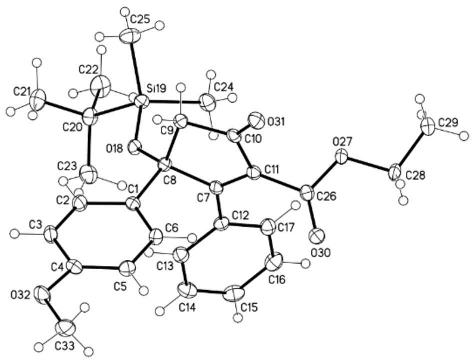

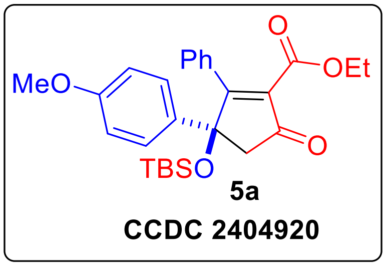

Table 1 Crystal data and structure refinement for 2410107lt_auto.

Identification code	2410107lt_auto
Empirical formula	$C_{27}H_{34}O_5Si$
Formula weight	466.63
Temperature/K	100.02(12)
Crystal system	triclinic
Space group	P-1
a/Å	9.75810(10)
b/Å	10.86410(10)
c/Å	13.90890(10)
α/°	98.9260(10)
β/°	102.4320(10)
γ/°	115.1510(10)
Volume/Å^3	1251.66(2)
Z	2
ρ_{calc}g/cm^3	1.238
μ/mm^{-1}	1.109
F(000)	500.0
Crystal size/mm^3	0.16 × 0.15 × 0.11
Radiation	Cu Kα (λ = 1.54184)
2Θ range for data collection/°	9.366 to 147.7
Index ranges	$-11 \leq h \leq 12$, $-13 \leq k \leq 13$, $-16 \leq l \leq 17$
Reflections collected	17620
Independent reflections	4797 [R_{int} = 0.0176, R_{sigma} = 0.0163]

Data/restraints/parameters	4797/0/306
Goodness-of-fit on F^2	1.045
Final R indexes [I>=2σ (I)]	R_1 = 0.0297, wR_2 = 0.0729
Final R indexes [all data]	R_1 = 0.0310, wR_2 = 0.0737
Largest diff. peak/hole / e Å^{-3}	0.36/-0.26

9.2. X-ray Crystallographic data for compound 6g:

Ellipsoid contour % probability level = 50%

Sample Preparation for Crystal Growth: The compound **6g** was dissolved in 0.3 mL of DCM in a 5 mL vial, and this small vial was kept inside the 20 mL vial containing 10 mL of pentane and this system was left for 4 days inside the refrigerator.

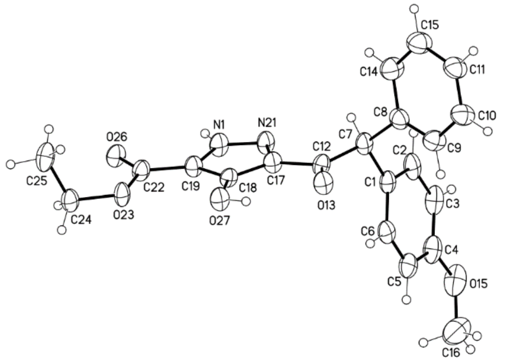

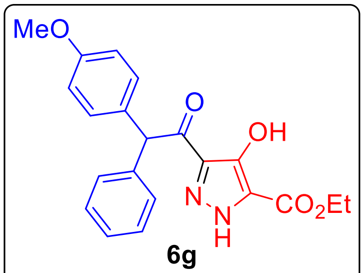

6g

CCDC 2393655

Crystal data and structure refinement for 230220lt_auto.

Identification code	230220lt_auto
Empirical formula	$C_{42}H_{40}N_4O_{10}$
Formula weight	760.78
Temperature/K	99.99(10)
Crystal system	monoclinic
Space group	$P2_1/c$
a/Å	7.1889(5)
b/Å	27.4282(19)
c/Å	19.4023(12)
α/°	90
β/°	99.675(7)
γ/°	90
Volume/Å^3	3771.3(4)
Z	4
ρ_{calc}g/cm^3	1.340
μ/mm^{-1}	0.799
F(000)	1600.0

Crystal size/mm^3	0.05 × 0.01 × 0.01
Radiation	Cu Kα (λ = 1.54184)
2Θ range for data collection/°	5.634 to 134.108
Index ranges	-8 ≤ h ≤ 8, -32 ≤ k ≤ 32, -17 ≤ l ≤ 23
Reflections collected	29356
Independent reflections	6727 [R_{int} = 0.0543, R_{sigma} = 0.0535]
Data/restraints/parameters	6727/265/594
Goodness-of-fit on F^2	1.263
Final R indexes [I>=2σ (I)]	R_1 = 0.1274, wR_2 = 0.3366
Final R indexes [all data]	R_1 = 0.1893, wR_2 = 0.3792
Largest diff. peak/hole / e Å^{-3}	0.87/-0.35

9.3. X-ray Crystallographic data for compound 8b:

Ellipsoid contour % probability level = 50%

Sample Preparation for Crystal Growth: The compound **8b** was dissolved in 0.3 mL of DCM in a 5 mL vial, and this small vial was kept inside the 20 mL vial containing 10 mL of pentane and this system was left for 3 days inside the refrigerator.

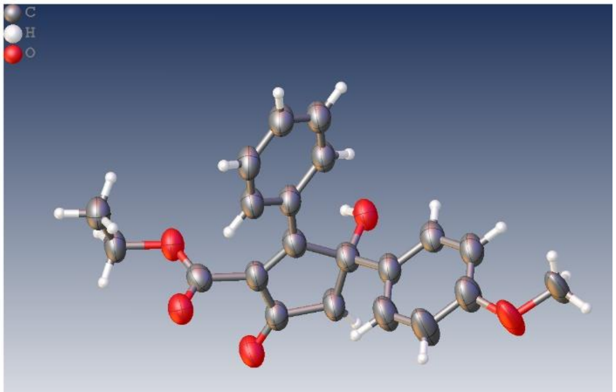

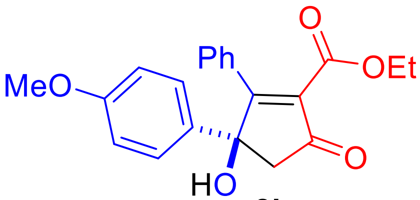

8b

CCDC 2393656

Crystal data and structure refinement for 240219LT_auto.

Identification code	240219LT_auto
Empirical formula	$C_{21}H_{20}O_5$
Formula weight	352.37
Temperature/K	100.00(10)
Crystal system	monoclinic
Space group	C2/c
a/Å	26.1025(18)
b/Å	7.9144(5)
c/Å	17.6627(15)
α/°	90
β/°	97.989(8)

γ/°	90
Volume/Å^3	3613.4(5)
Z	8
ρ_{calc}g/cm^3	1.295
μ/mm^{-1}	0.757
F(000)	1488.0
Crystal size/mm^3	0.17 × 0.03 × 0.01
Radiation	Cu Kα (λ = 1.54184)
2Θ range for data collection/°	6.84 to 134.14
Index ranges	-31 ≤ h ≤ 30, -5 ≤ k ≤ 9, -21 ≤ l ≤ 20
Reflections collected	24057
Independent reflections	3219 [R_{int} = 0.0561, R_{sigma} = 0.0247]
Data/restraints/parameters	3219/43/258
Goodness-of-fit on F^2	1.435
Final R indexes [I>=2σ (I)]	R_1 = 0.1037, wR_2 = 0.3390
Final R indexes [all data]	R_1 = 0.1218, wR_2 = 0.3557
Largest diff. peak/hole / e Å^{-3}	0.34/-0.45

9.4. X-ray Crystallographic data for compound 9a:

Ellipsoid contour % probability level = 50%

Sample Preparation for Crystal Growth: The compound **9a** was dissolved in 0.3 mL of DCM in a 5 mL vial, and this small vial was kept inside the 20 mL vial containing 10 mL of pentane and this system was left for 2 days inside the refrigerator.

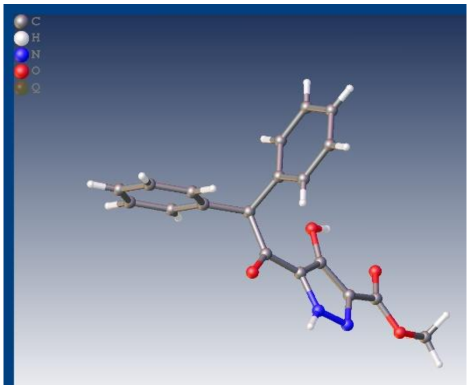

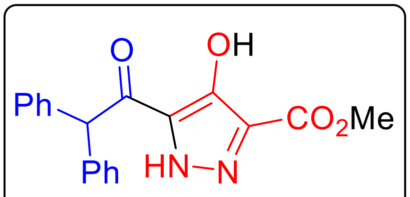

9a

CCDC 2393569

Crystal data and structure refinement for 240882lt.

Identification code	240882lt
Empirical formula	$C_{20}H_{17}Cl_3N_2O_4$
Formula weight	455.70
Temperature/K	100.00(13)
Crystal system	monoclinic
Space group	P2/c

a/Å	11.31310(10)
b/Å	7.12250(10)
c/Å	28.6273(3)
α/°	90
β/°	100.7780(10)
γ/°	90
Volume/Å^3	2266.03(5)
Z	4
ρ_{calc}g/cm^3	1.336
μ/mm^{-1}	3.900
F(000)	936.0
Crystal size/mm^3	0.19 × 0.05 × 0.02
Radiation	Cu Kα (λ = 1.54184)
2Θ range for data collection/°	7.956 to 149.034
Index ranges	$-13 \leq h \leq 13$, $-7 \leq k \leq 8$, $-34 \leq l \leq 34$
Reflections collected	30165
Independent reflections	4478 [R_{int} = 0.0302, R_{sigma} = 0.0151]
Data/restraints/parameters	4478/0/265
Goodness-of-fit on F^2	1.088
Final R indexes [I>=2σ (I)]	R_1 = 0.0440, wR_2 = 0.1253
Final R indexes [all data]	R_1 = 0.0467, wR_2 = 0.1274
Largest diff. peak/hole / e Å^{-3}	0.83/-0.79

^{1}H and ^{13}C spectra of key compounds:

^{1}H NMR ($CDCl_3$, 400 MHz)

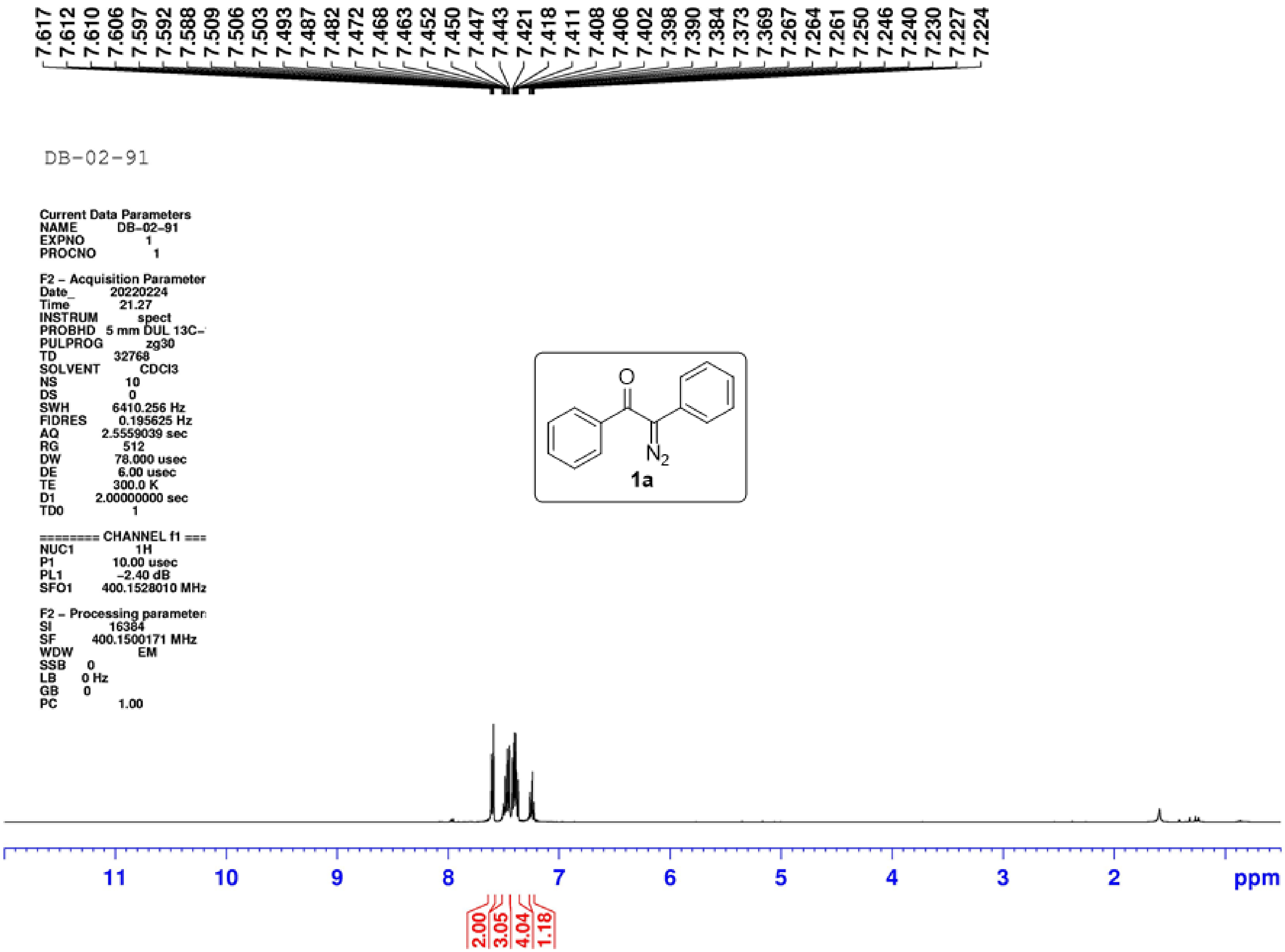

^{13}C NMR ($CDCl_3$, 100 MHz)

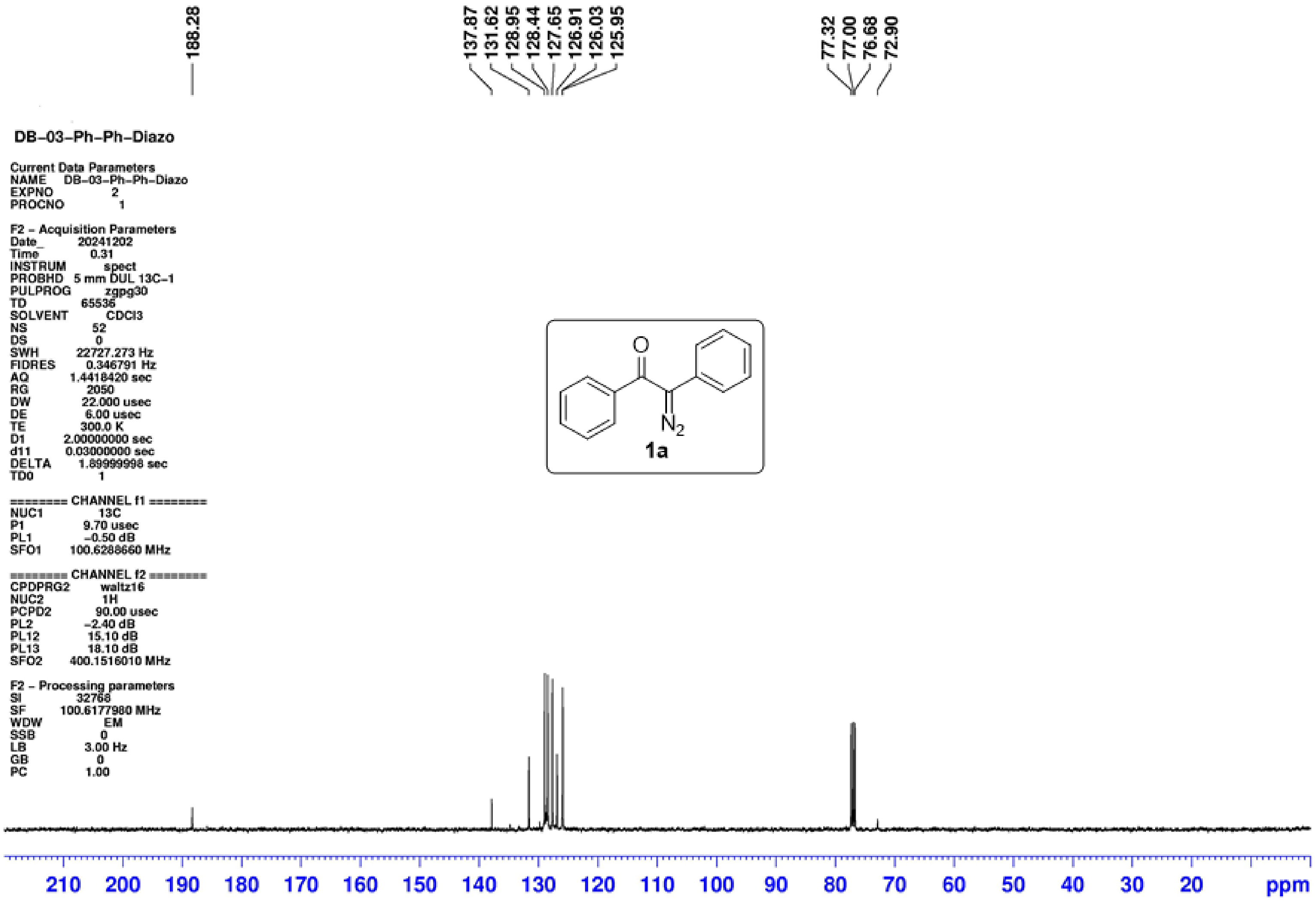

^{1}H NMR ($CDCl_3$, 400 MHz)

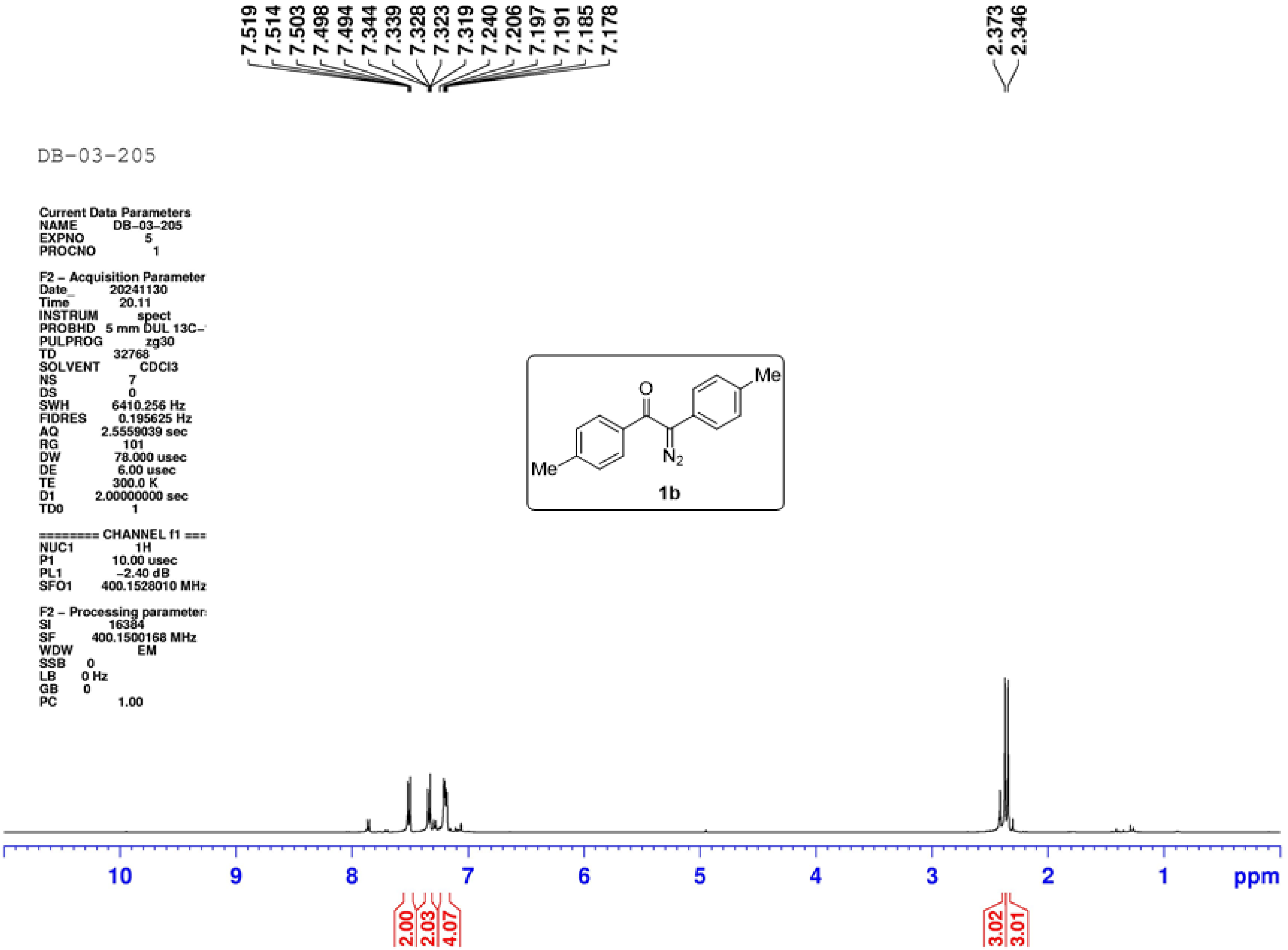

^{13}C NMR (CDCl$_3$, 100 MHz)

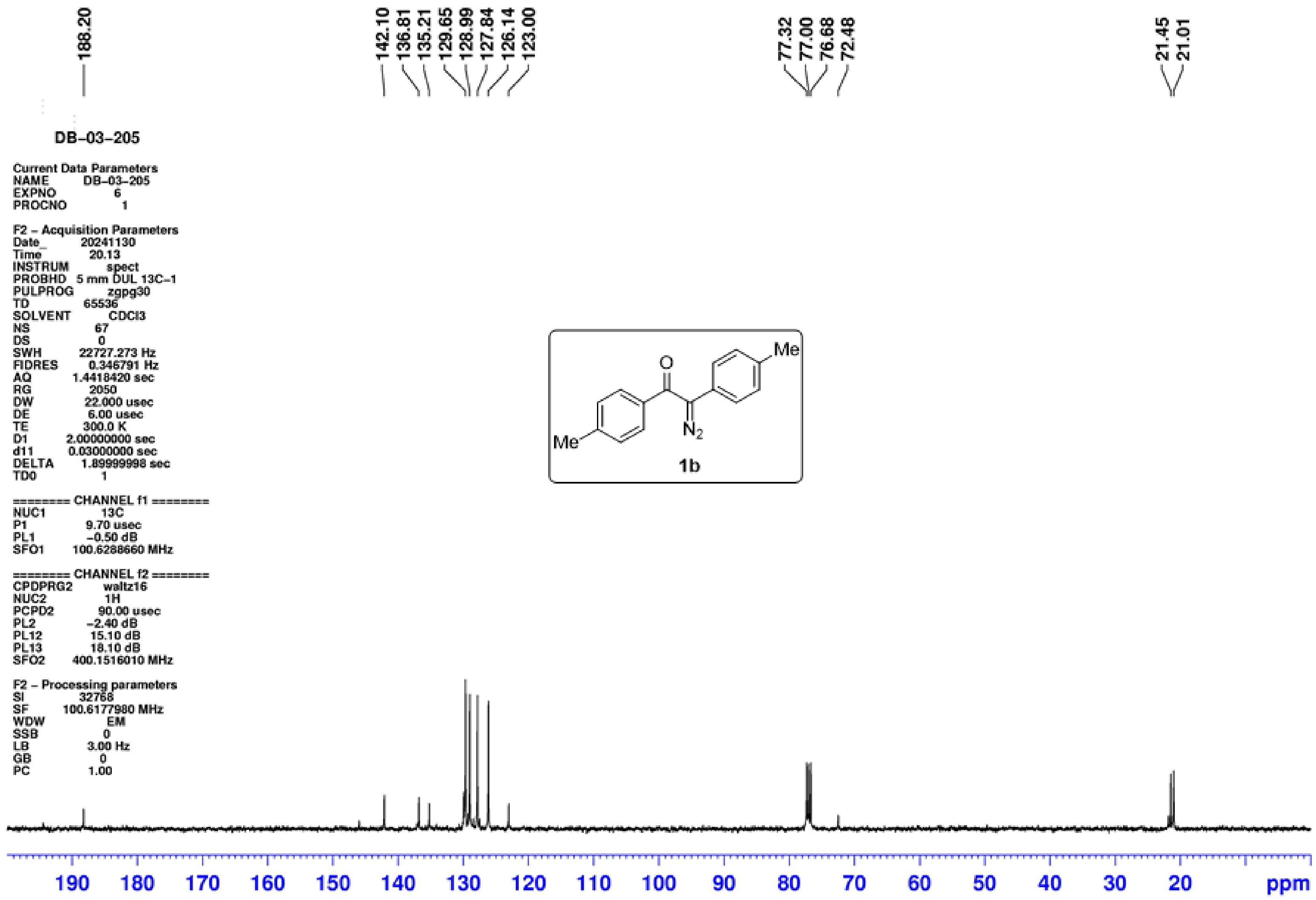

^{1}H NMR ($CDCl_3$, 400 MHz)

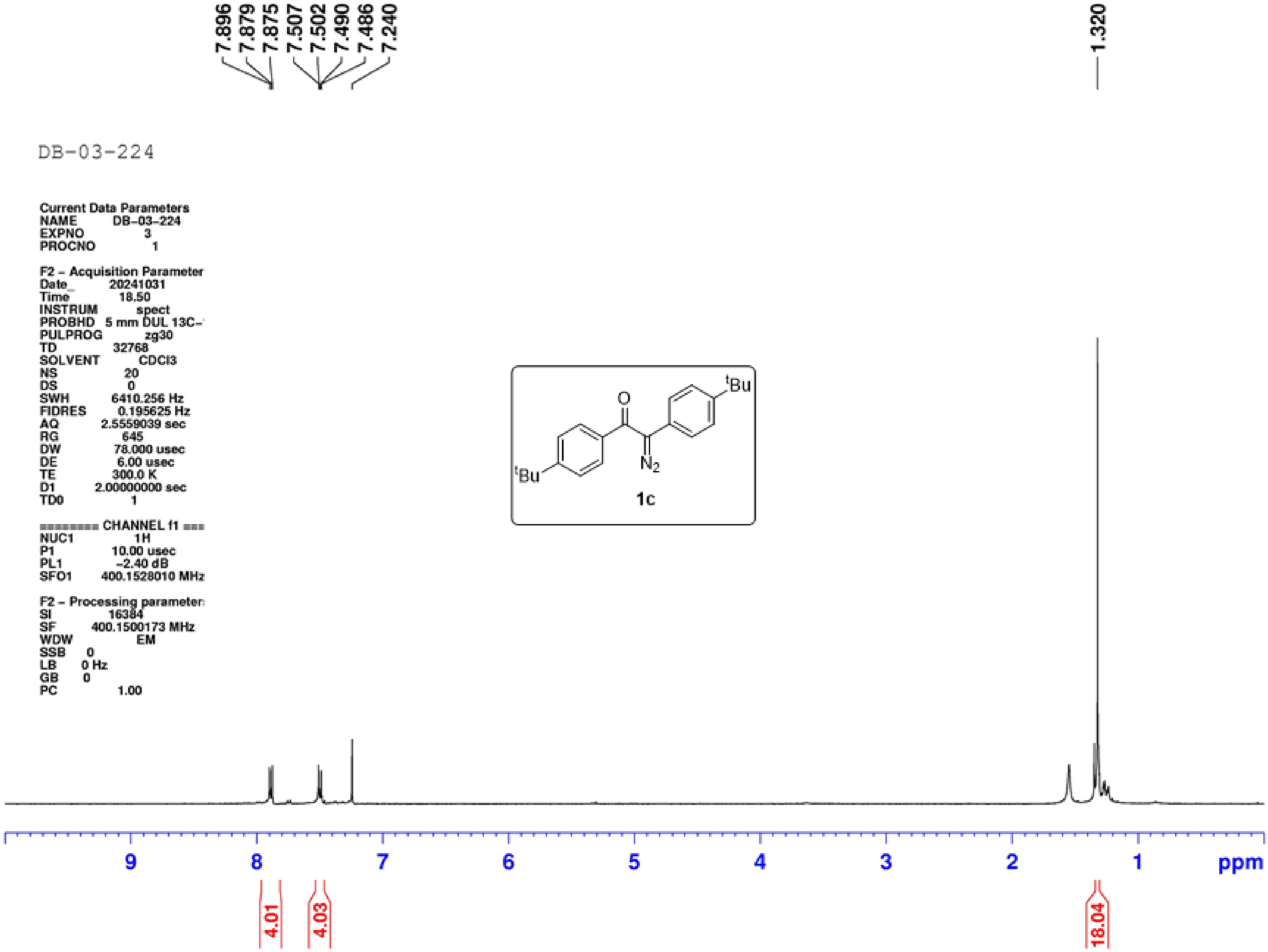

^{13}C NMR ($CDCl_3$, 100 MHz)

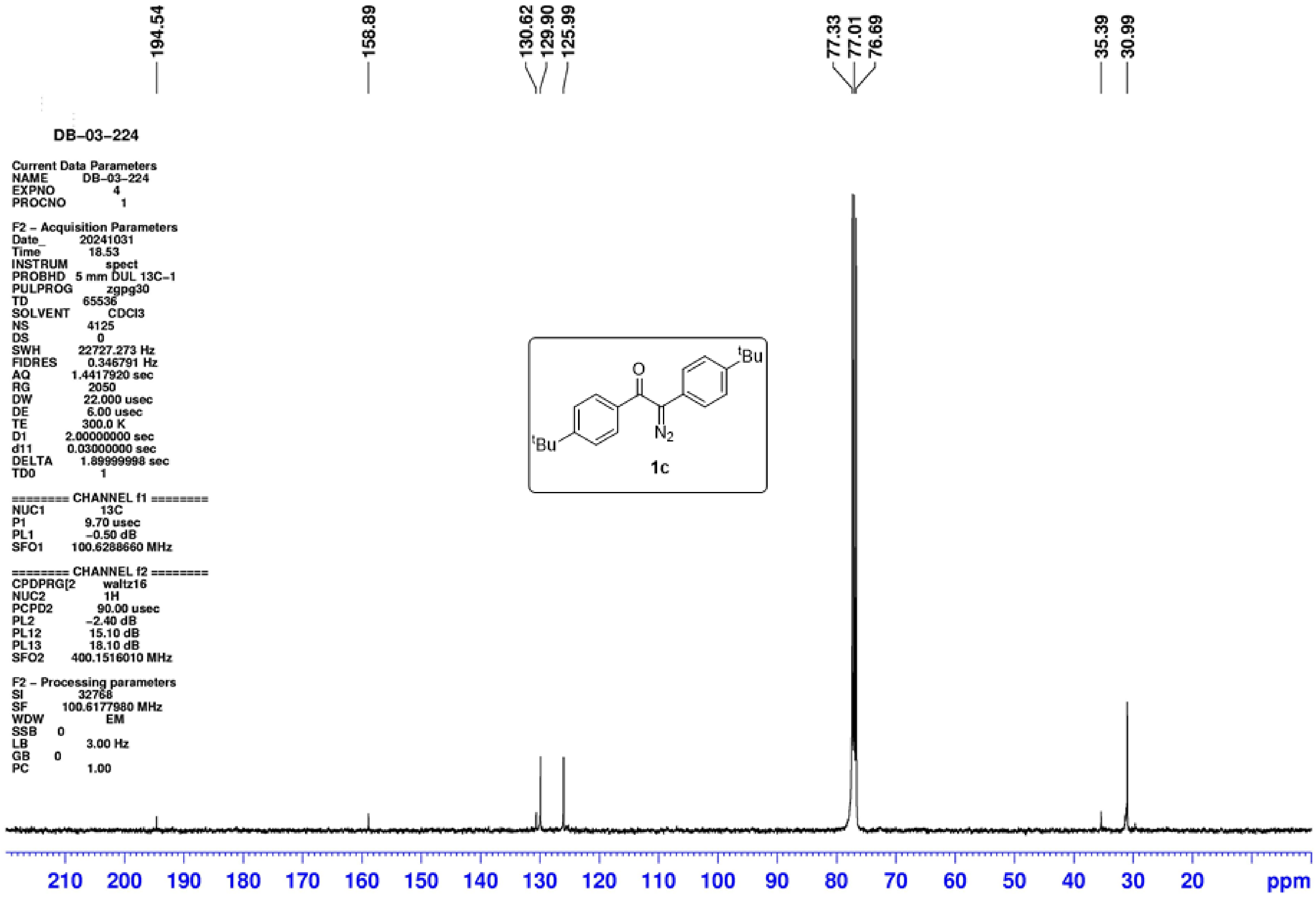

^{1}H NMR ($CDCl_3$, 400 MHz)

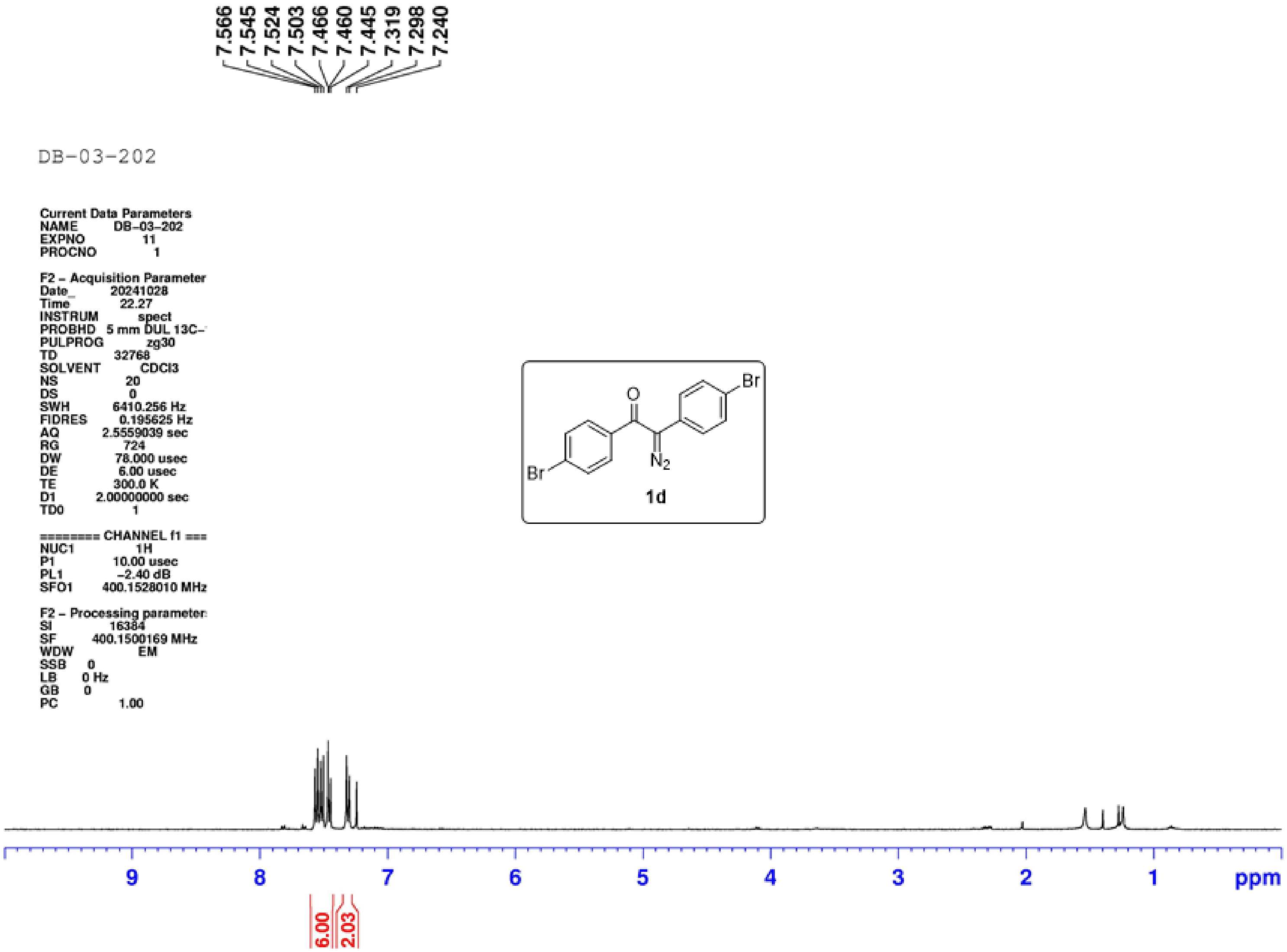

^{13}C NMR ($CDCl_3$, 100 MHz)

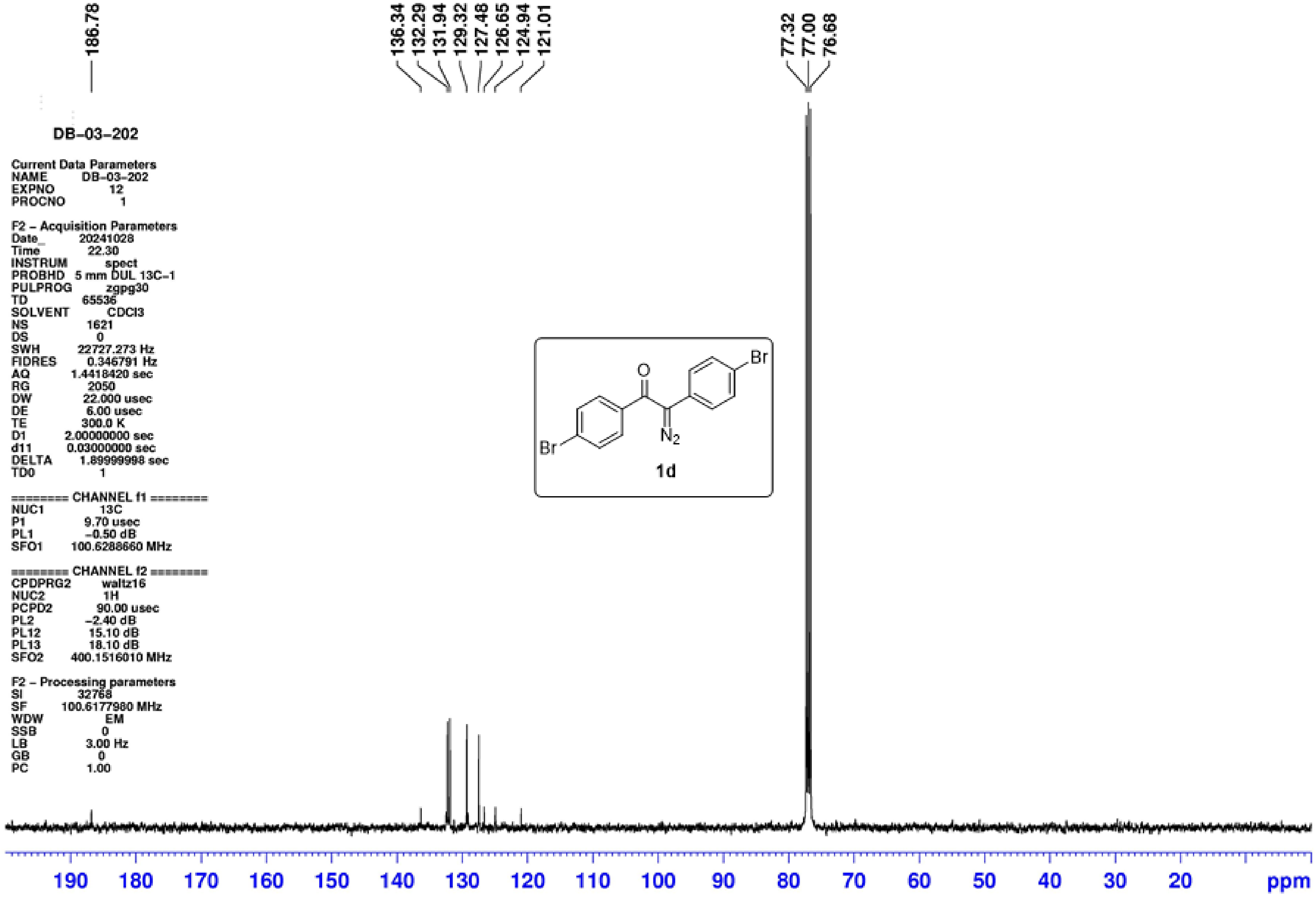

^{1}H NMR ($CDCl_3$, 400 MHz)

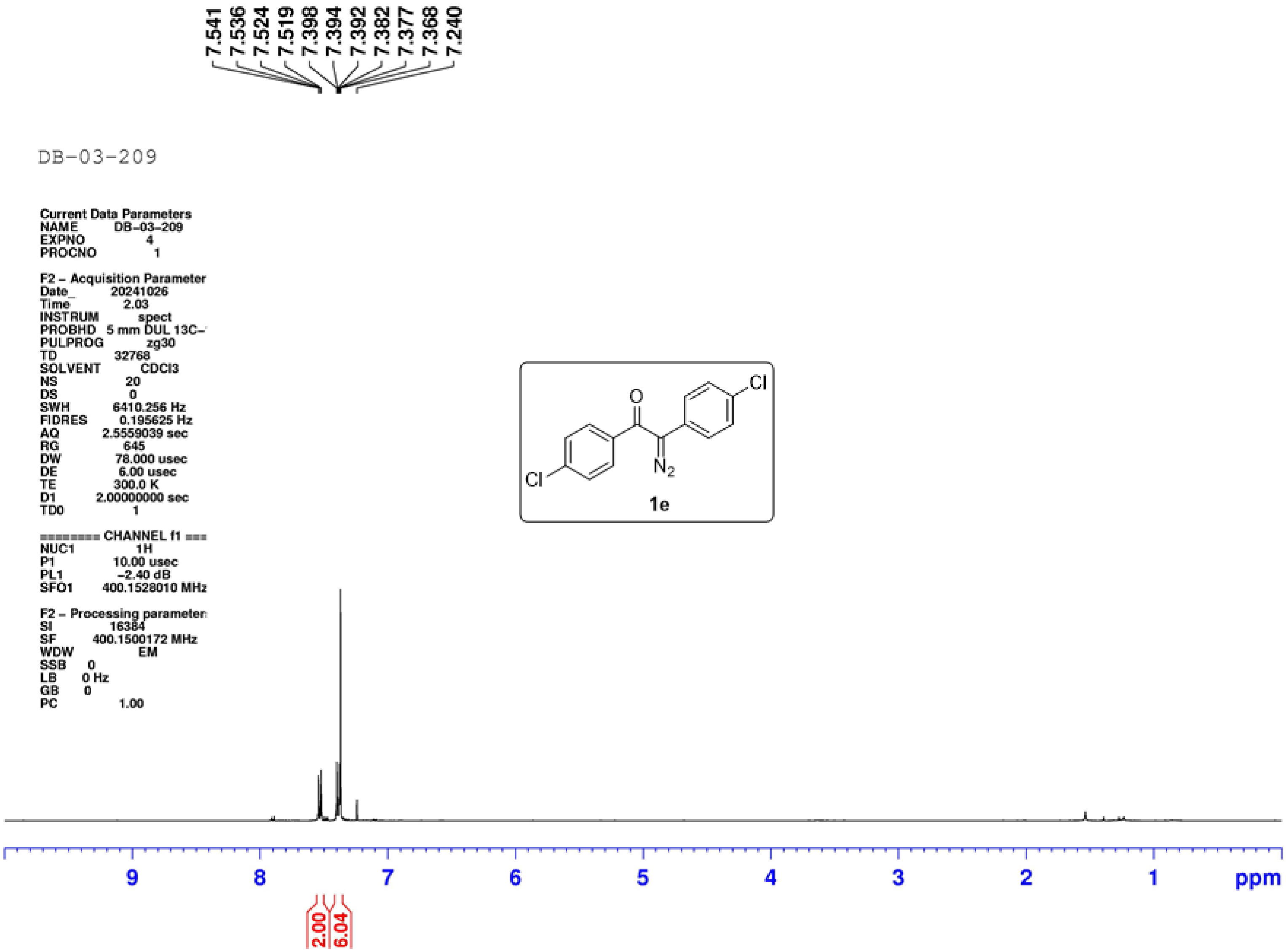

^{13}C NMR ($CDCl_3$, 100 MHz)

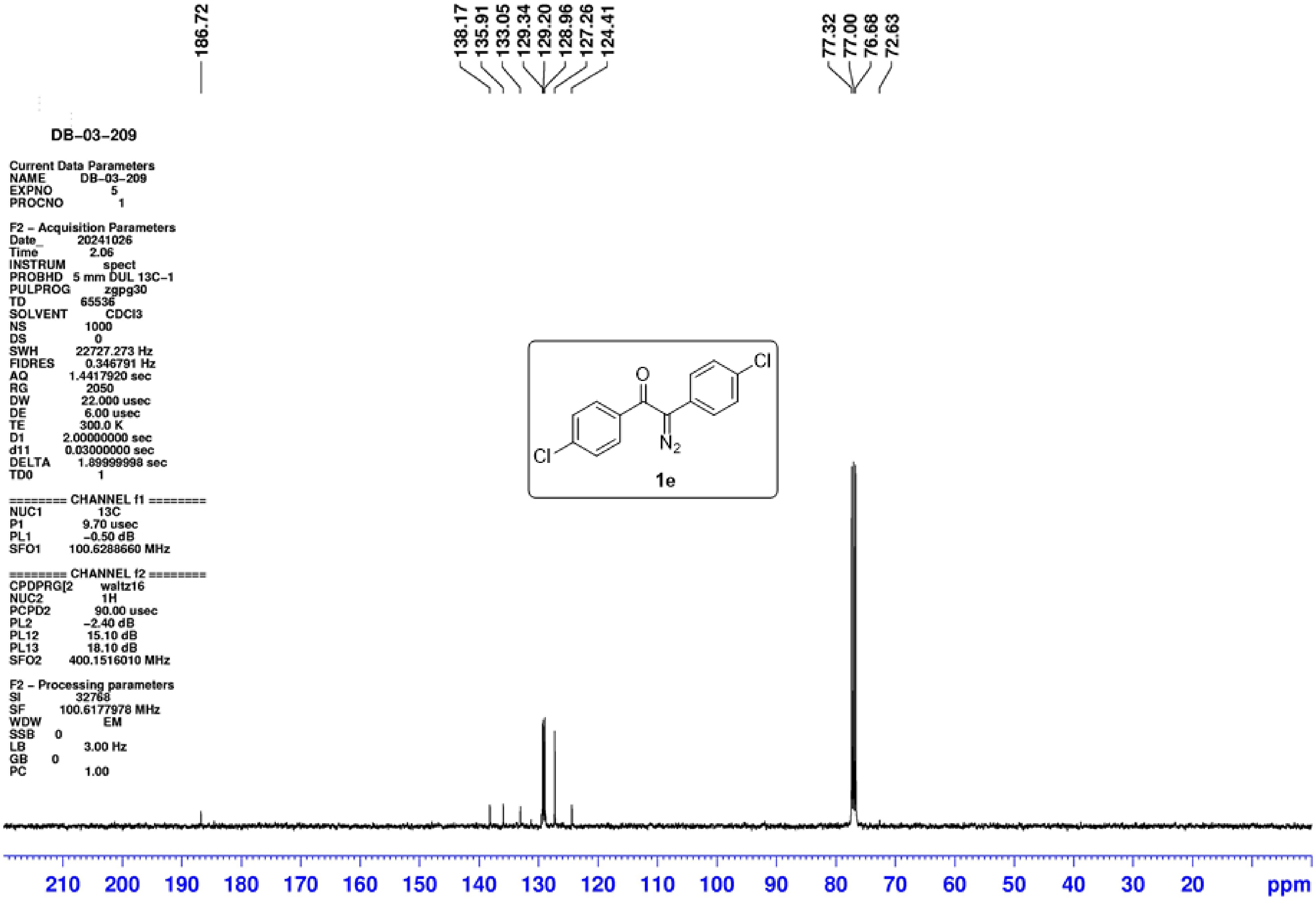

^{1}H NMR ($CDCl_3$, 400 MHz)

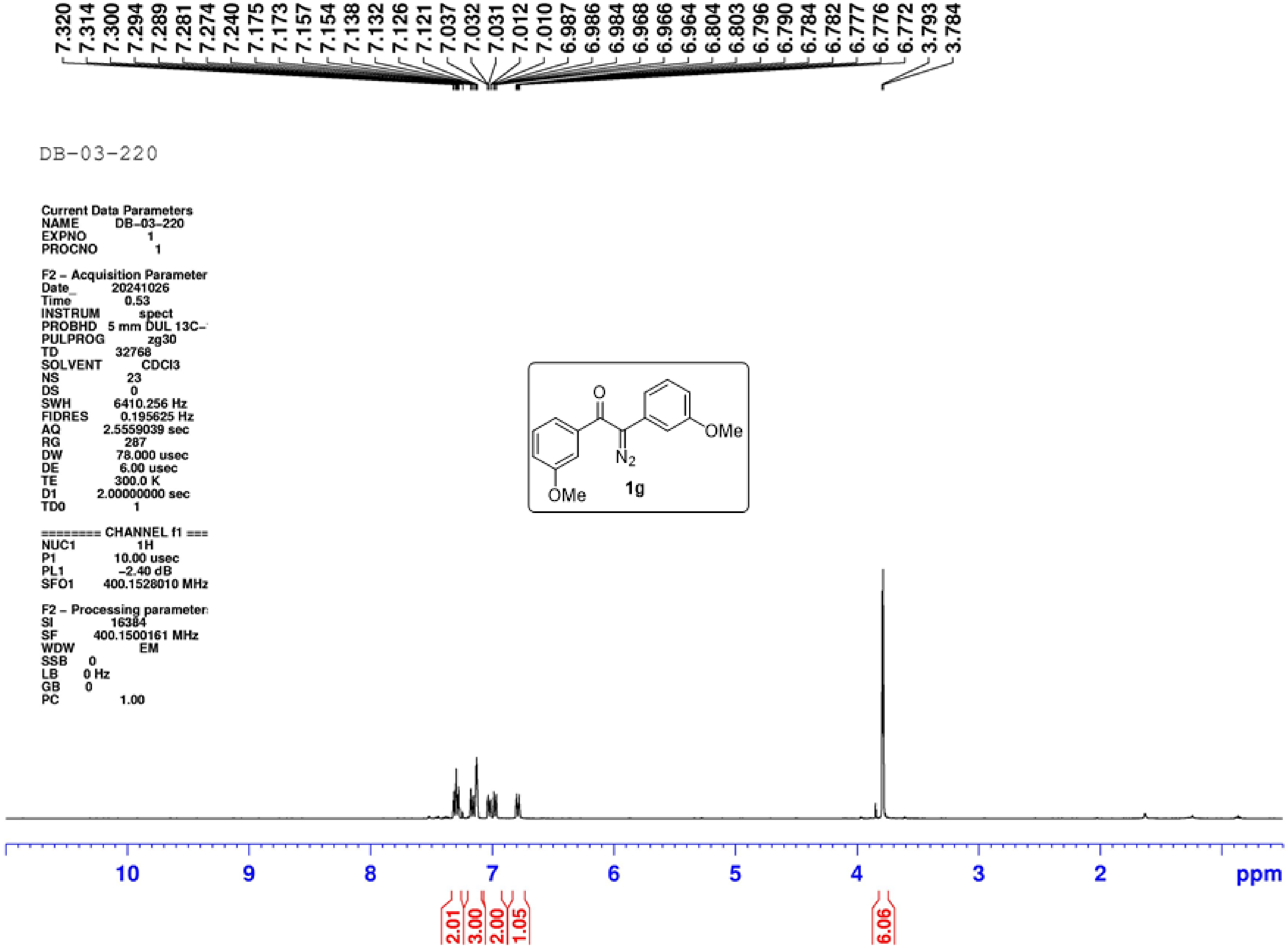

^{13}C NMR ($CDCl_3$, 100 MHz)

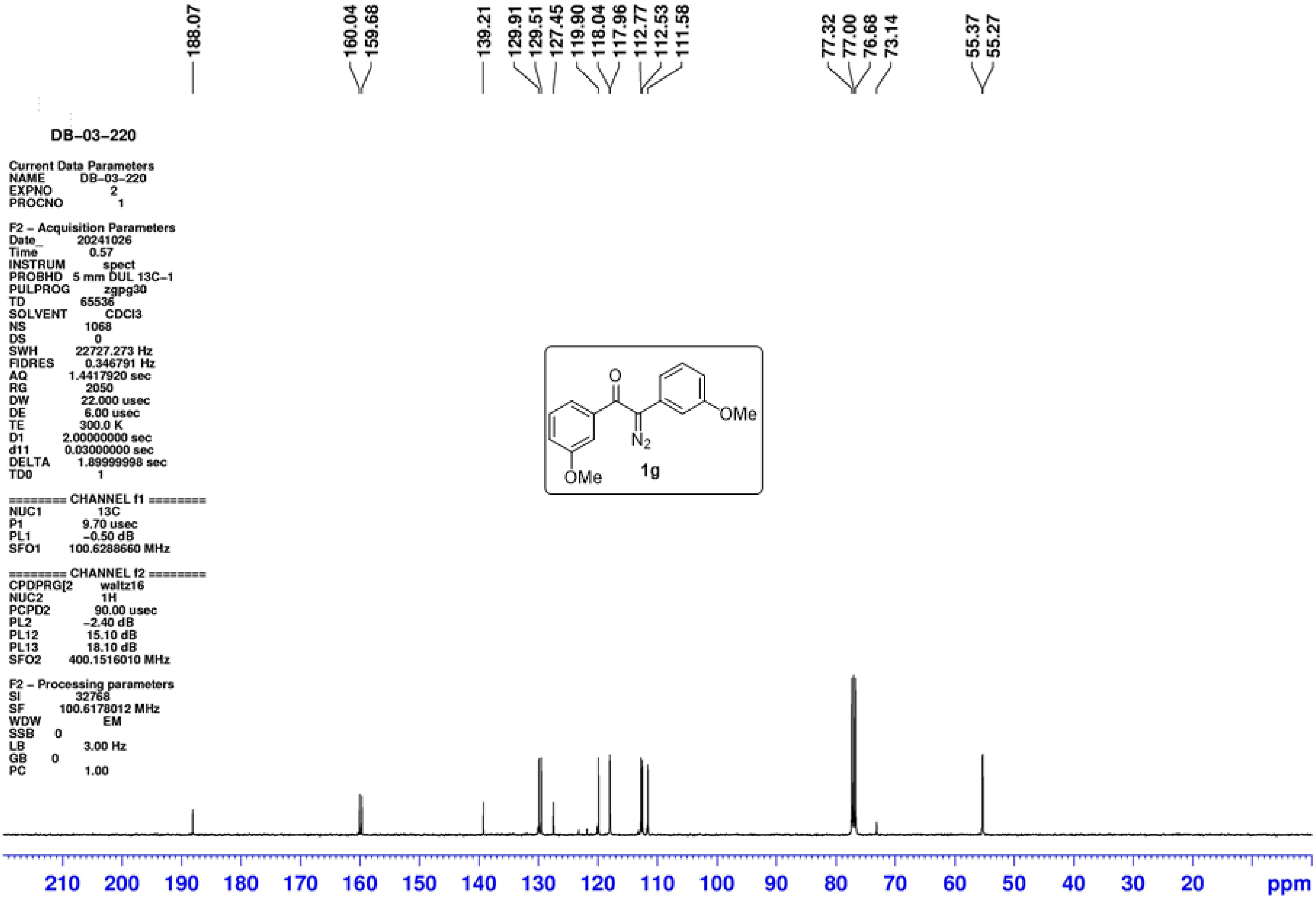

^{1}H NMR ($CDCl_3$, 400 MHz)

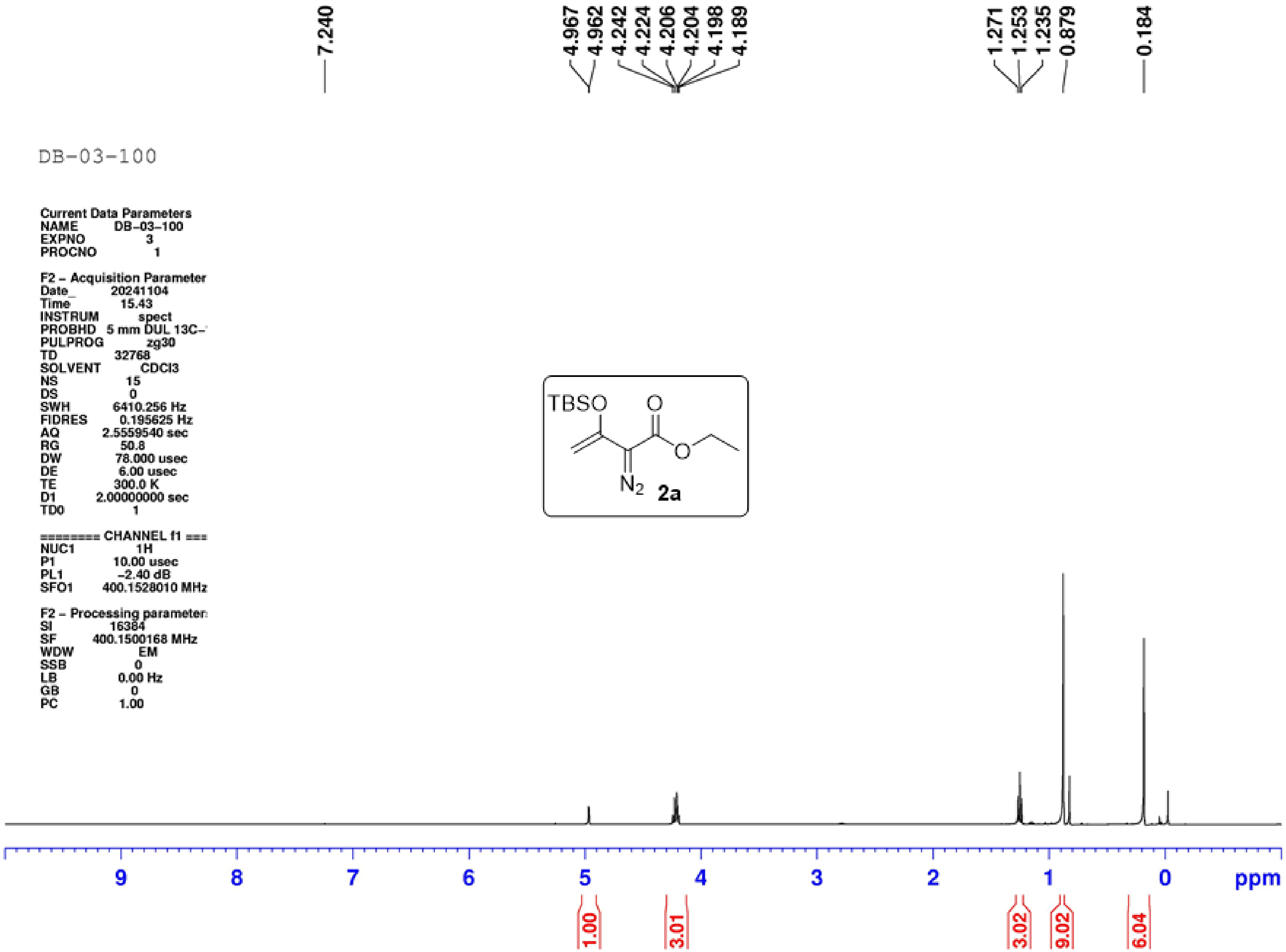

^{13}C NMR ($CDCl_3$, 100 MHz)

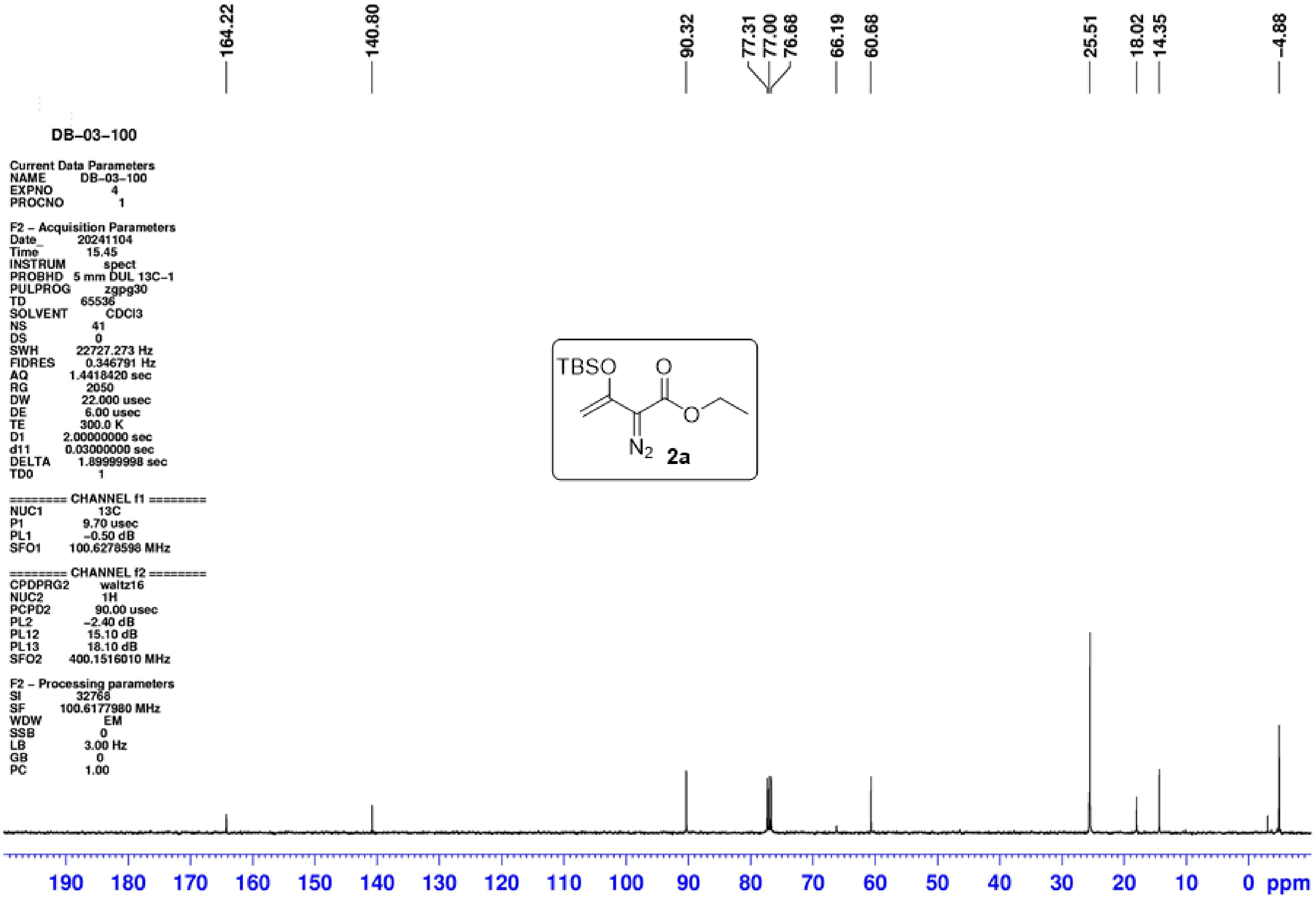

^{1}H NMR ($CDCl_3$, 400 MHz)

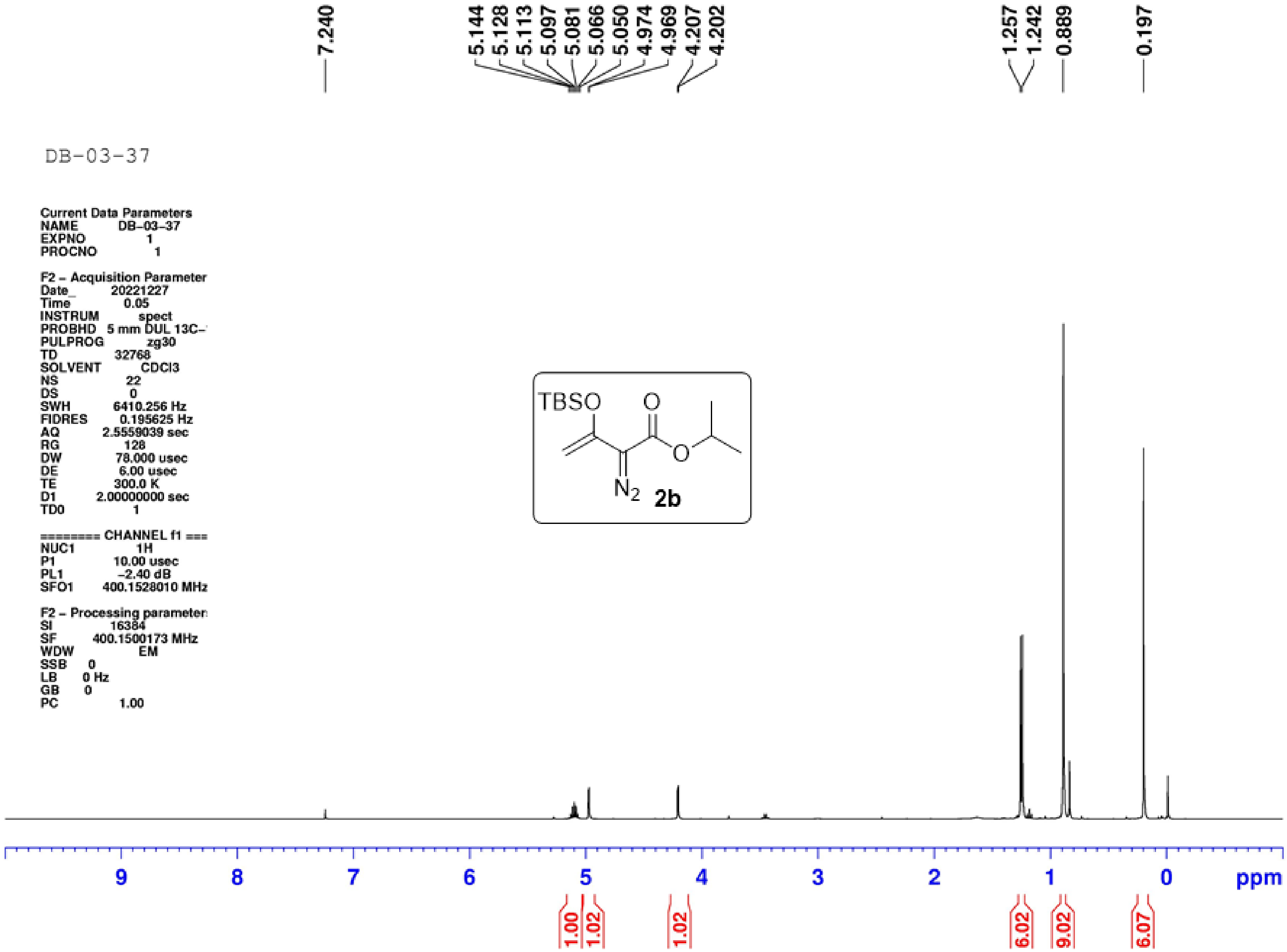

^{13}C NMR ($CDCl_3$, 100 MHz)

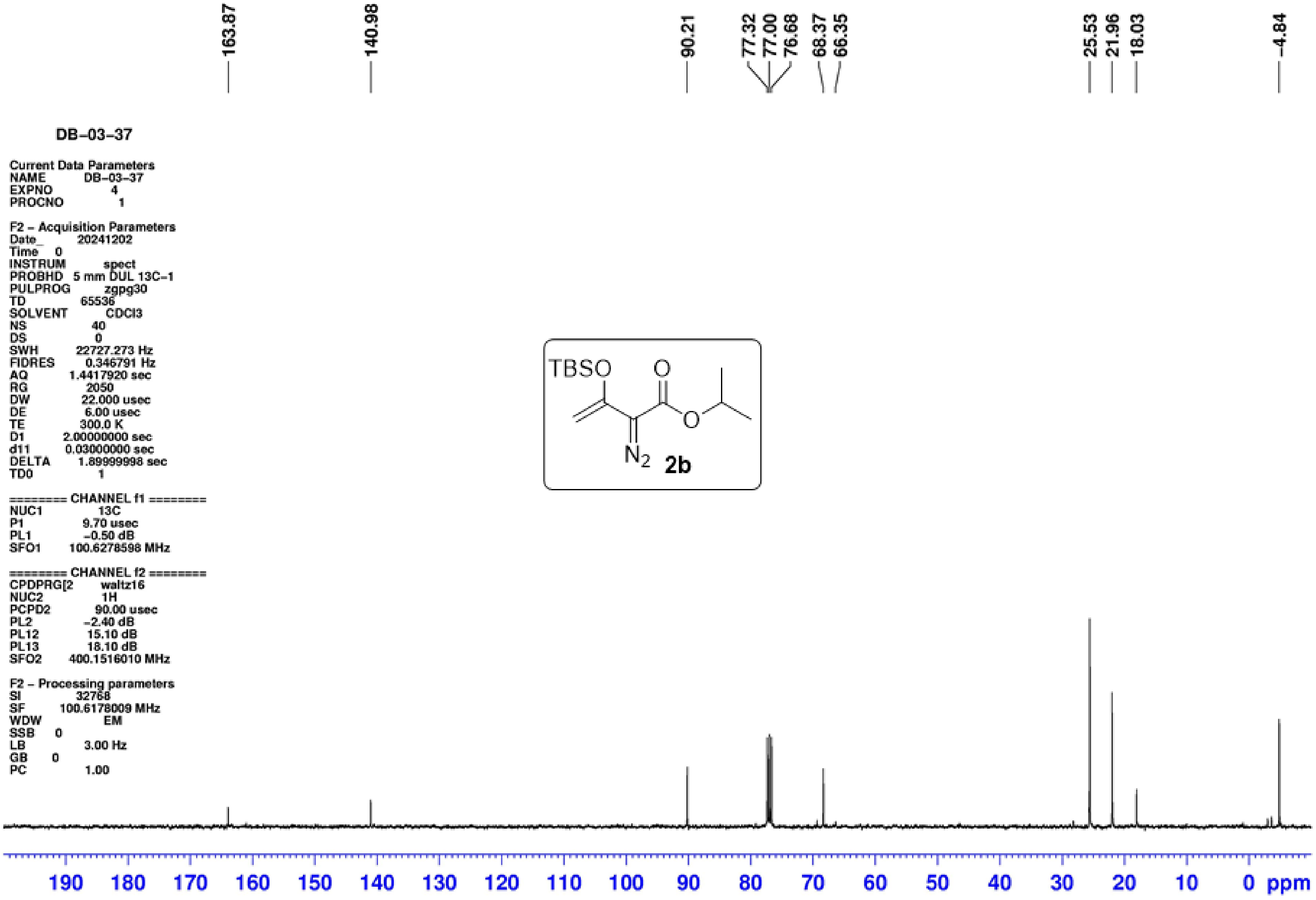

^{1}H NMR ($CDCl_3$, 400 MHz)

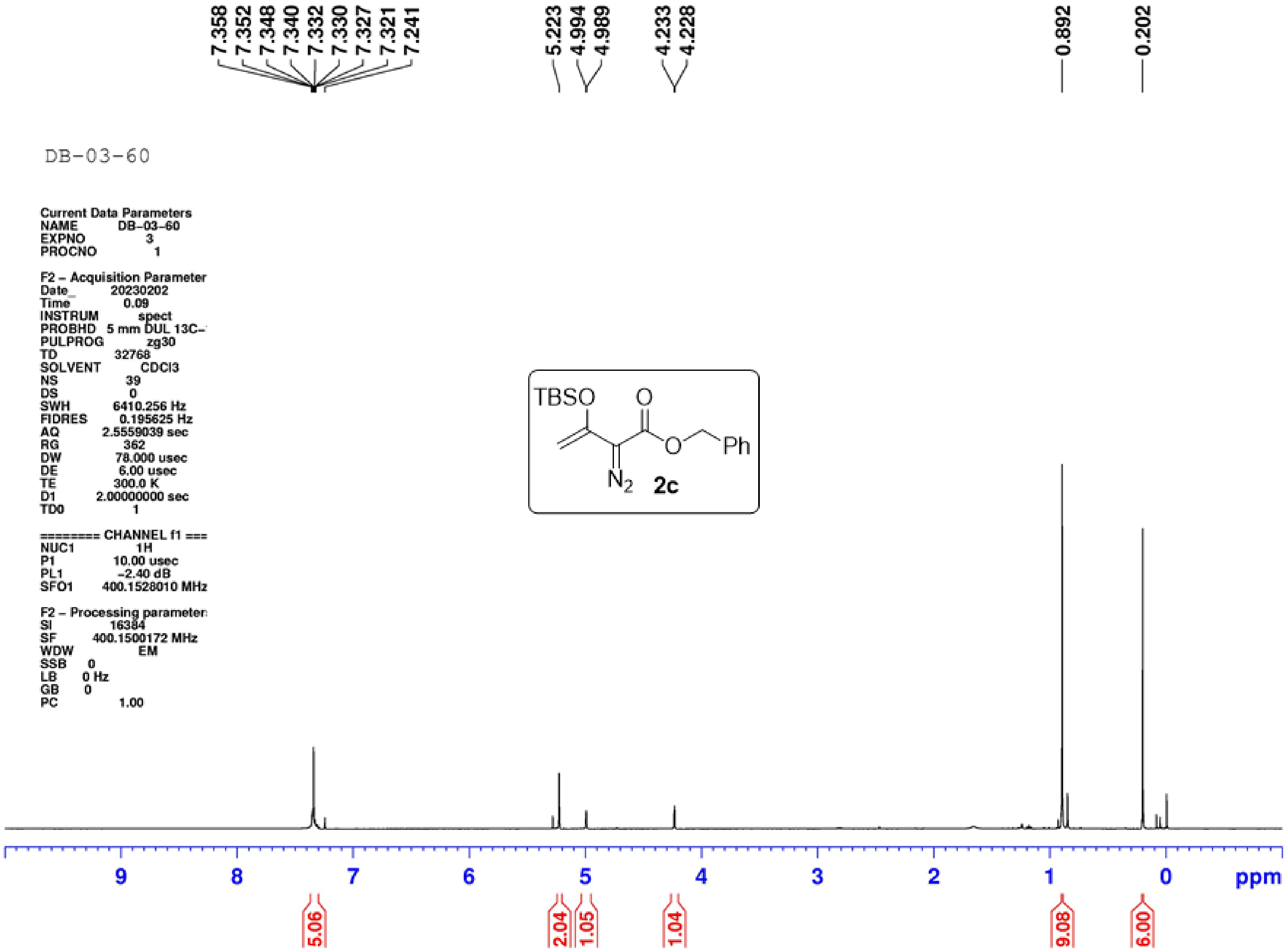

^{13}C NMR ($CDCl_3$, 100 MHz)

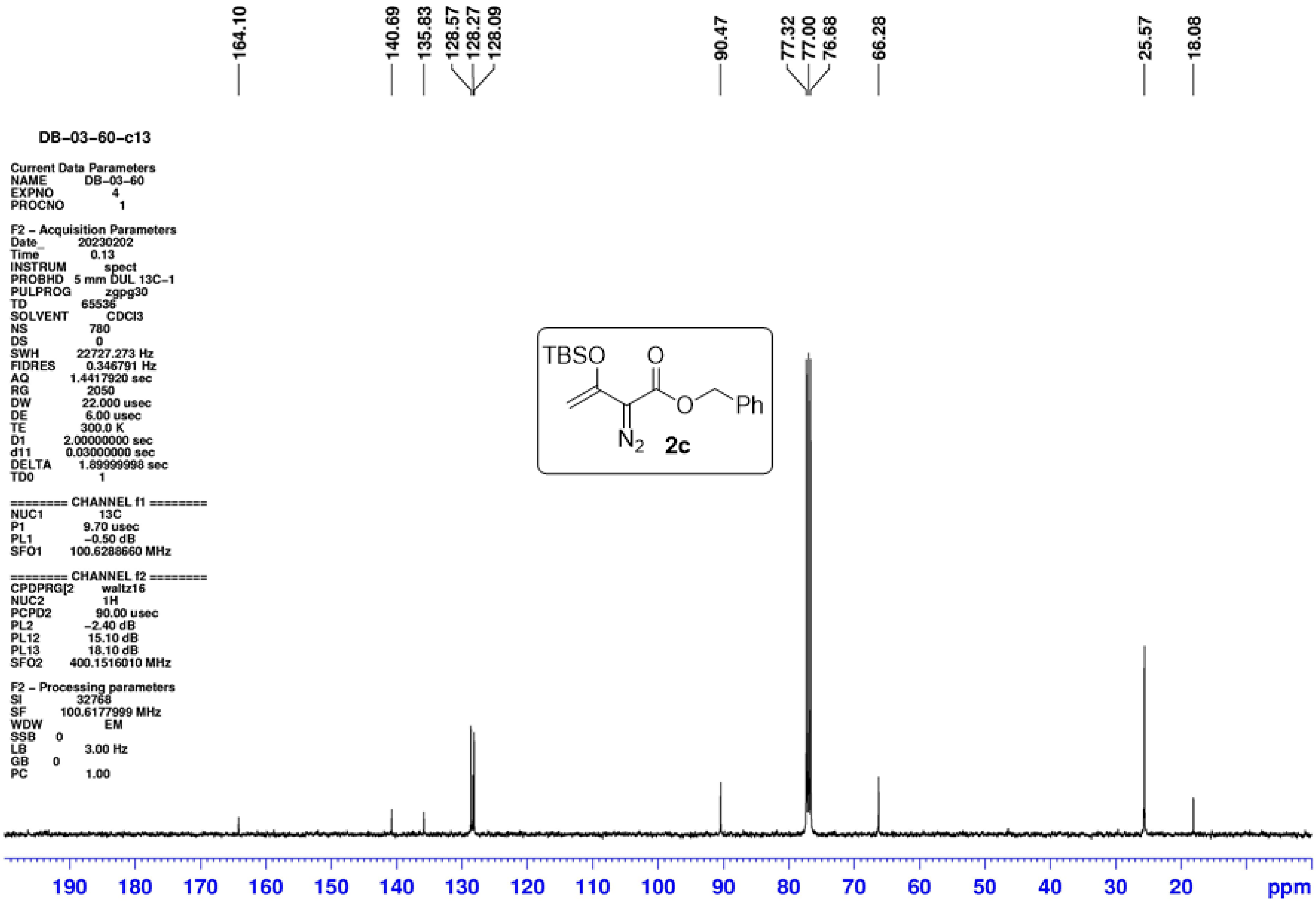

^{1}H NMR ($CDCl_3$, 400 MHz)

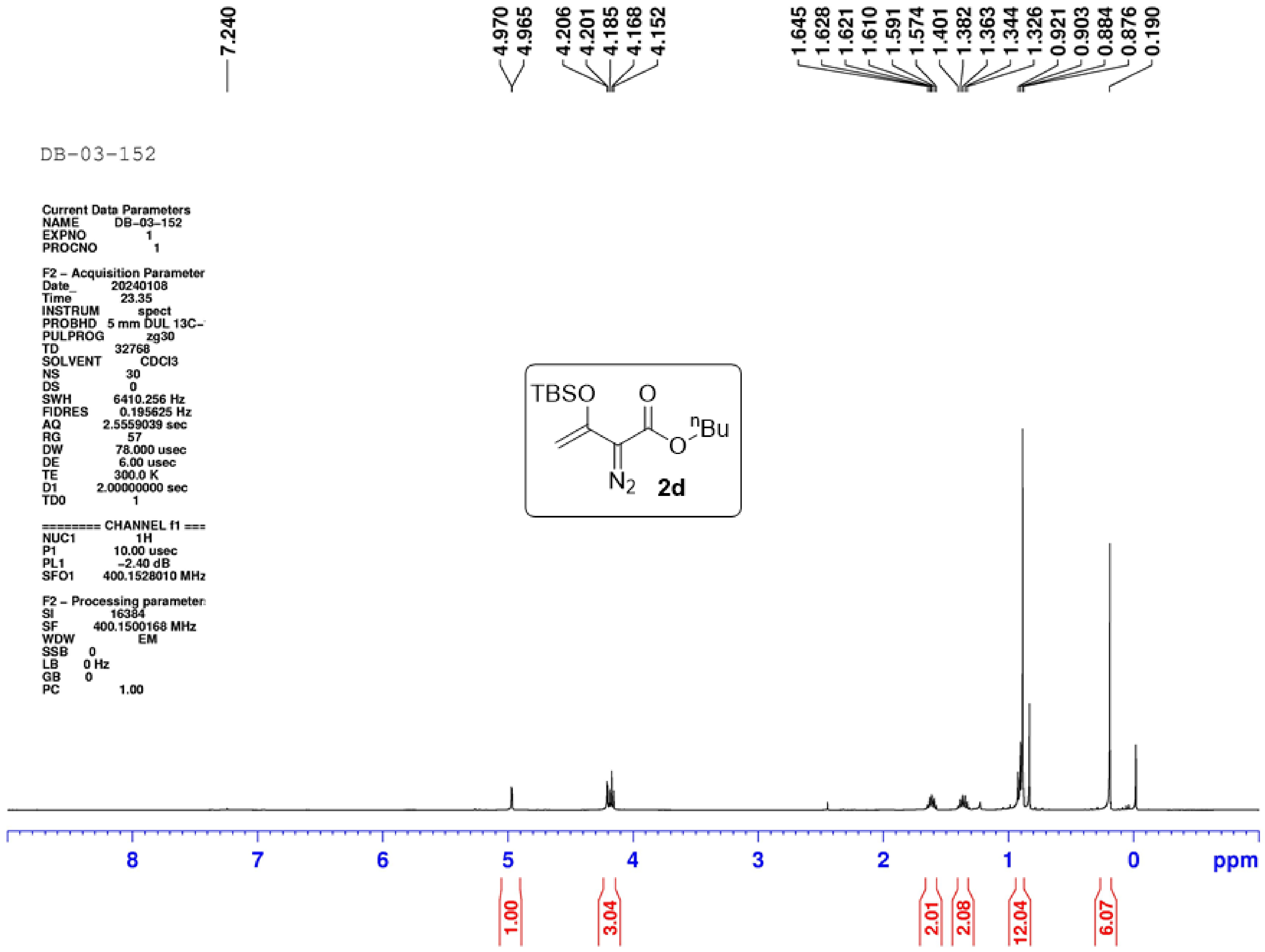

^{13}C NMR ($CDCl_3$, 175 MHz)

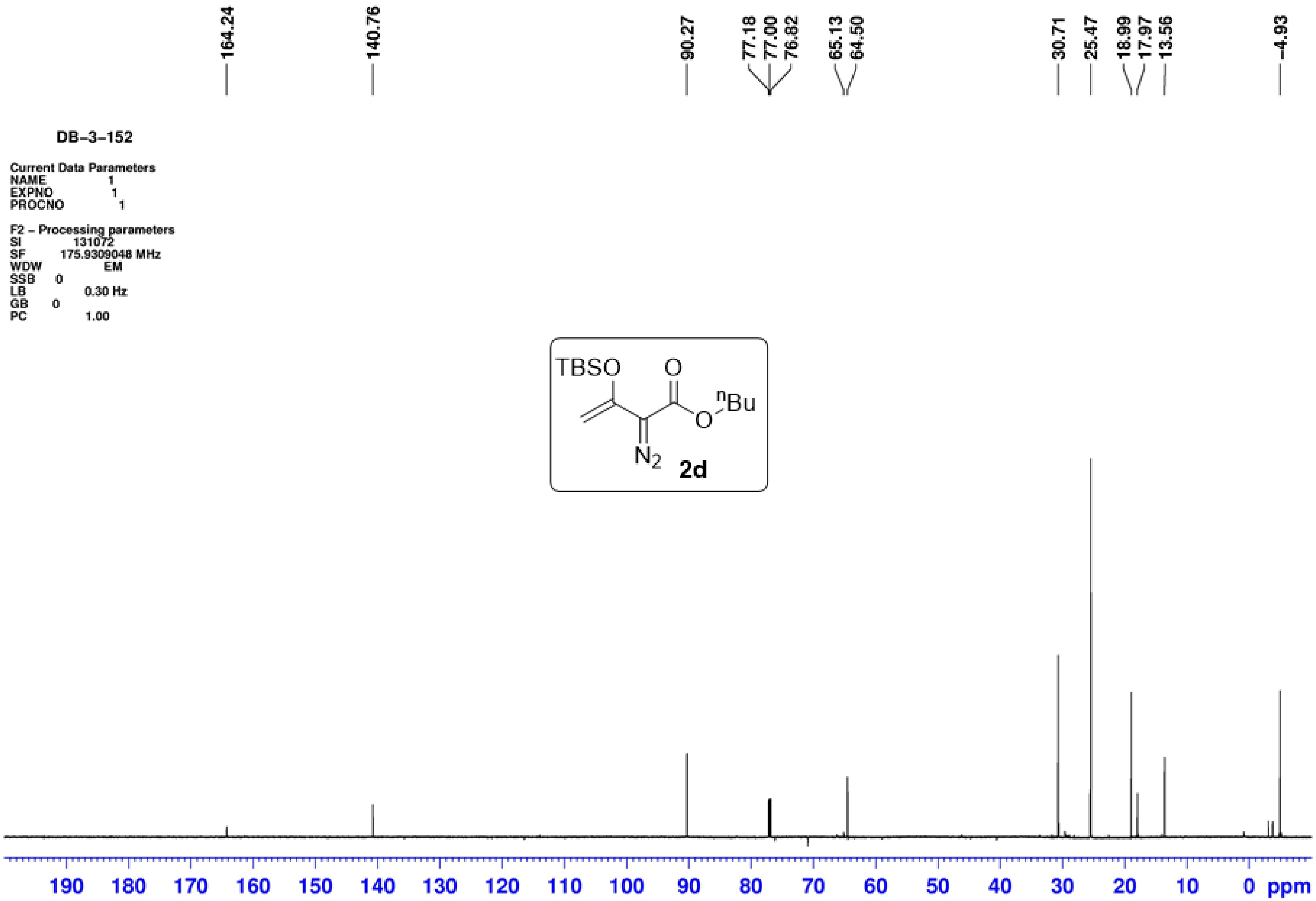

^{1}H NMR ($CDCl_3$, 400 MHz)

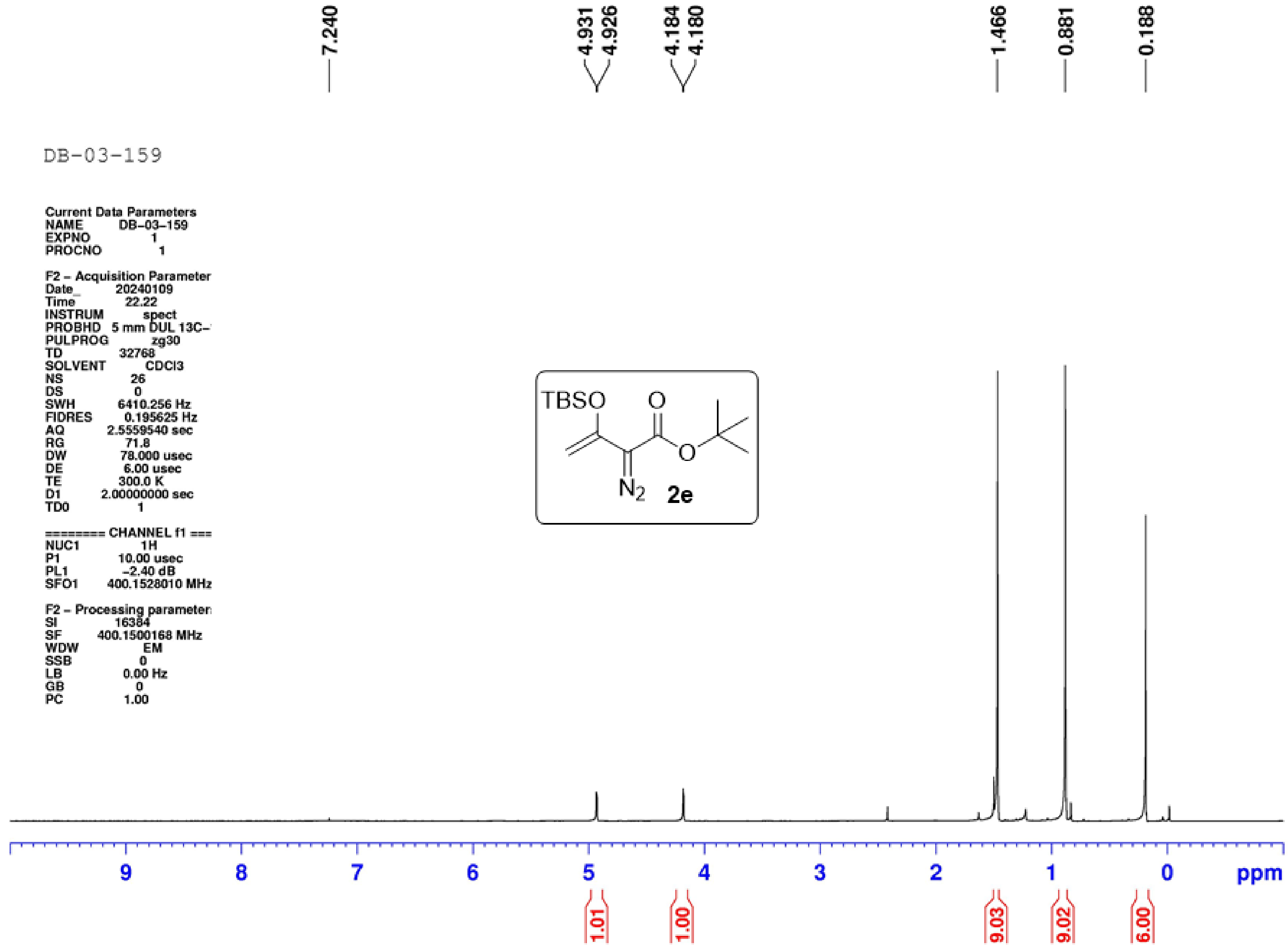

^{13}C NMR (CDCl$_3$, 100 MHz)

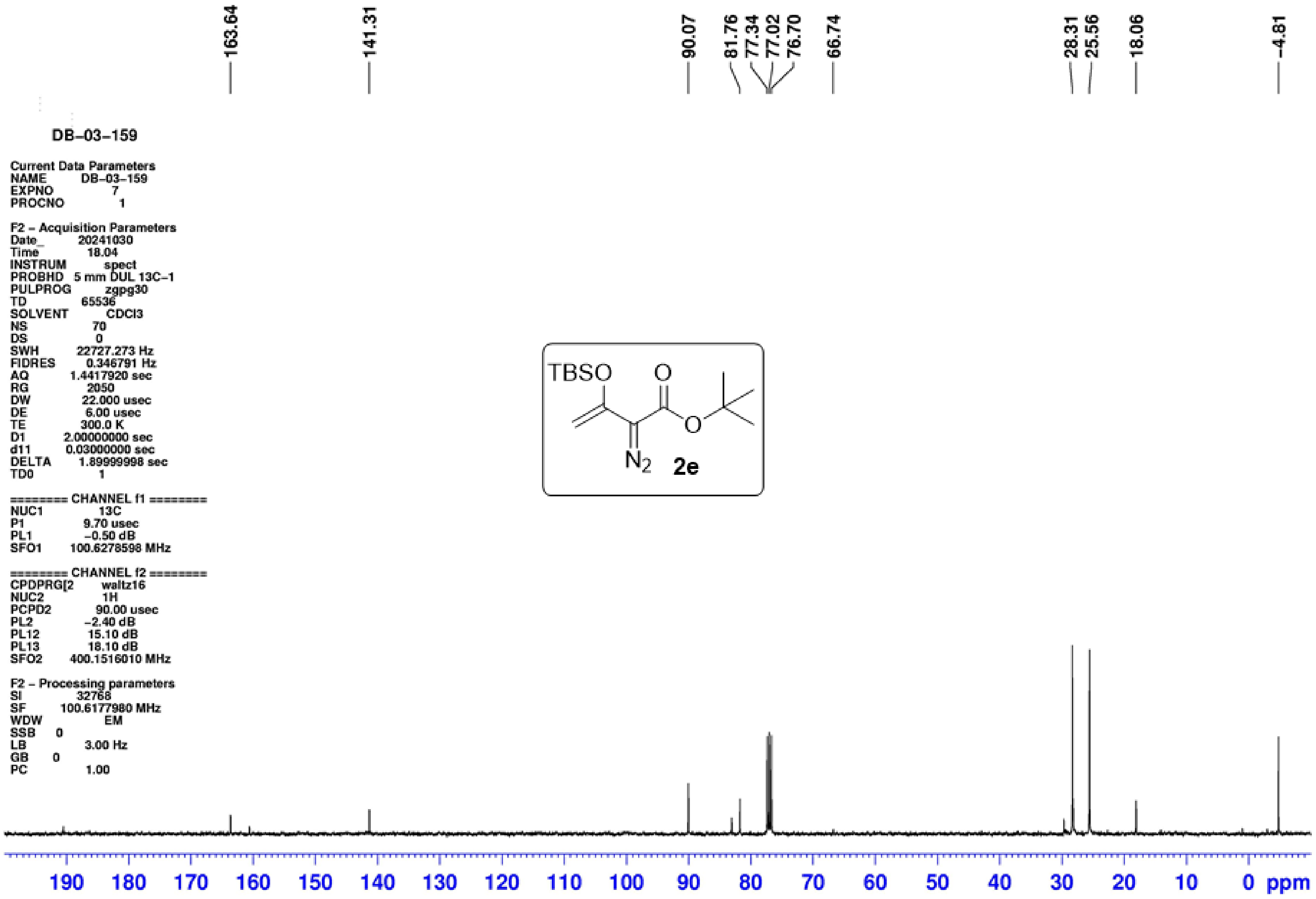

^{1}H NMR ($CDCl_3$, 400 MHz)

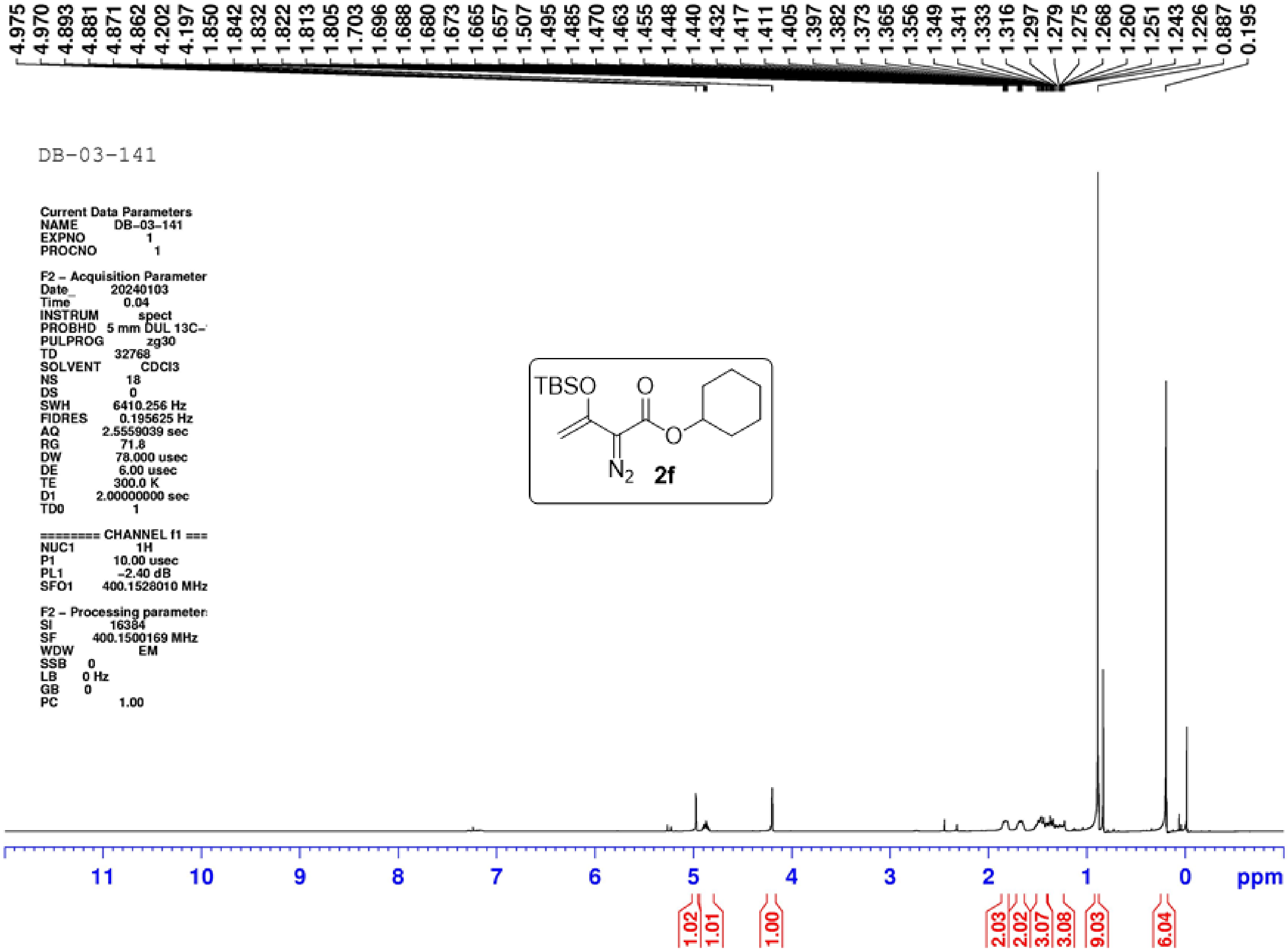

^{13}C NMR ($CDCl_3$, 100 MHz)

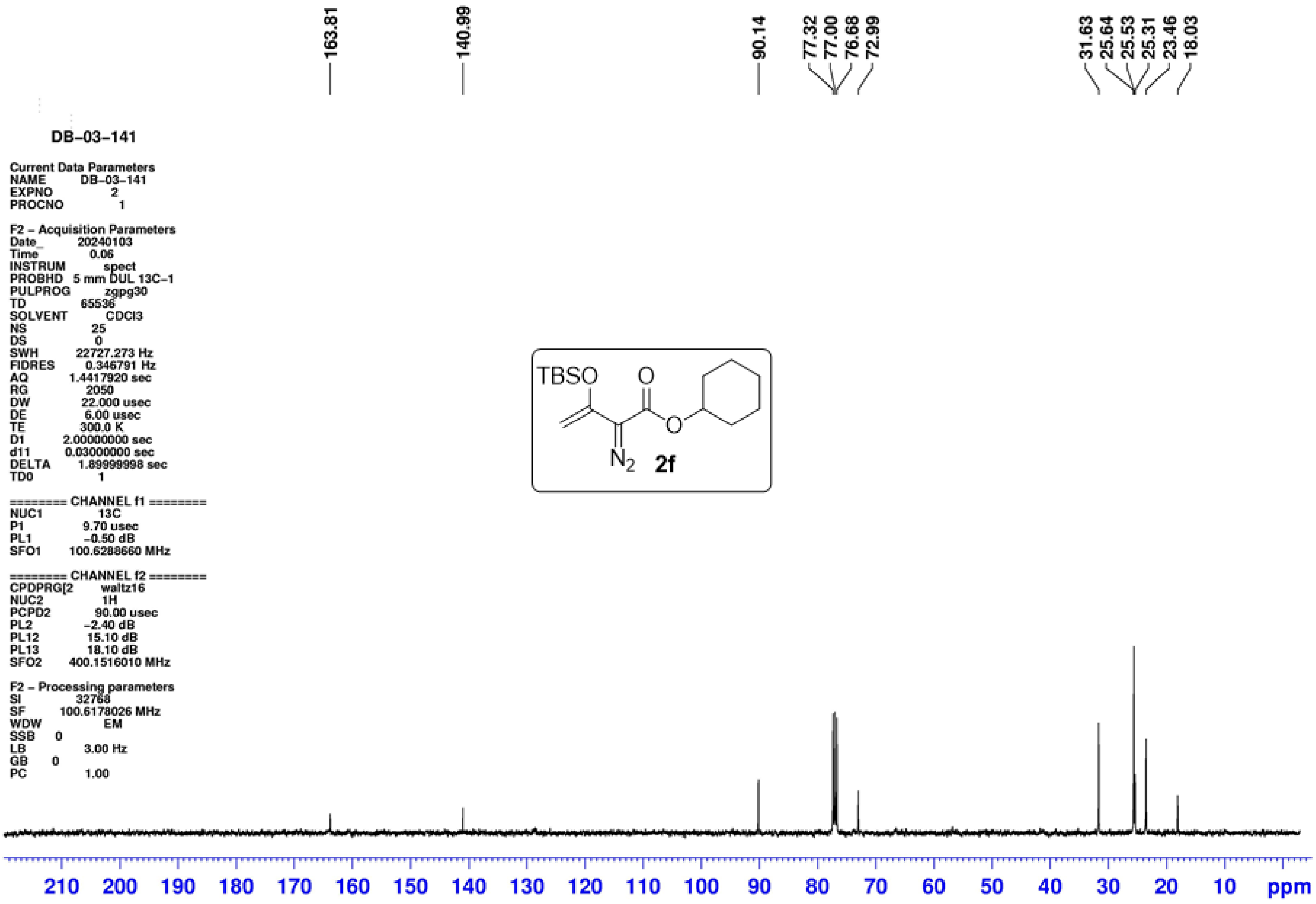

^{1}H NMR ($CDCl_3$, 400 MHz)

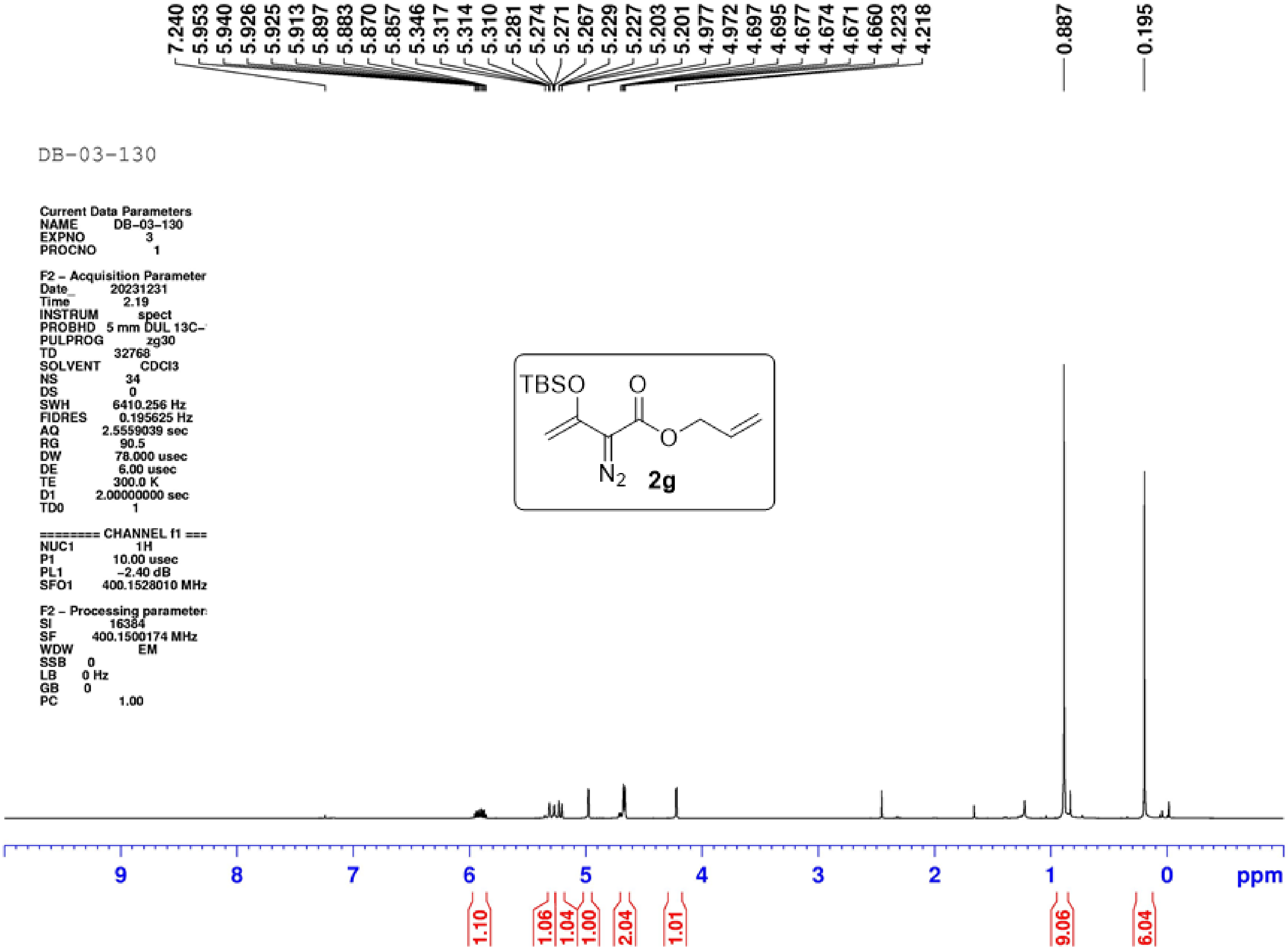

^{13}C NMR ($CDCl_3$, 100 MHz)

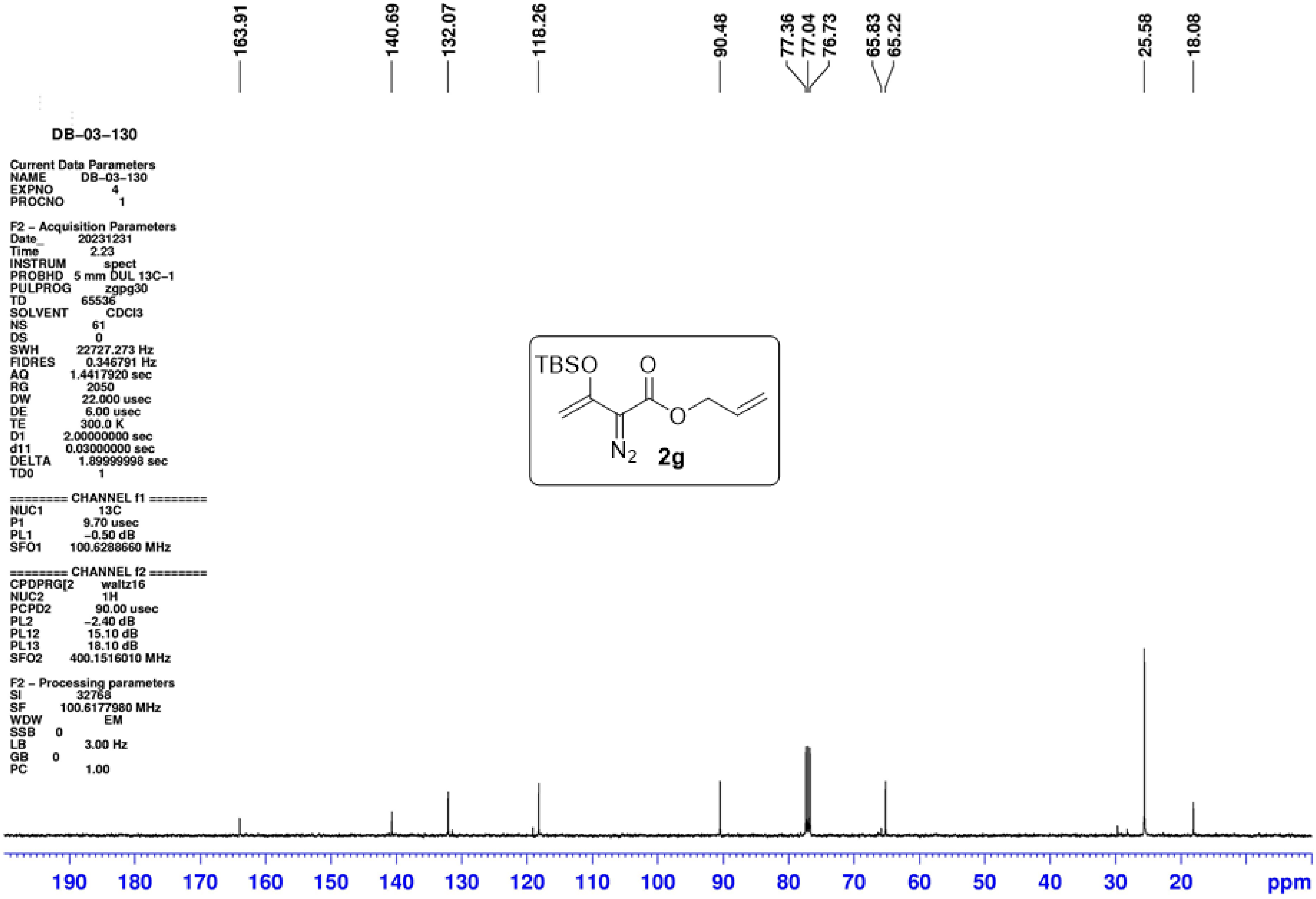

^{1}H NMR ($CDCl_3$, 400 MHz)

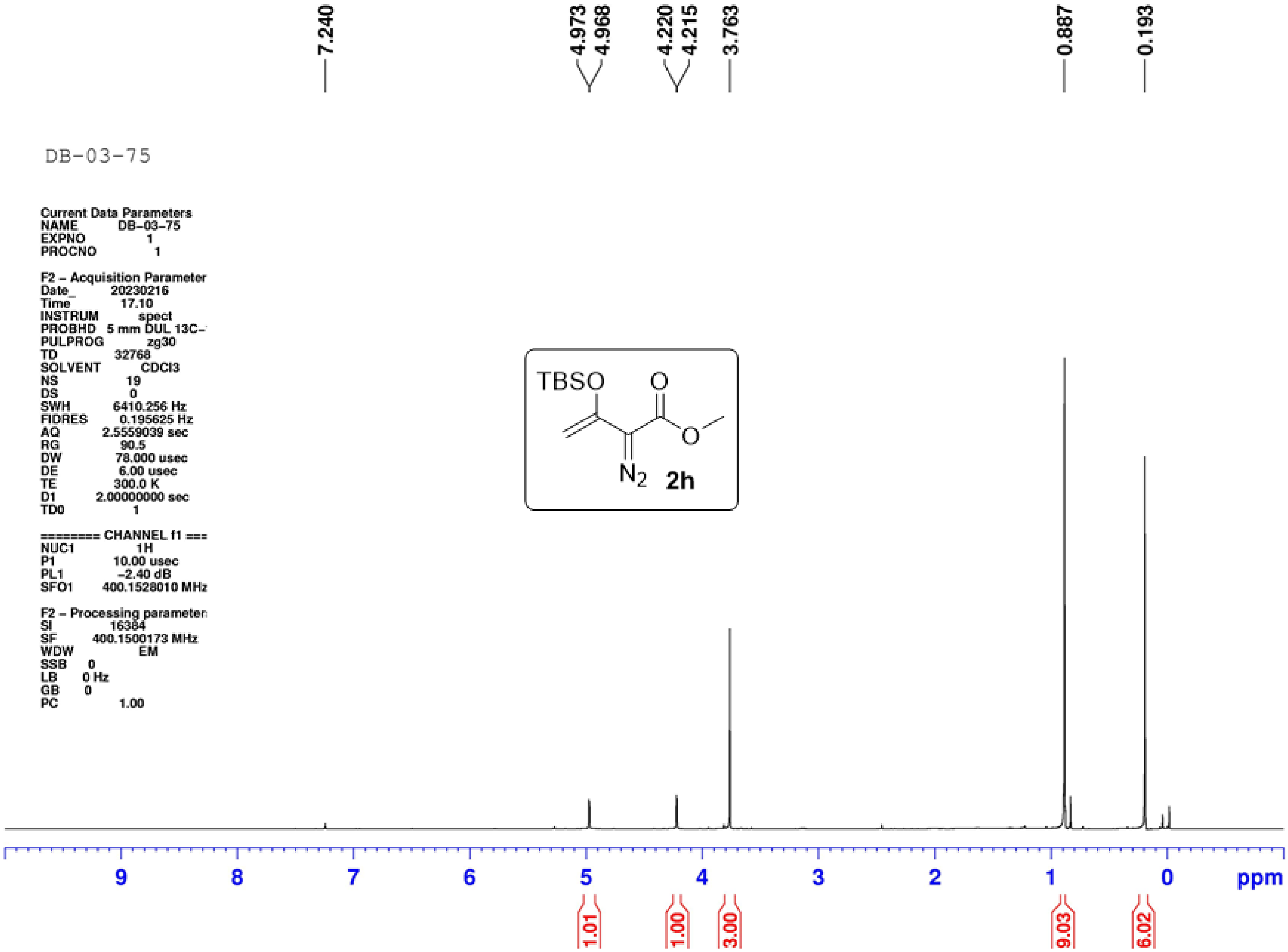

^{13}C NMR ($CDCl_3$, 100 MHz)

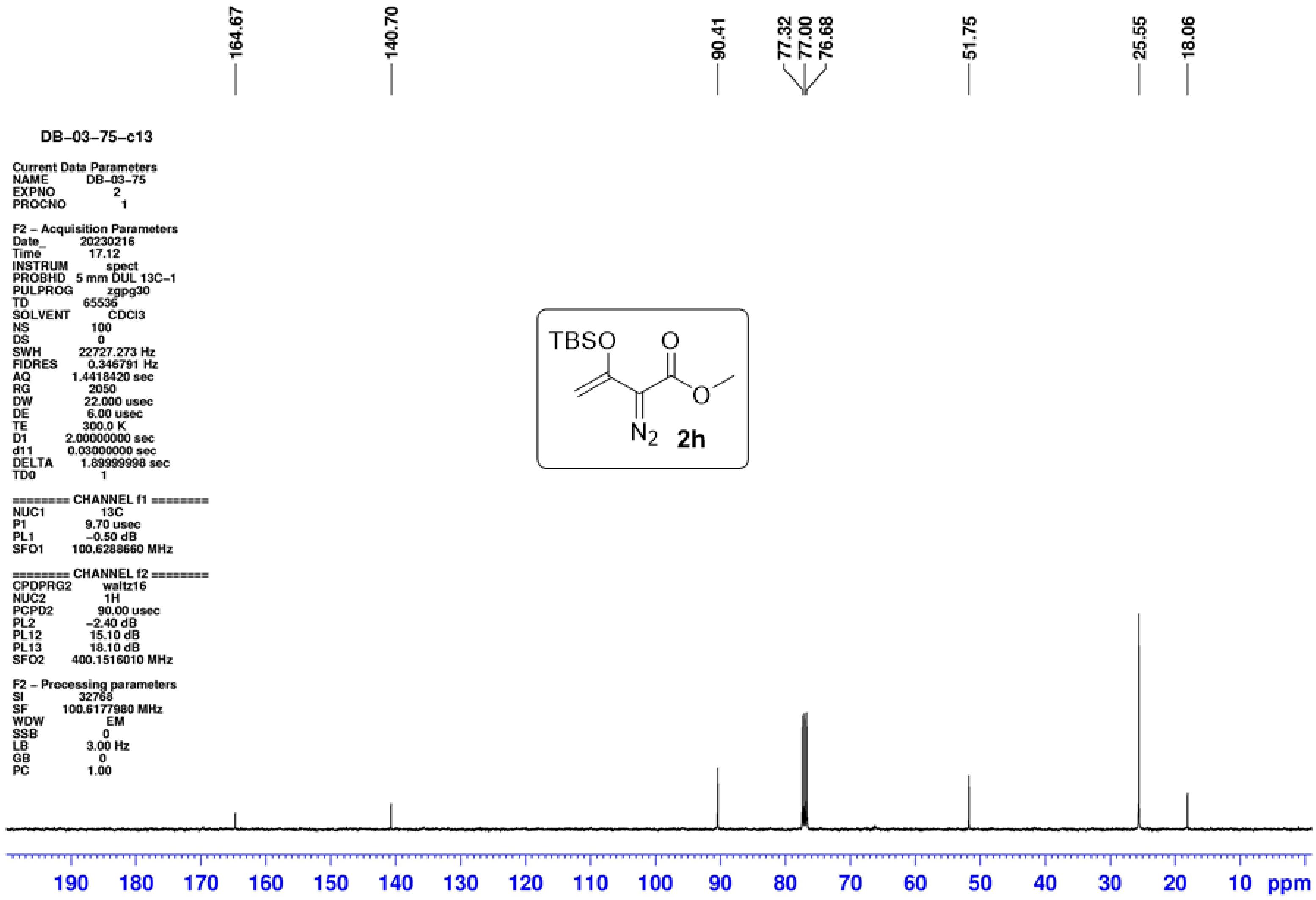

^{1}H NMR ($CDCl_3$, 700 MHz)

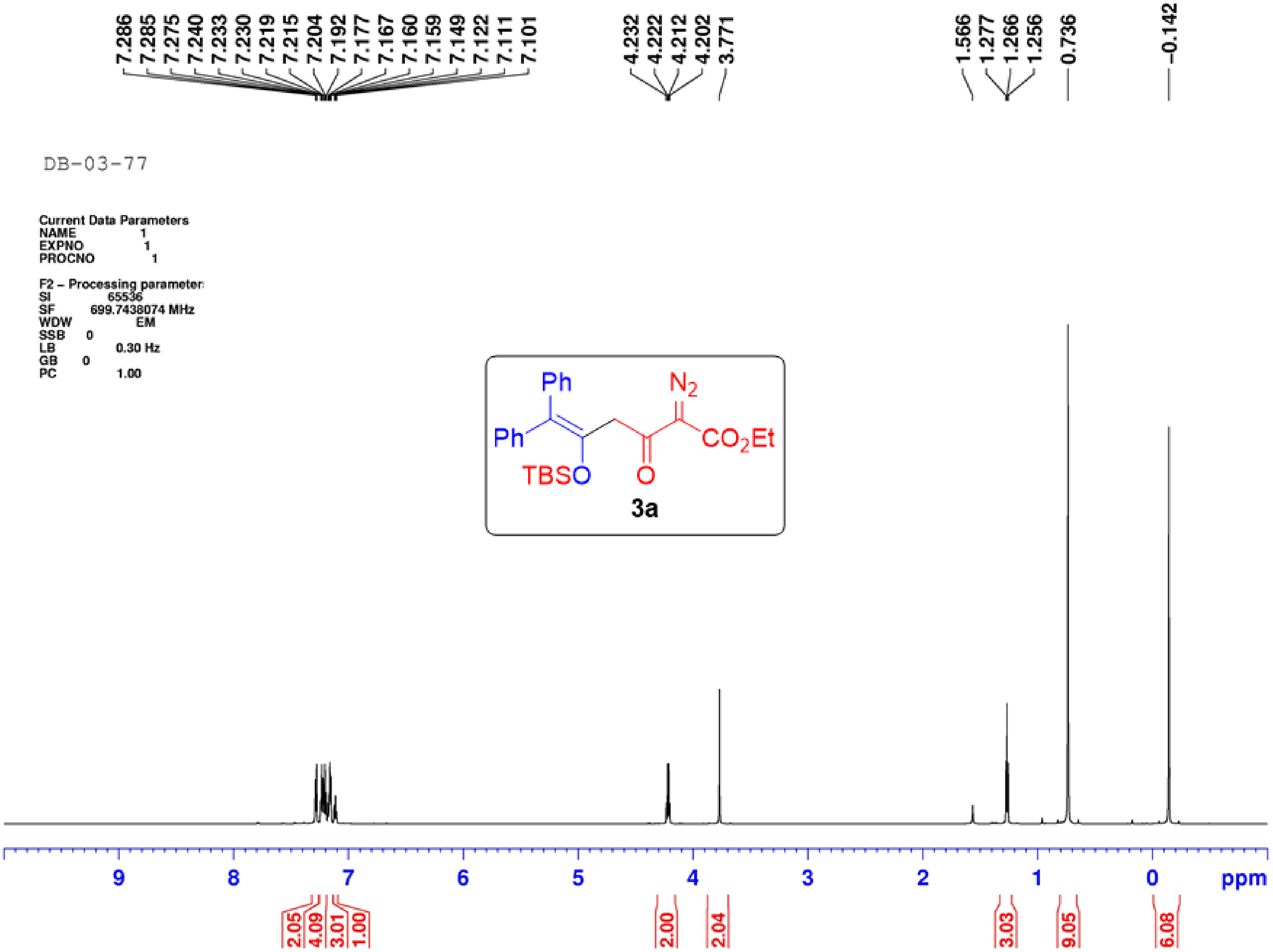

^{13}C NMR ($CDCl_3$, 175 MHz)

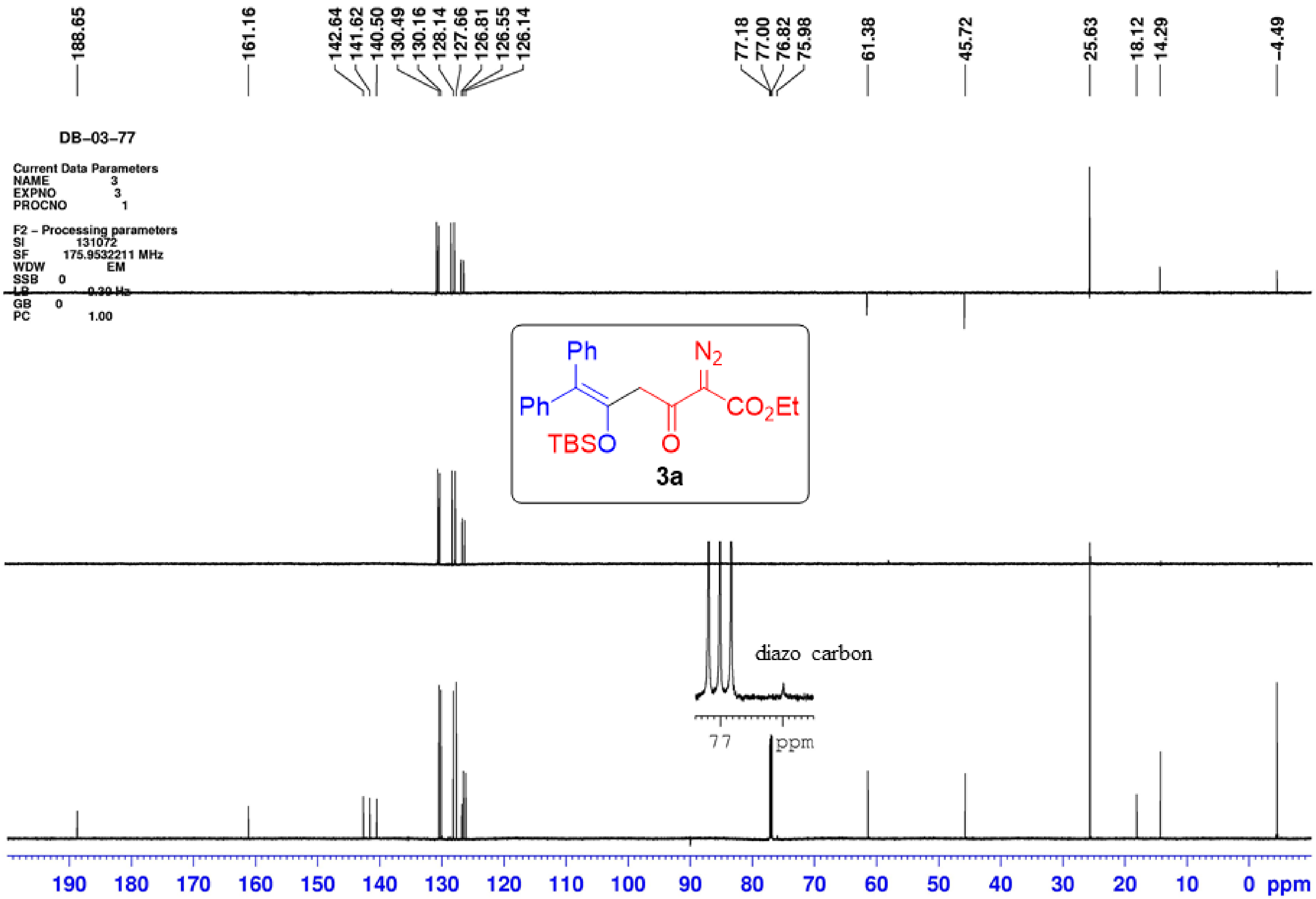

HMBC NMR (700 MHz)

Sample Name:
DB-03-77
Data Collected on:
Varian-NMR-vnmrs700
Archive directory:

Sample directory:

FidFile: DB-03-77-HMBC

Pulse Sequence: gHMBC
Solvent: cdcl3
Data collected on: May 17 2024

Temp. 25.0 C / 298.1 K
Operator: peng

Relax. delay 1.000 sec
Mixing 0.080 sec
Acq. time 0.268 sec
Width 8389.3 Hz
2D Width 47534.2 Hz
8 repetitions
2 x 400 increments
OBSERVE H1, 699.7430936 MHz
DATA PROCESSING
Sq. sine bell 0.075 sec
F1 DATA PROCESSING
Gauss apodization 0.004 sec
FT size 1024 x 2048
Total time 2 hr, 24 min

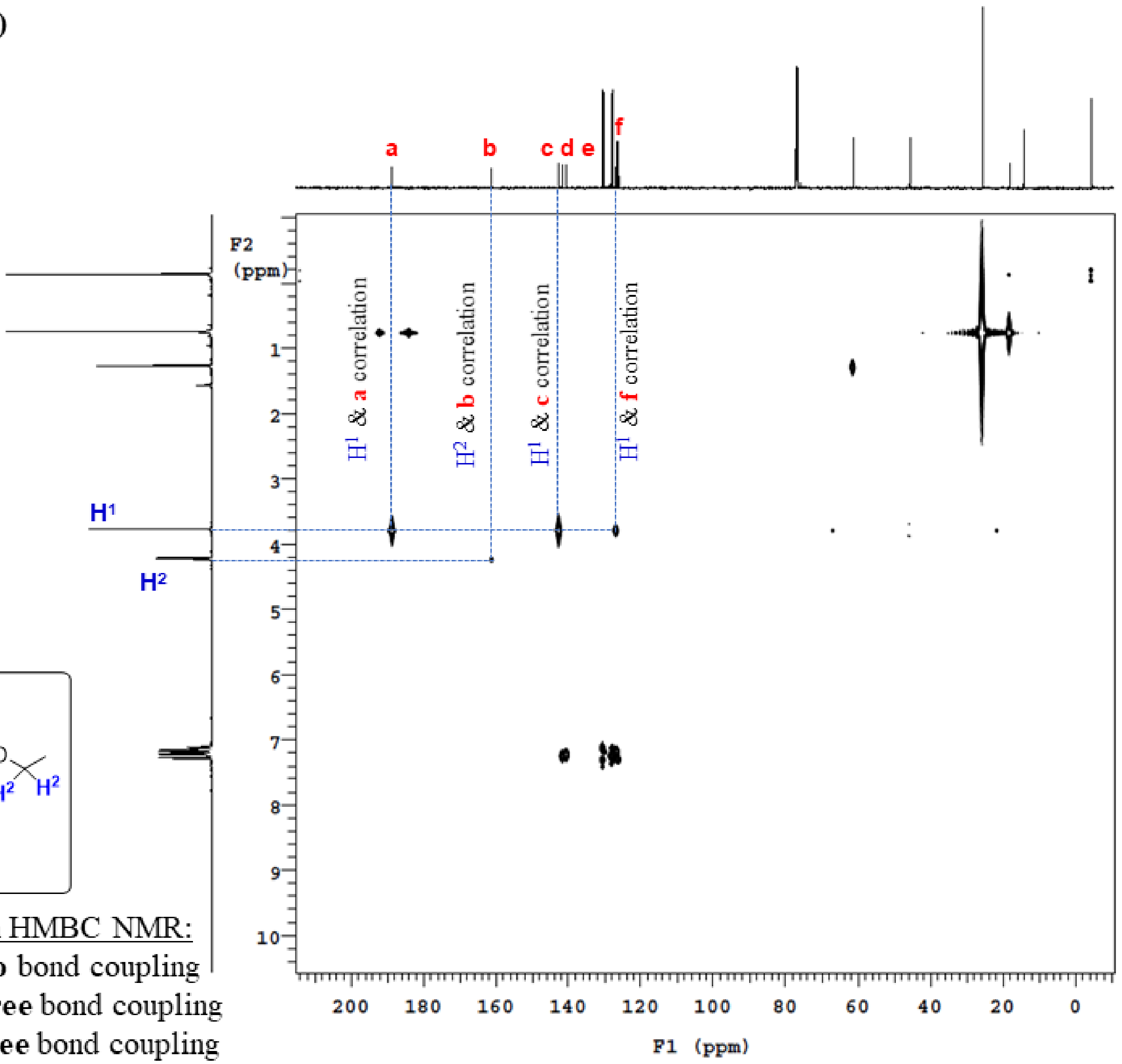

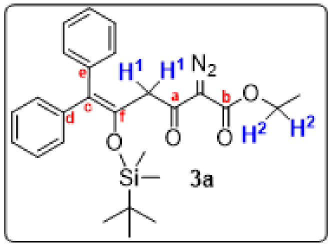

Observed correlation in HMBC NMR:

(1) H^1 and a carbon **two** bond coupling

(2) H^2 and b carbon **three** bond coupling

(3) H^1 and c carbon **three** bond coupling

(4) H^1 and f carbon **two** bond coupling

HSQC NMR (700 MHz)

Sample Name:
DB-03-77
Data Collected on:
Varian-NMR-vnmrs700
Archive directory:

Sample directory:

FidFile: DB-03-77-HSQC

Pulse Sequence: gHSQC
Solvent: cdcl3
Data collected on: May 17 2024

Temp. 25.0 C / 298.1 K
Operator: peng

Relax. delay 1.000 sec
Acq. time 0.286 sec
Width 8389.3 Hz
2D Width 52770.4 Hz
8 repetitions
2 x 400 increments
OBSERVE H1, 699.7431143 MHz
DECOUPLE C13, 175.9663793 MHz
Power 40 dB
on during acquisition
off during delay
W40_Cold modulated
DATA PROCESSING
Gauss apodization 0.069 sec
F1 DATA PROCESSING
Gauss apodization 0.004 sec
FT size 4096 x 4096
Total time 2 hr, 21 min

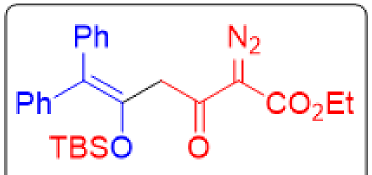

3a

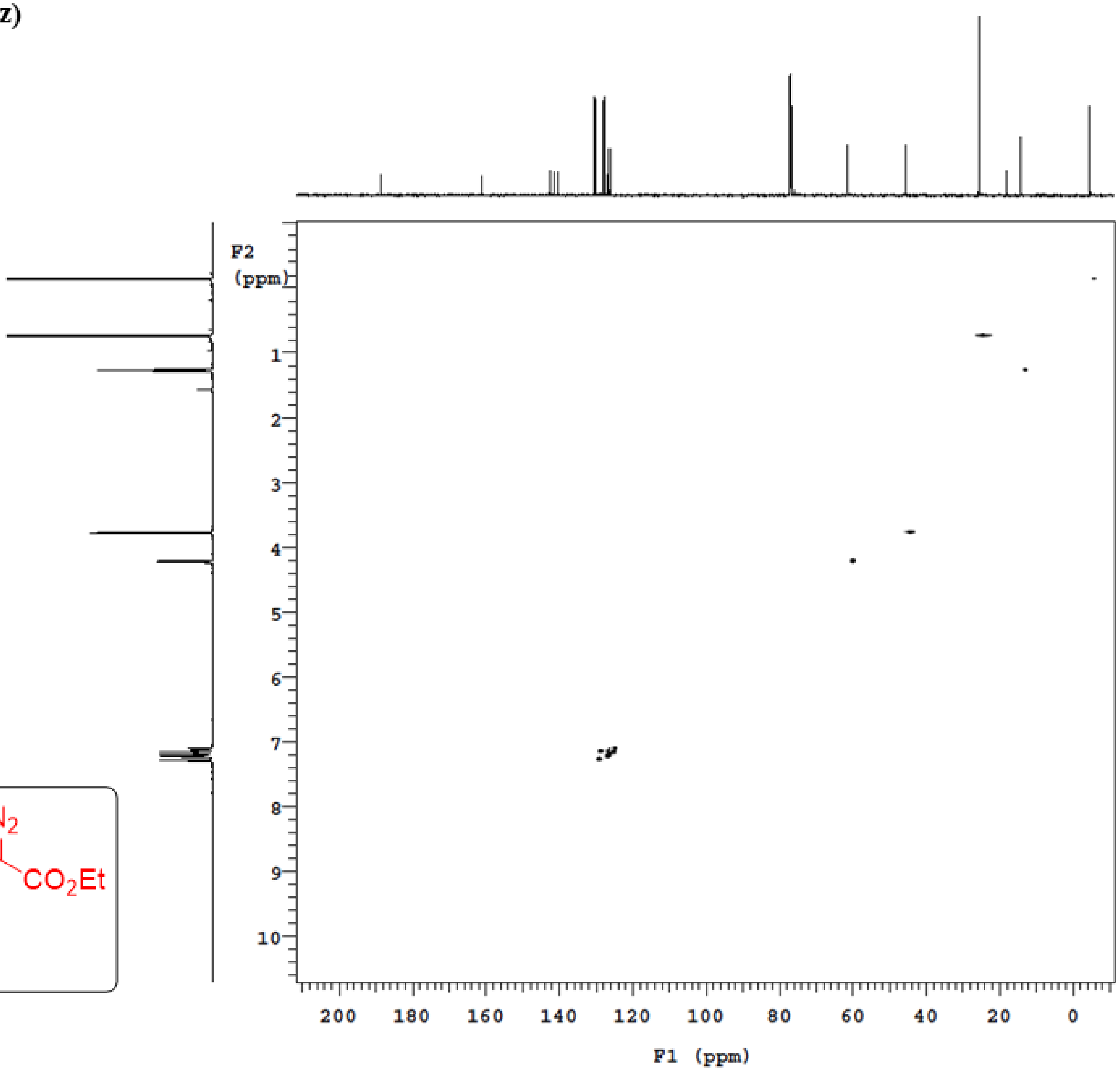

IR Spectral data

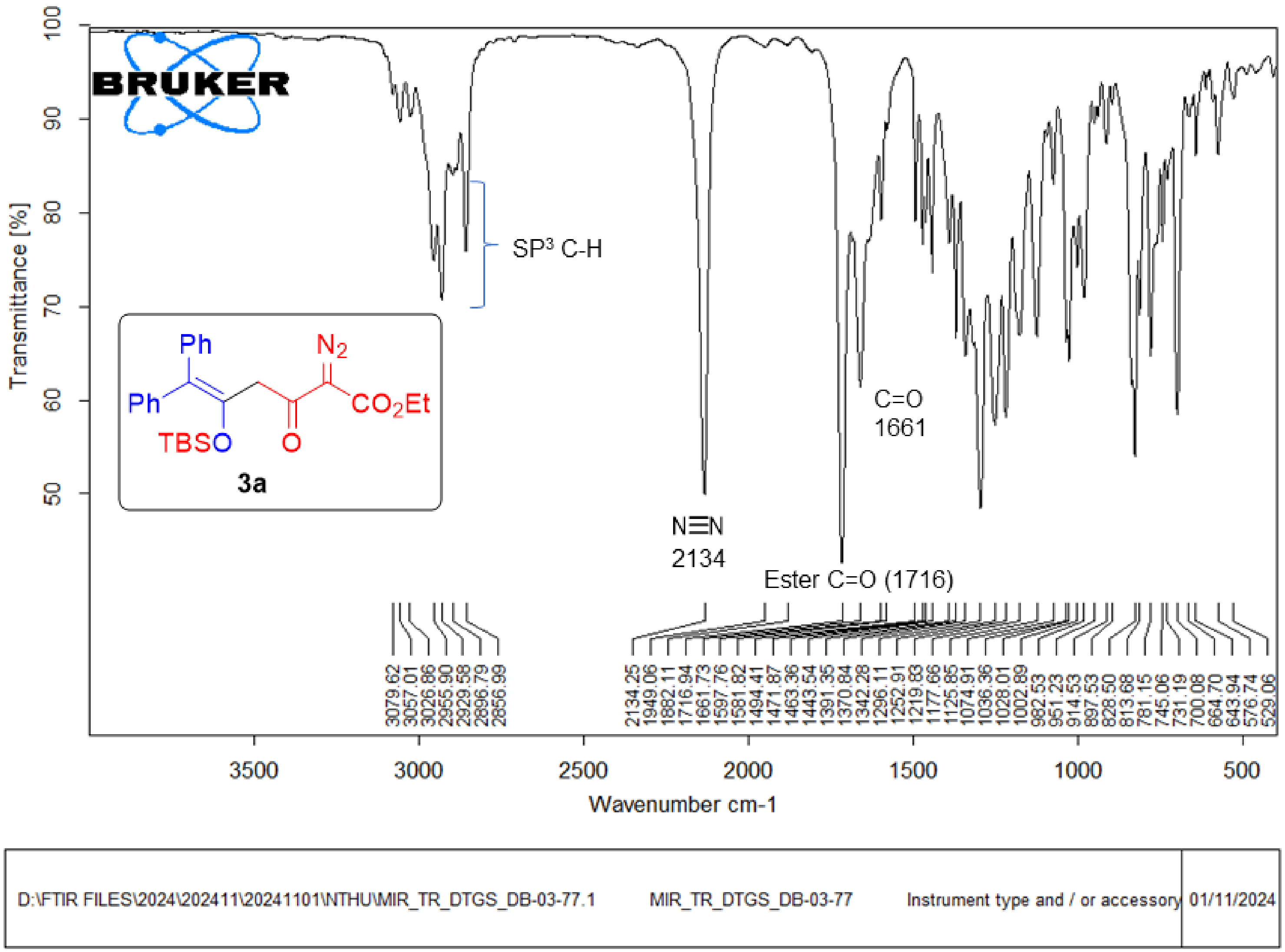

^{1}H NMR ($CDCl_3$, 700 MHz)

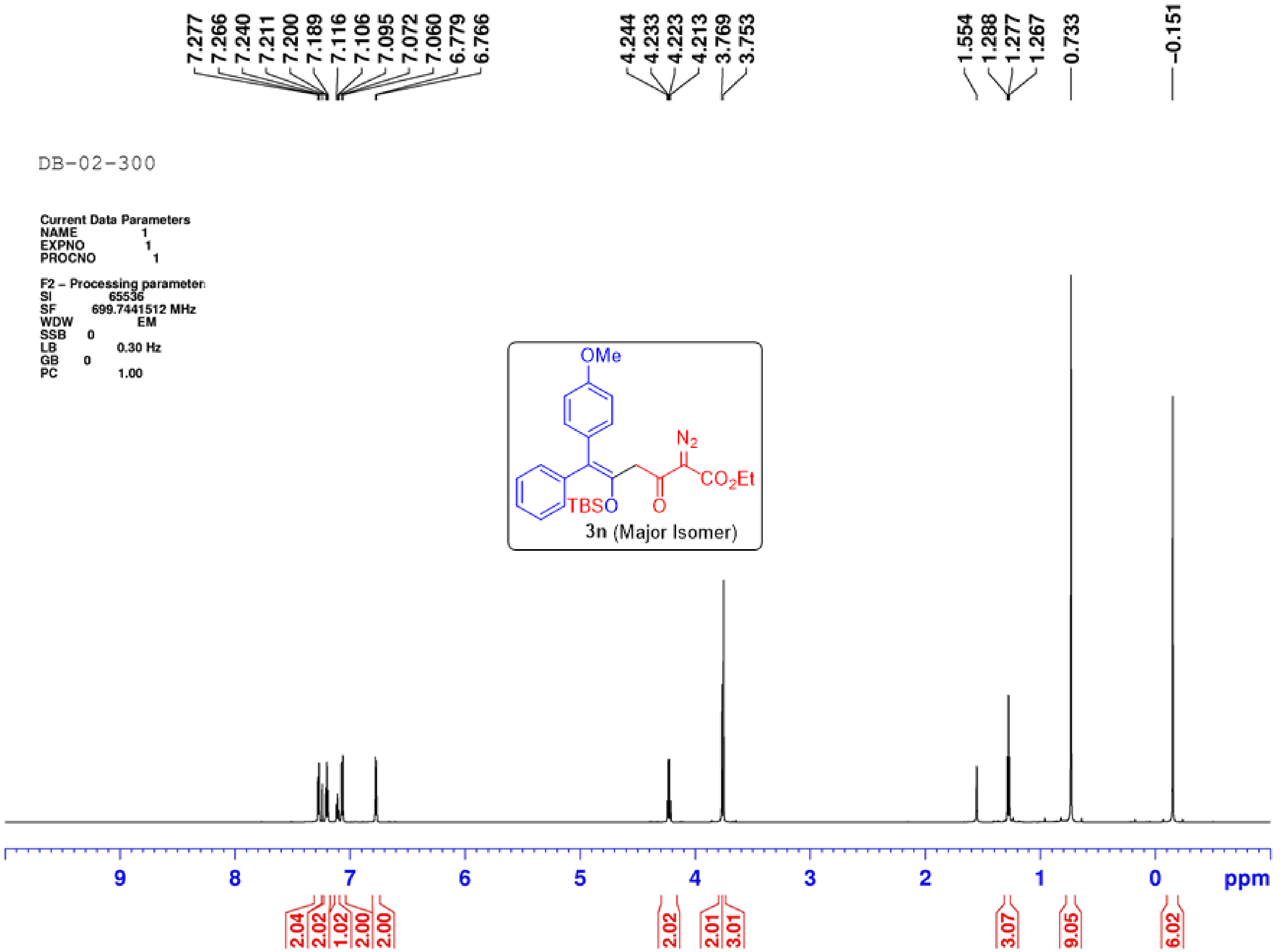

DEPT NMR ($CDCl_3$, 175 MHz)

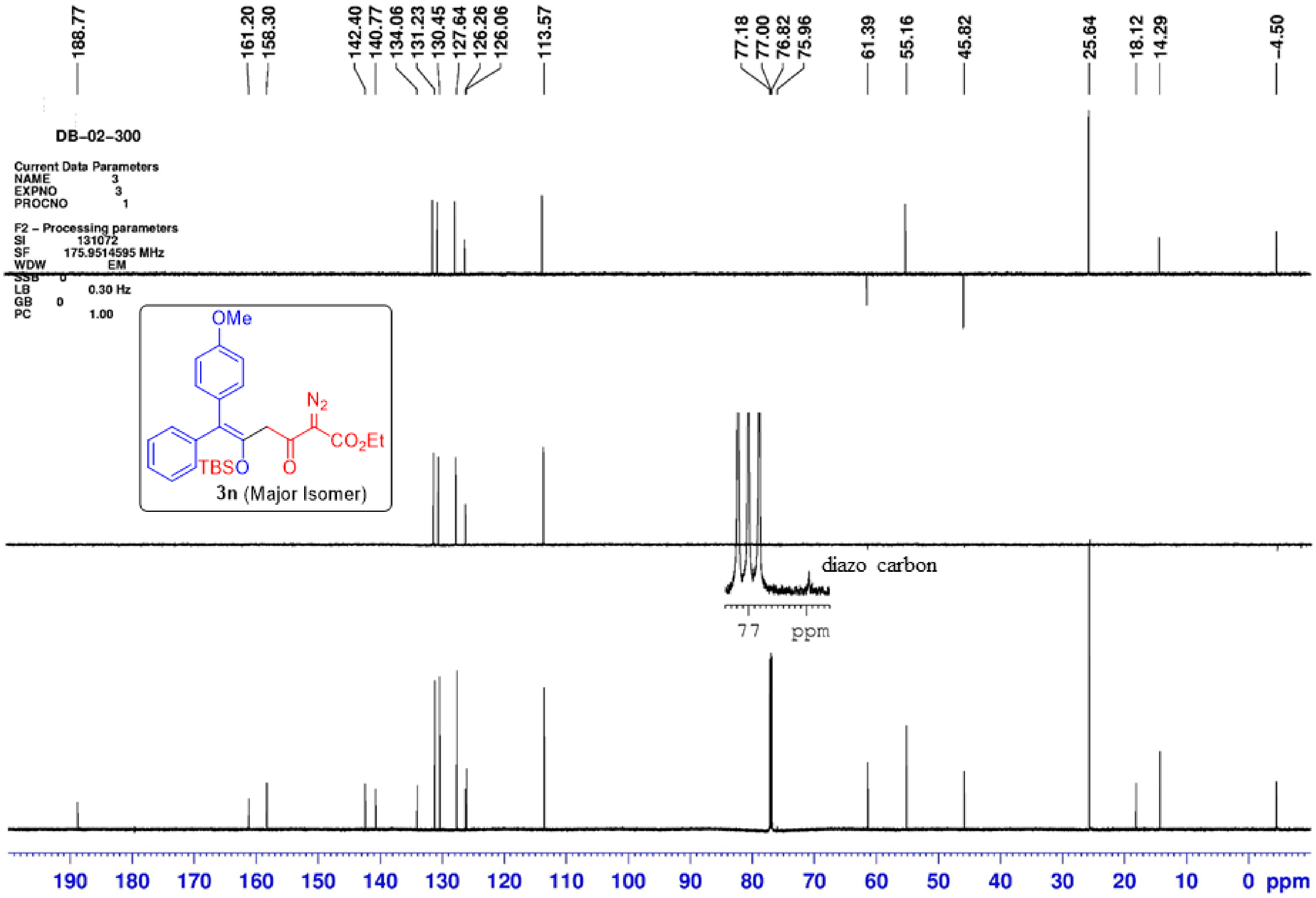

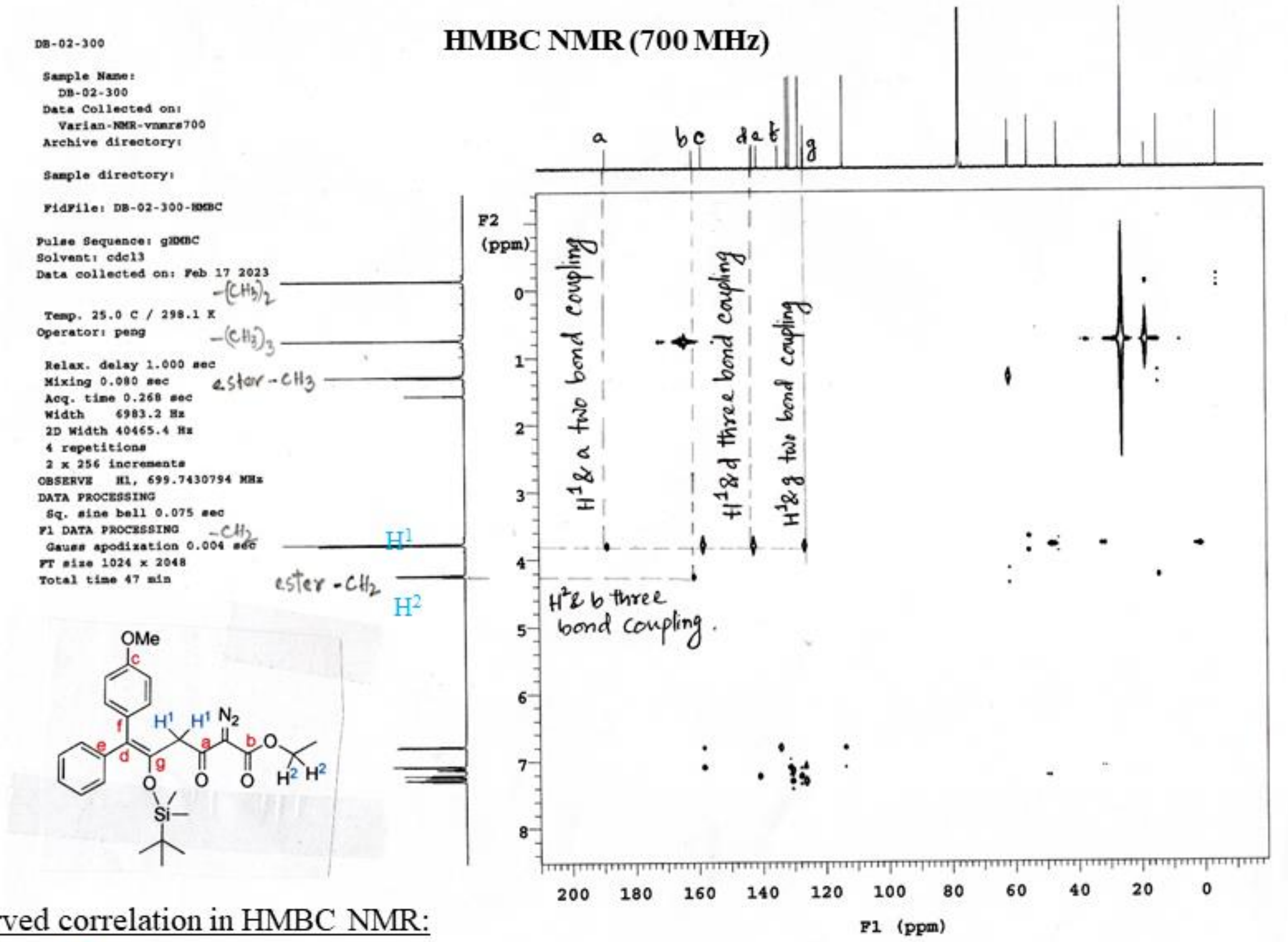

Observed correlation in HMBC NMR:

(1) H^1 and d carbon **3** bond coupling

(2) H^1 and g carbon **2** bond coupling

(3) H^1 and a carbon **2** bond coupling

(4) H^2 and b carbon **3** bond coupling

HSQC NMR (700 MHz)

DB-02-300

Sample Name:
DB-02-300
Data Collected on:
Varian-NMR-vnmrs700
Archive directory:
/home/peng/vnmrsys/data
Sample directory:
DB-02-300
FidFile: gHSQC_01

Pulse Sequence: gHSQC
Solvent: cdcl3
Data collected on: Feb 22 2023

Temp. 25.0 C / 298.1 K
Operator: peng

Relax. delay 1.000 sec
Acq. time 0.286 sec
Width 8389.3 Hz
2D Width 45766.6 Hz
4 repetitions
2 x 256 increments
OBSERVE H1, 699.7430776 MHz
DECOUPLE C13, 175.9637374 MHz
Power 40 dB
on during acquisition
off during delay
W40_Cold modulated
DATA PROCESSING
Gauss apodization 0.069 sec
F1 DATA PROCESSING
Gauss apodization 0.003 sec
FT size 4096 x 4096
Total time 45 min

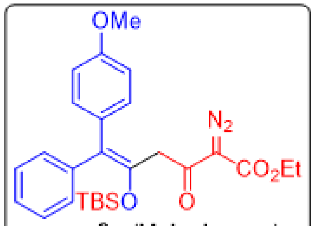

3n (Major Isomer)

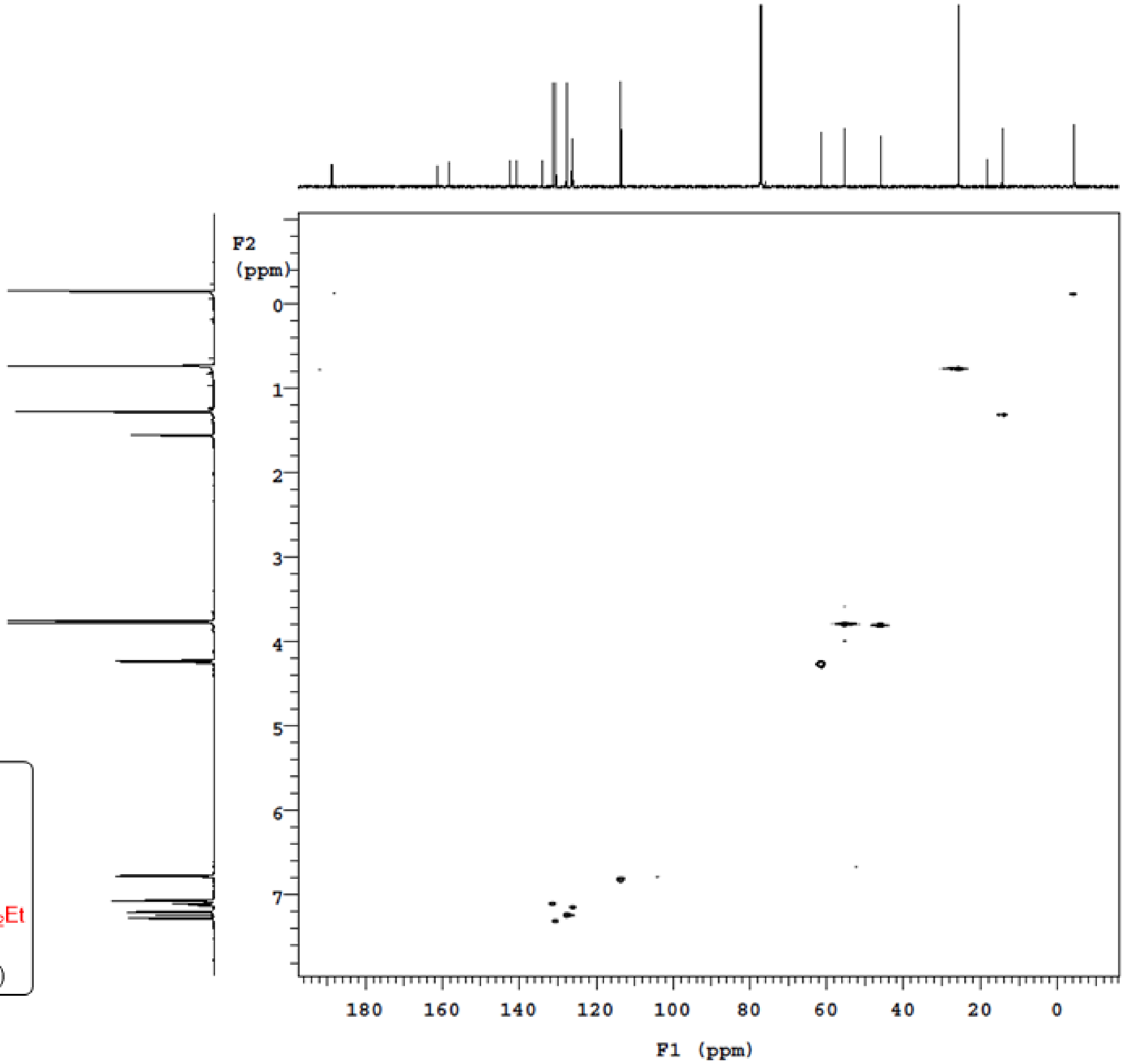

IR Spectral data

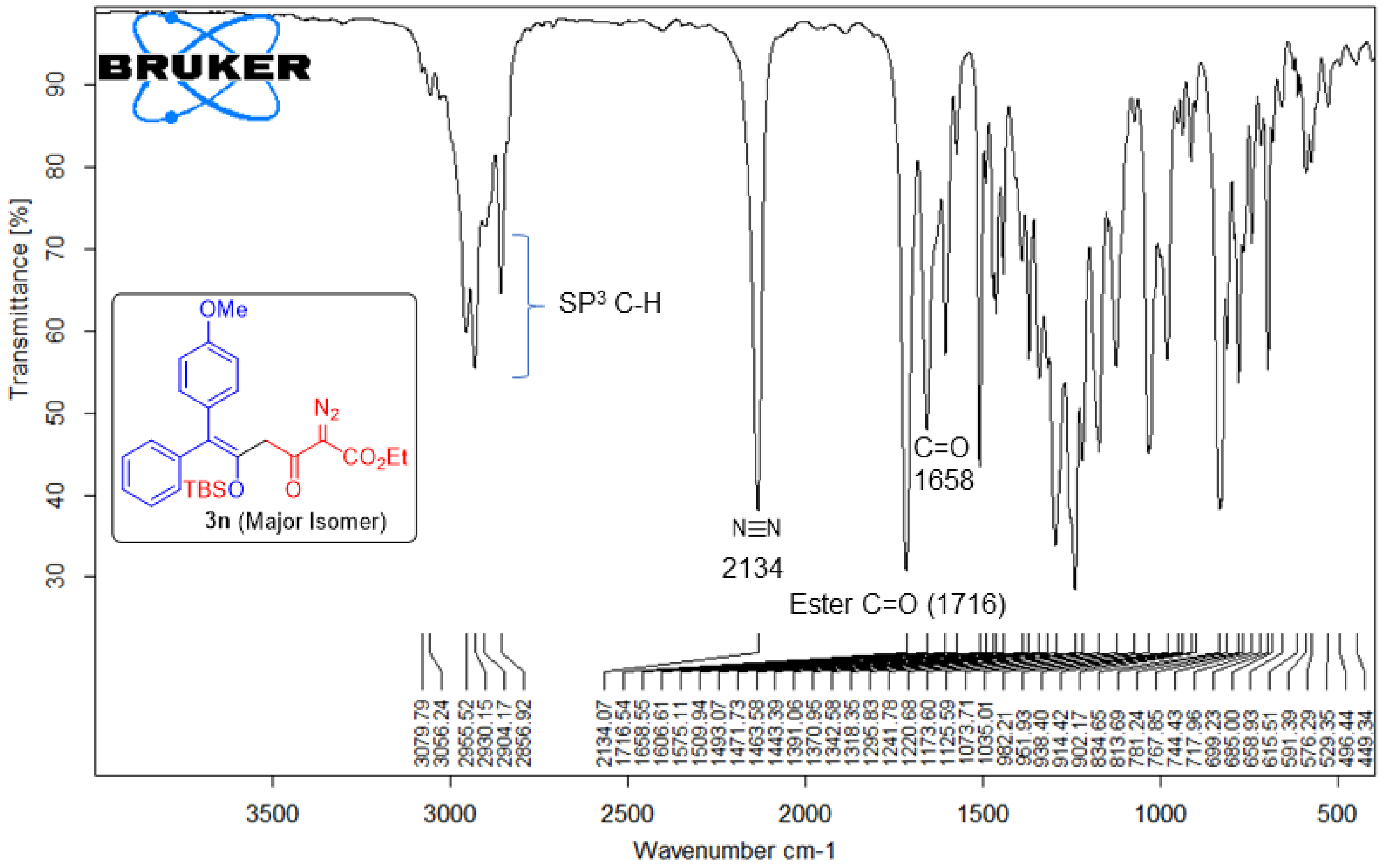

D:\FTIR FILES\2023\202302\20230221\NTHU\MIR_TR_DTGS_DB-O2-300 in DCM then air dry.0	MIR_TR_DTGS_DB-O2-300 in DCM then air d	21/02/2023

Page 1/1

^{1}H NOE (700 MHz)

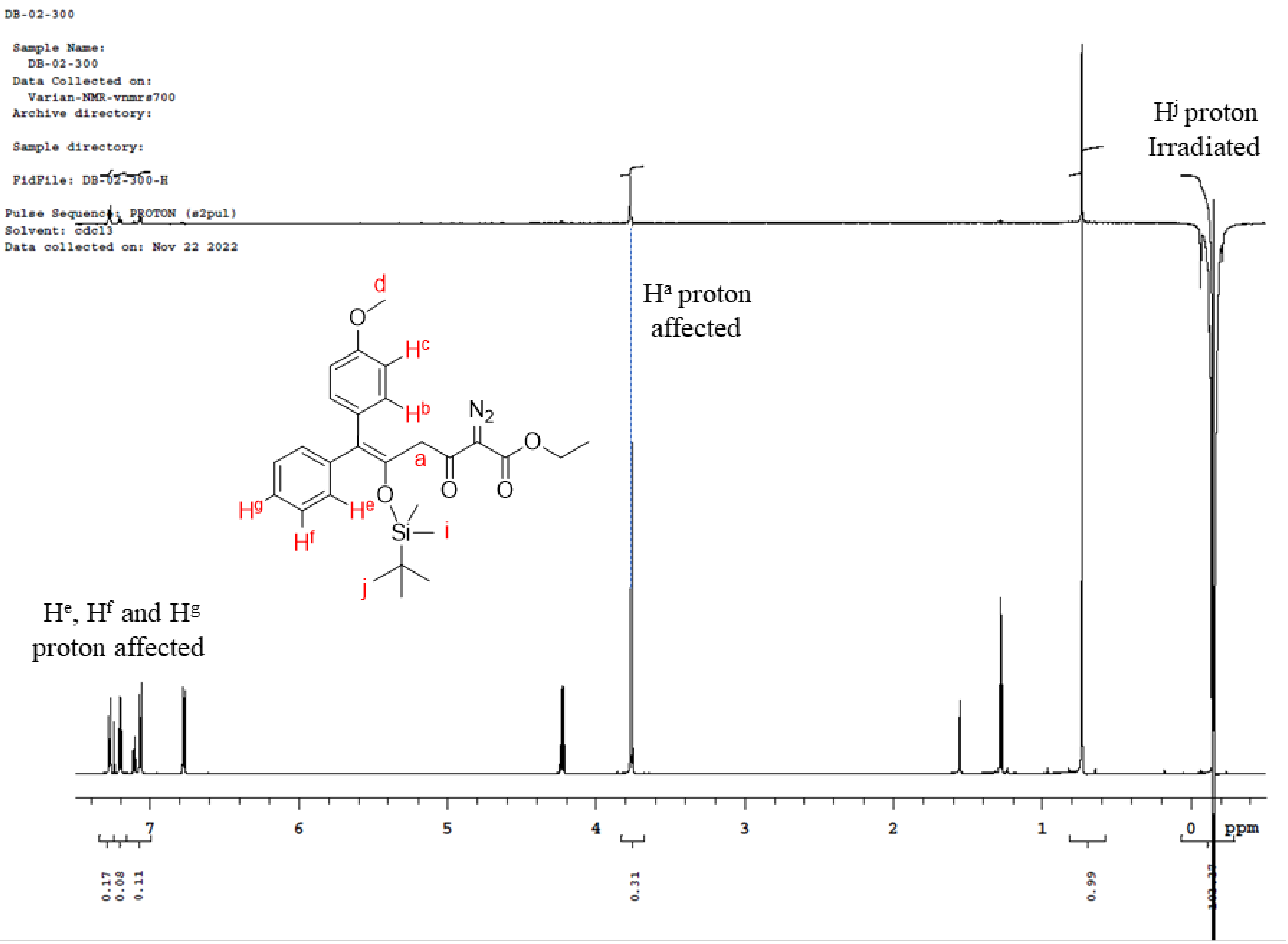

^{1}H NOE (700 MHz)

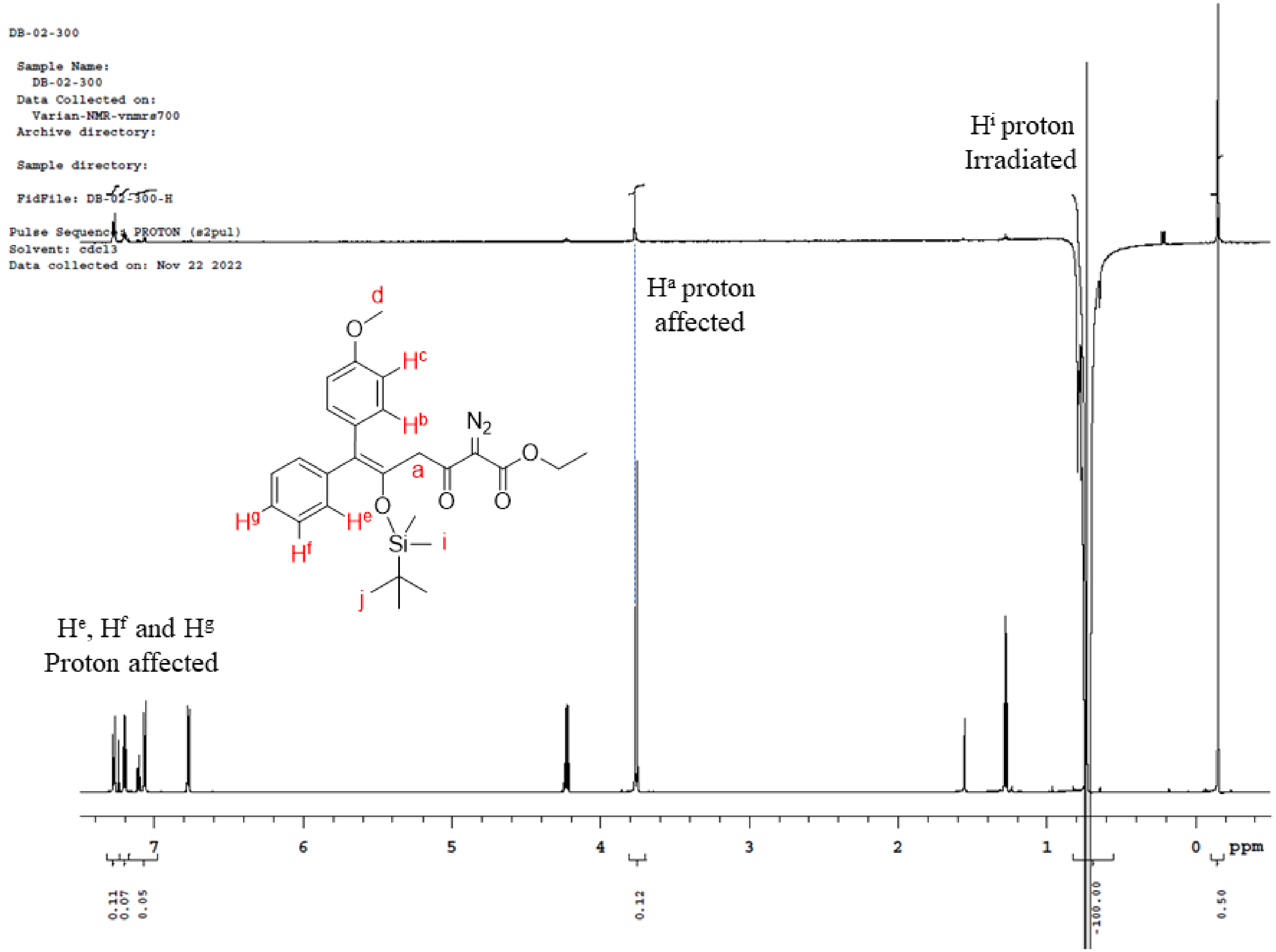

^{1}H NOE (700 MHz)

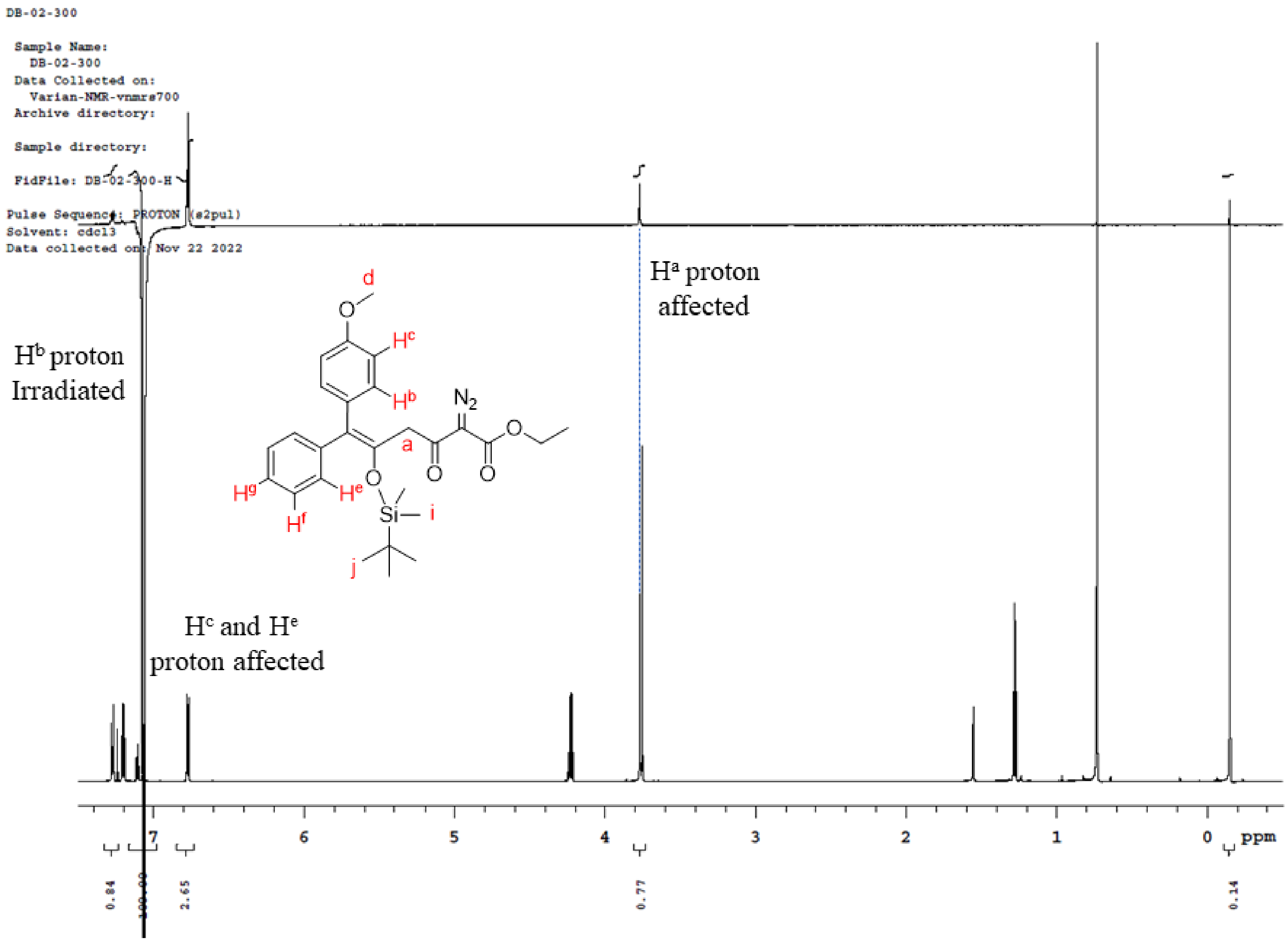

^{1}H NOE (700 MHz)

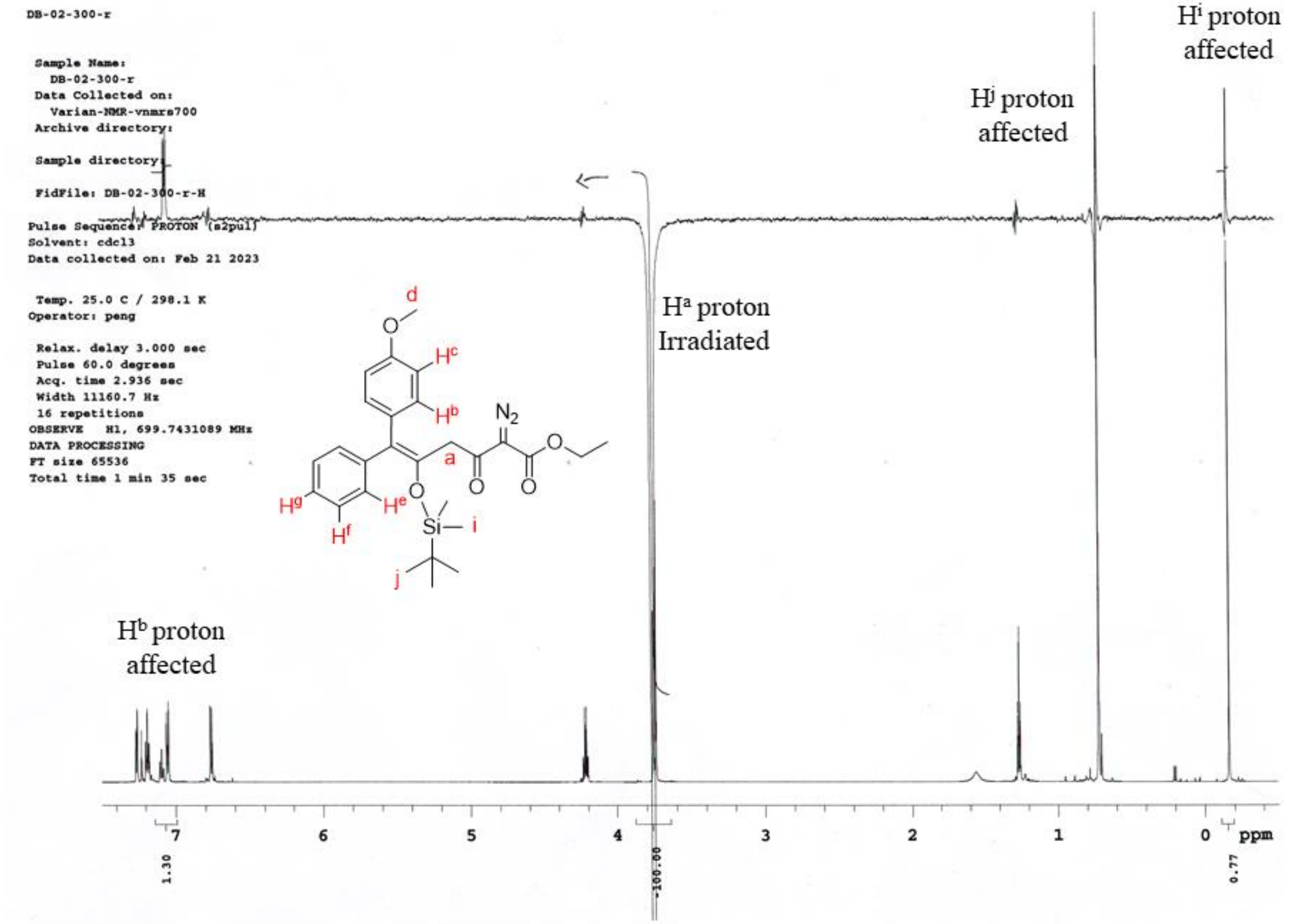

^{1}H NMR (CDCl$_3$, 700 MHz)

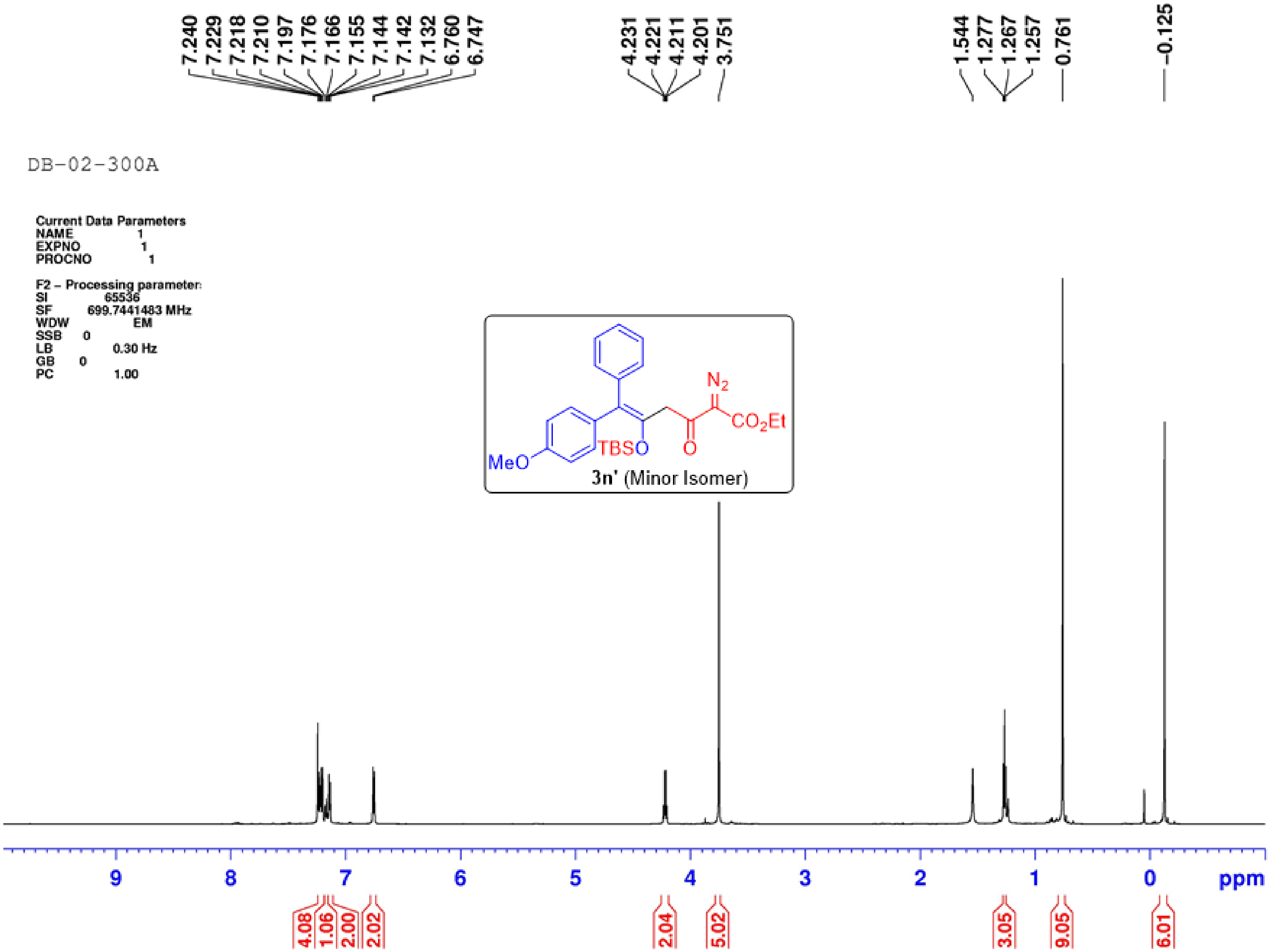

DEPT NMR ($CDCl_3$, 175 MHz)

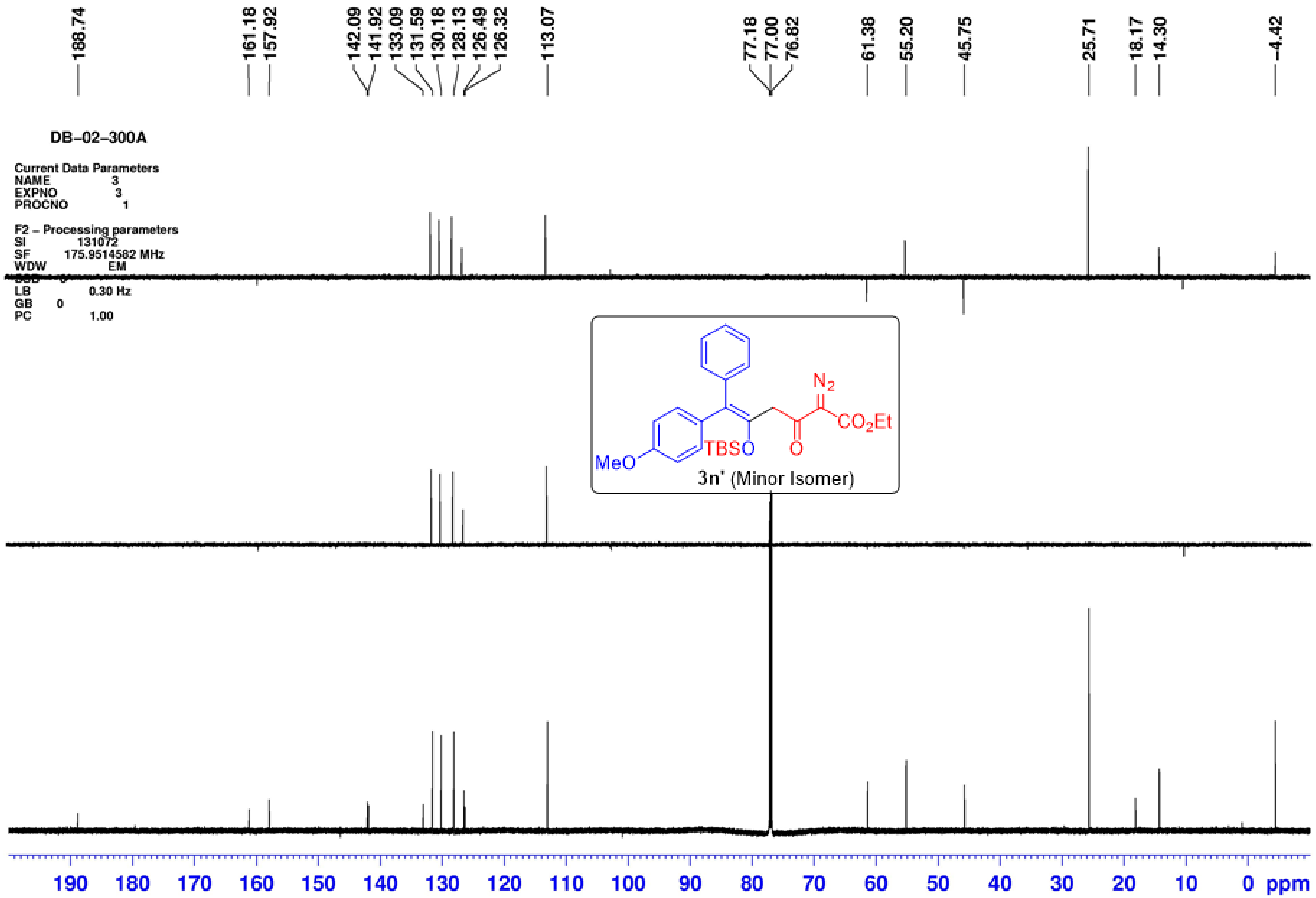

HMBC NMR ($CDCl_3$, 700 MHz)

DB-02-300A

Sample Name:
DB-02-300A
Data Collected on:
Varian-NMR-vnmrs700
Archive directory:

Sample directory:

FidFile: DB-02-300A-HMBC

Pulse Sequence: gHMBC
Solvent: cdcl3
Data collected on: Feb 17 2023

Temp. 25.0 C / 298.1 K
Operator: peng

Relax. delay 1.000 sec
Mixing 0.080 sec
Acq. time 0.268 sec
Width 6983.2 Hz
2D Width 40465.4 Hz
4 repetitions
2 x 256 increments
OBSERVE H1, 699.7430936 MHz
DATA PROCESSING
Sq. sine bell 0.075 sec
F1 DATA PROCESSING
Gauss apodization 0.004 sec
FT size 1024 x 2048
Total time 47 min

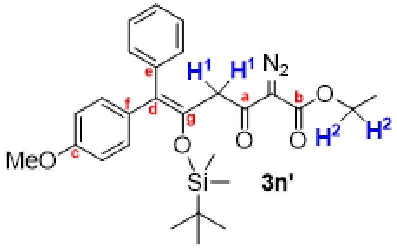

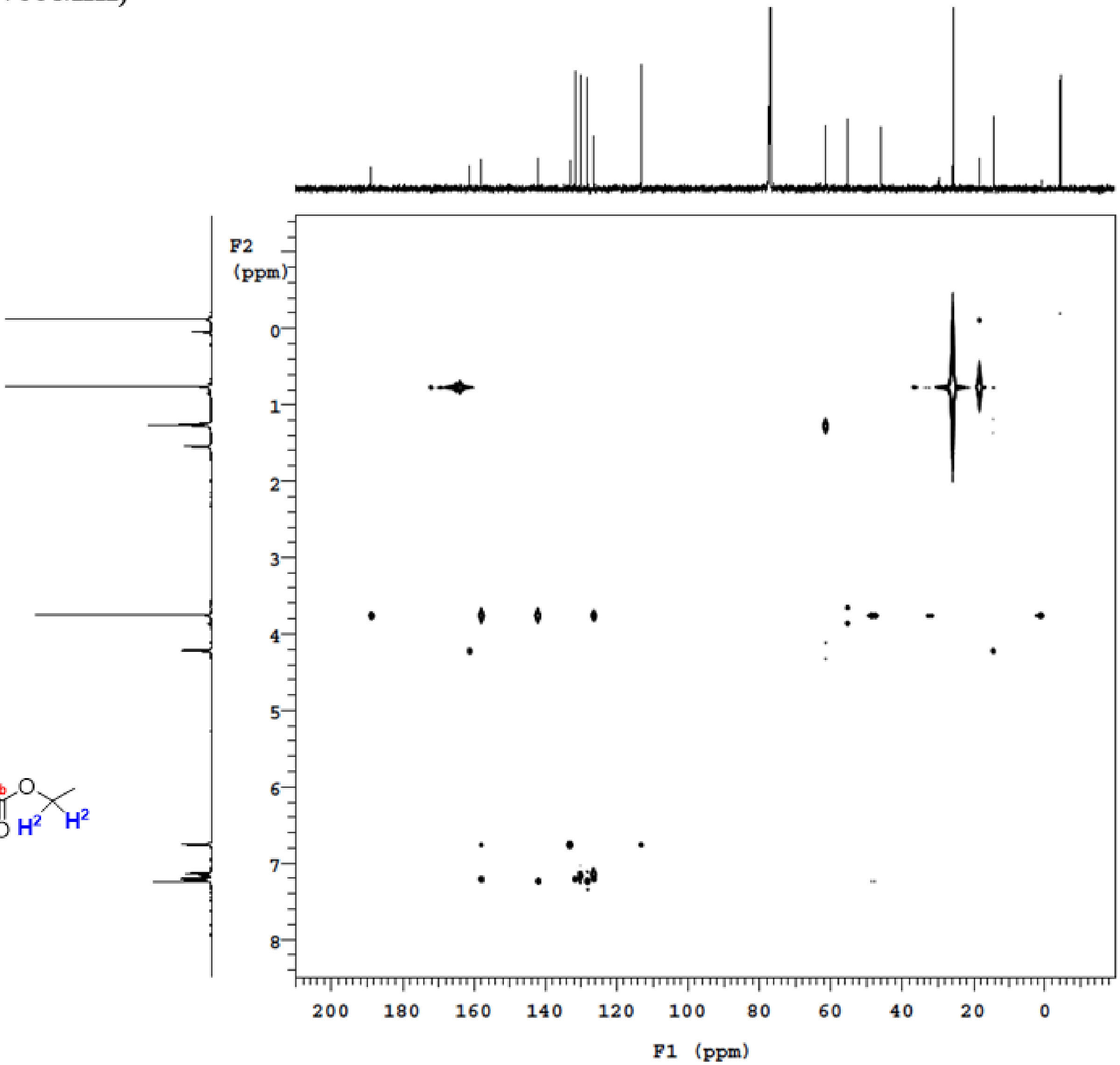

HSQC NMR (700 MHz)

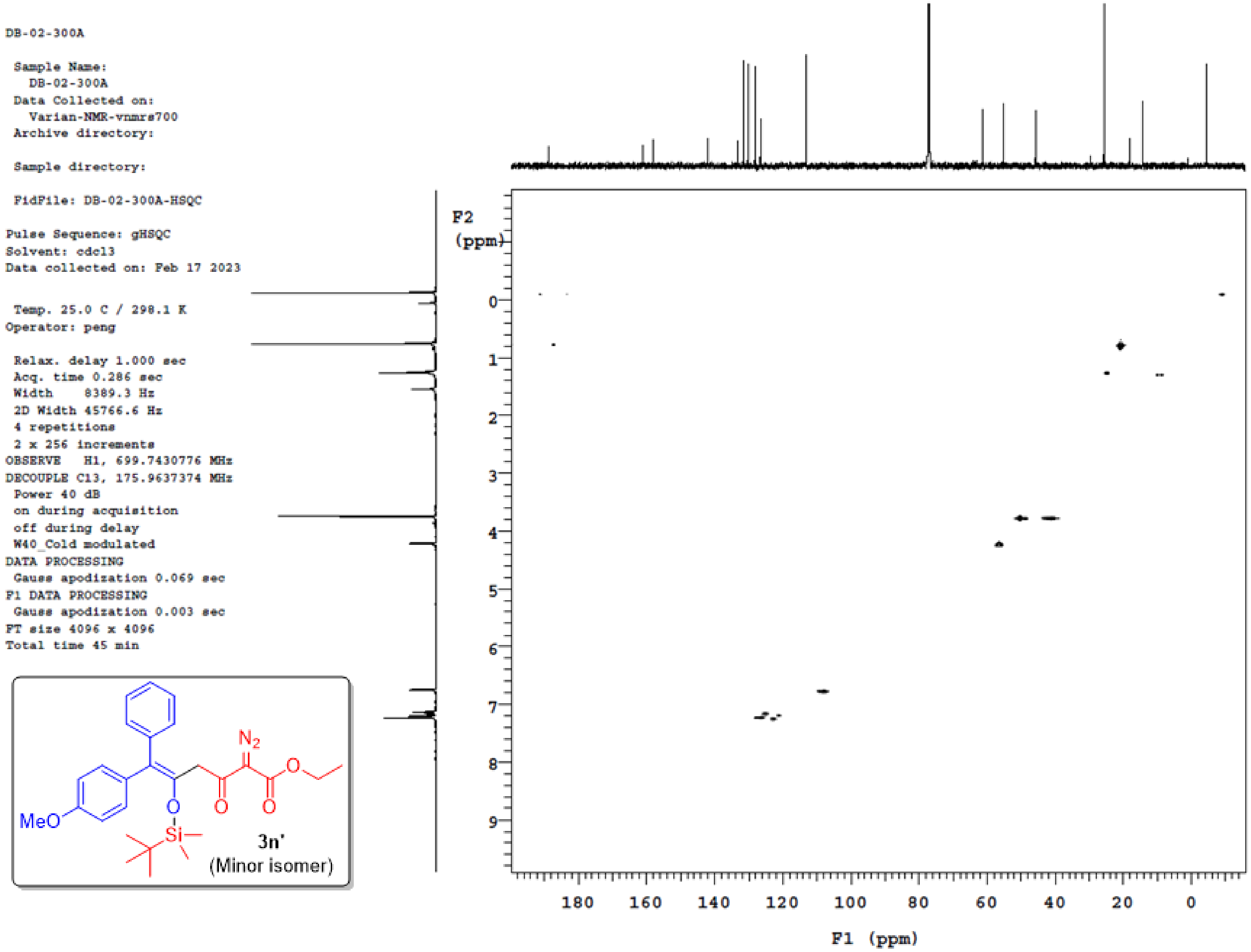

IR Spectral data

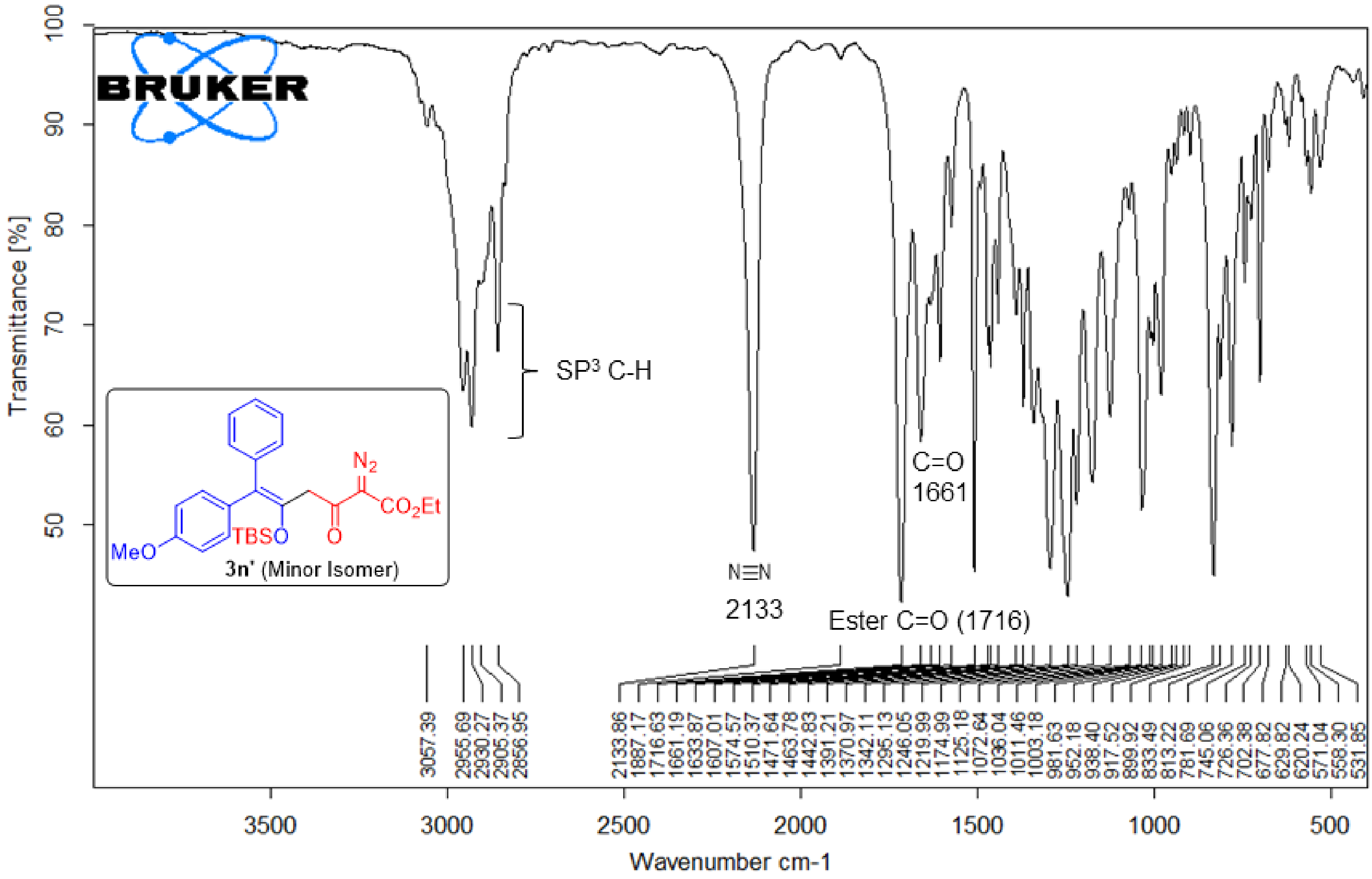

D:\FTIR FILES\2024\202411\20241101\NTHU\MIR_TR_DTGS_DB-02-300-A.0	MIR_TR_DTGS_DB-02-300-A	Instrument type and / or acc	01/11/2024

^{1}H NOE NMR (700 MHz)

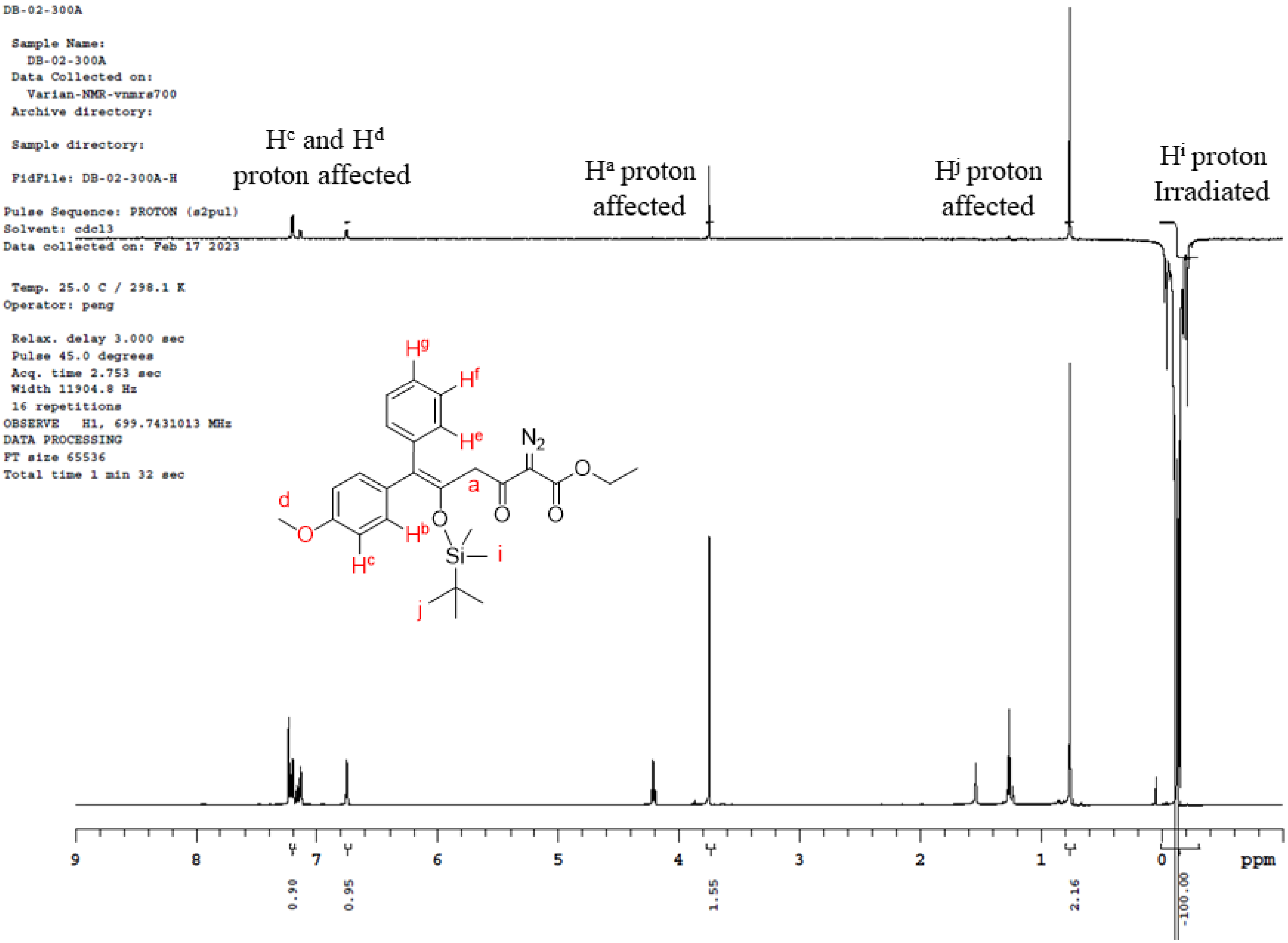

^{1}H NOE NMR (700 MHz)

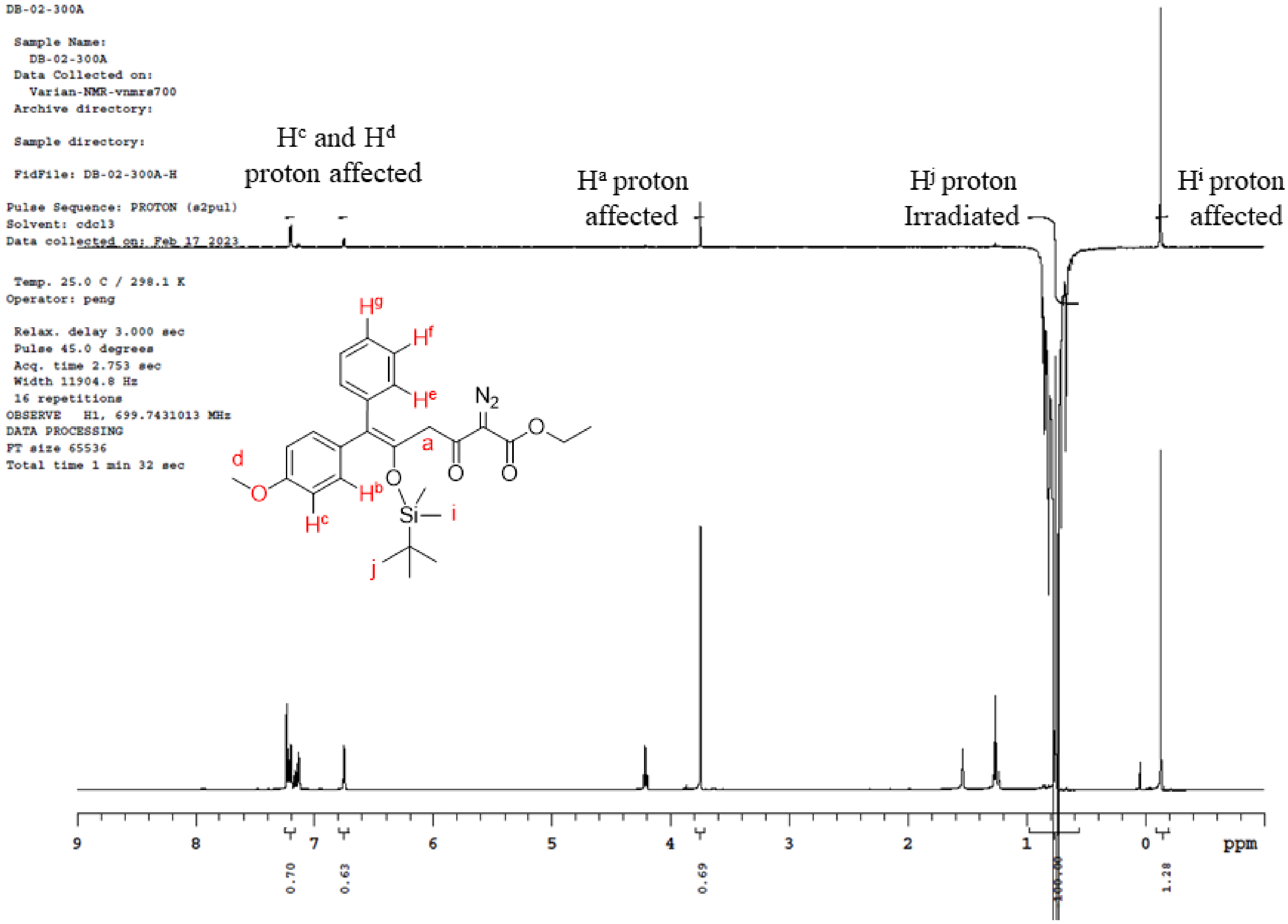

^{1}H NMR (CDCl$_3$, 700 MHz)

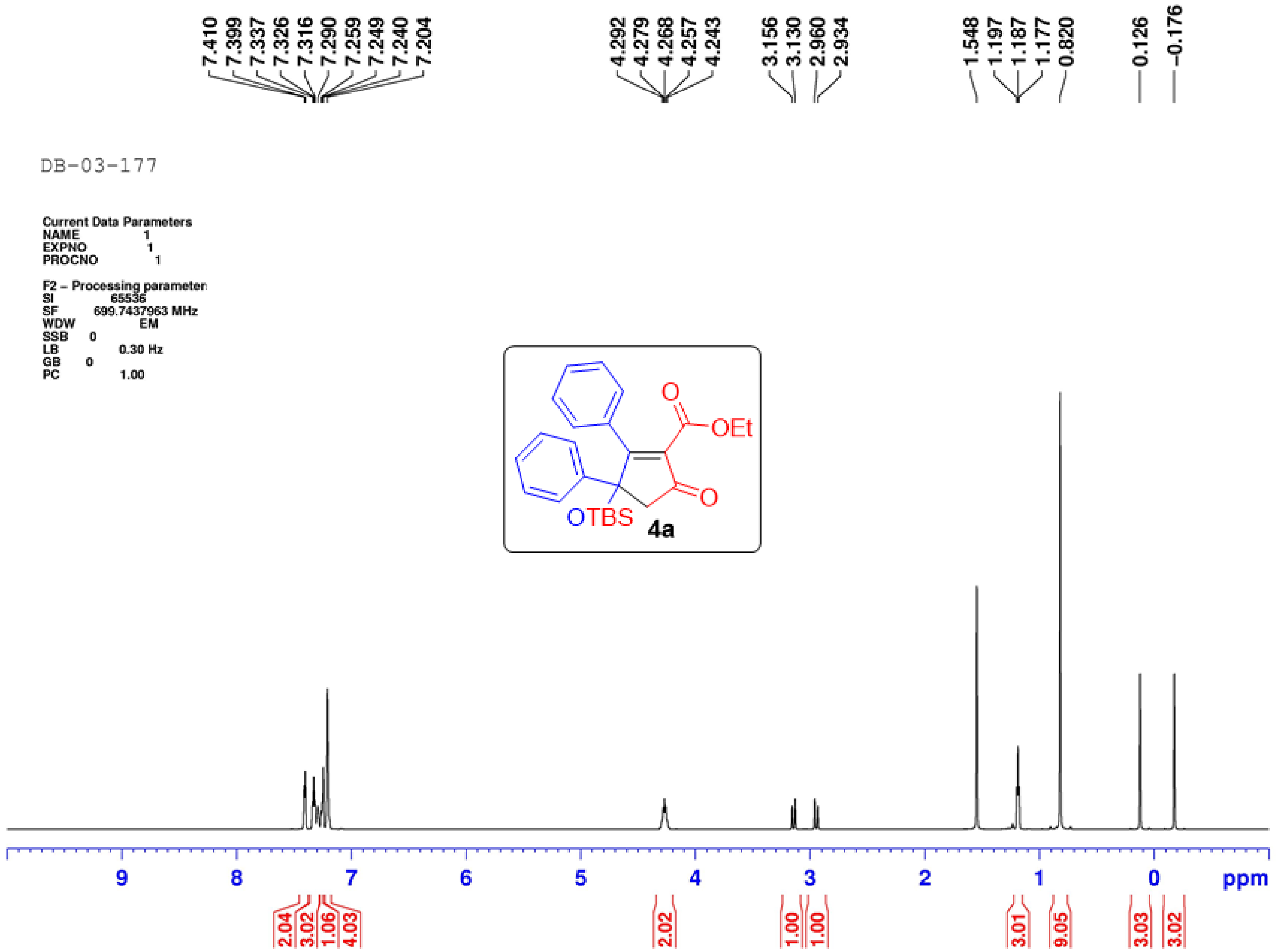

^{13}C NMR ($CDCl_3$, 175 MHz)

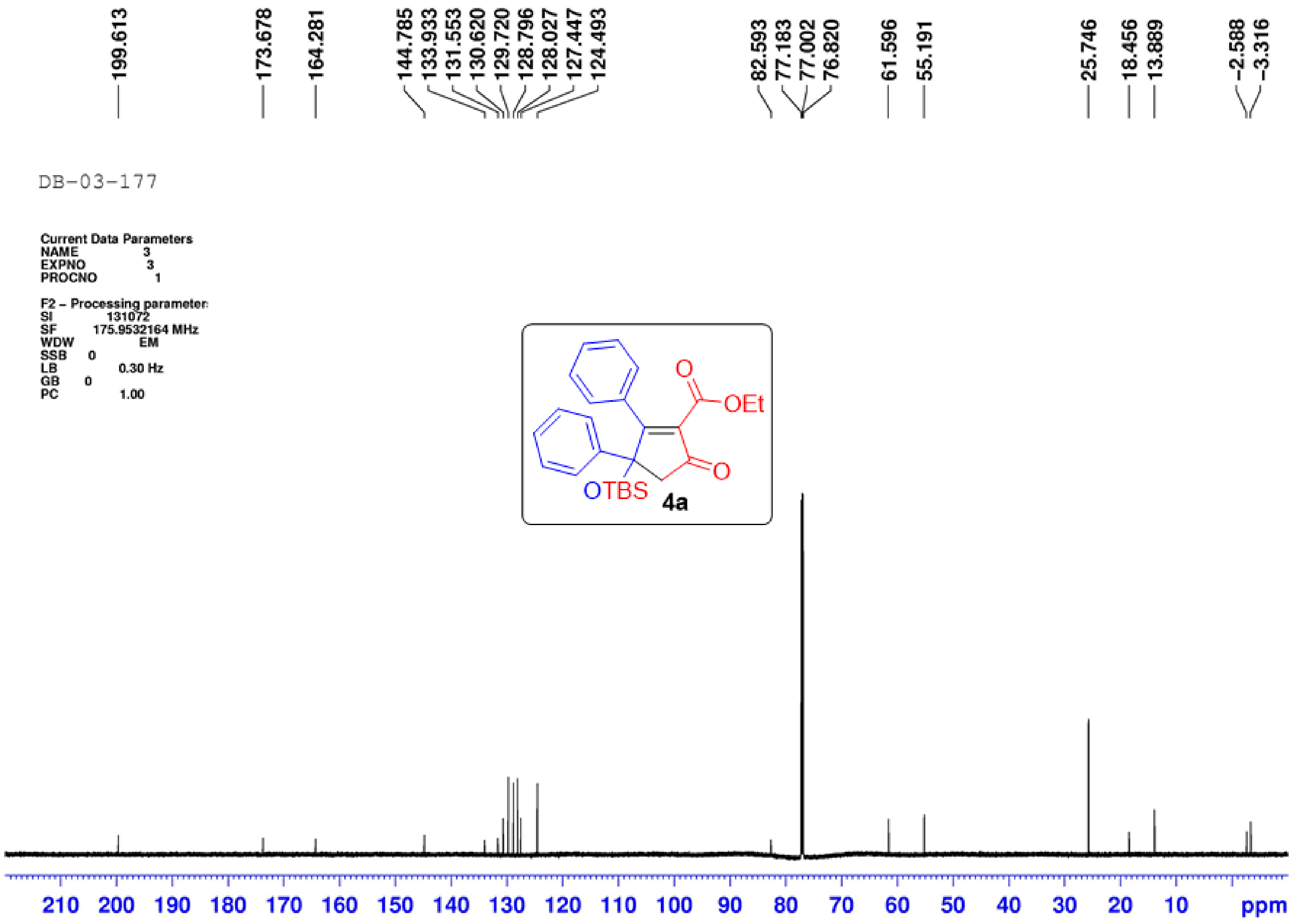

^{1}H NMR ($CDCl_3$, 700 MHz)

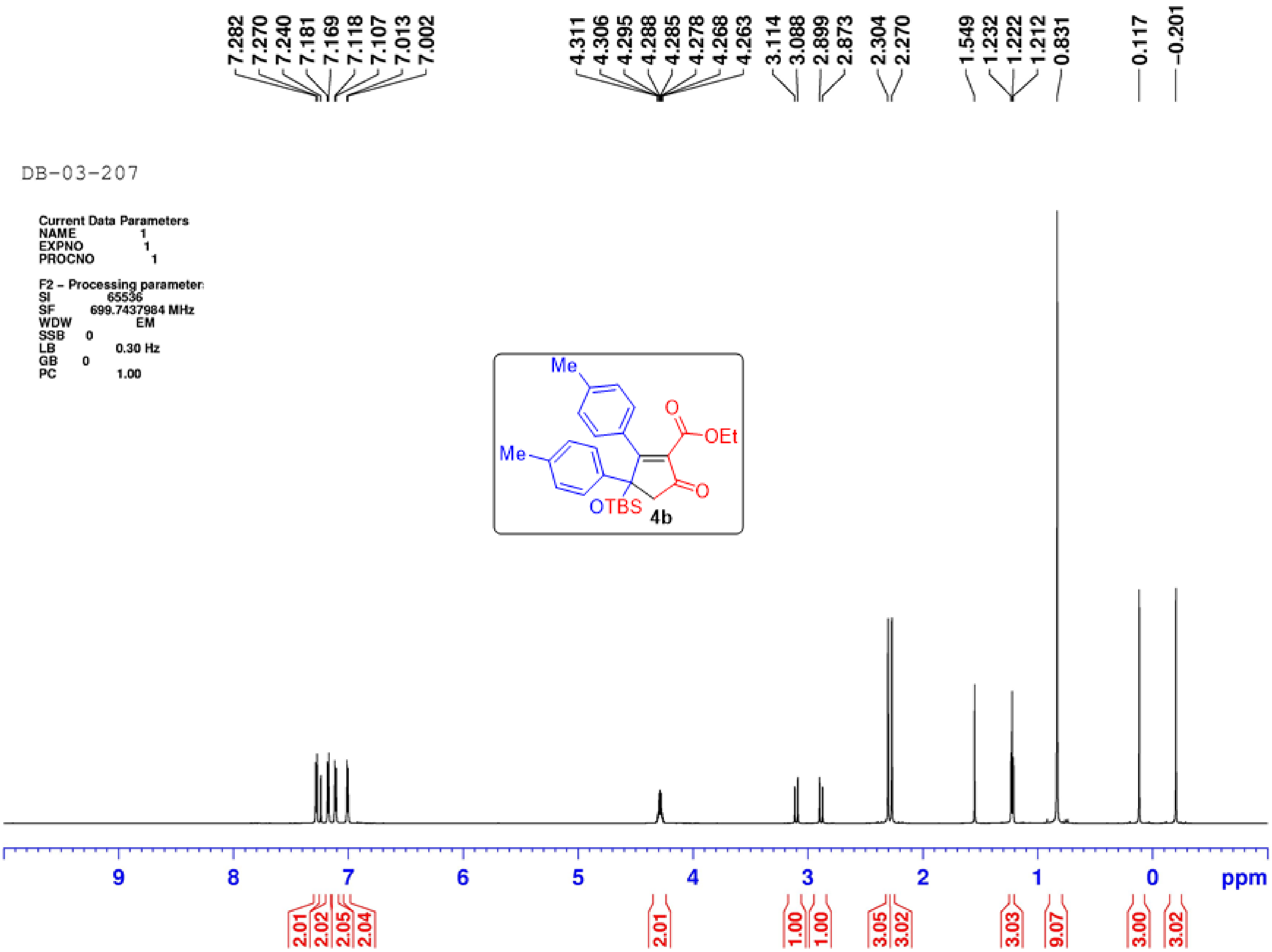

^{13}C NMR ($CDCl_3$, 175 MHz)

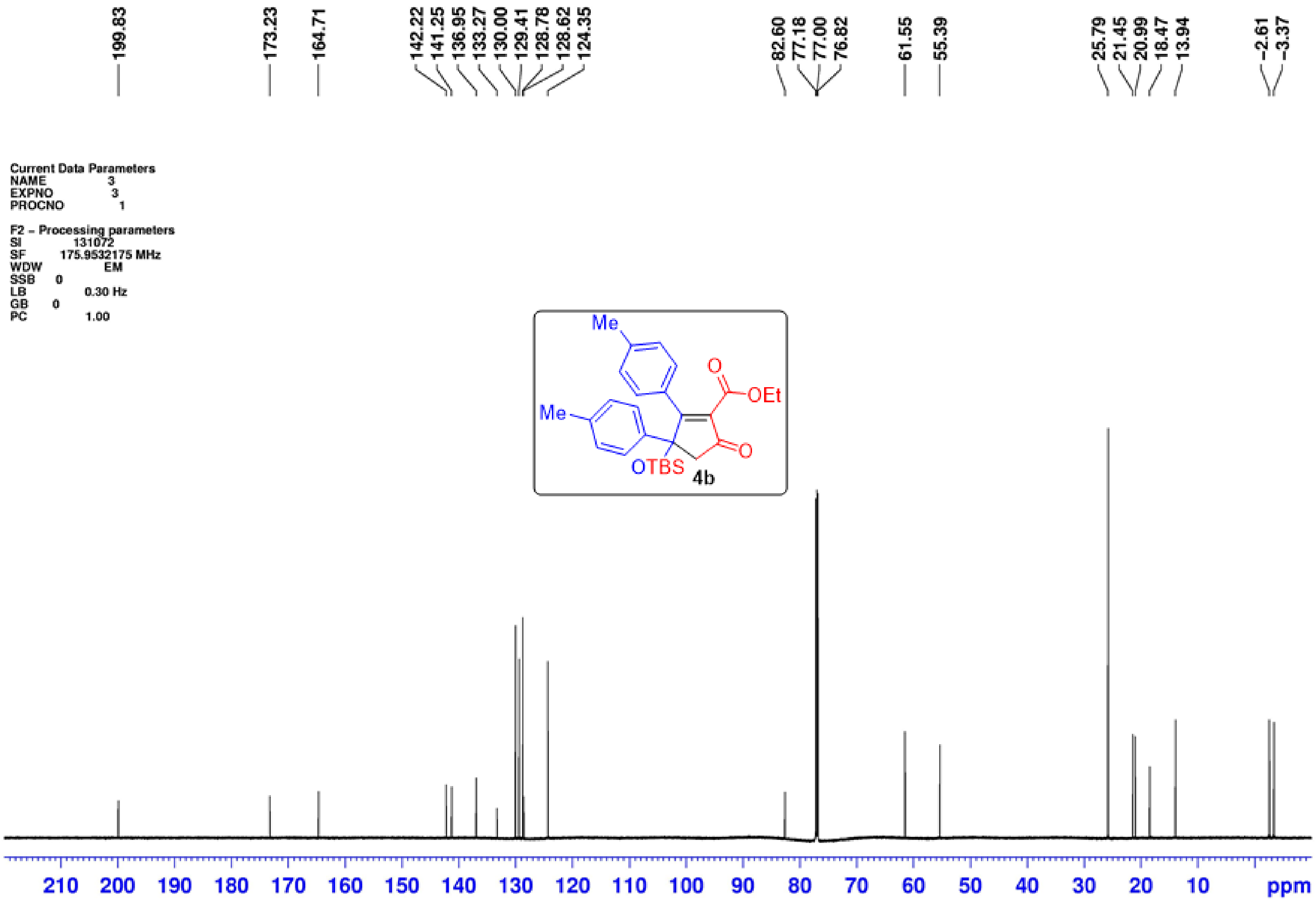

^{1}H NMR ($CDCl_3$, 700 MHz)

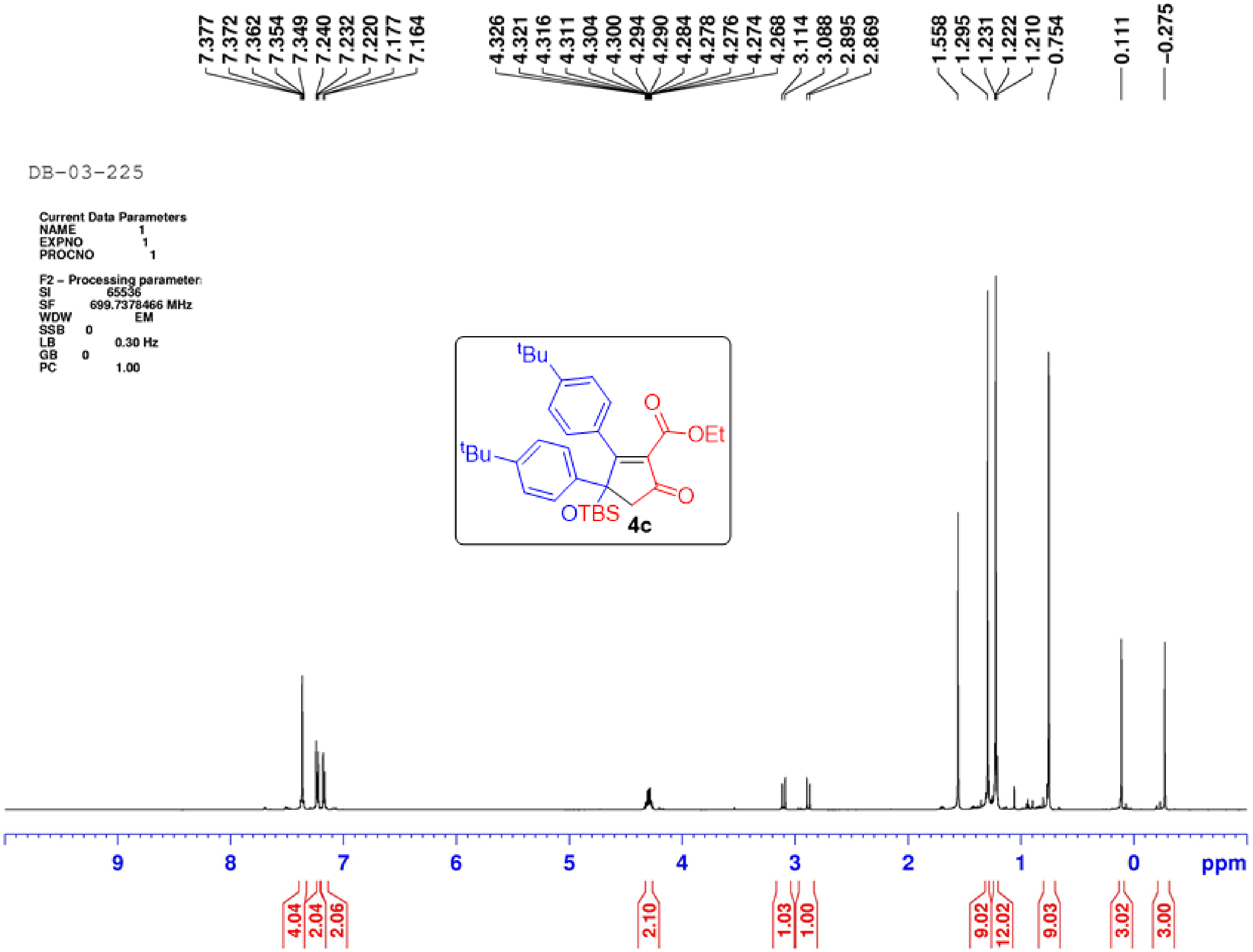

^{13}C NMR ($CDCl_3$, 175 MHz)

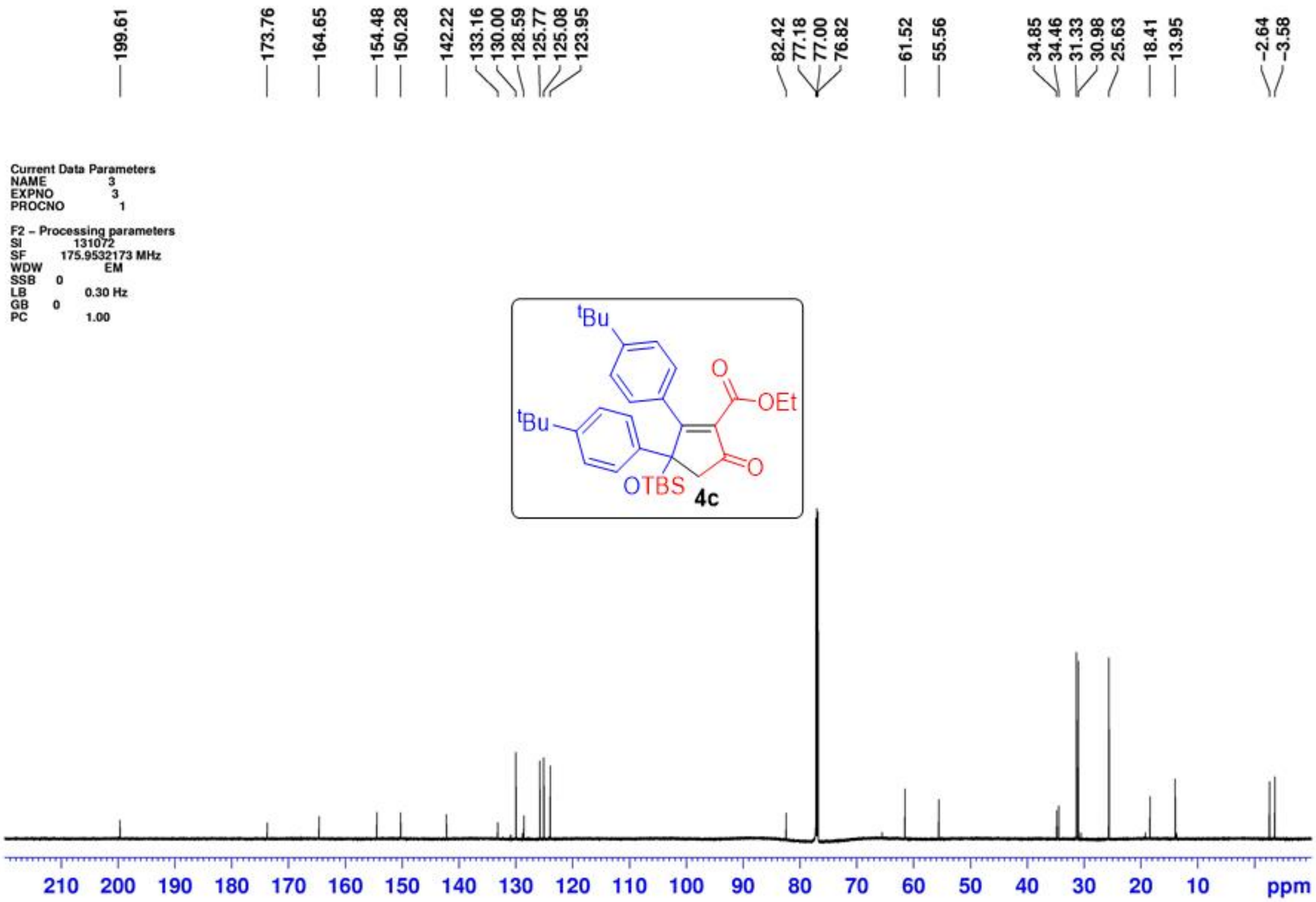

^{1}H NMR ($CDCl_3$, 700 MHz)

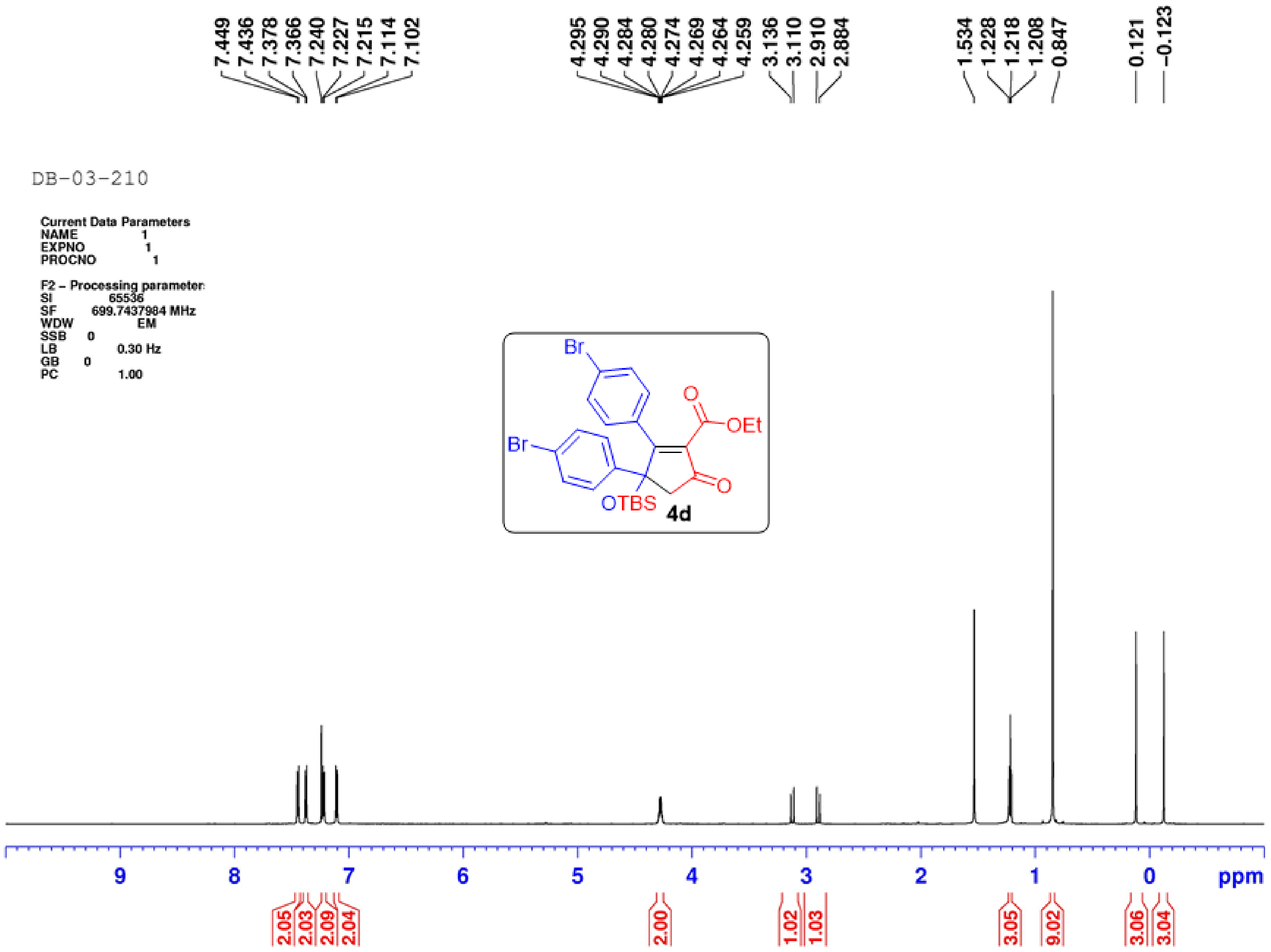

^{13}C NMR ($CDCl_3$, 175 MHz)

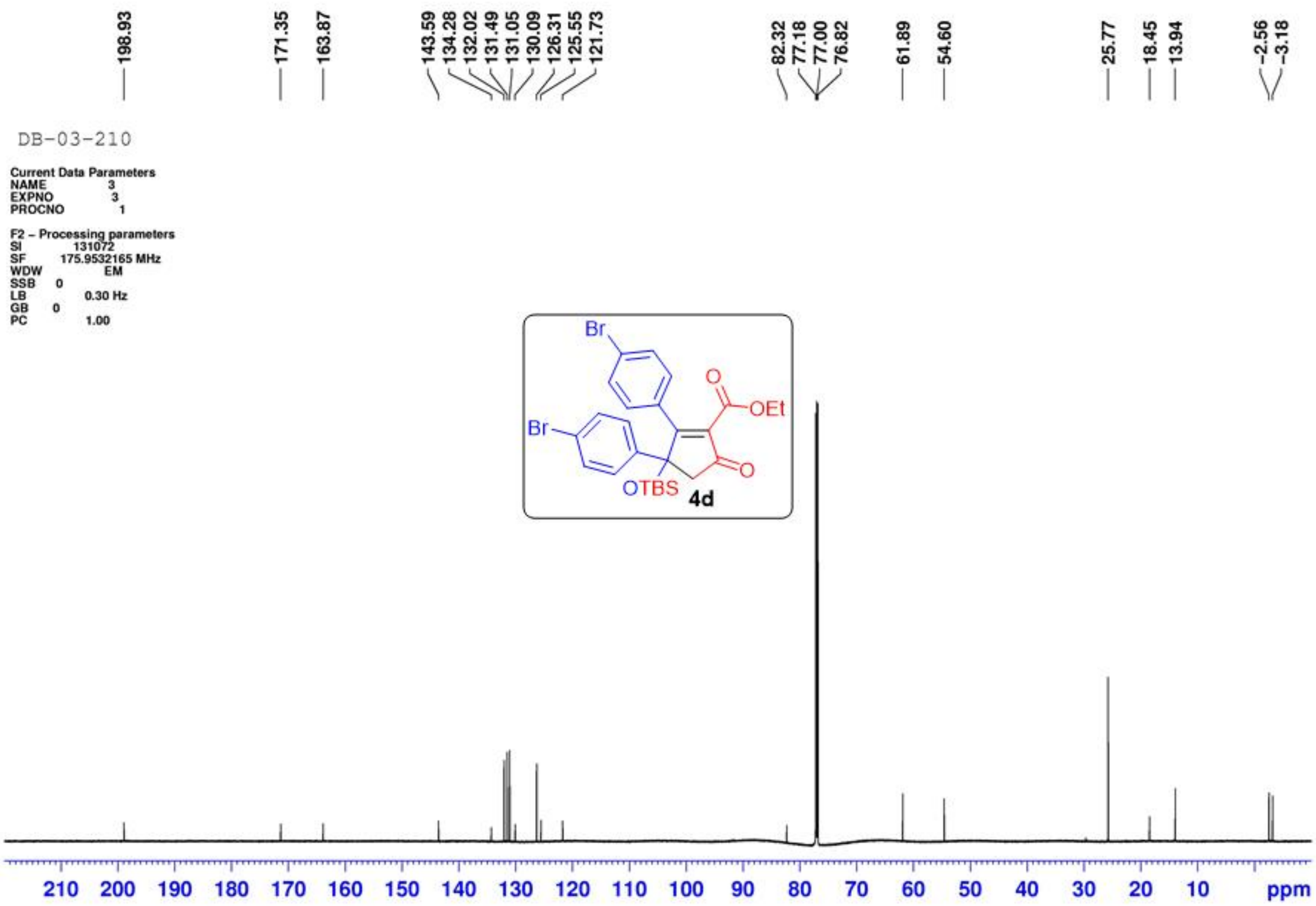

^{1}H NMR ($CDCl_3$, 700 MHz)

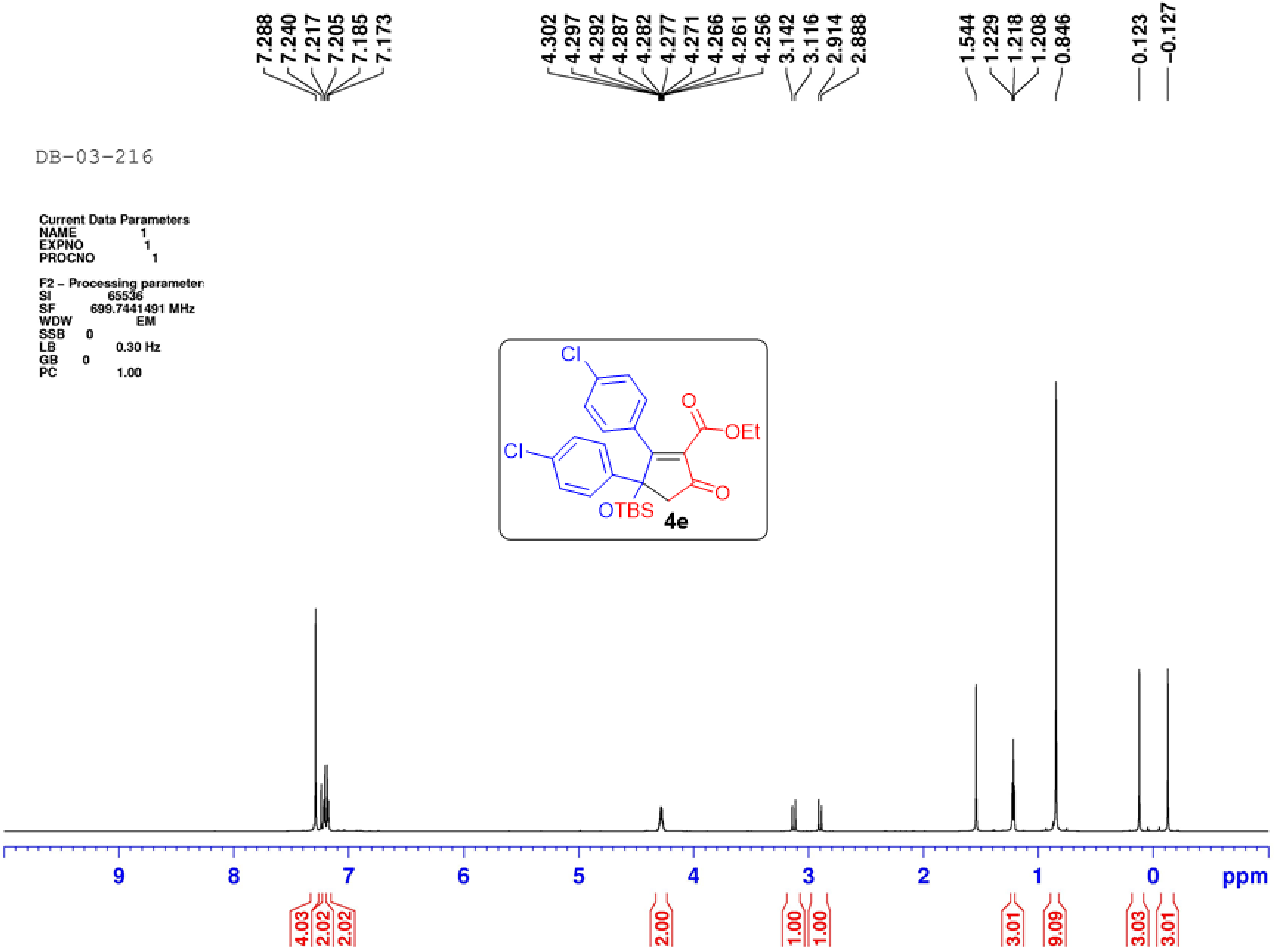

^{13}C NMR (CDCl$_3$, 175 MHz)

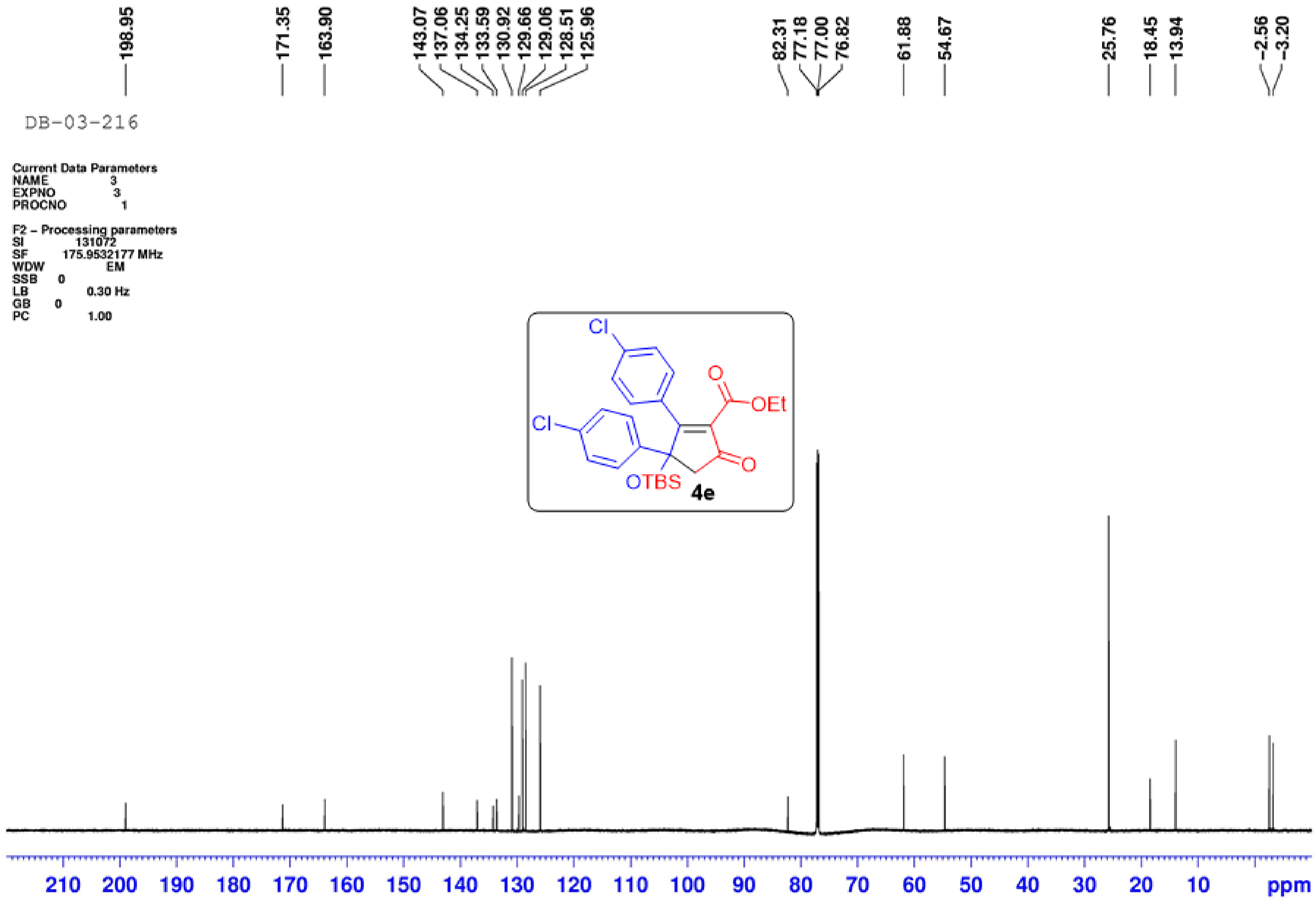

^{1}H NMR ($CDCl_3$, 700 MHz)

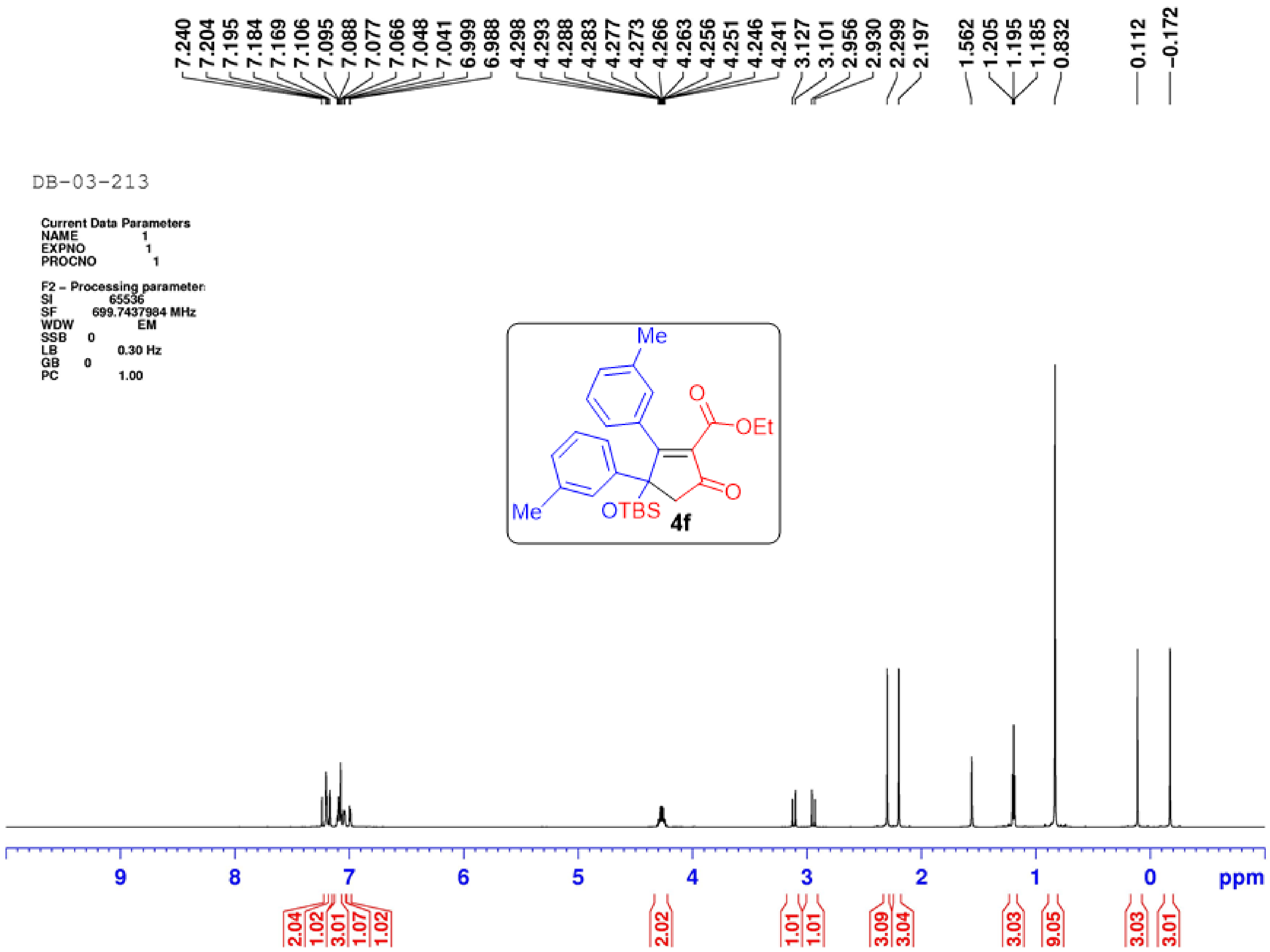

^{13}C NMR (CDCl$_3$, 175 MHz)

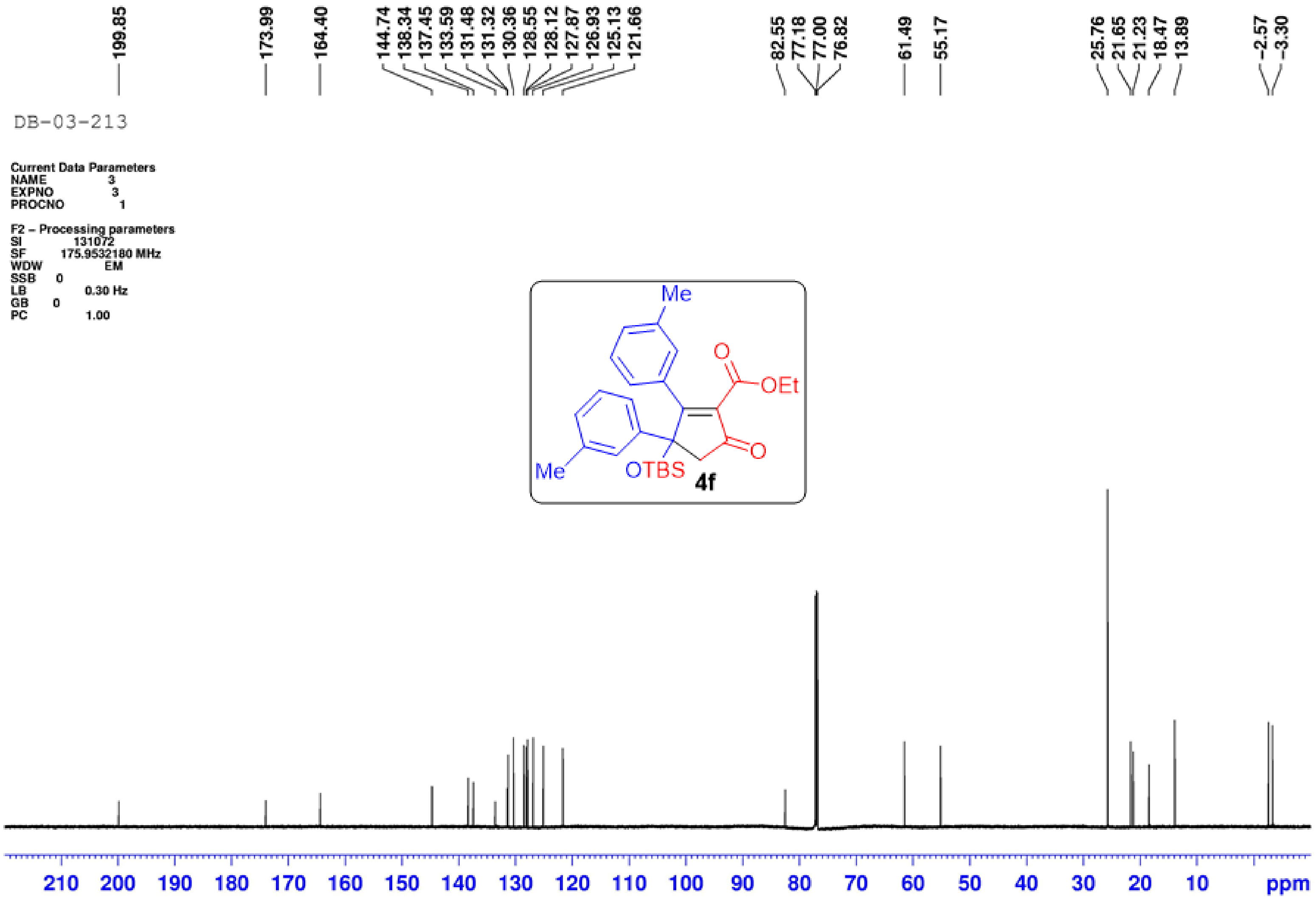

^{1}H NMR (CDCl$_3$, 700 MHz)

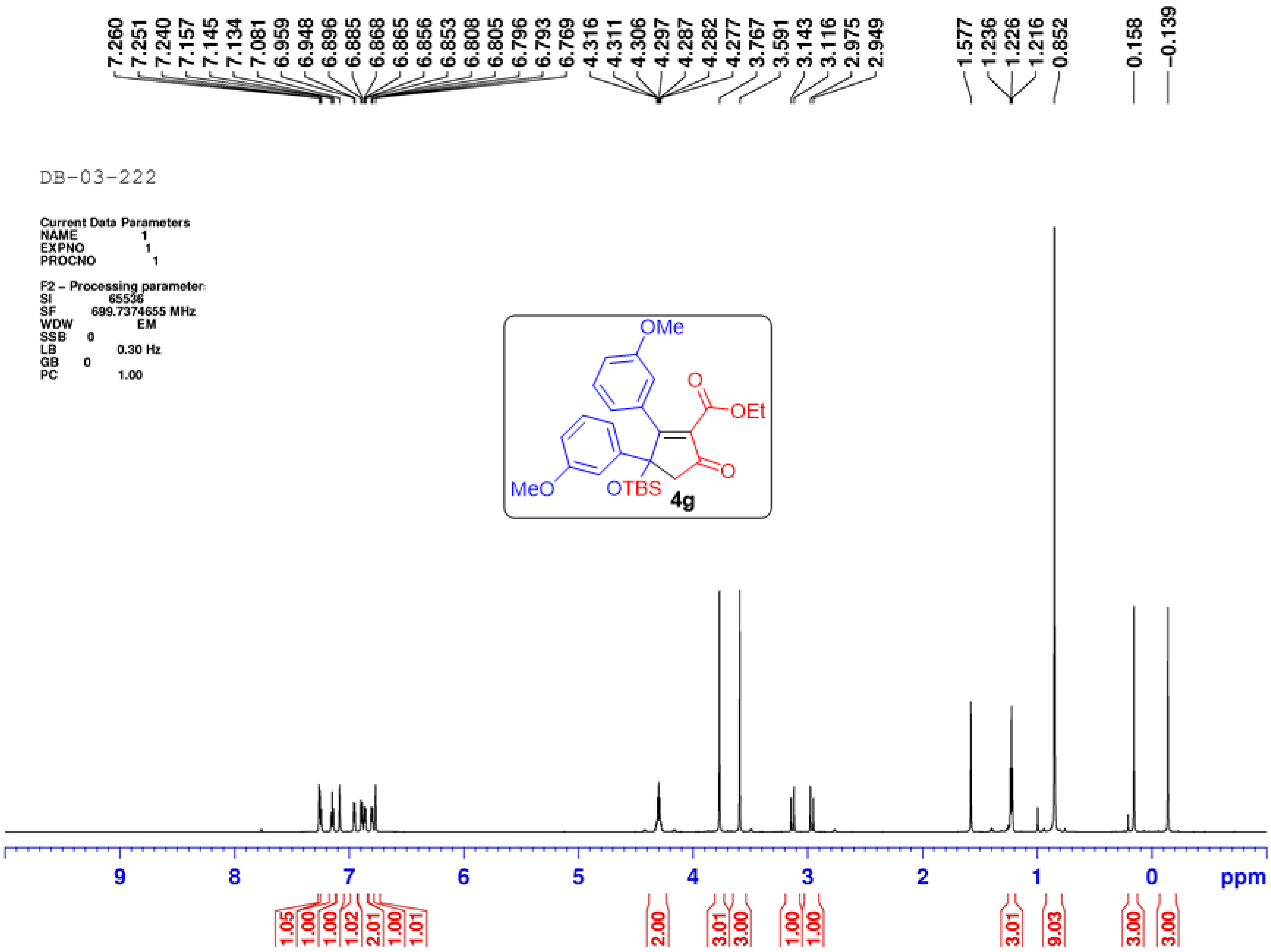

^{13}C NMR (CDCl$_3$, 175 MHz)

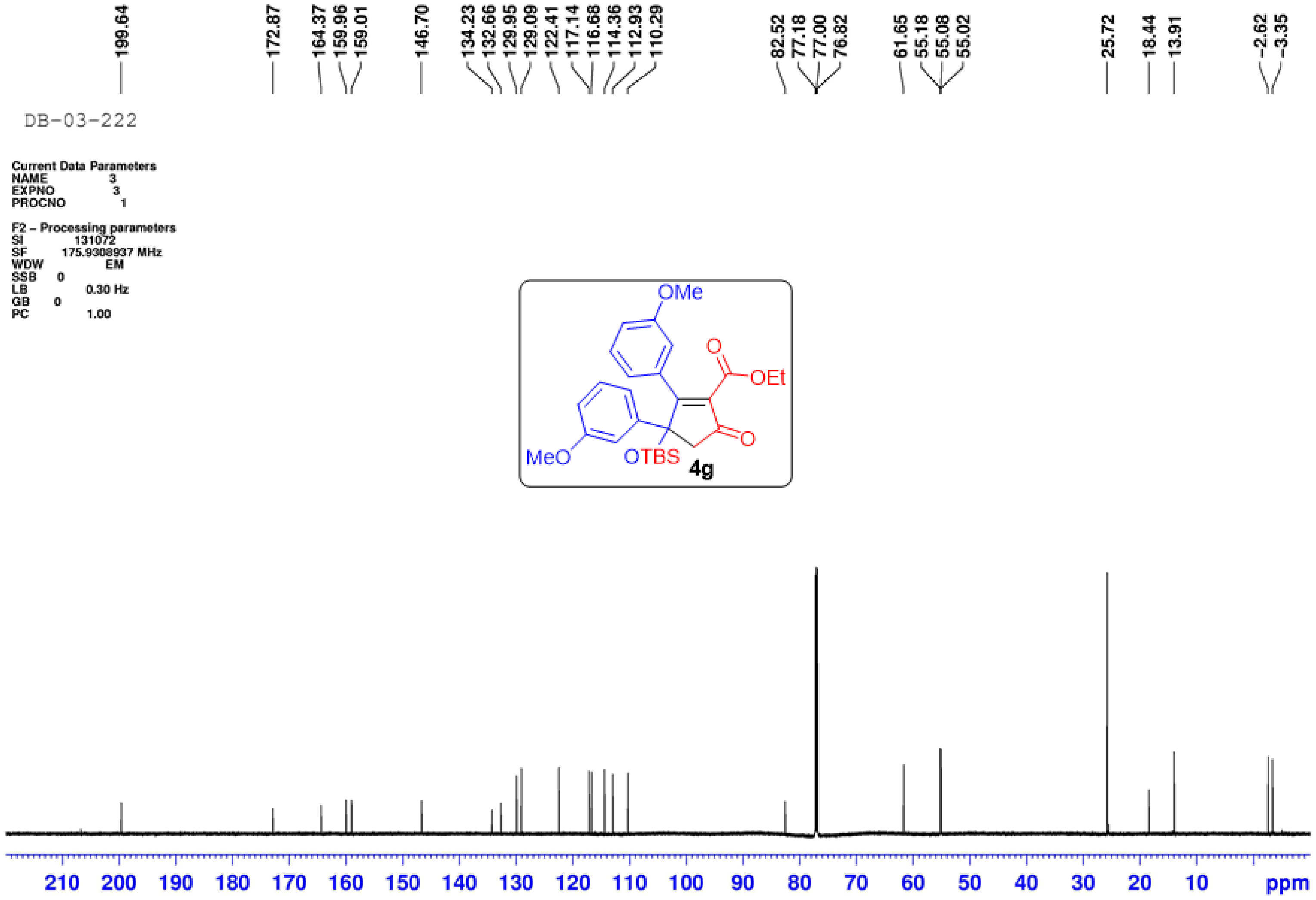

^{1}H NMR ($CDCl_3$, 700 MHz)

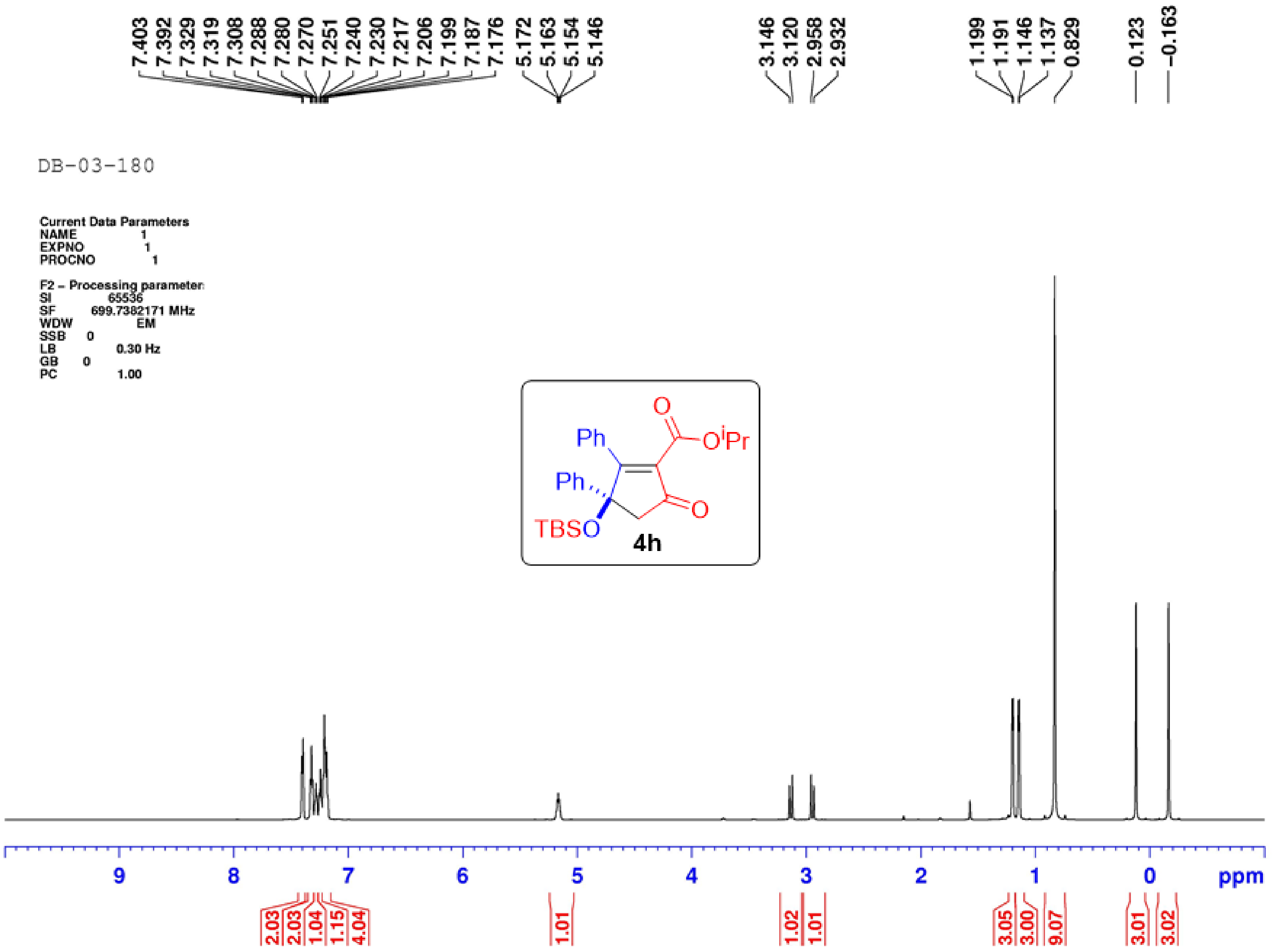

^{13}C NMR ($CDCl_3$, 175 MHz)

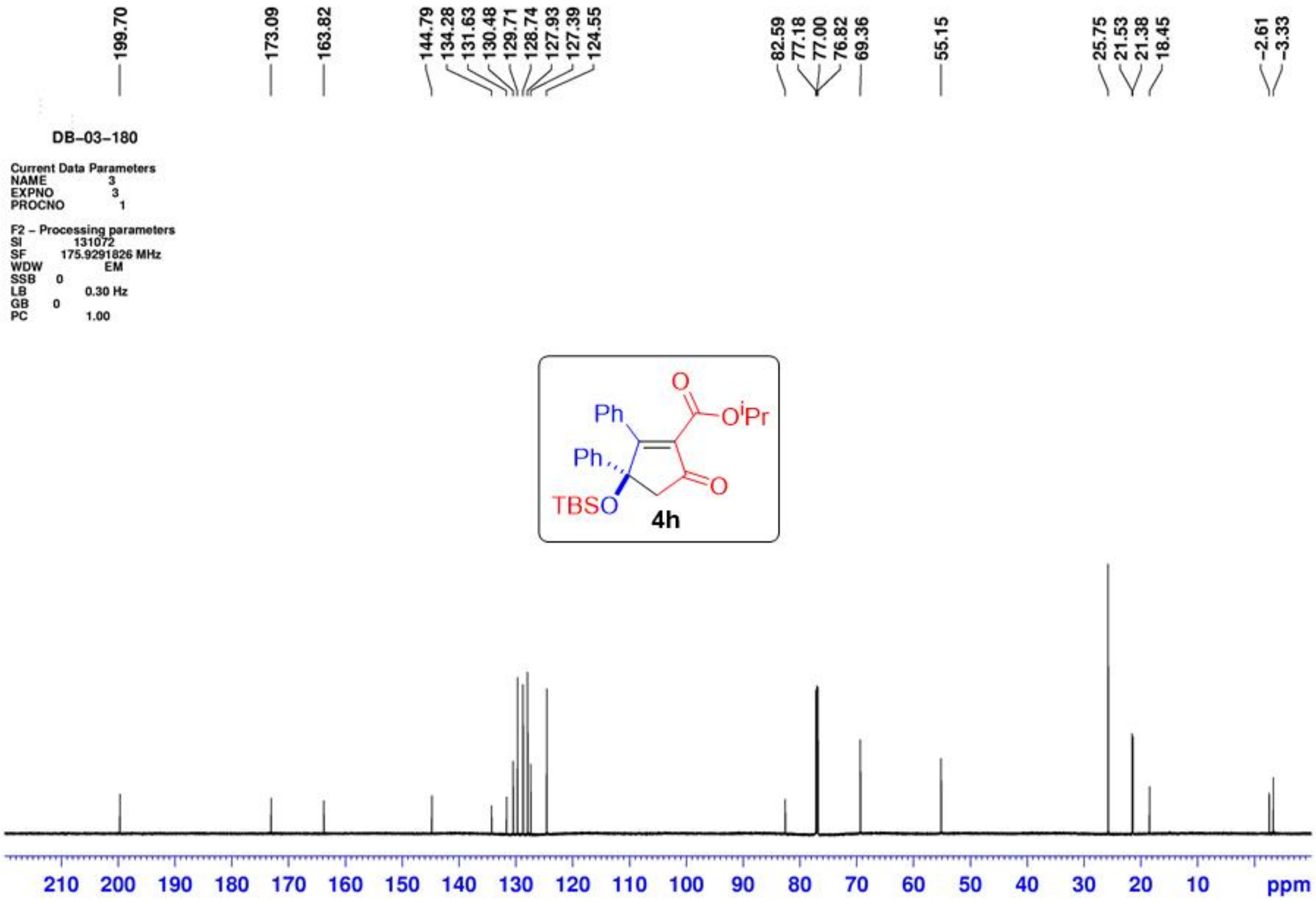

^{1}H NMR (CDCl$_3$, 700 MHz)

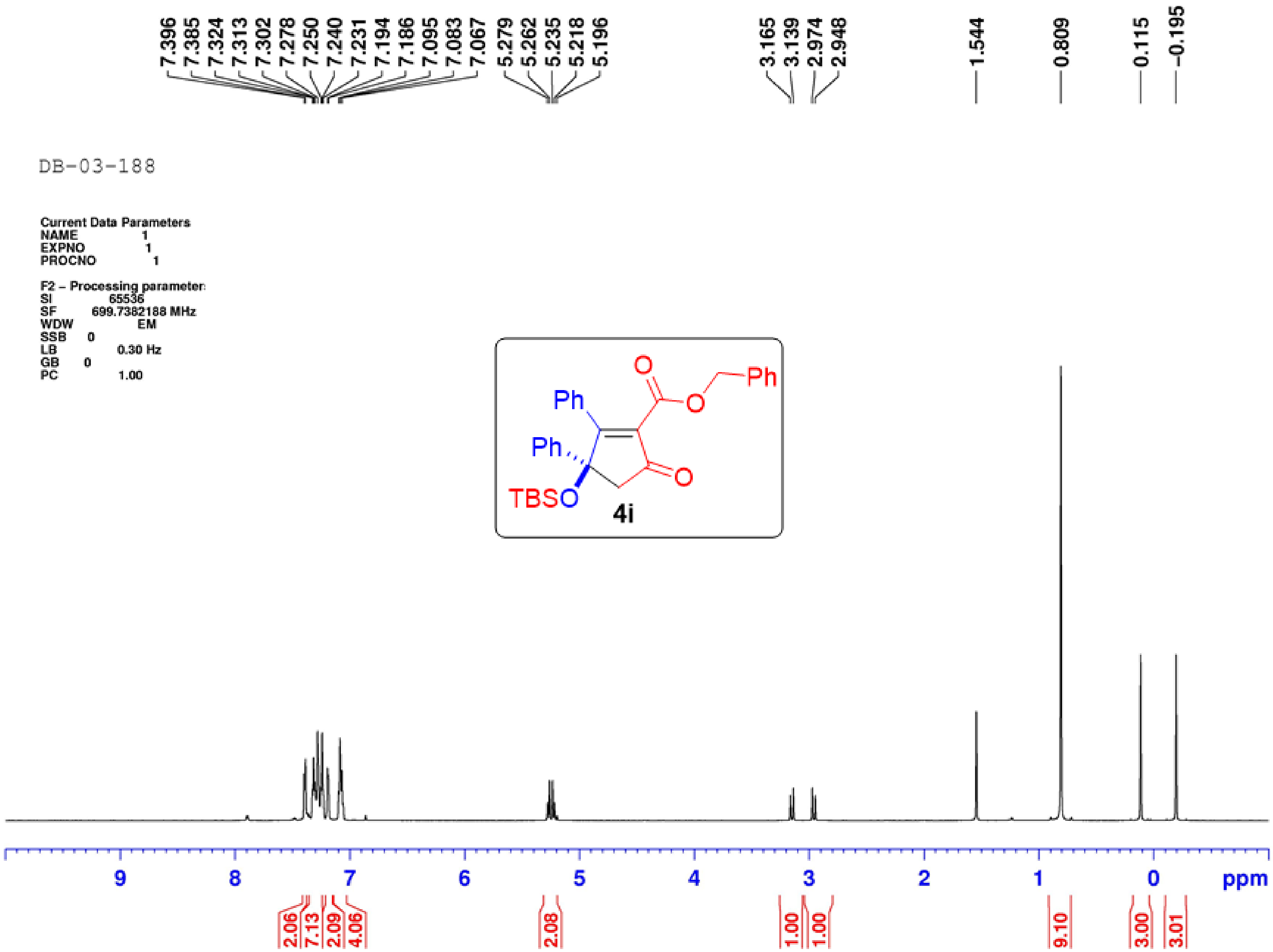

^{13}C NMR ($CDCl_3$, 175 MHz)

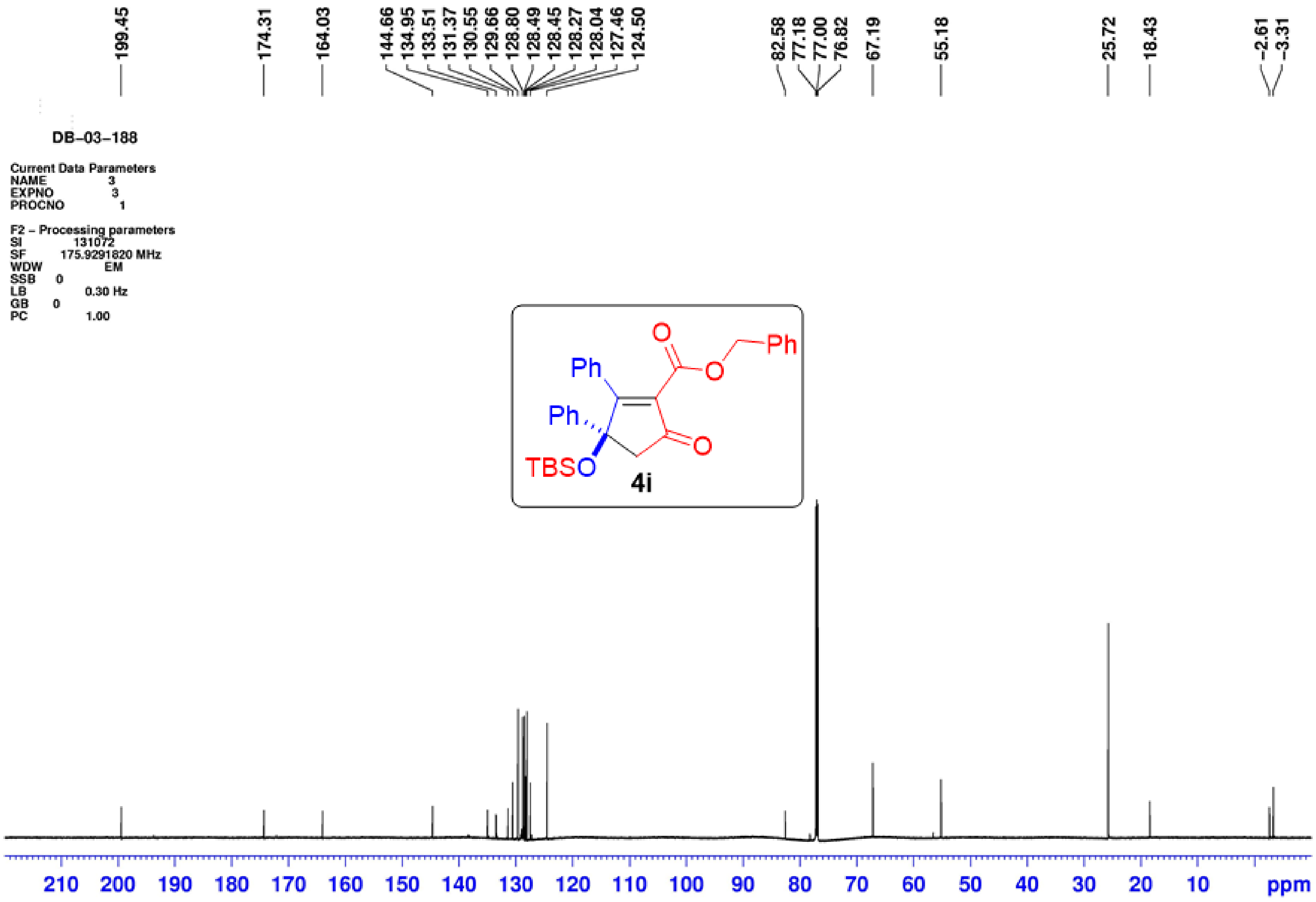

^{1}H NMR ($CDCl_3$, 700 MHz)

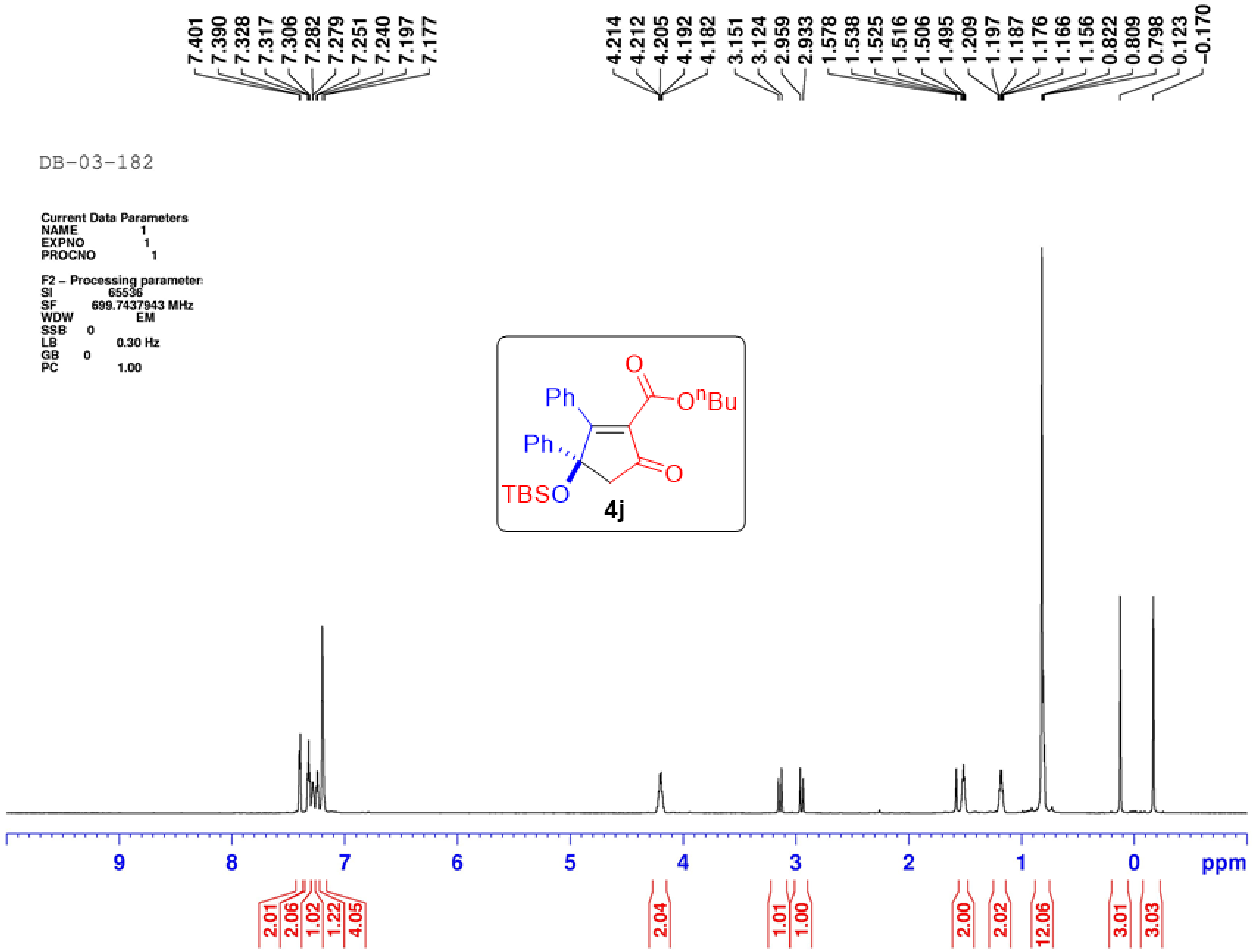

^{13}C NMR ($CDCl_3$, 175 MHz)

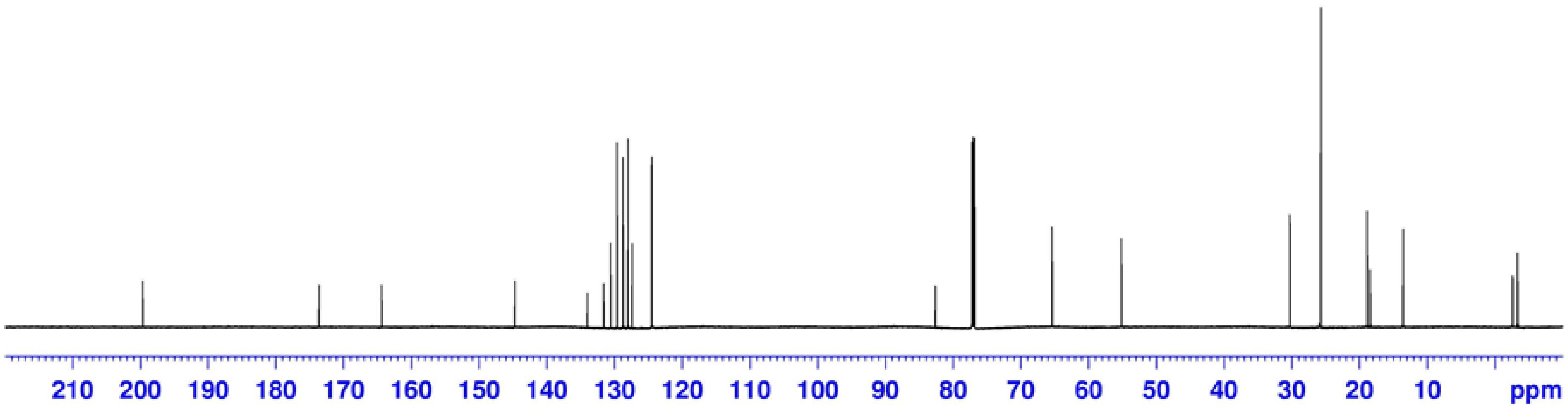

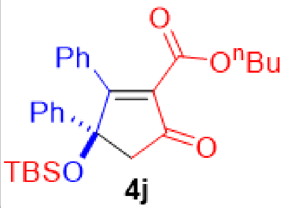

4j

^{1}H NMR ($CDCl_3$, 700 MHz)

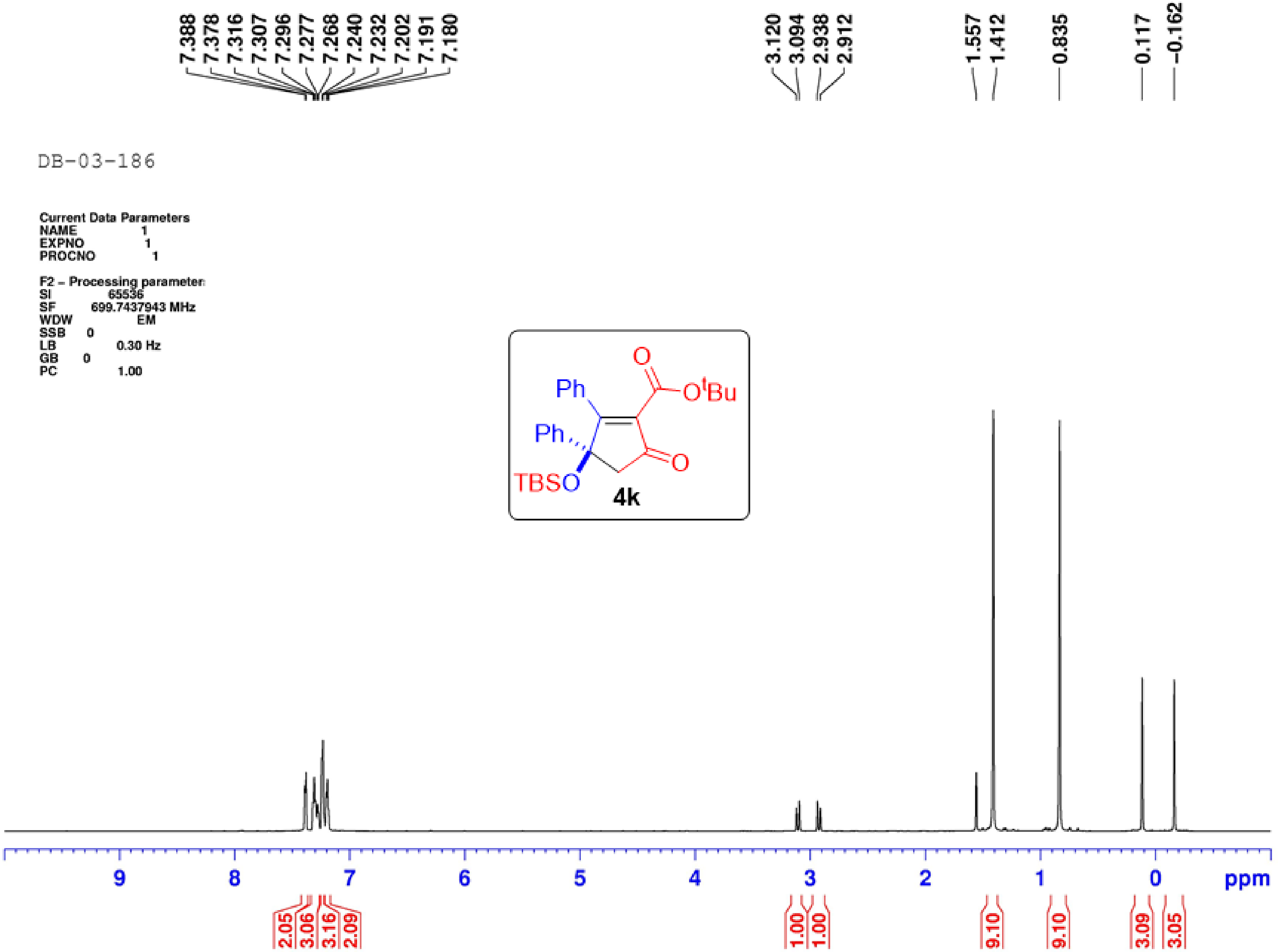

^{13}C NMR (CDCl$_3$, 175 MHz)

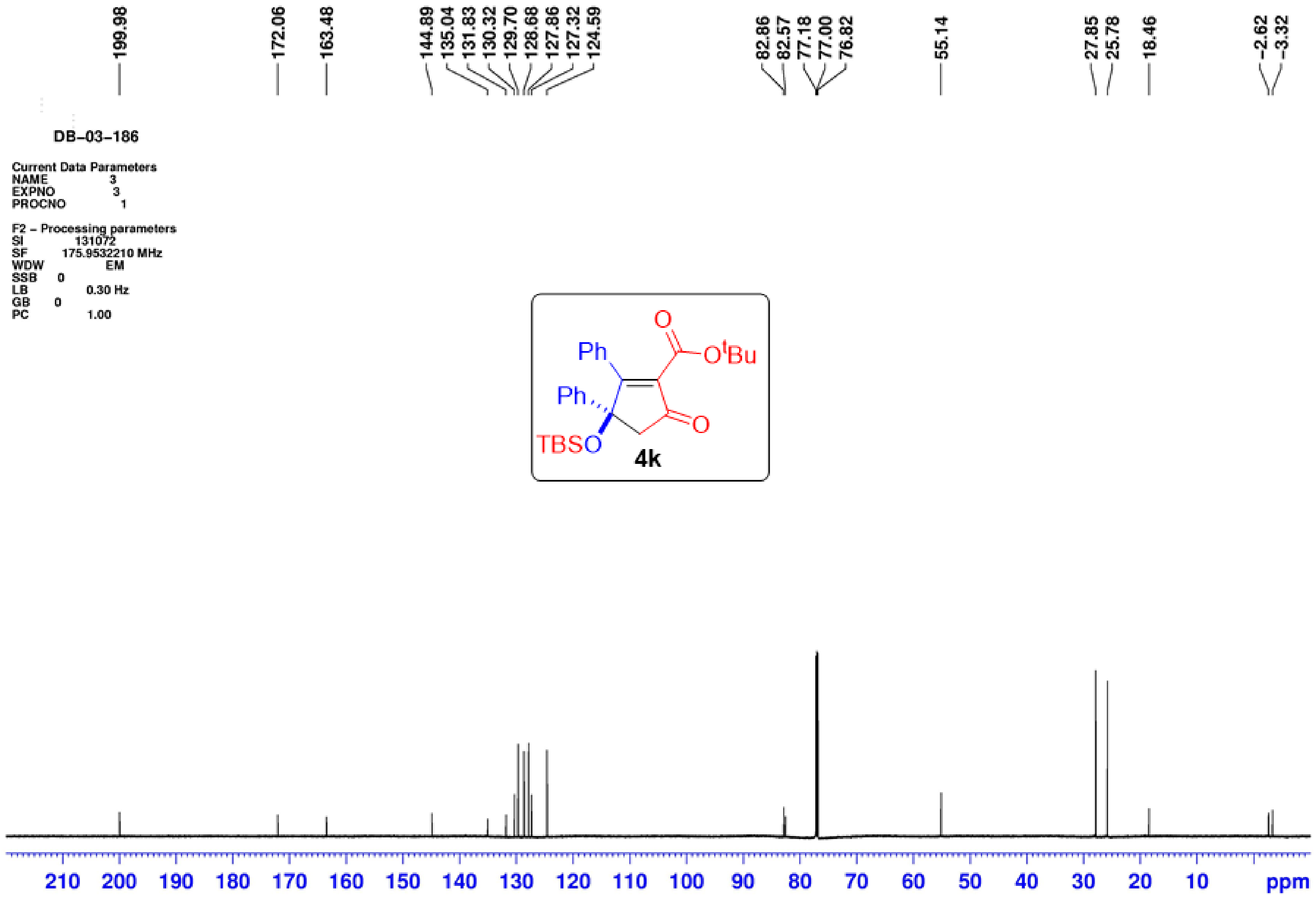

^{1}H NMR ($CDCl_3$, 700 MHz)

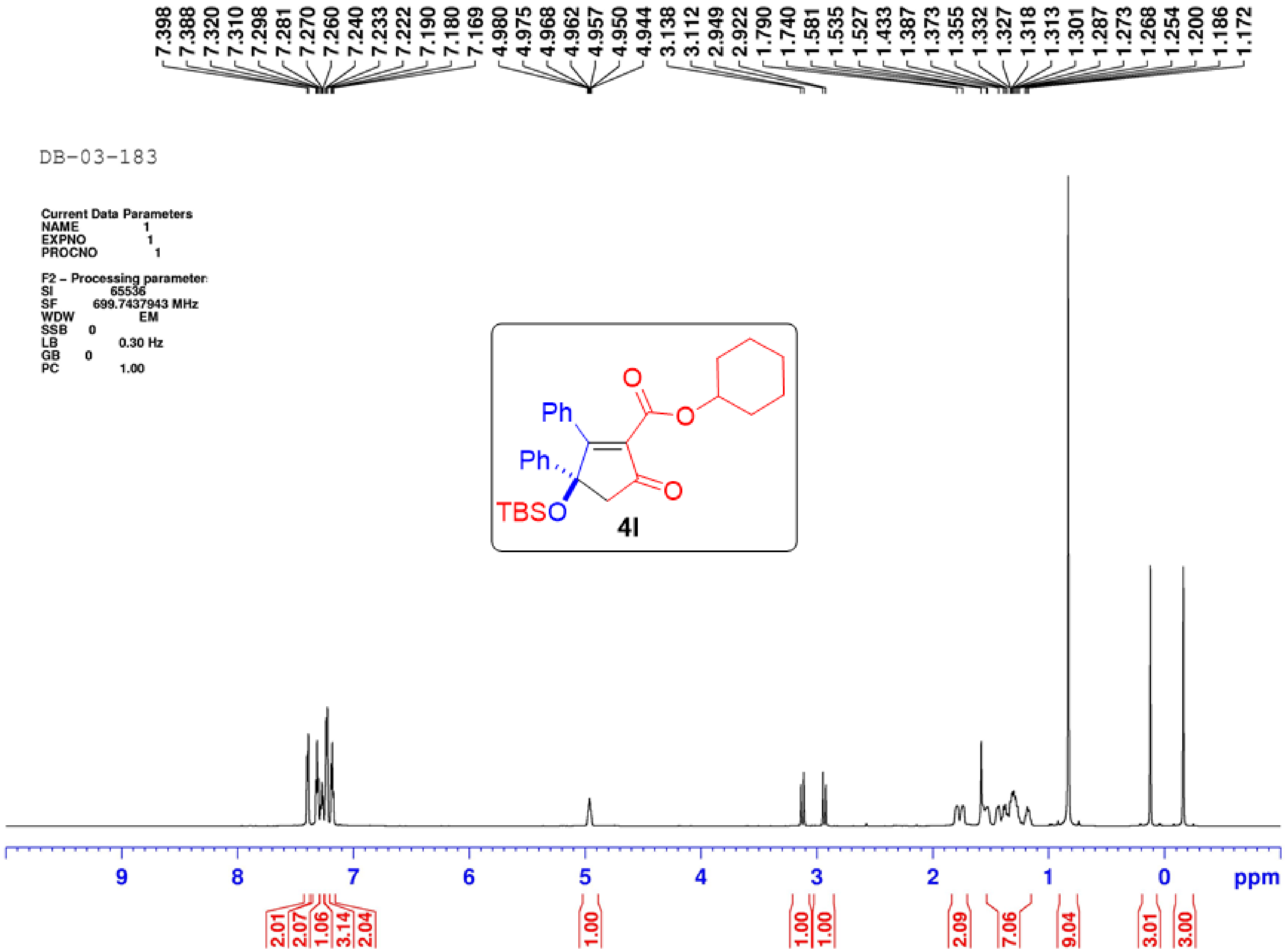

^{13}C NMR (CDCl$_3$, 175 MHz)

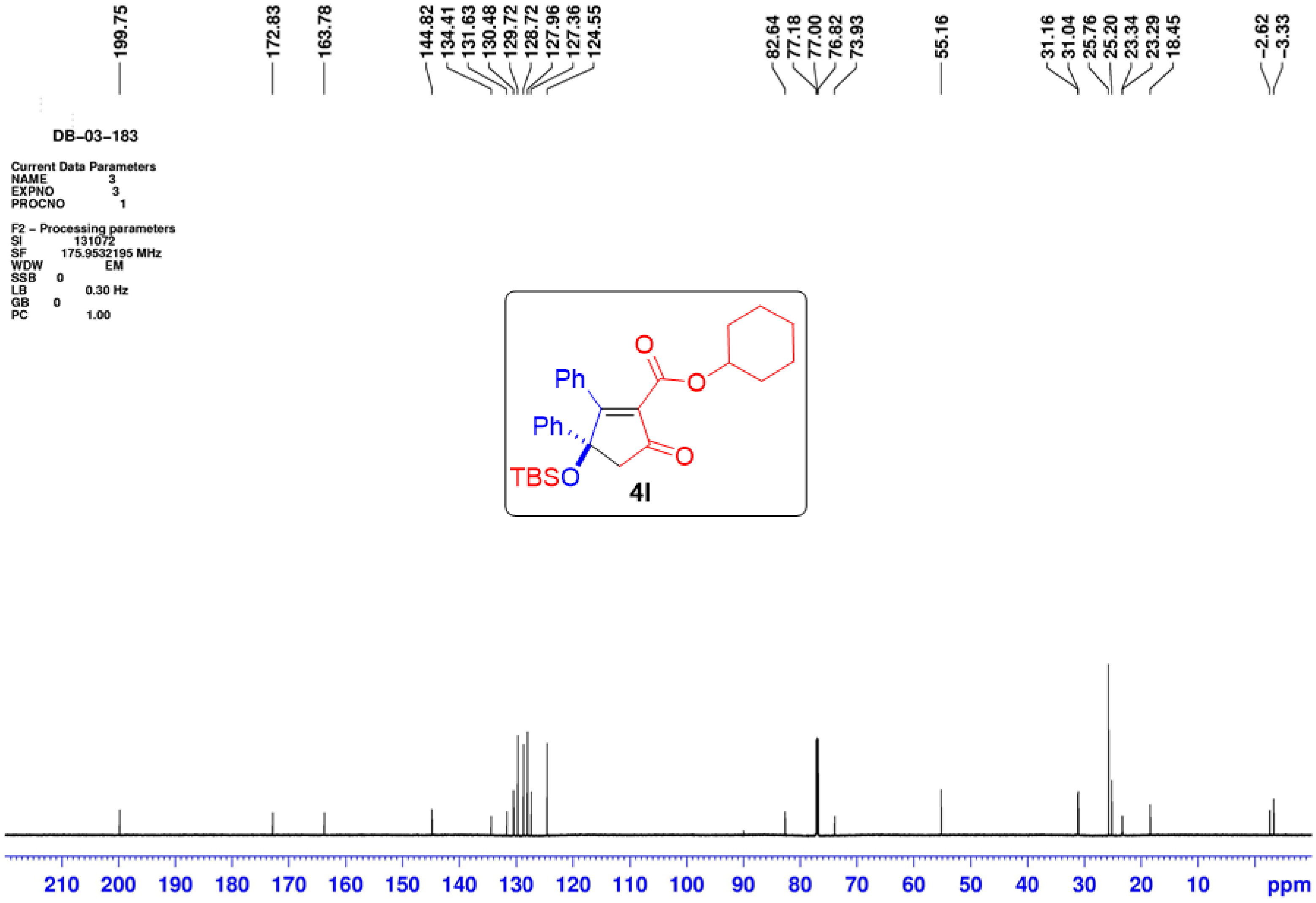

^{1}H NMR ($CDCl_3$, 700 MHz)

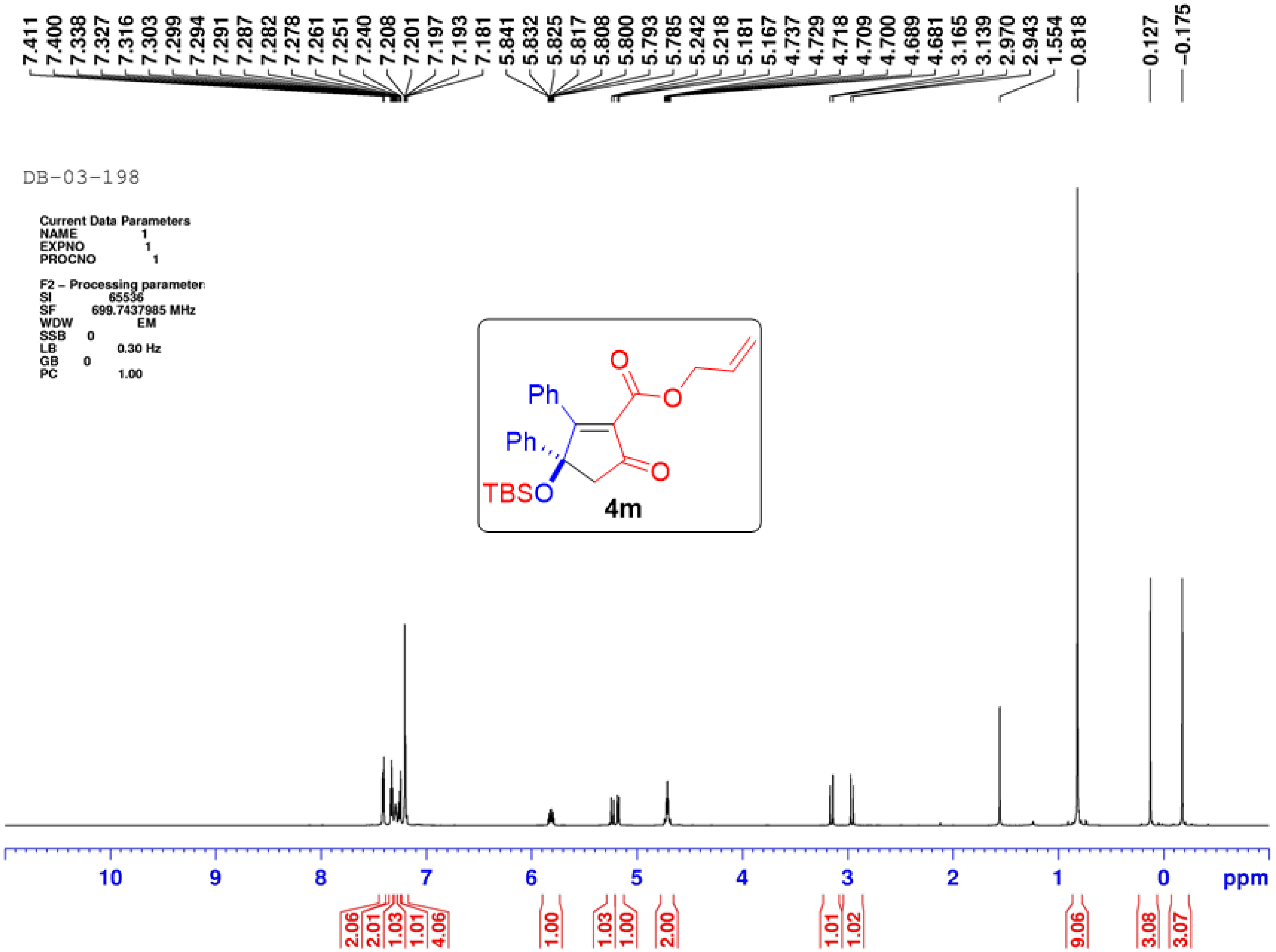

^{13}C NMR ($CDCl_3$, 175 MHz)

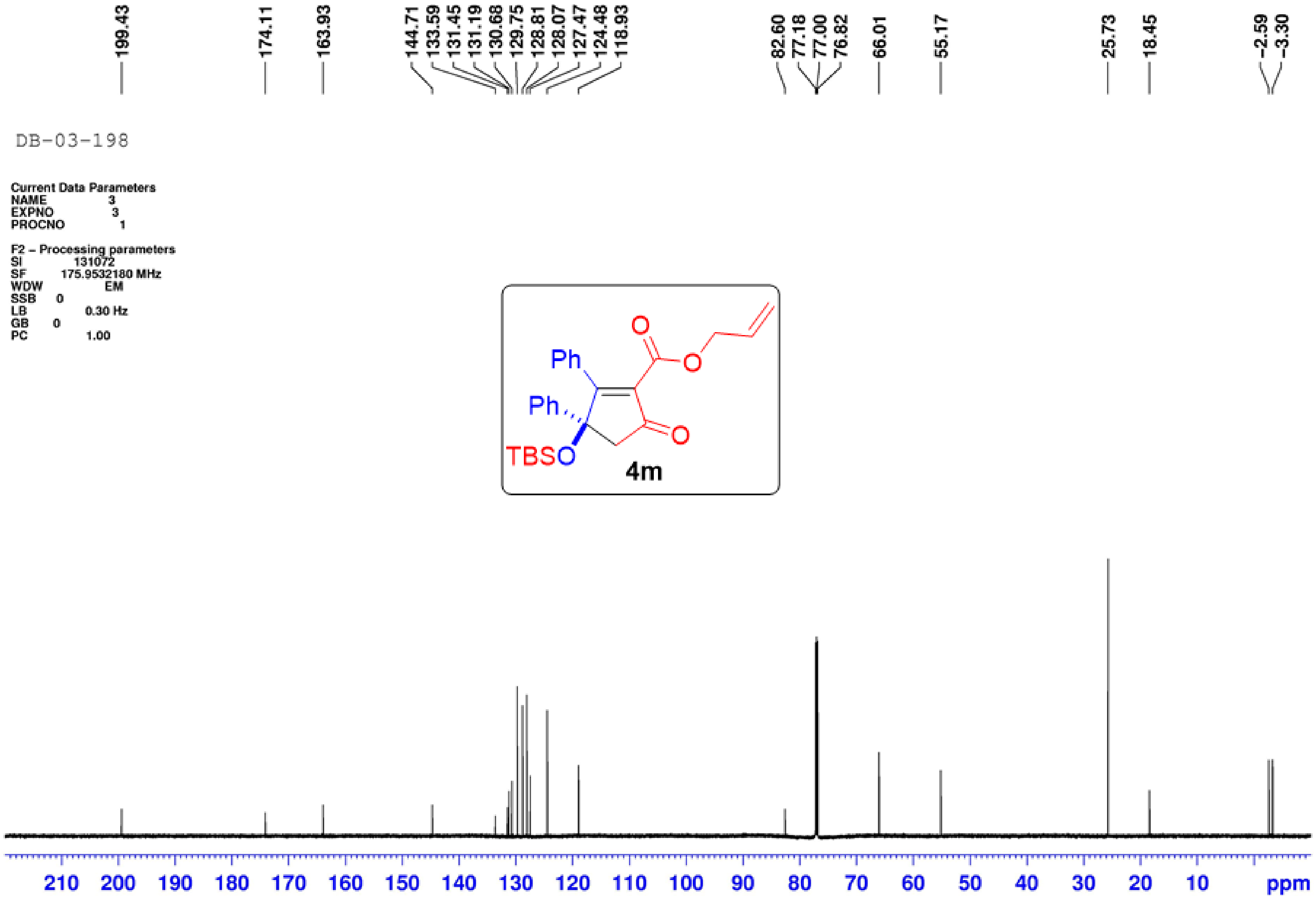

^{1}H NMR ($CDCl_3$, 700 MHz)

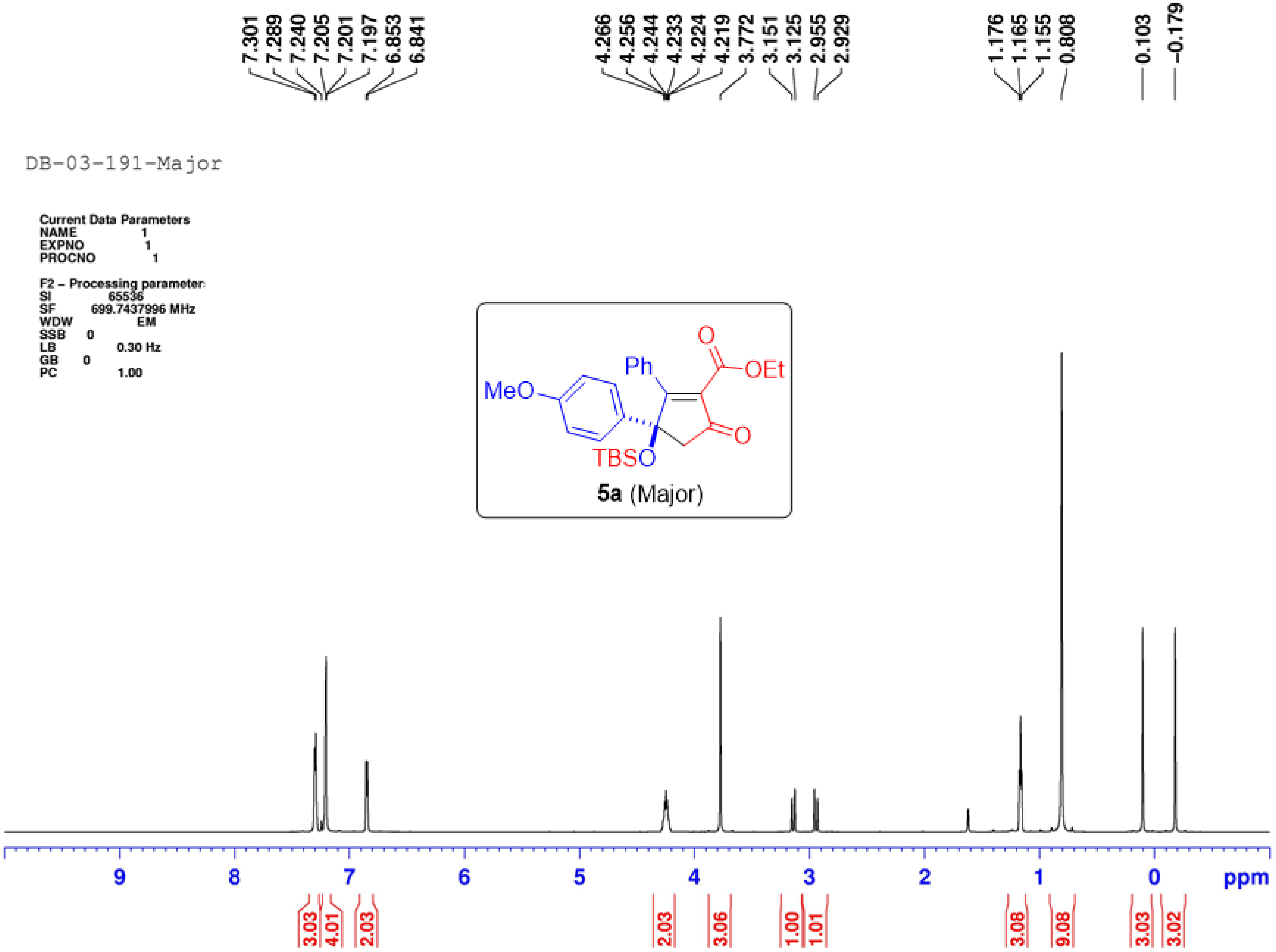

^{13}C NMR ($CDCl_3$, 175 MHz)

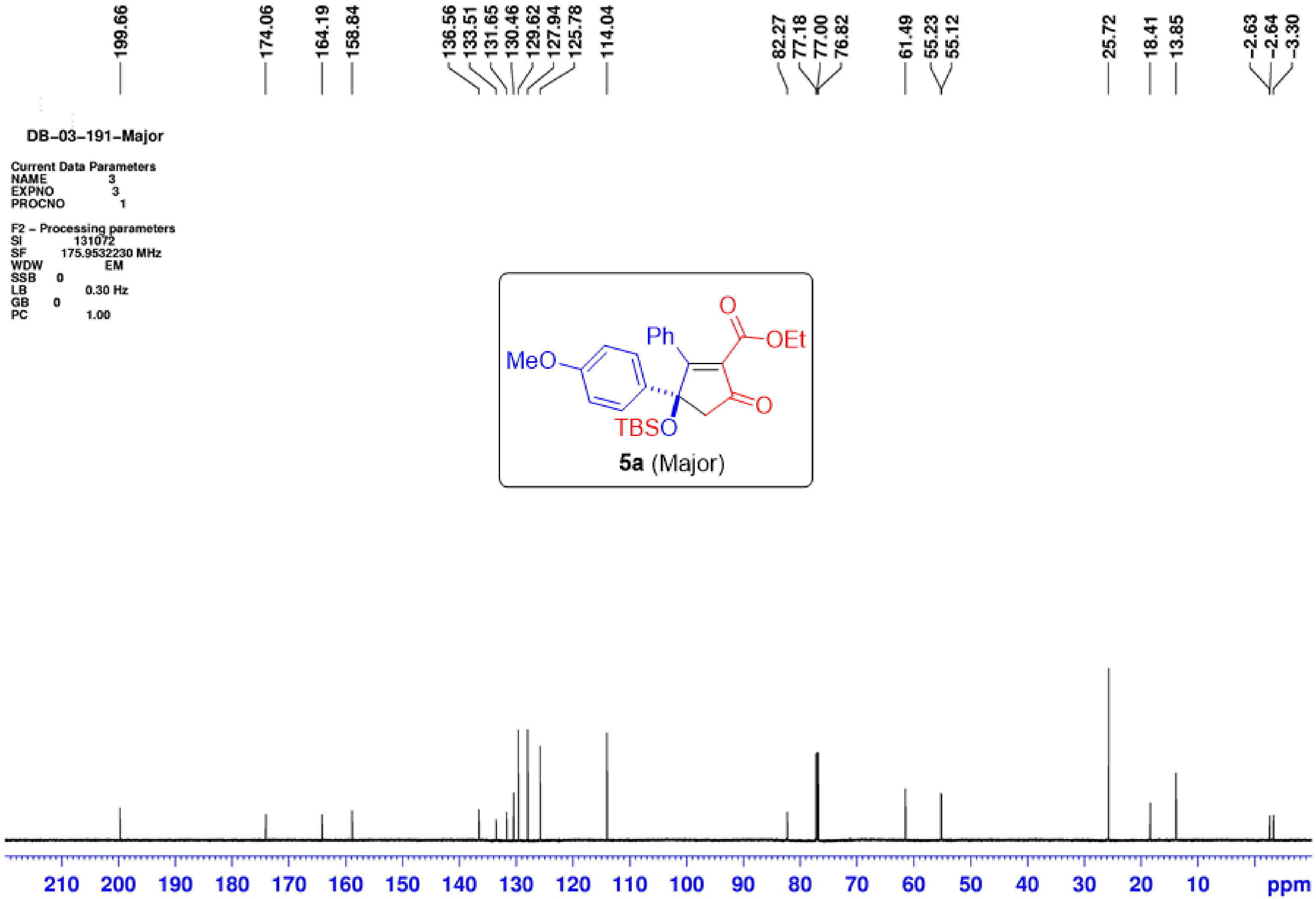

^{1}H NMR (CDCl$_3$, 700 MHz)

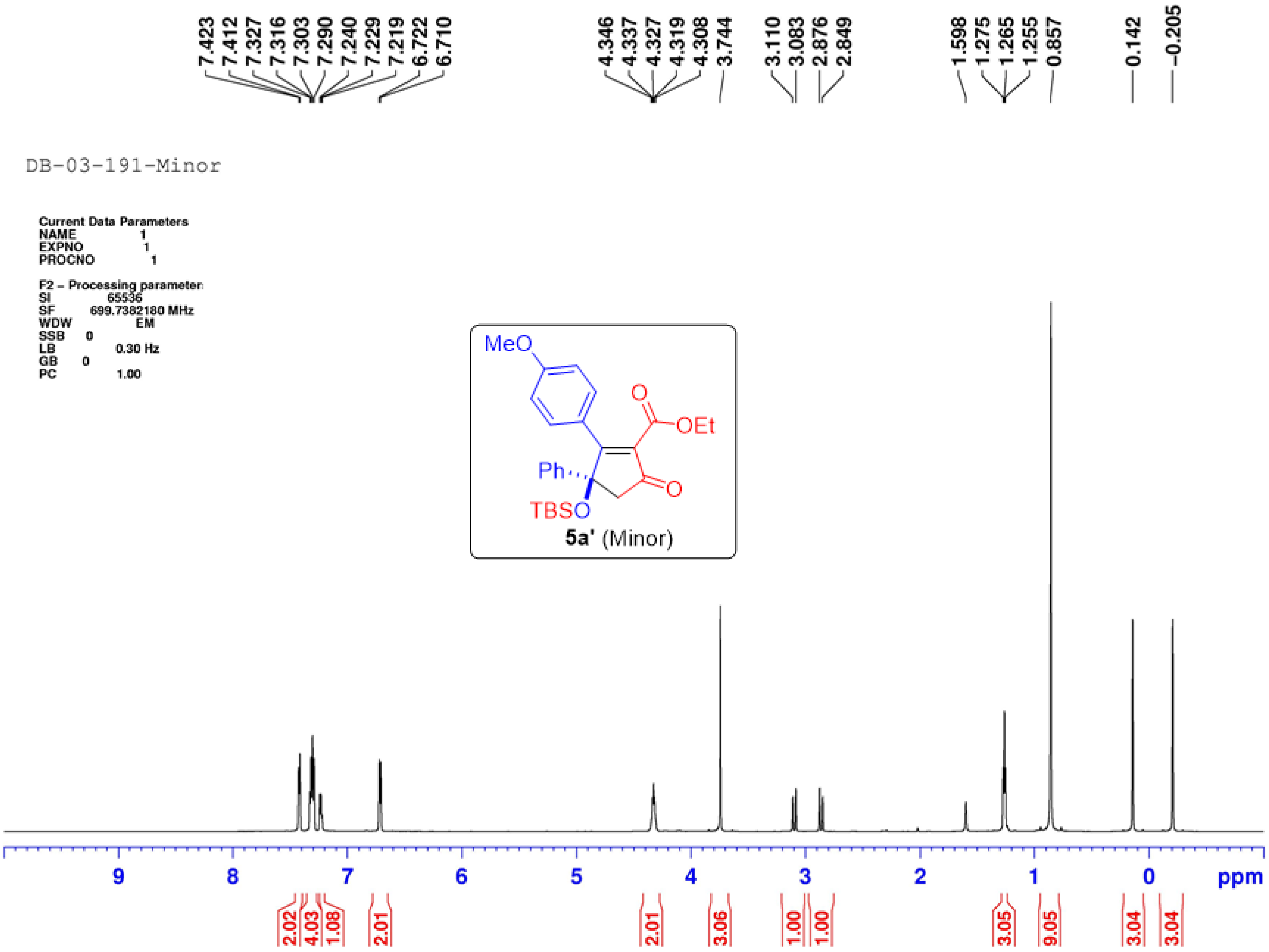

^{13}C NMR ($CDCl_3$, 175 MHz)

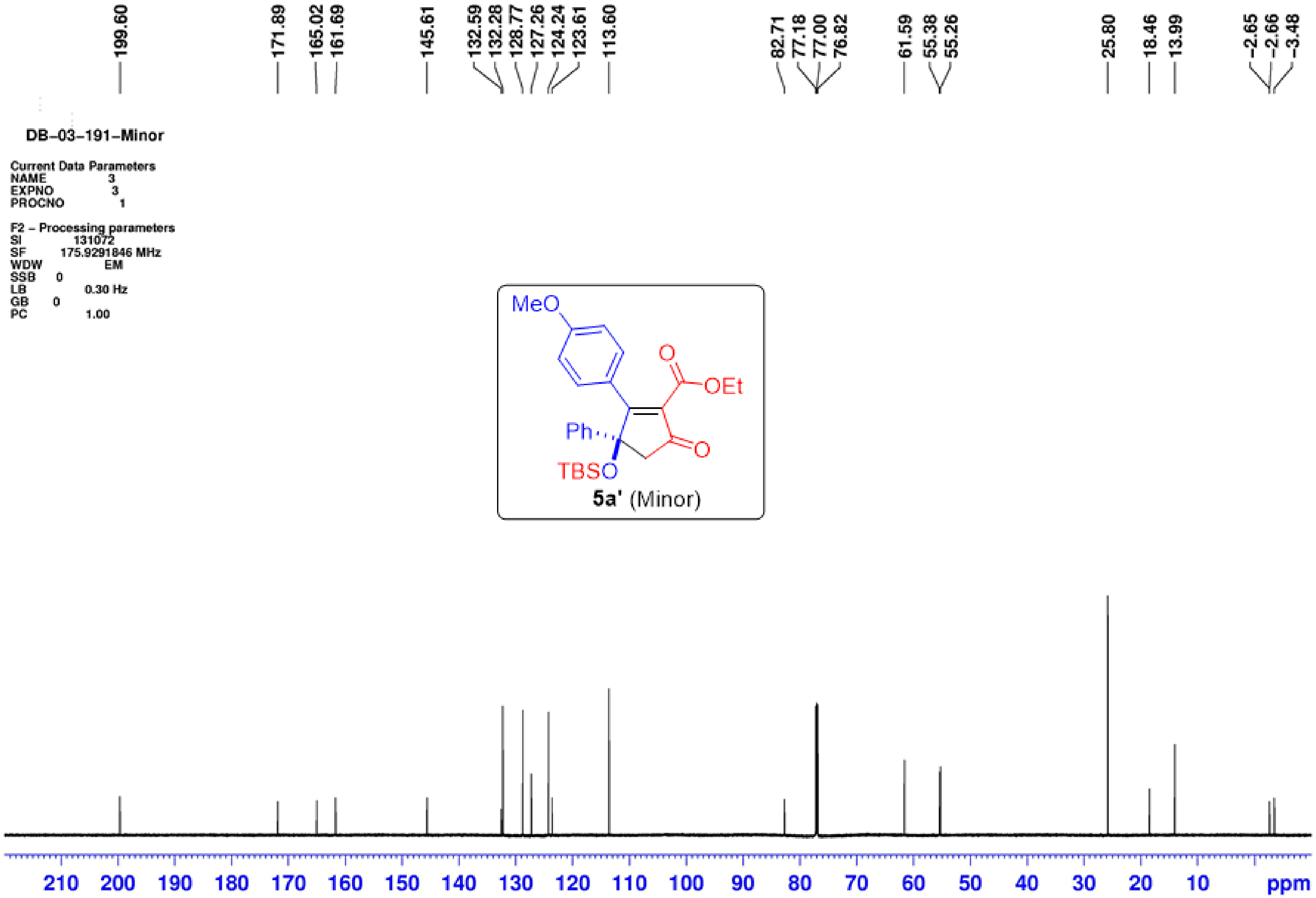

5a' (Minor)

^{1}H NMR ($CDCl_3$, 700 MHz)

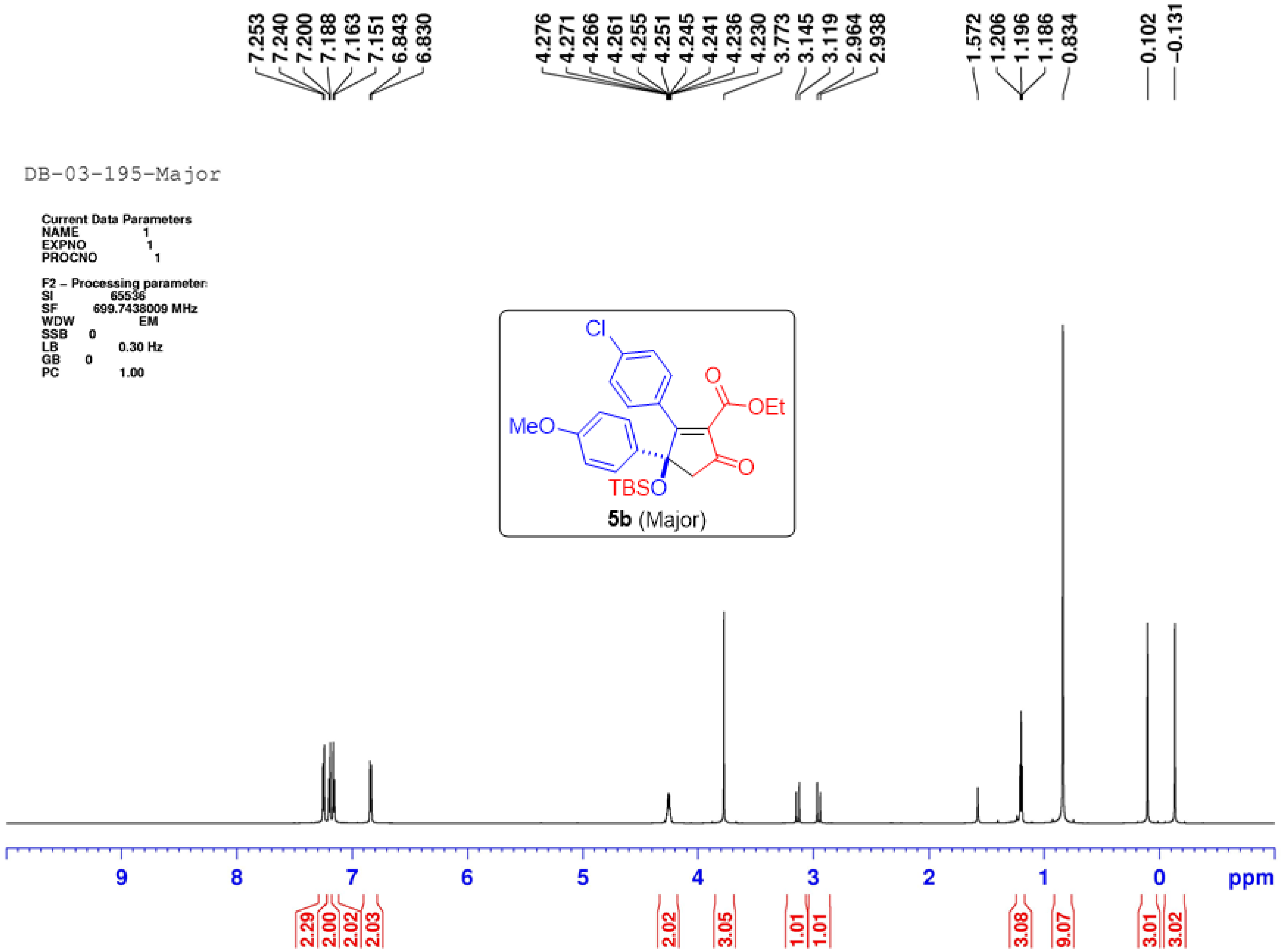

^{13}C NMR ($CDCl_3$, 175 MHz)

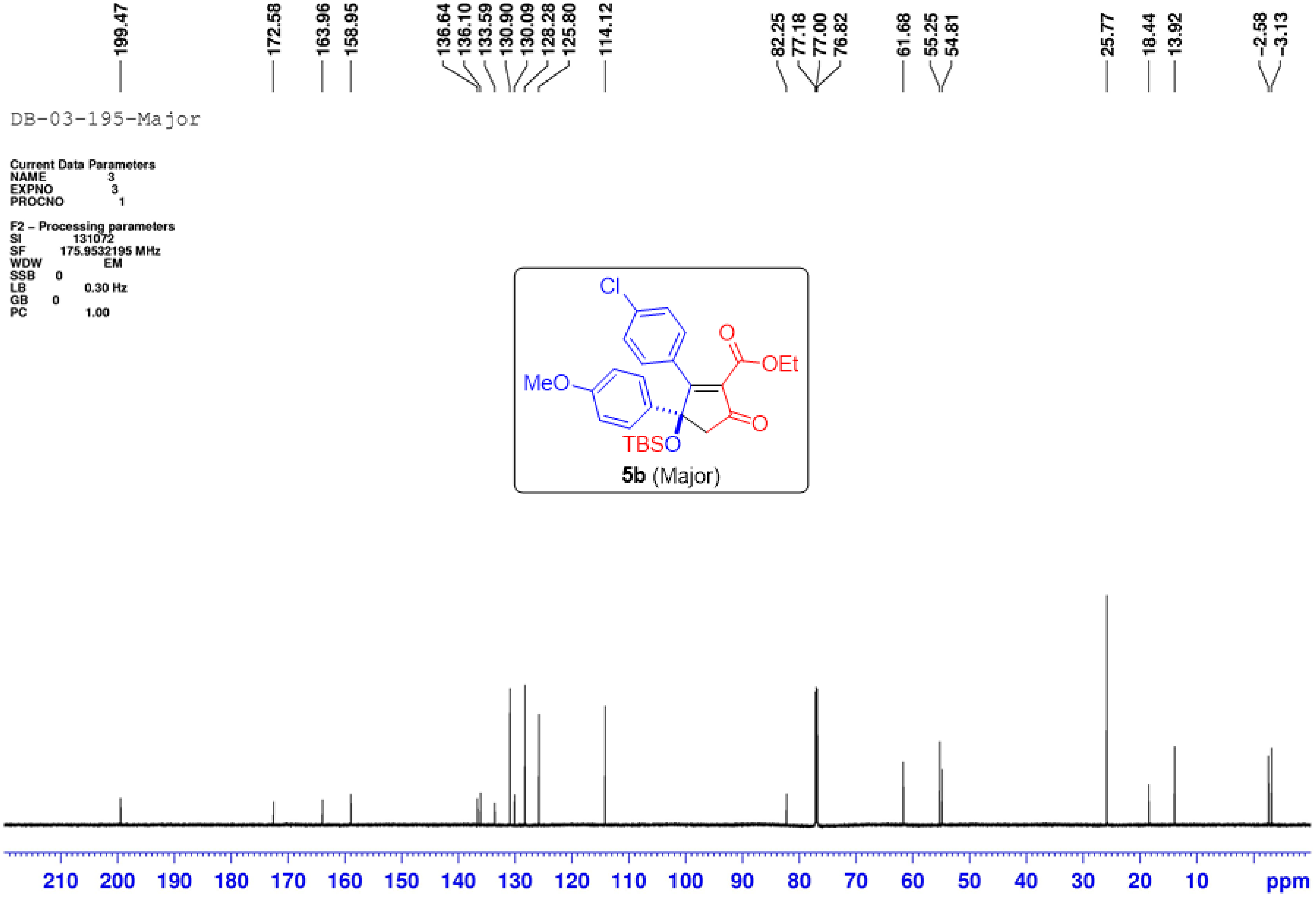

^1H NMR ($CDCl_3$, 700 MHz)

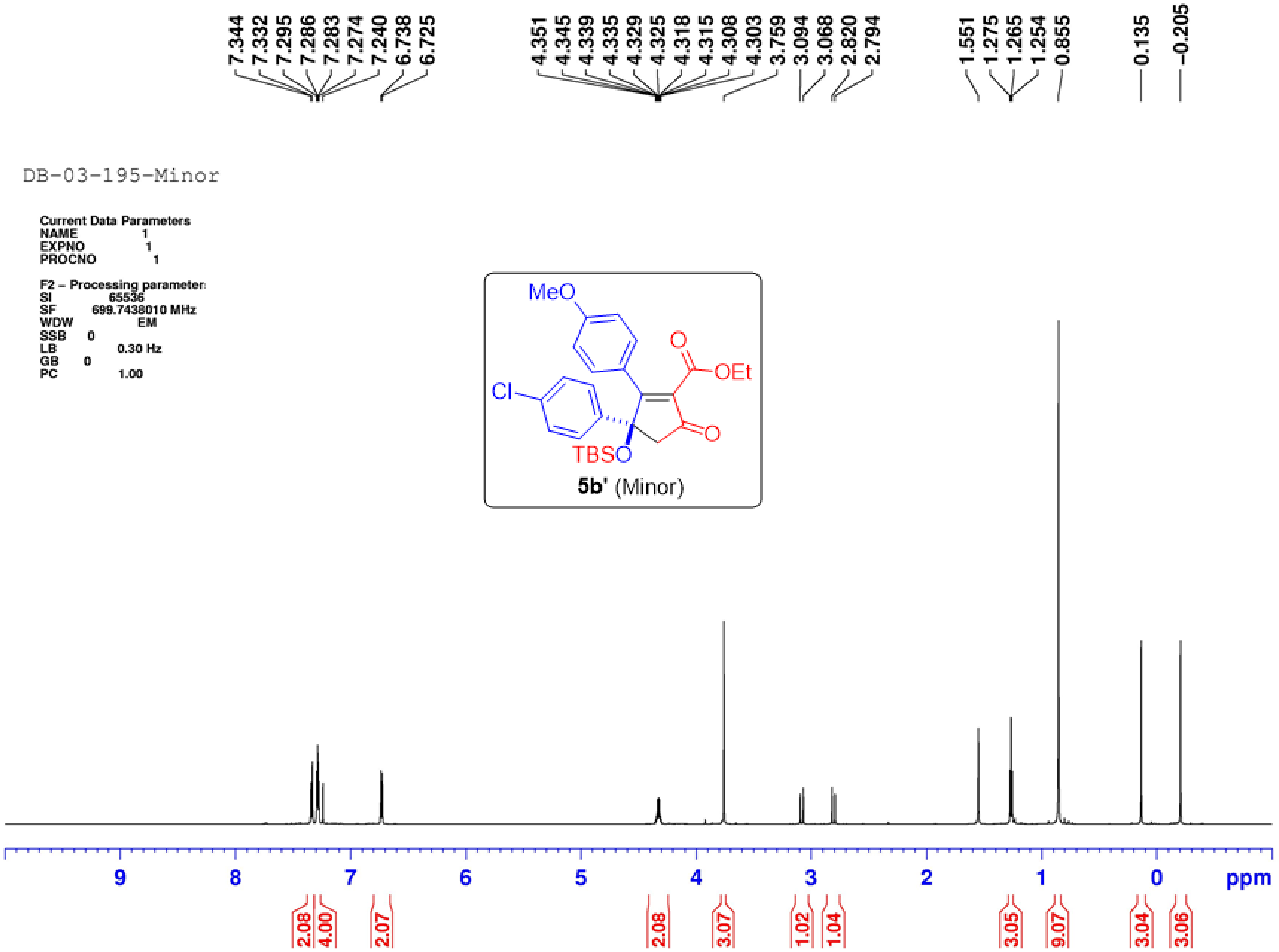

^{13}C NMR ($CDCl_3$, 175 MHz)

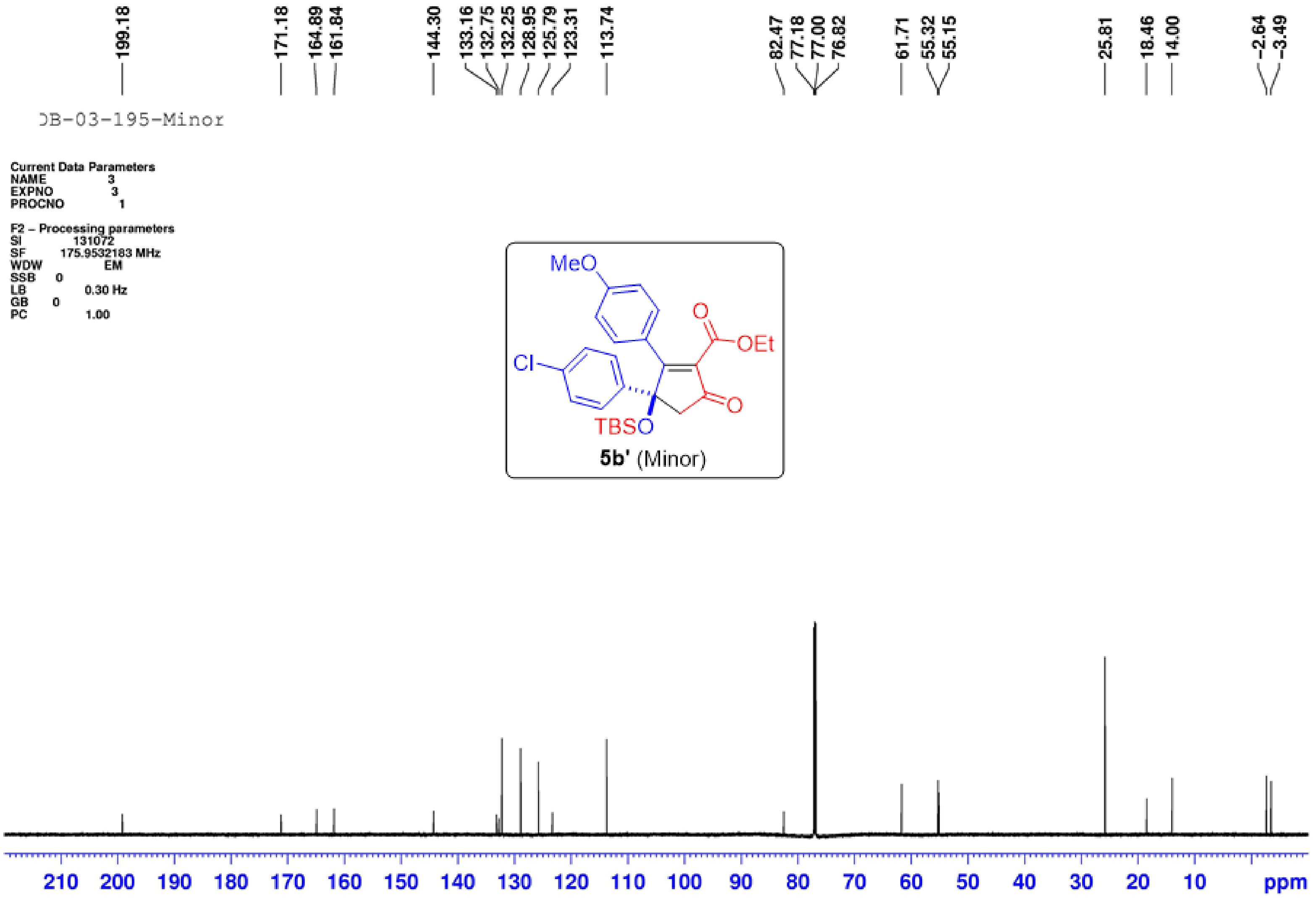

^{1}H NMR ($CDCl_3$, 700 MHz)

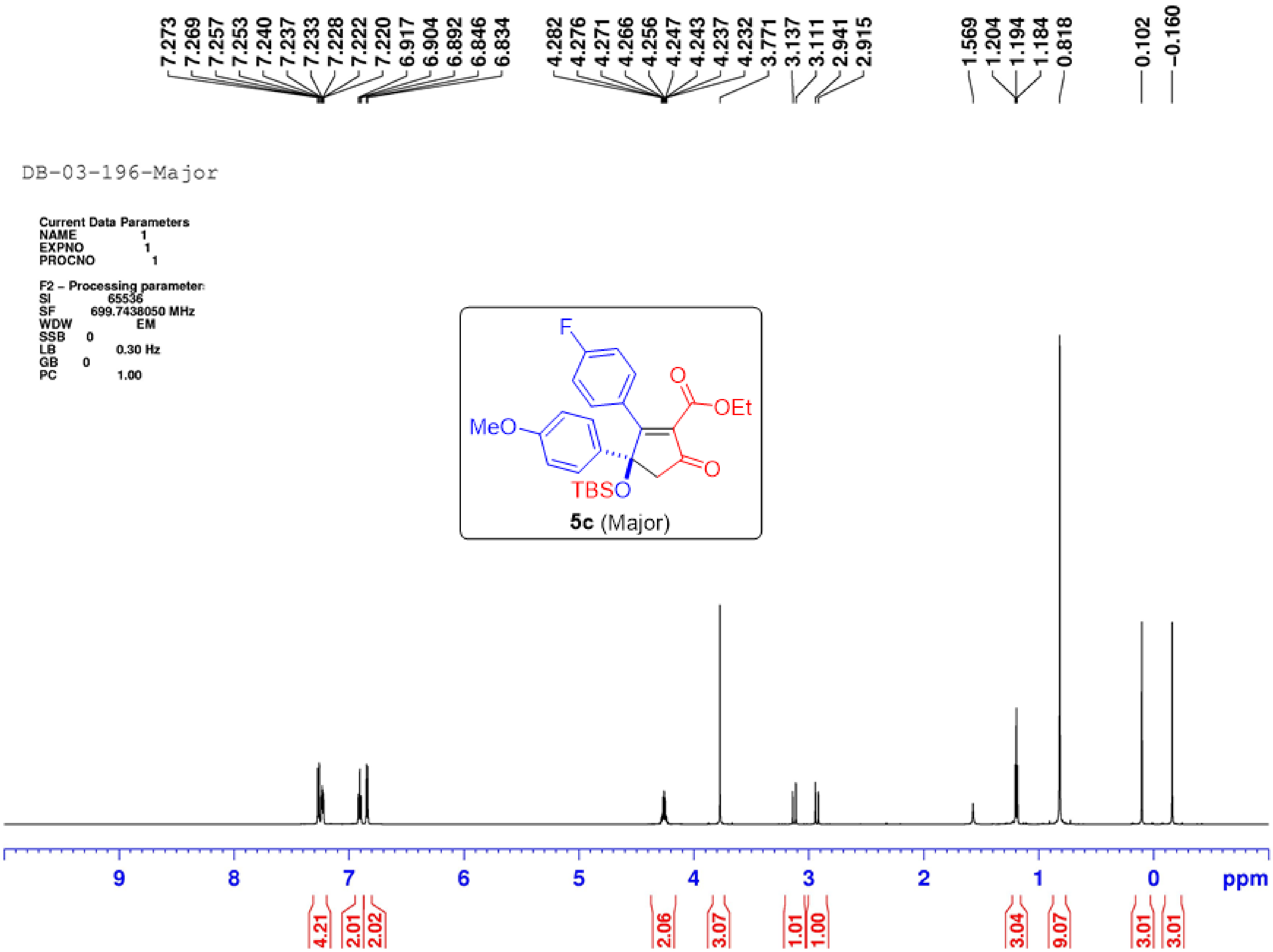

^{13}C NMR ($CDCl_3$, 175 MHz)

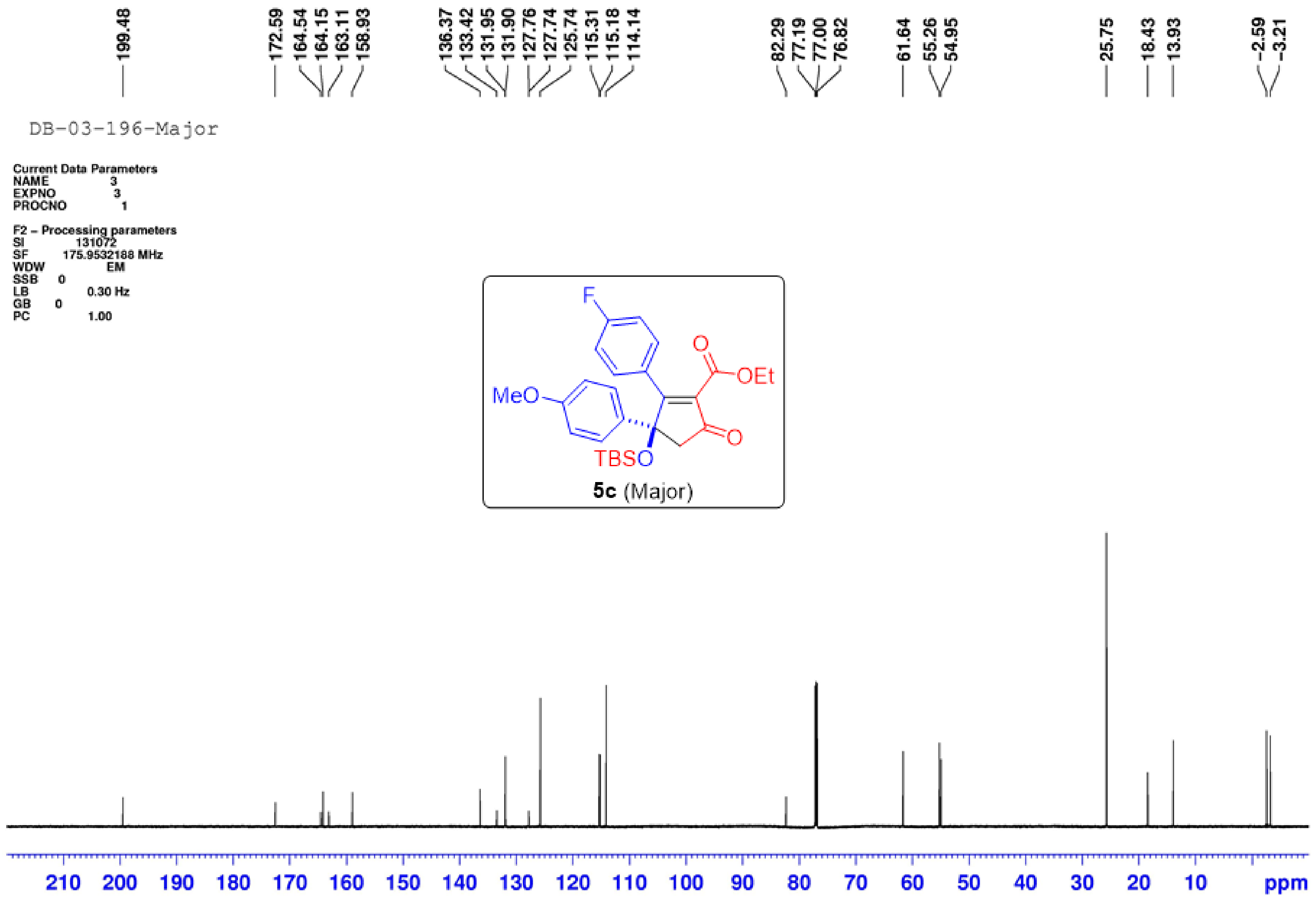

^{19}F NMR (CDCl$_3$, 471 MHz)

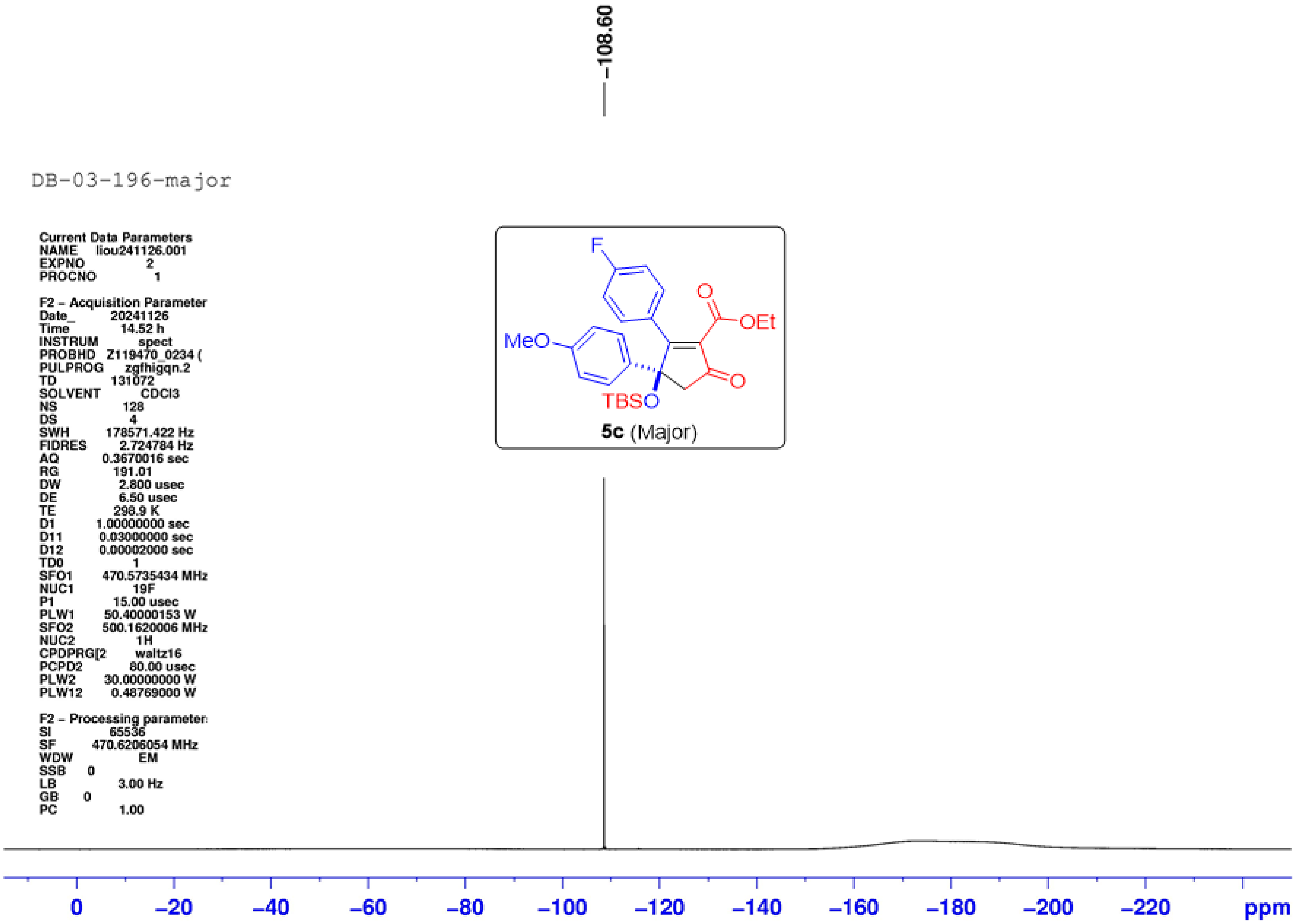

^{1}H NMR ($CDCl_3$, 700 MHz)

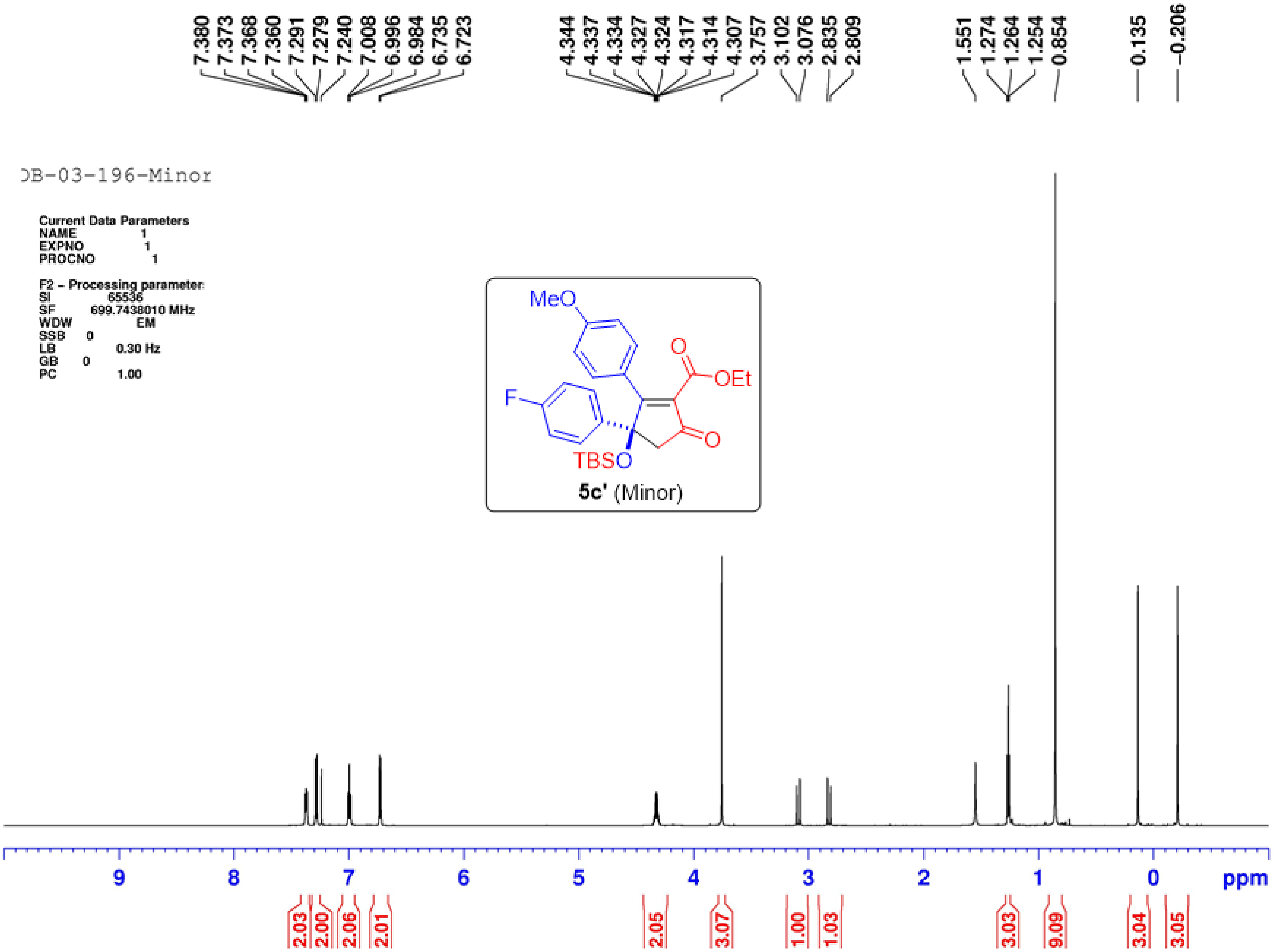

5c' (Minor)

^{13}C NMR (CDCl$_3$, 175 MHz)

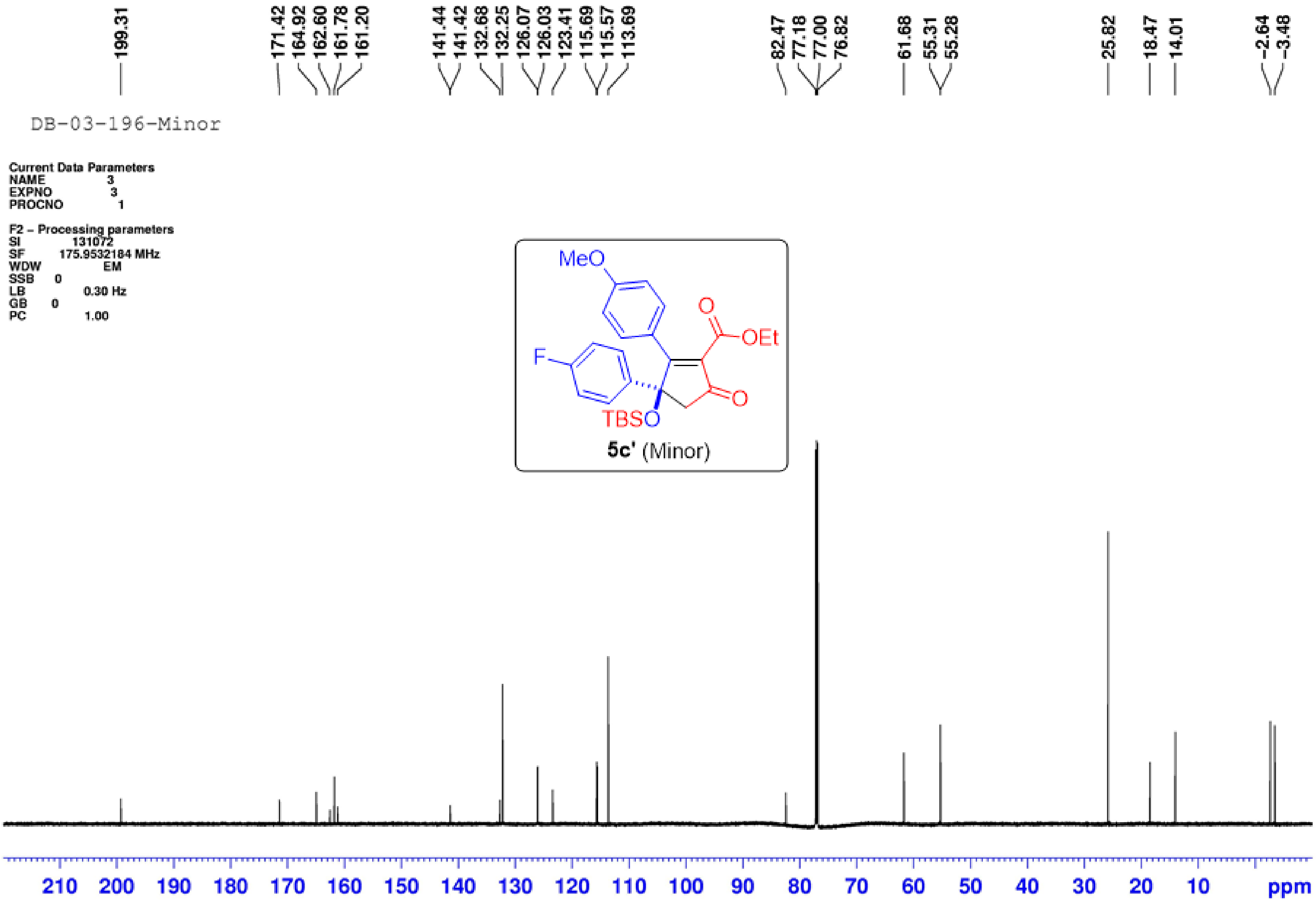

^{19}F NMR (CDCl$_3$, 471 MHz)

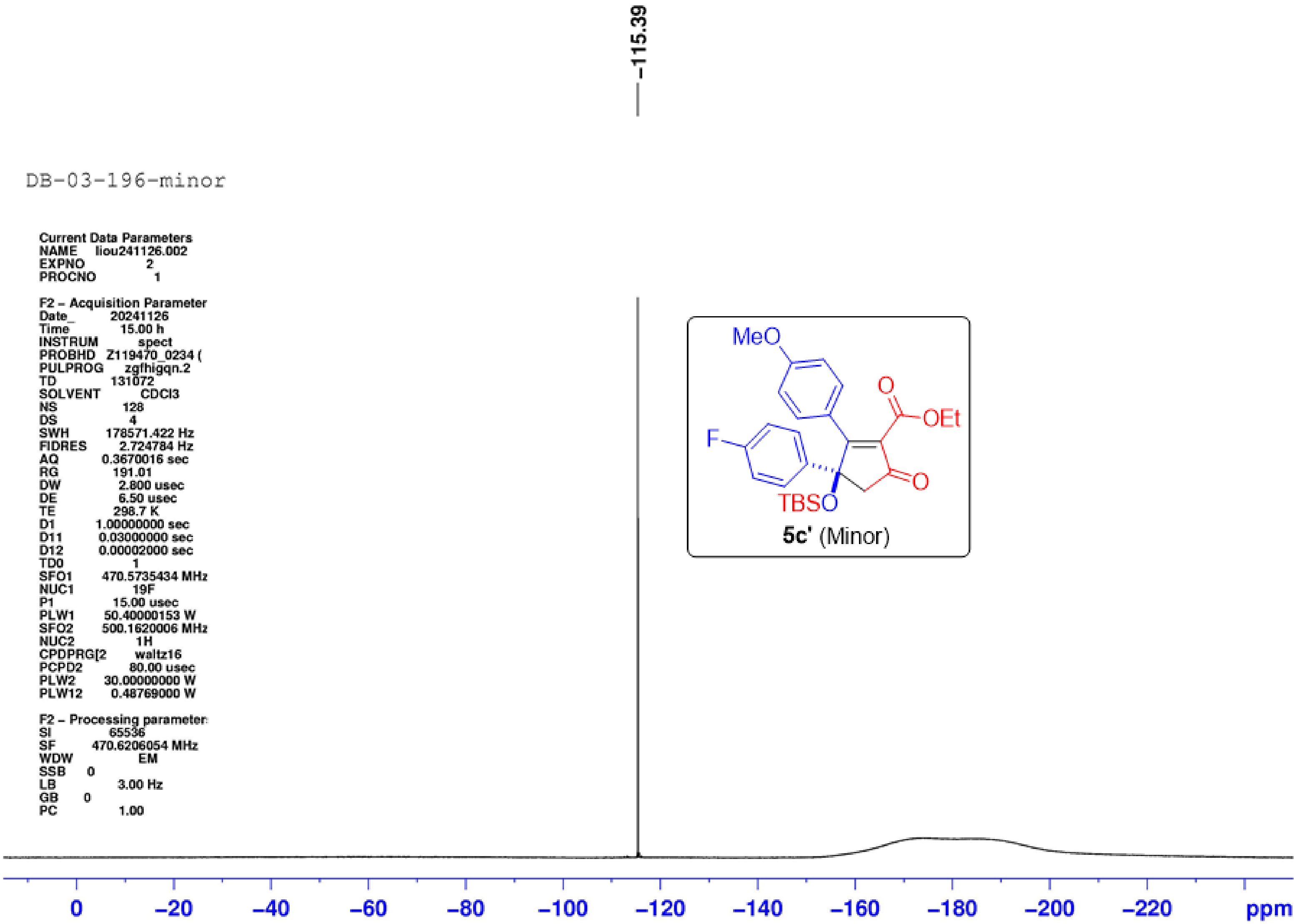

^{1}H NOE NMR (700 MHz)

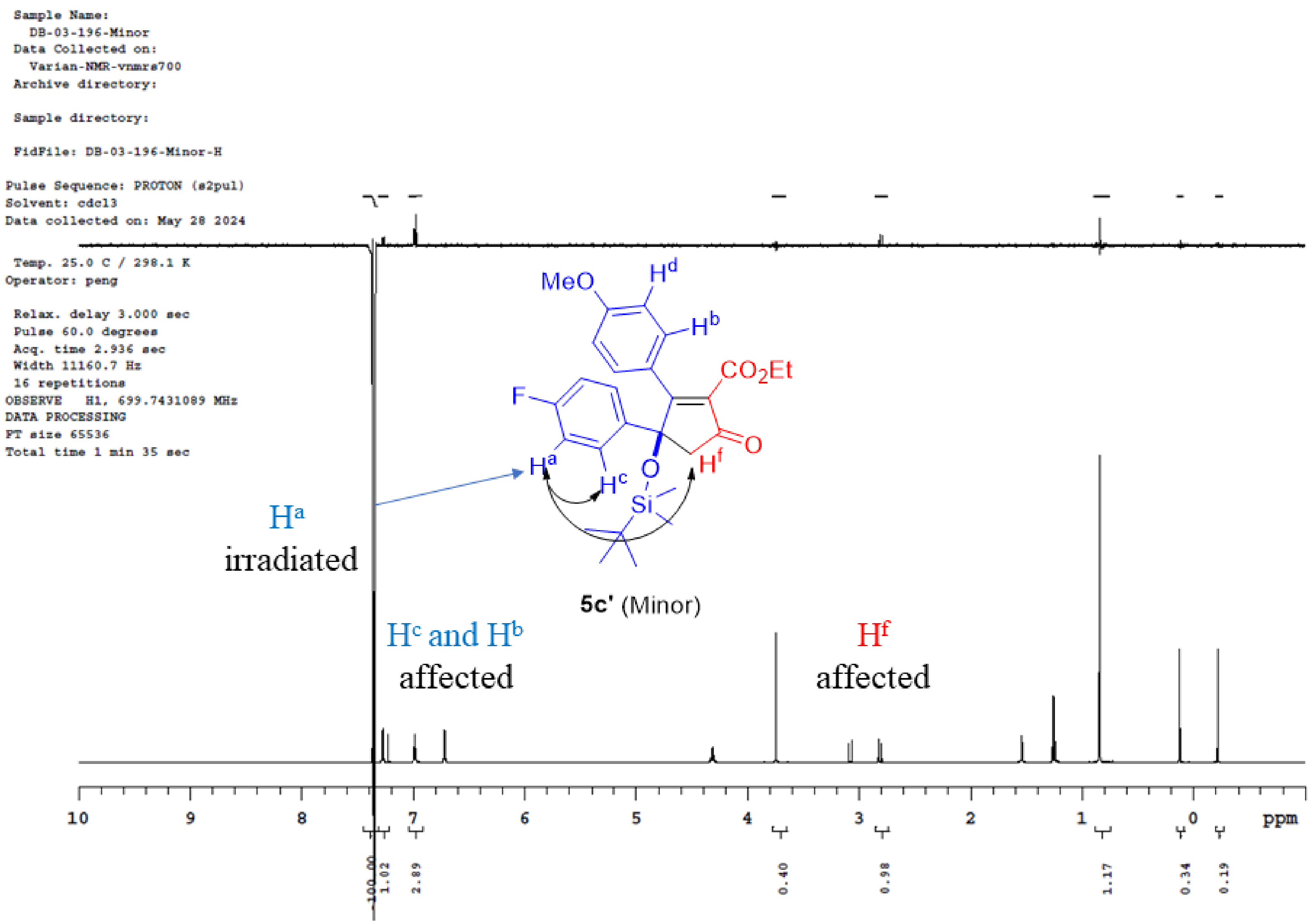

^{1}H NOE NMR (700 MHz)

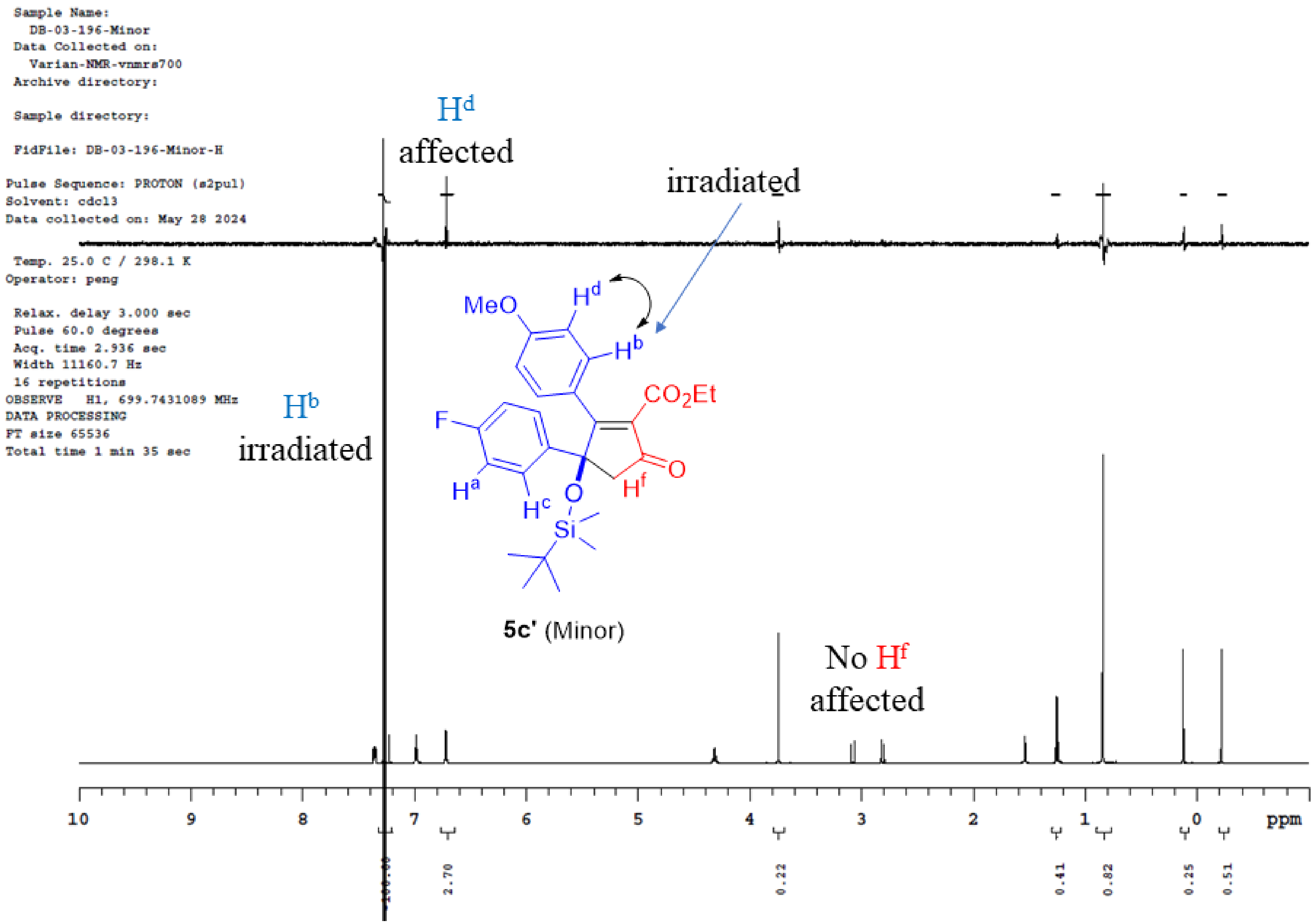

^{1}H NOE NMR (700 MHz)

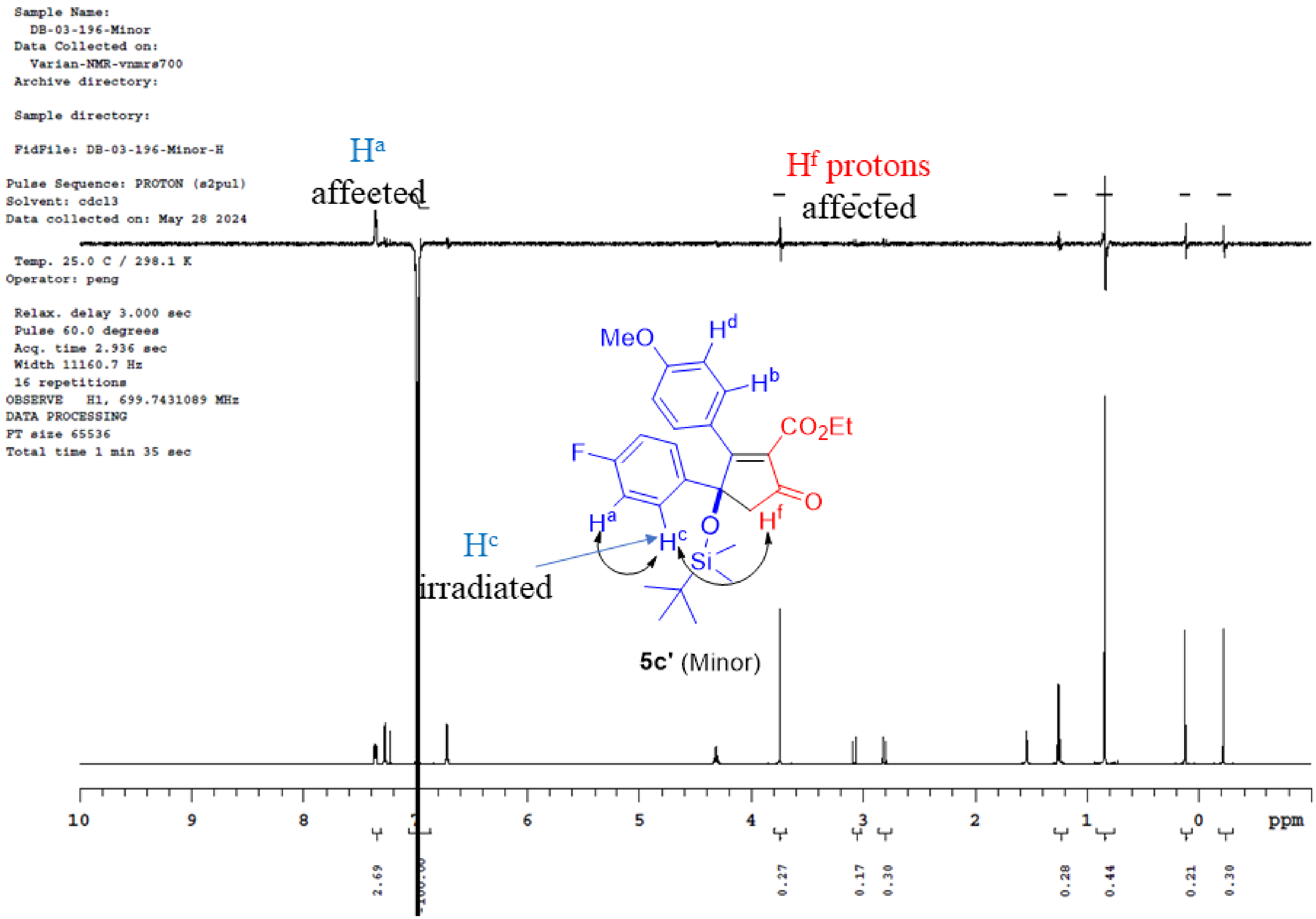

^{1}H NOE NMR (700 MHz)

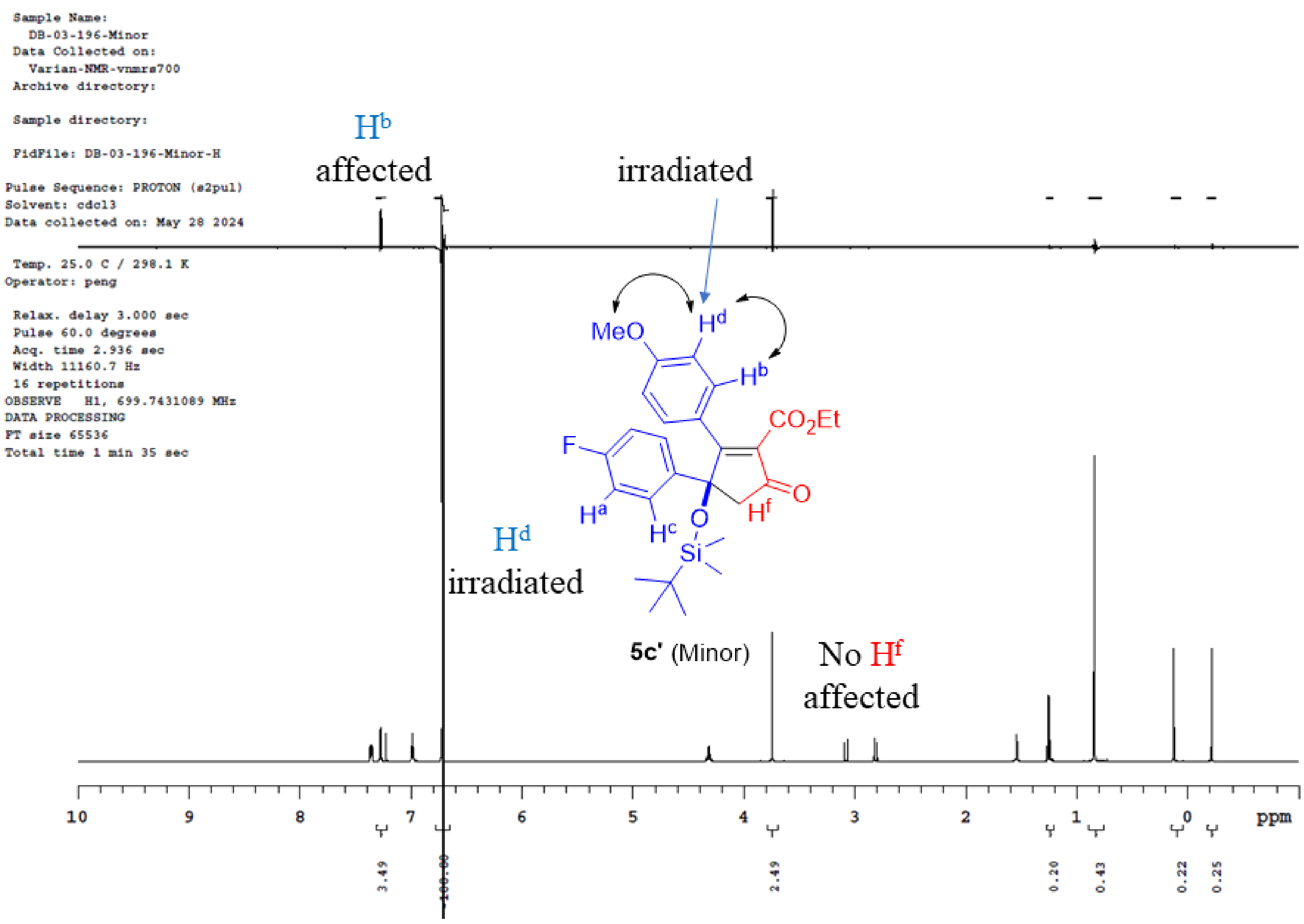

^{1}H NMR ($CDCl_3$, 700 MHz)

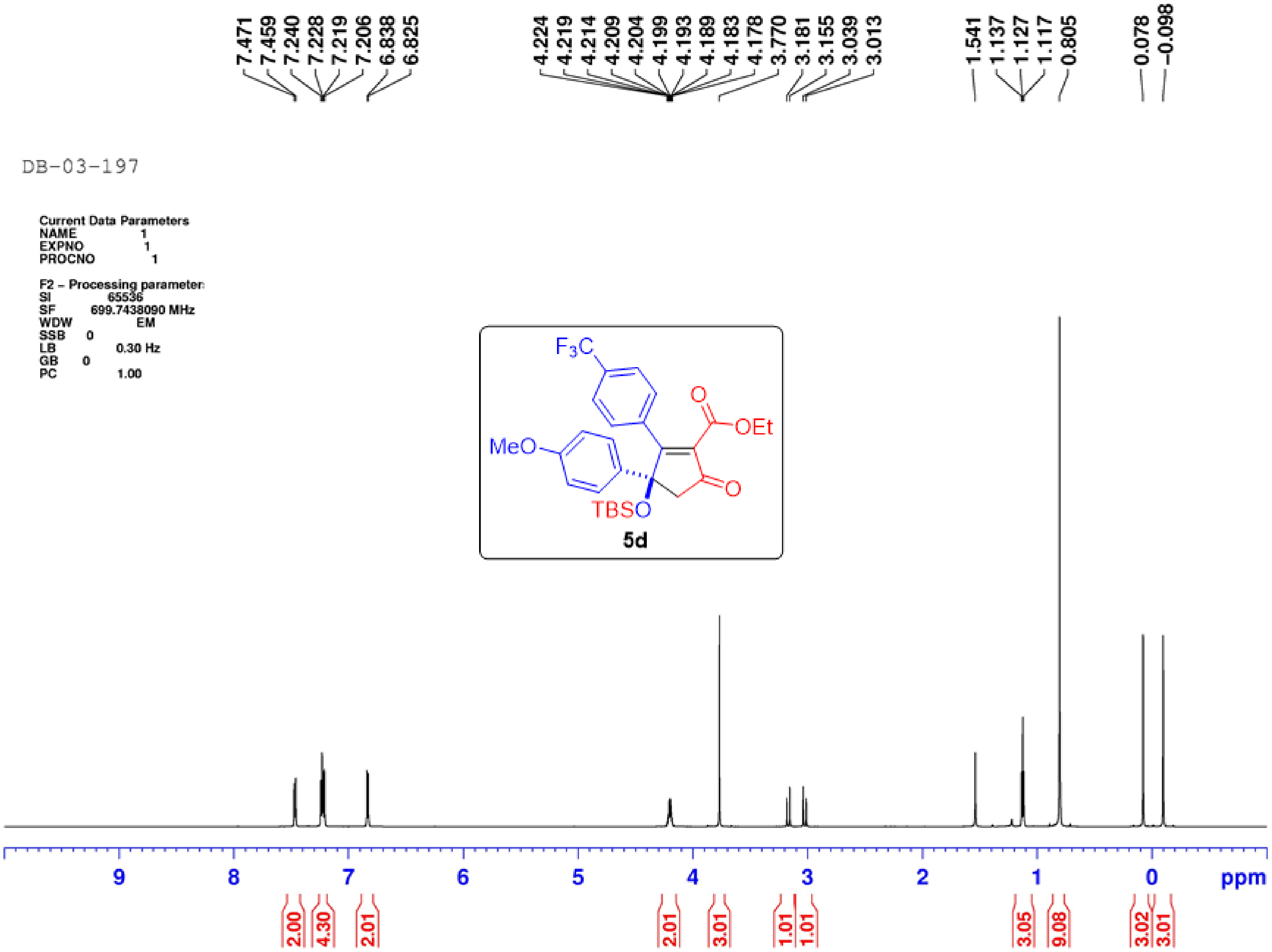

^{13}C NMR ($CDCl_3$, 175 MHz)

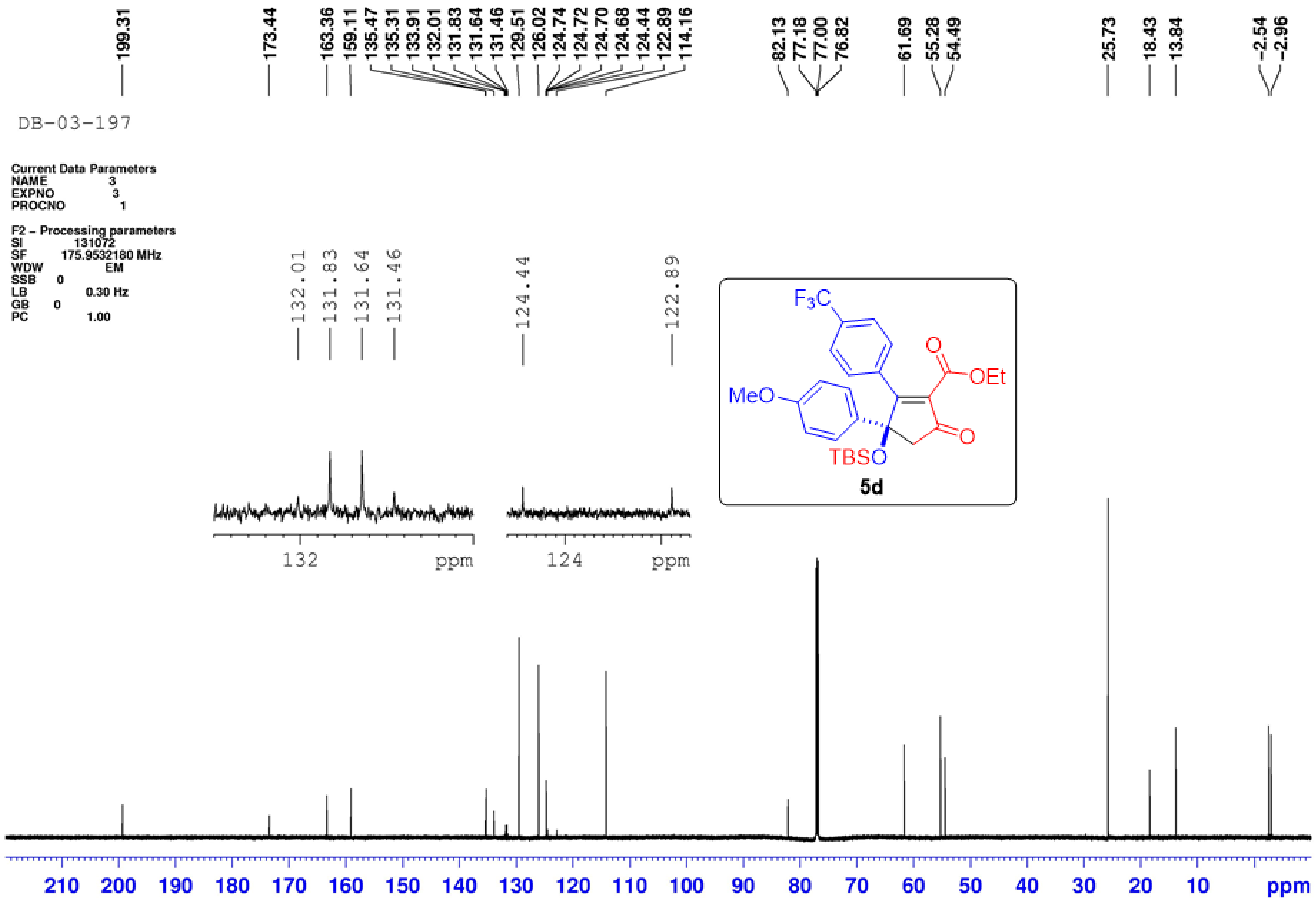

^{19}F NMR (CDCl$_3$, 471 MHz)

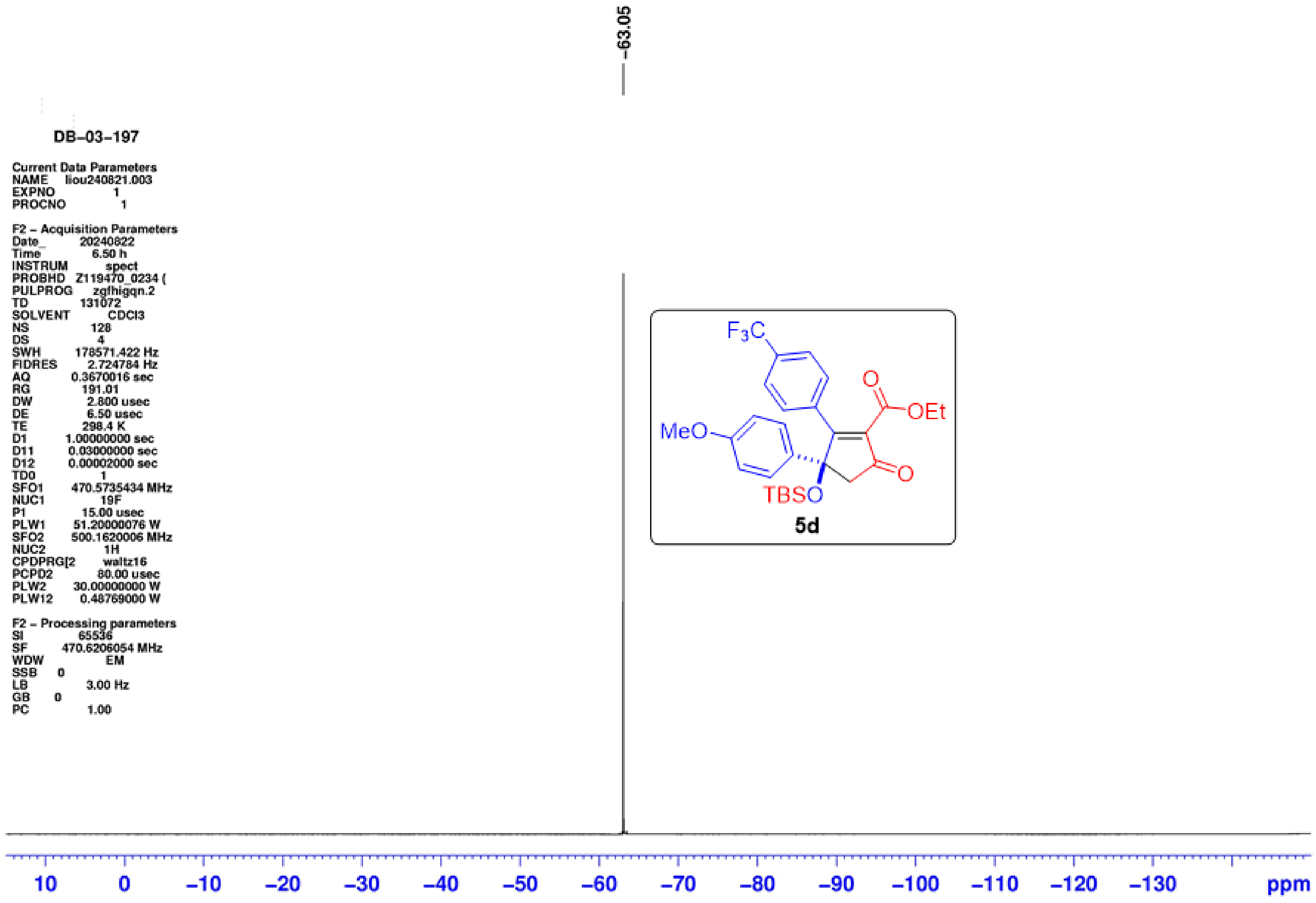

^{1}H NMR ($CDCl_3$, 700 MHz)

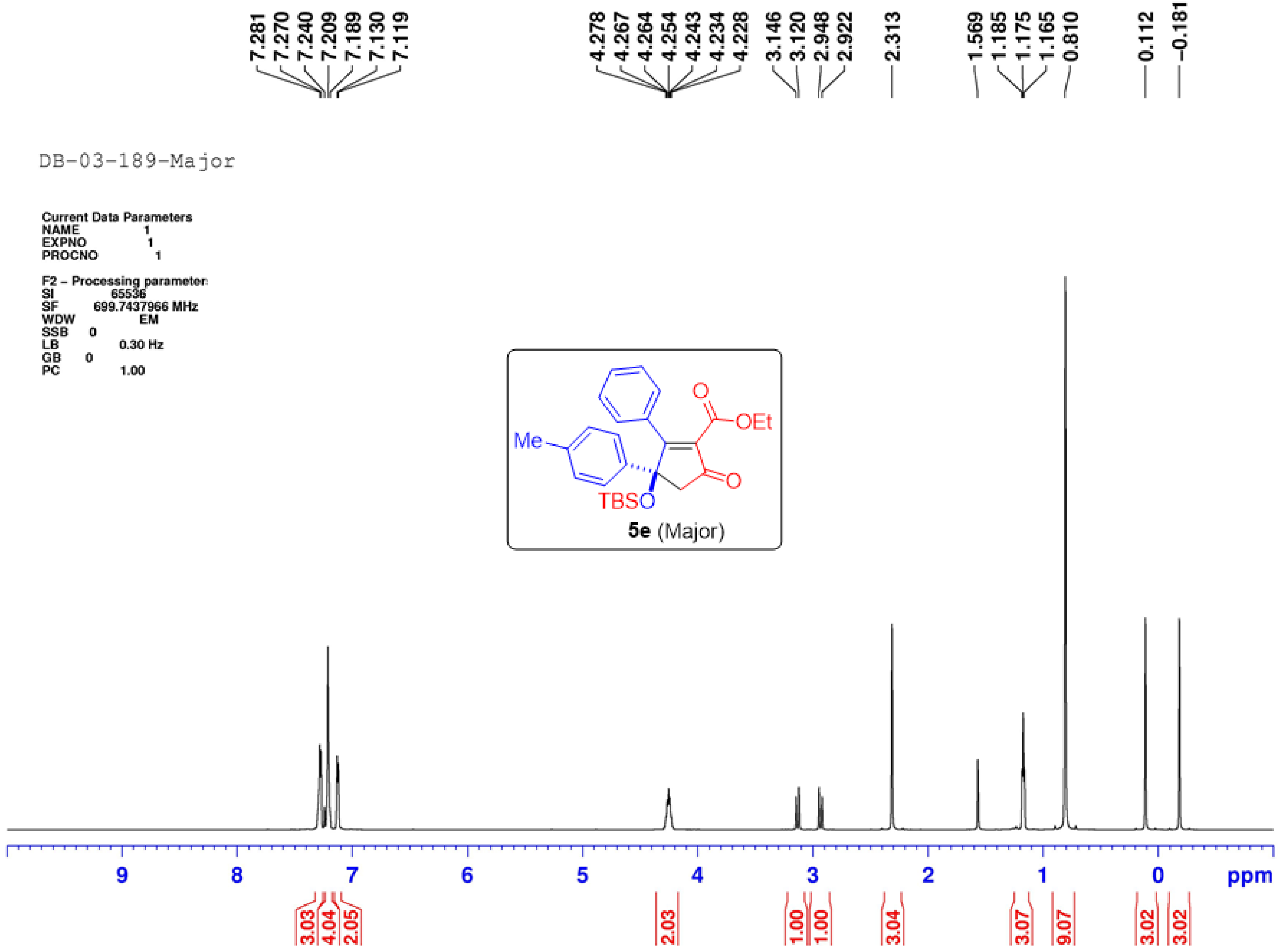

^{13}C NMR ($CDCl_3$, 175 MHz)

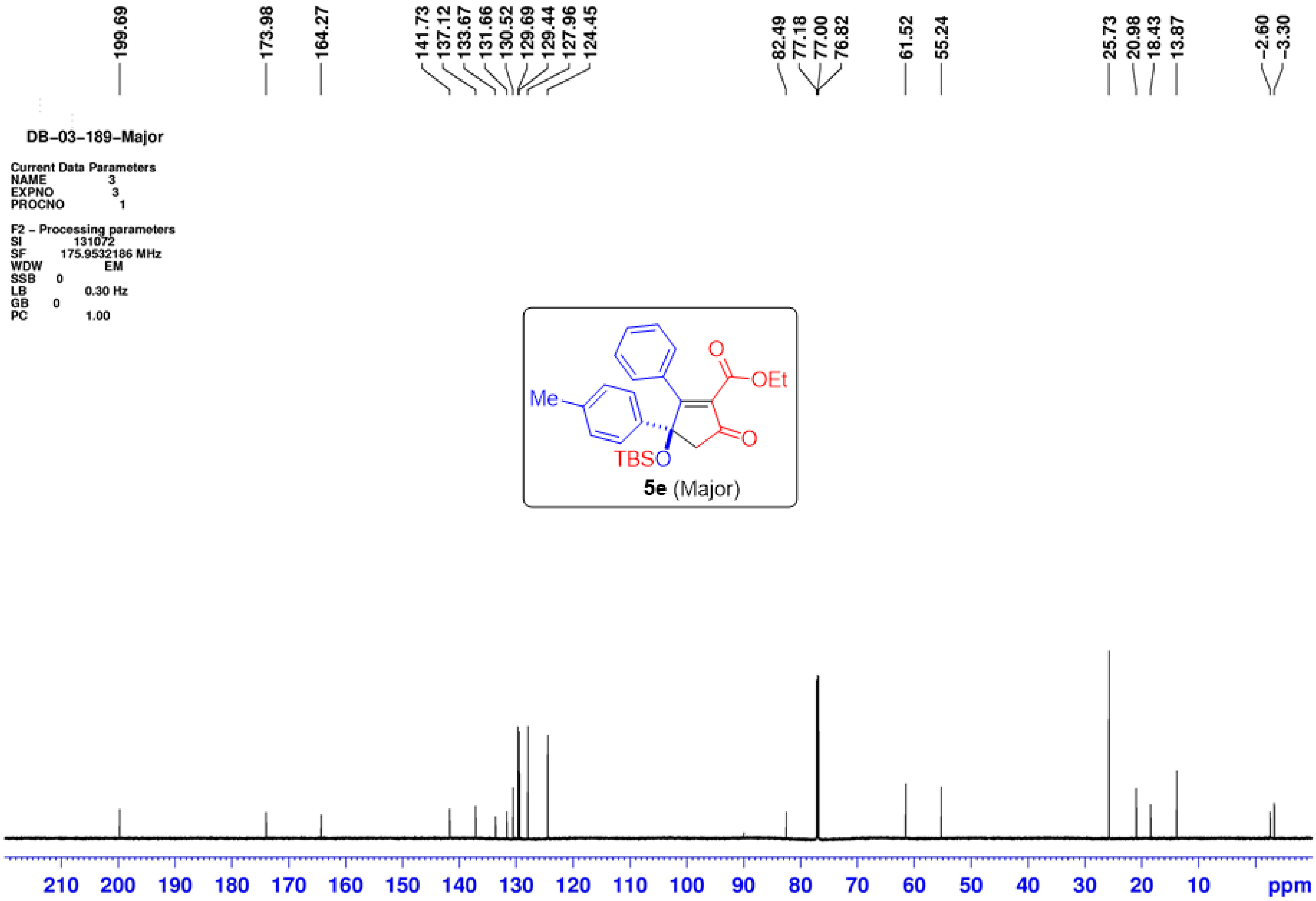

^{1}H NMR (CDCl$_3$, 700 MHz)

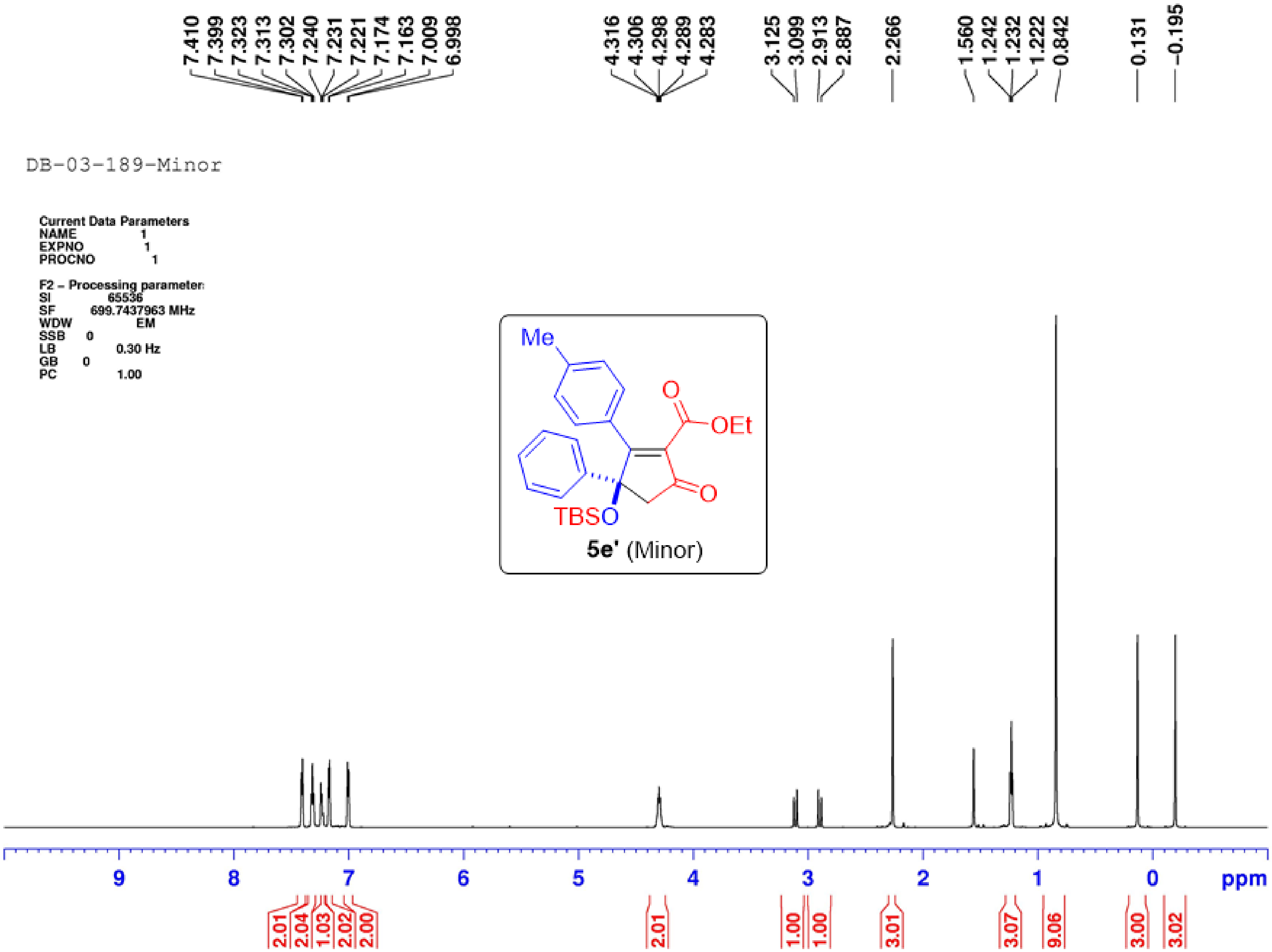

^{13}C NMR ($CDCl_3$, 175 MHz)

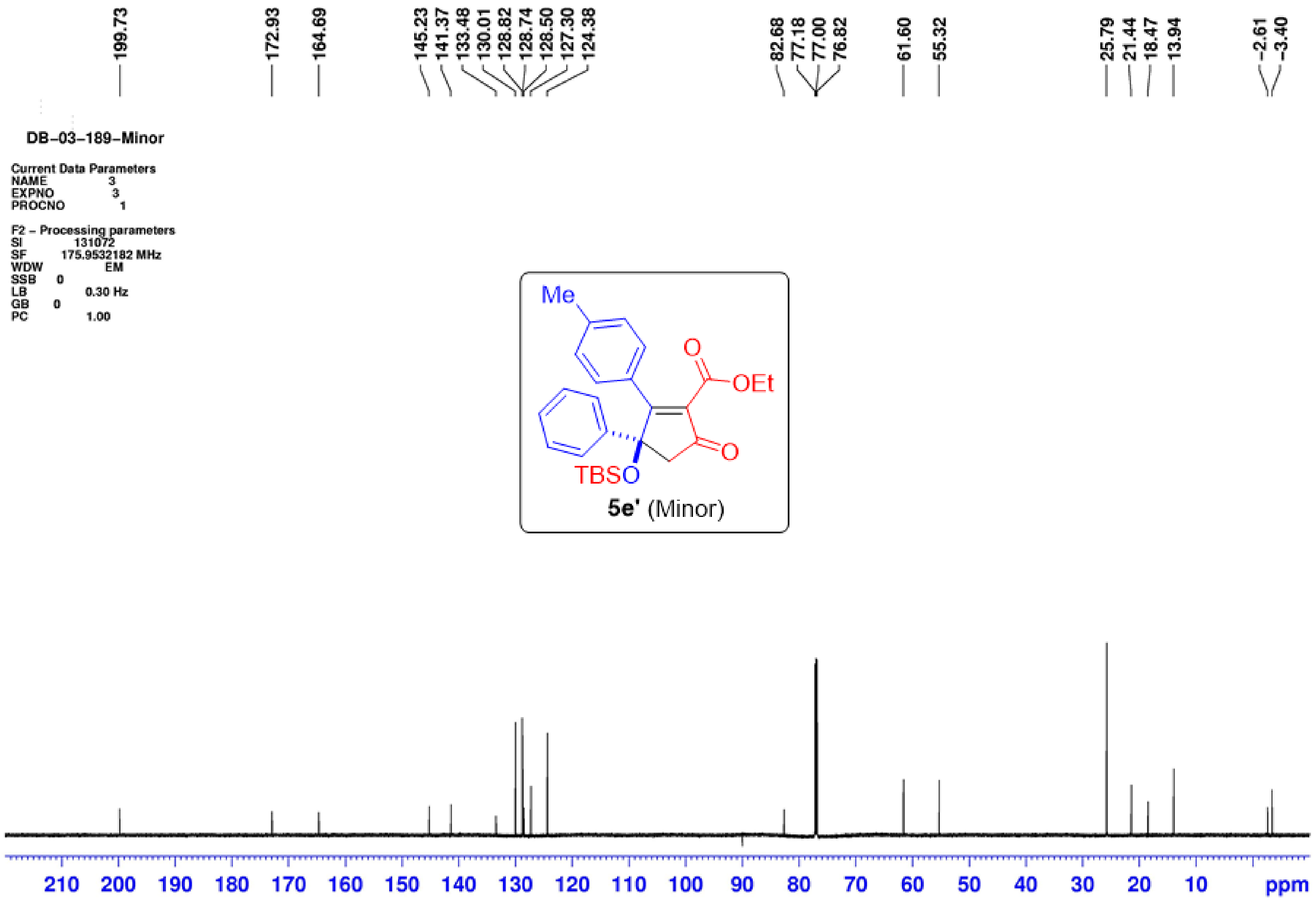

^{1}H NMR ($CDCl_3$, 700 MHz)

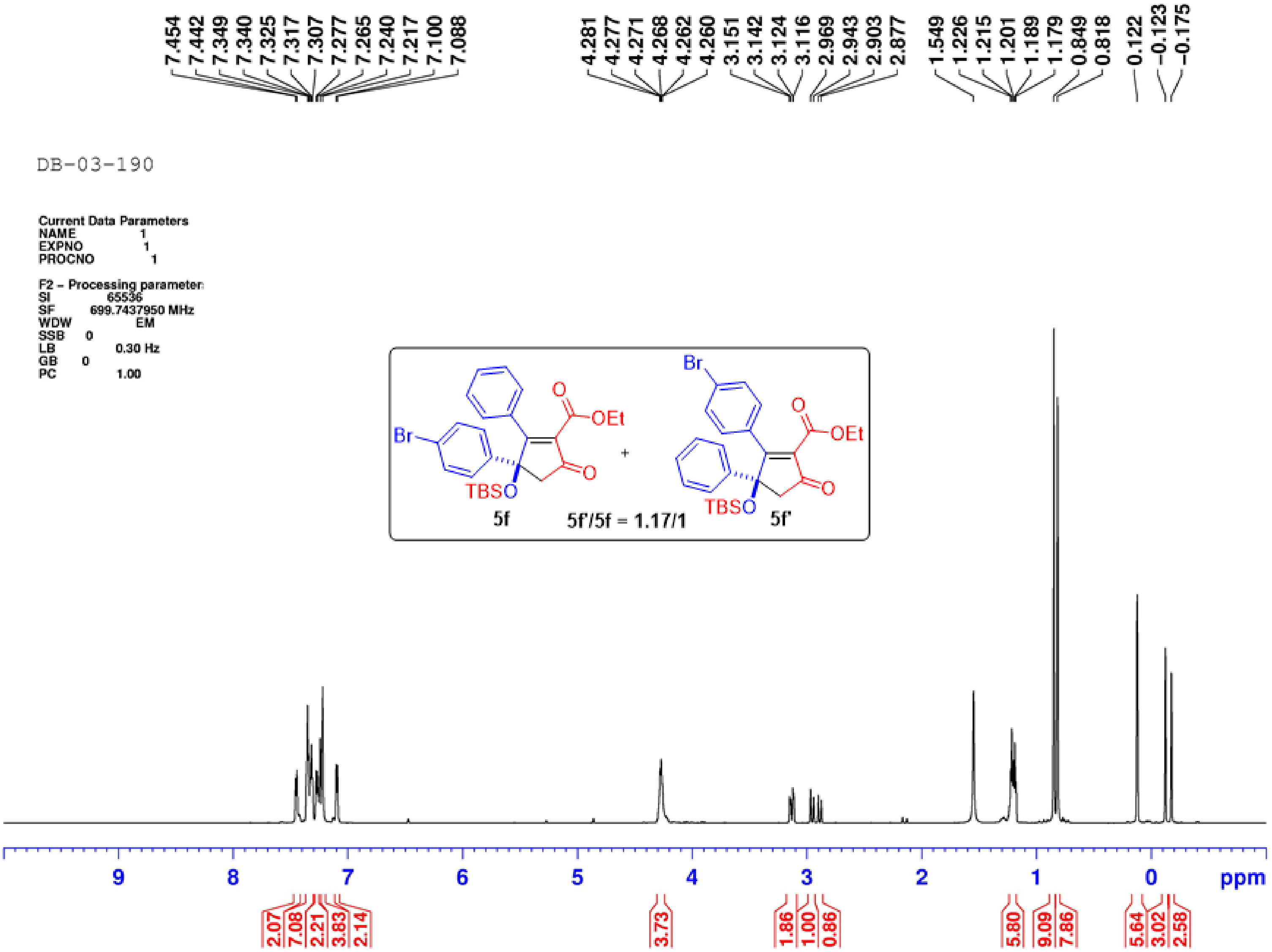

^{13}C NMR ($CDCl_3$, 175 MHz)

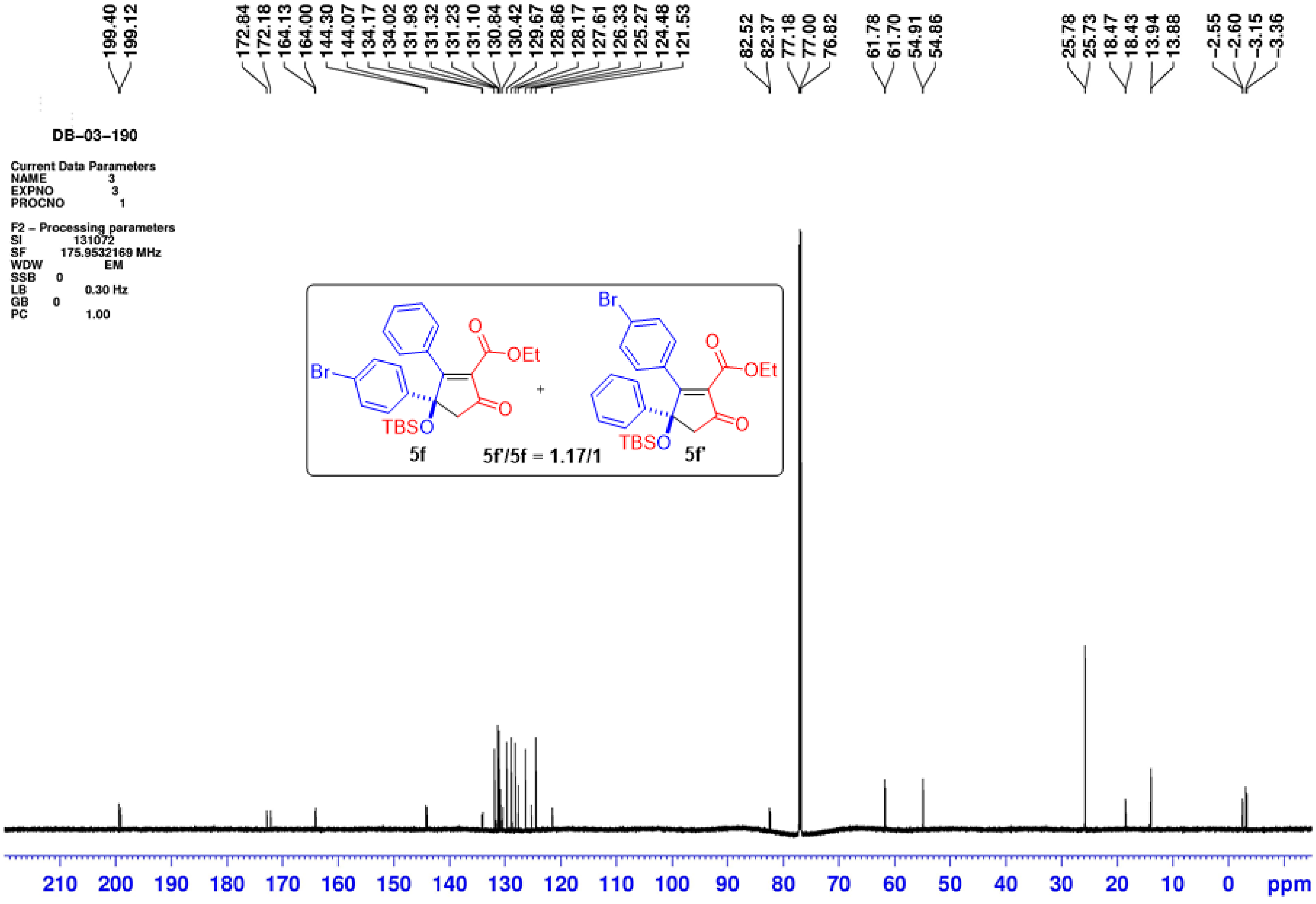

^{1}H NMR ($CDCl_3$, 700 MHz)

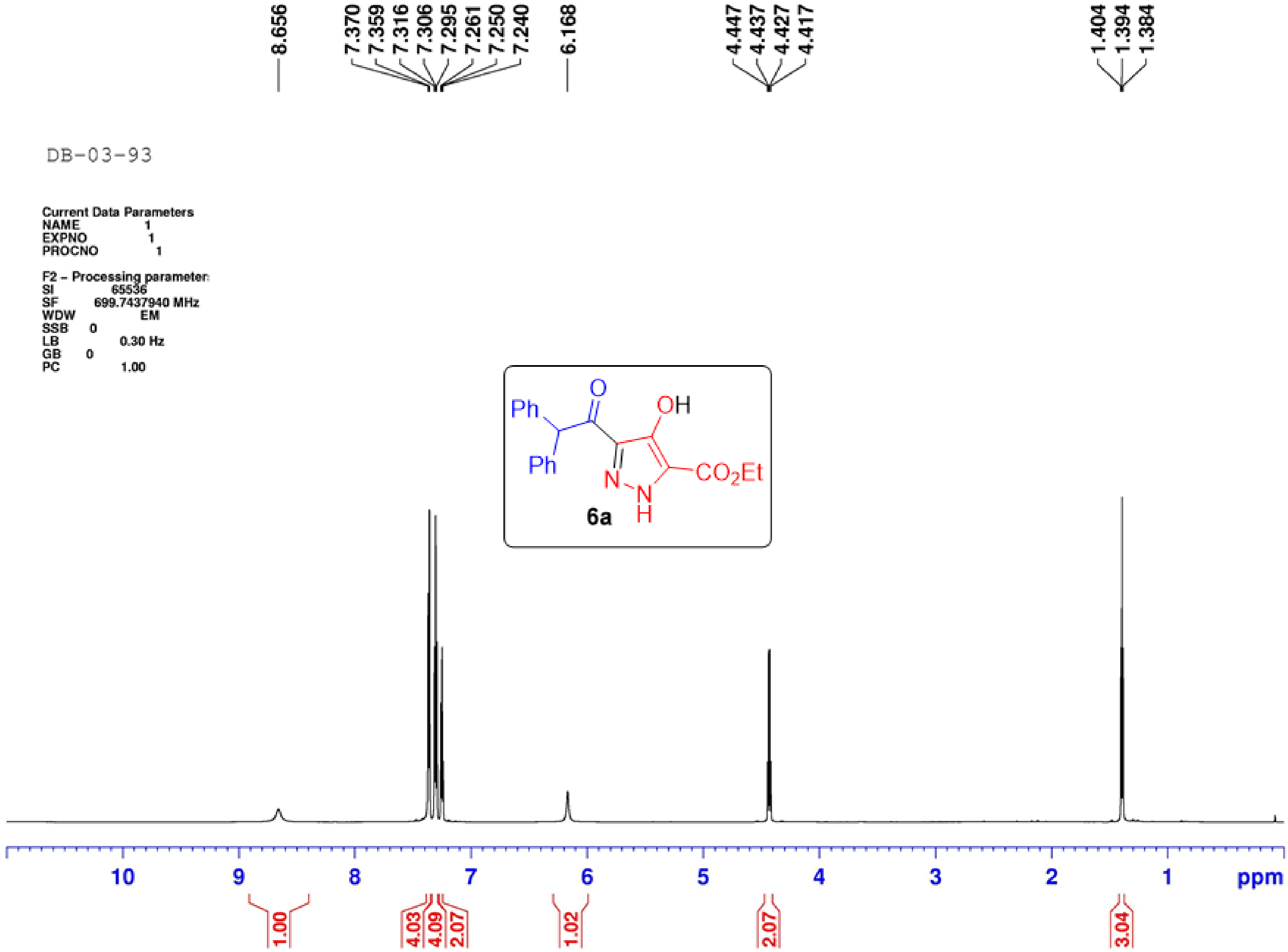

^{13}C NMR ($CDCl_3$, 175 MHz)

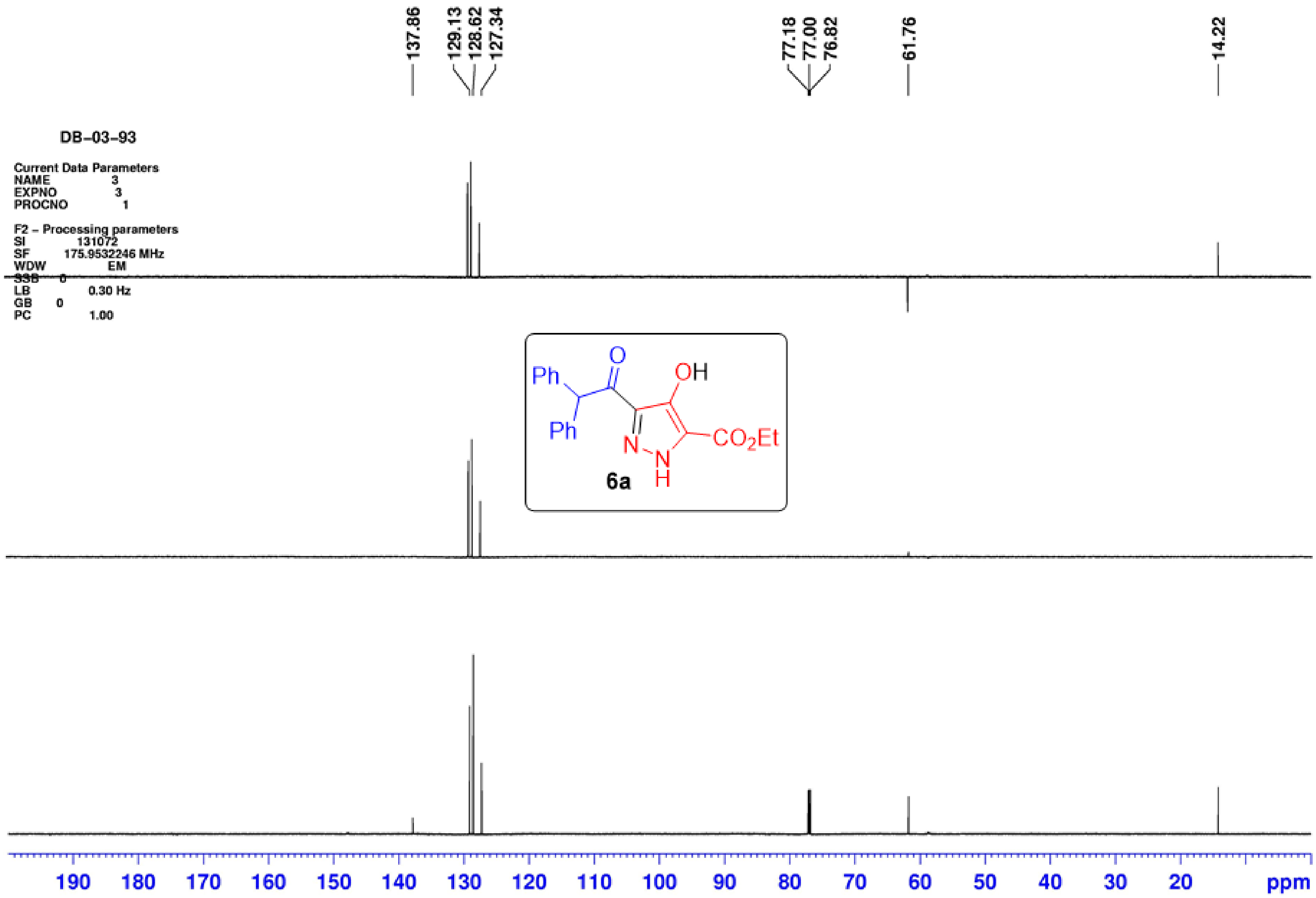

^{1}H NMR ($CDCl_3$, 700 MHz)

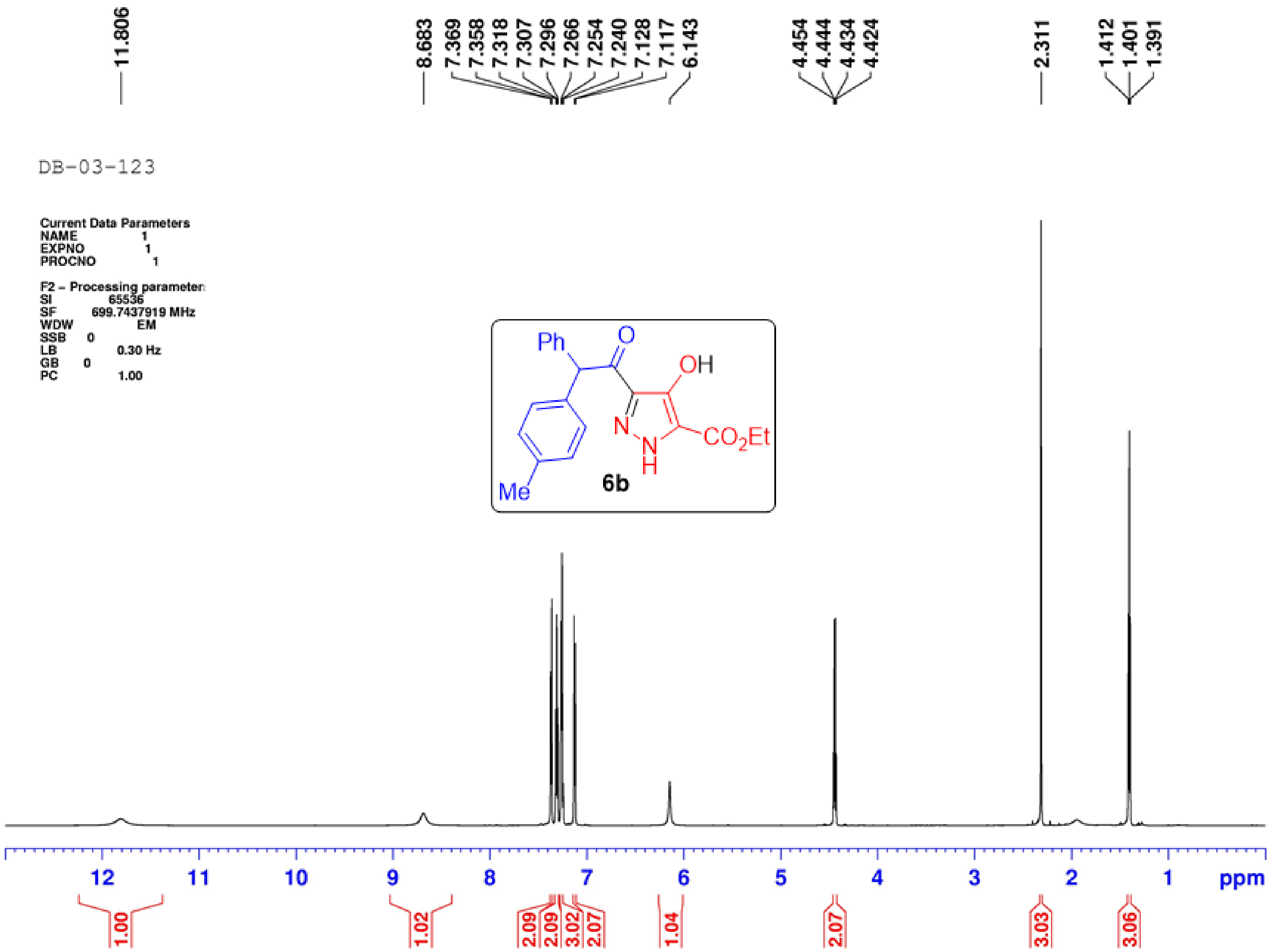

^{13}C NMR ($CDCl_3$, 175 MHz)

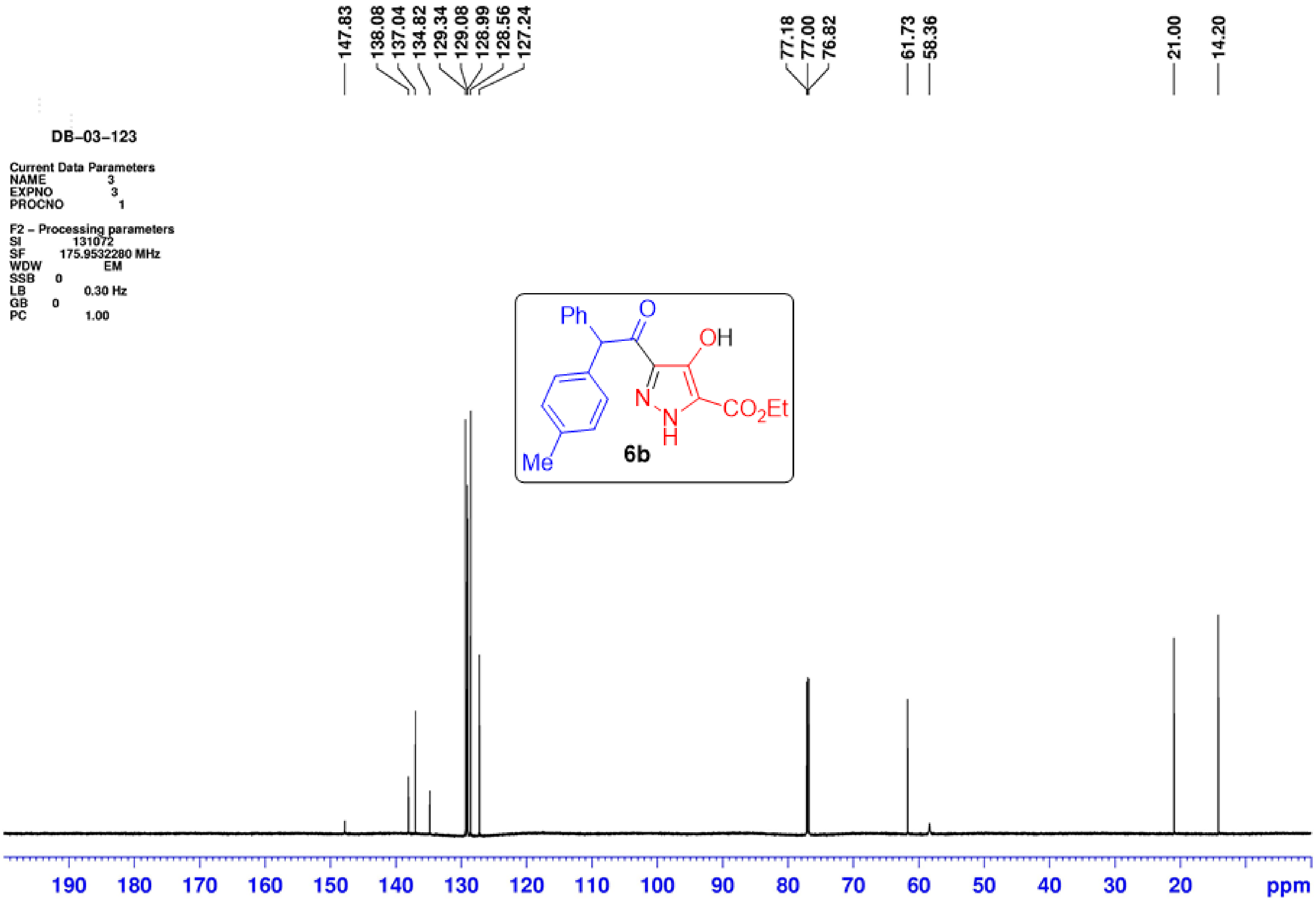

^{1}H NMR ($CDCl_3$, 700 MHz)

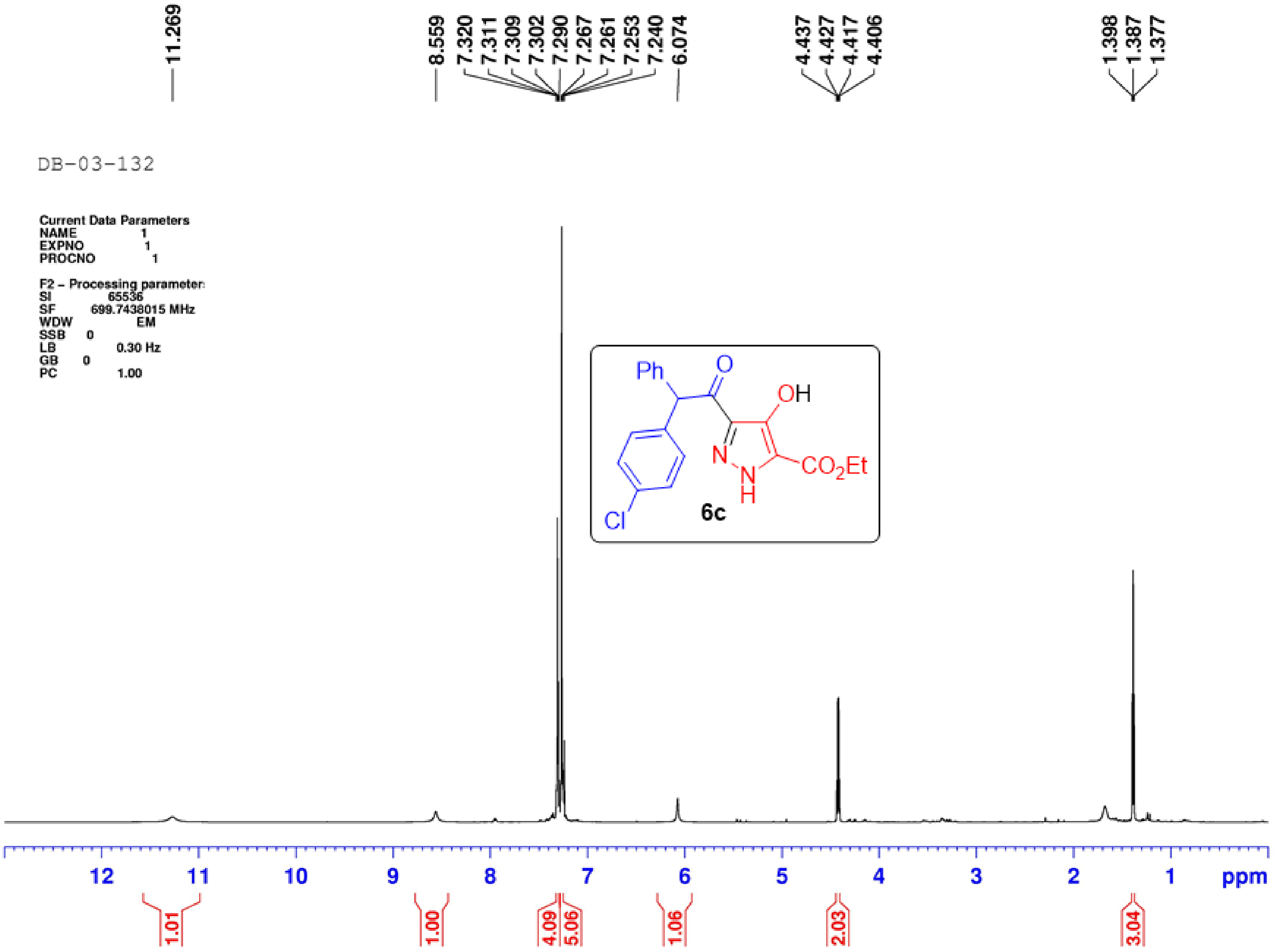

^{13}C NMR ($CDCl_3$, 175 MHz)

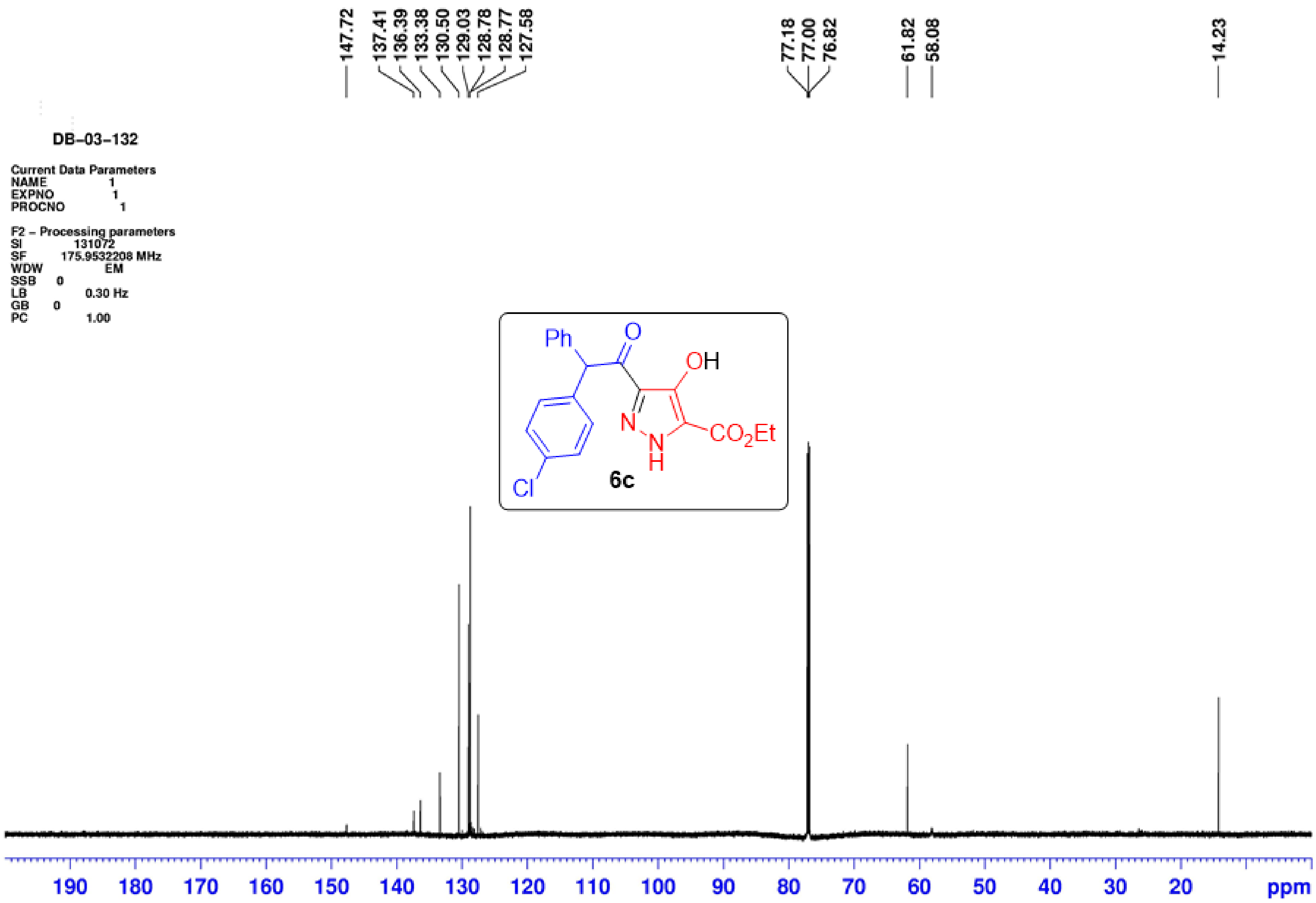

^{1}H NMR ($CDCl_3$, 700 MHz)

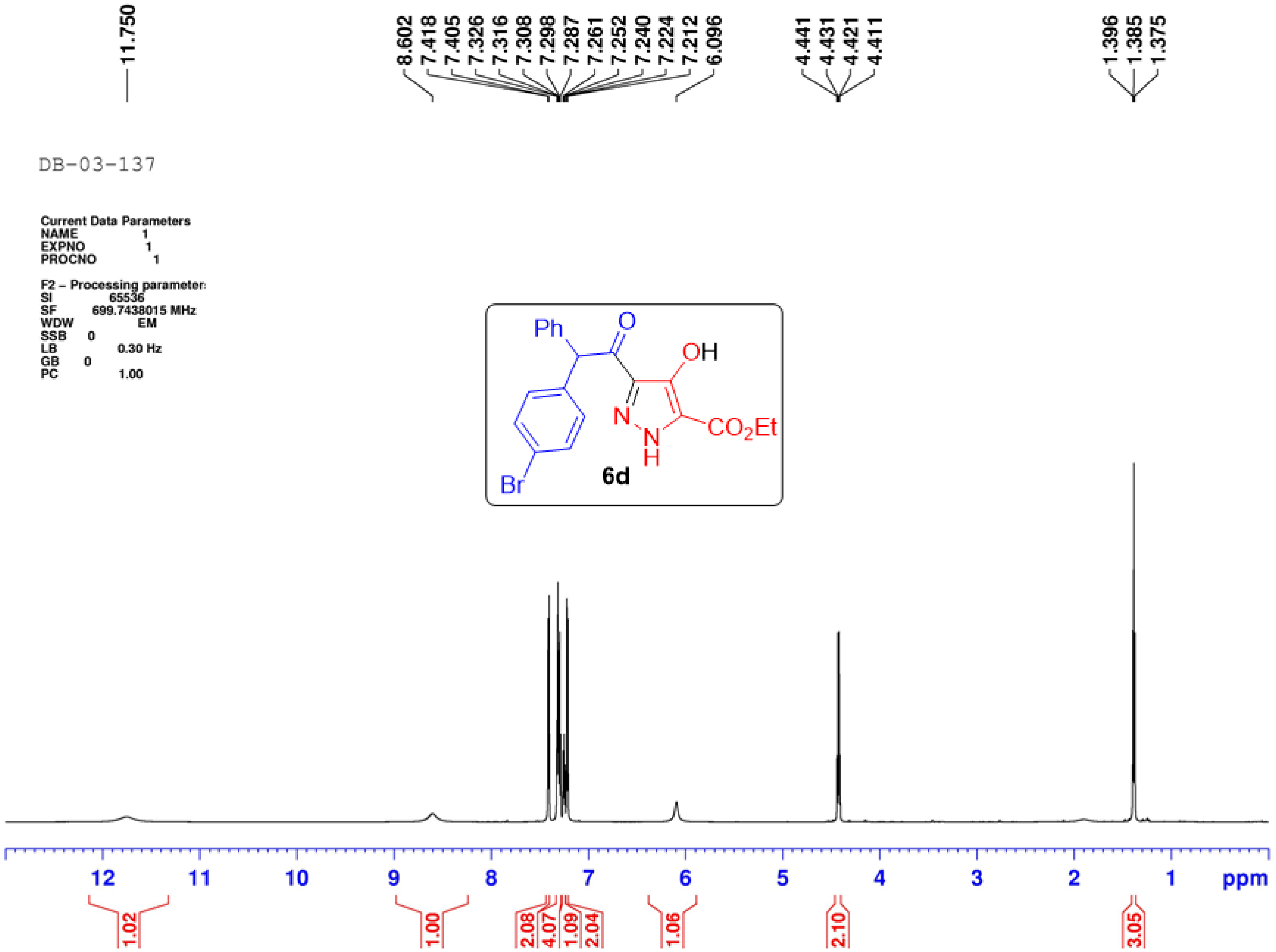

^{13}C NMR ($CDCl_3$, 175 MHz)

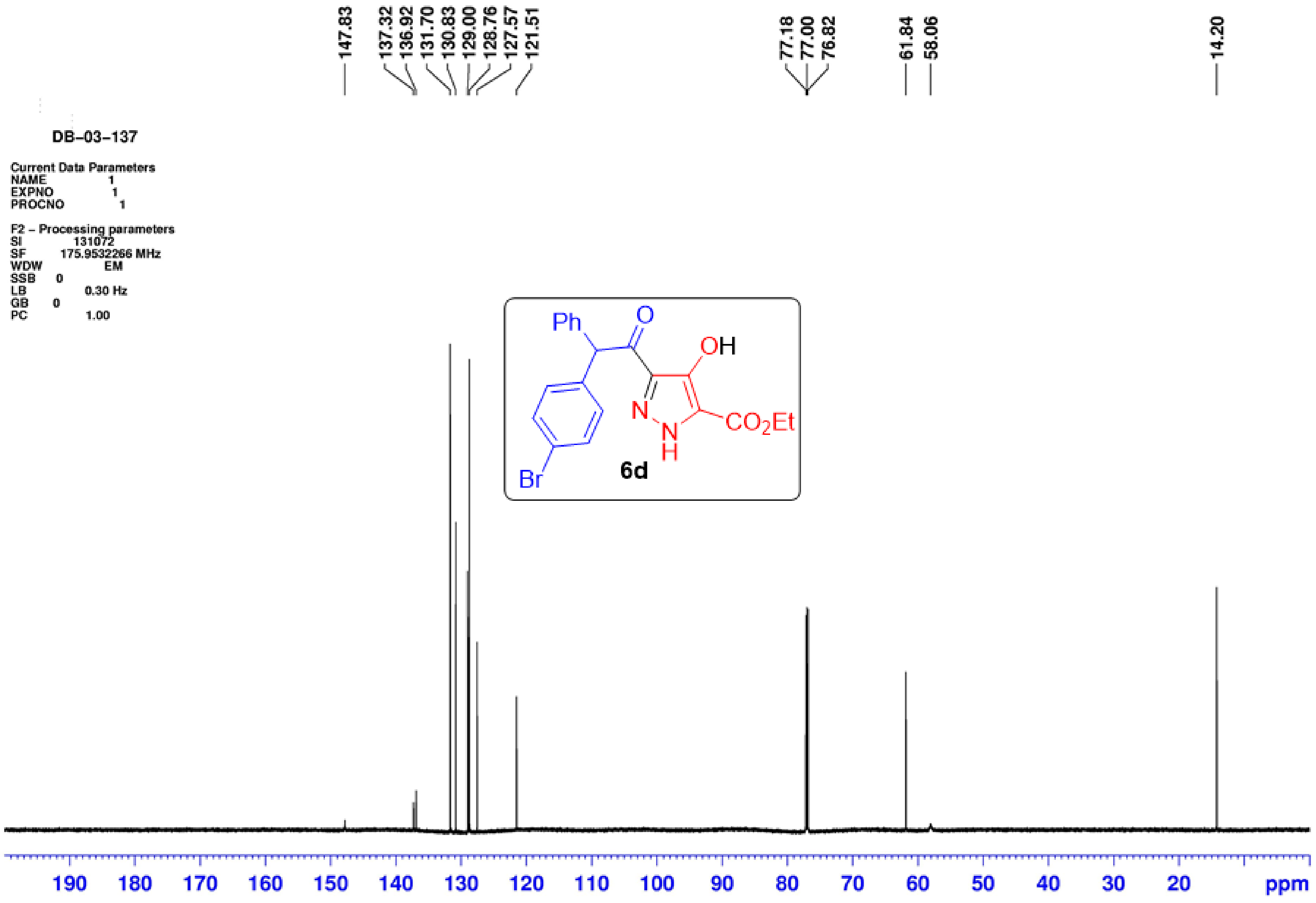

^{1}H NMR ($CDCl_3$, 700 MHz)

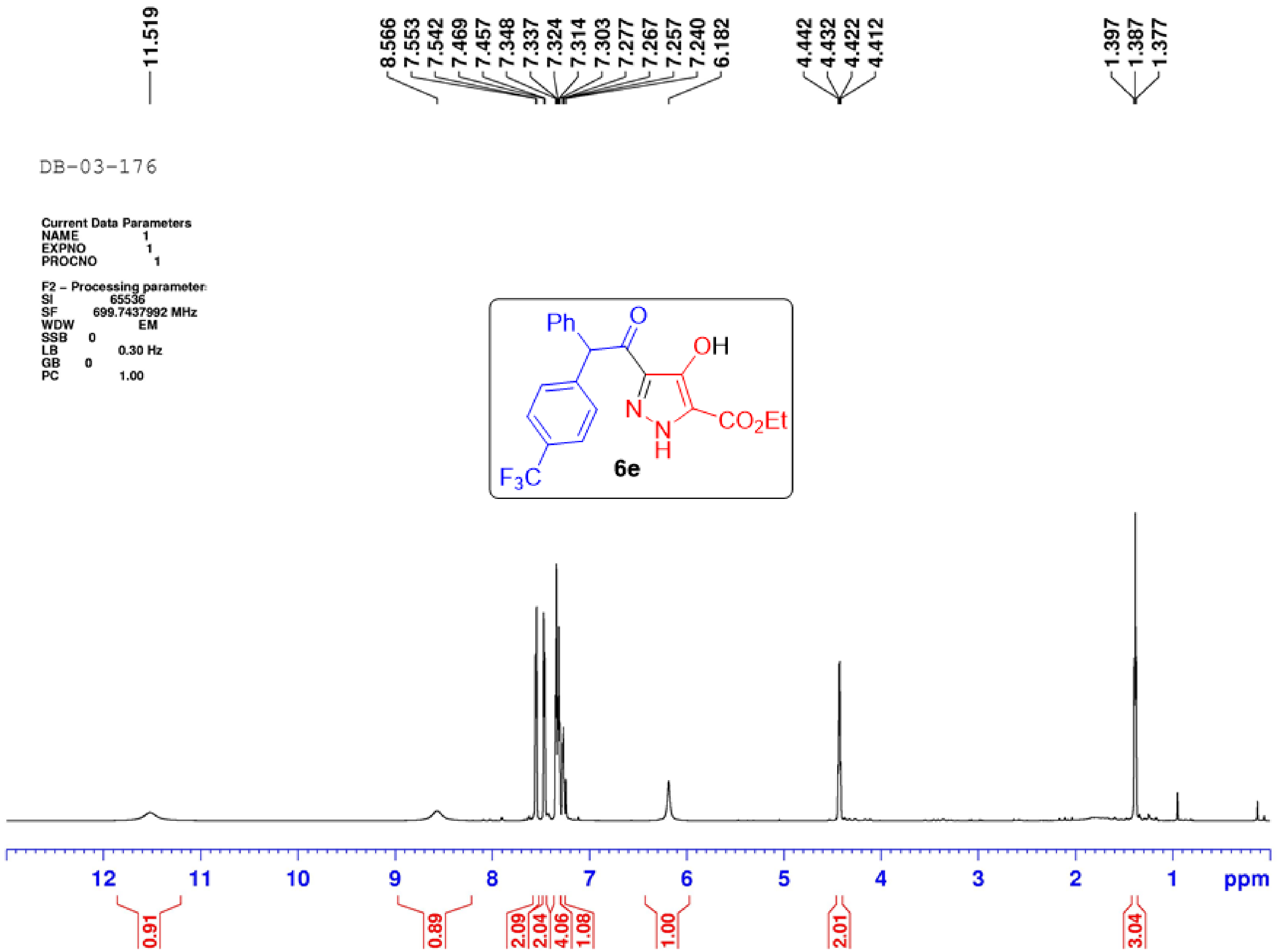

^{13}C NMR ($CDCl_3$, 175 MHz)

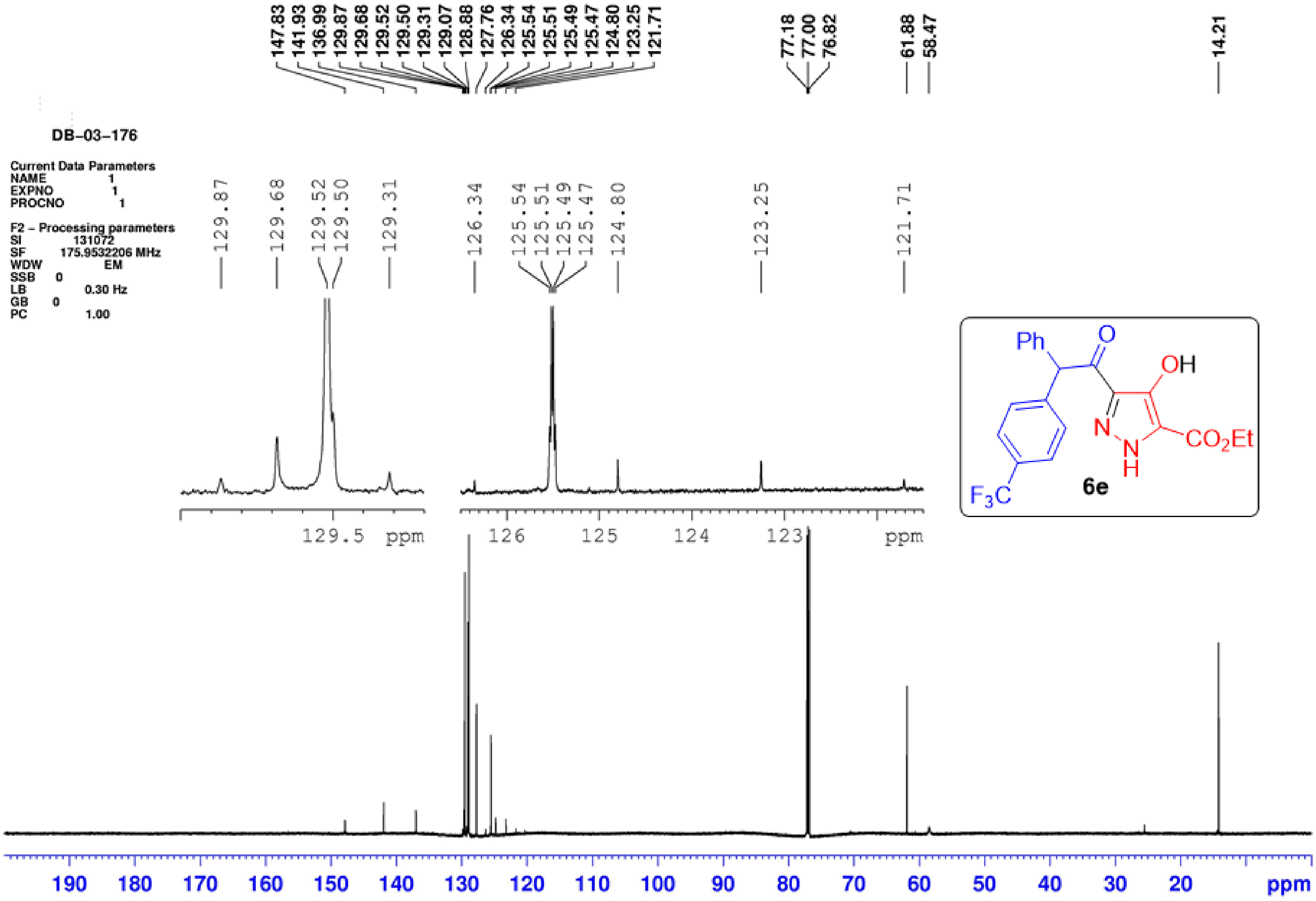

^{19}F NMR ($CDCl_3$, 471 MHz)

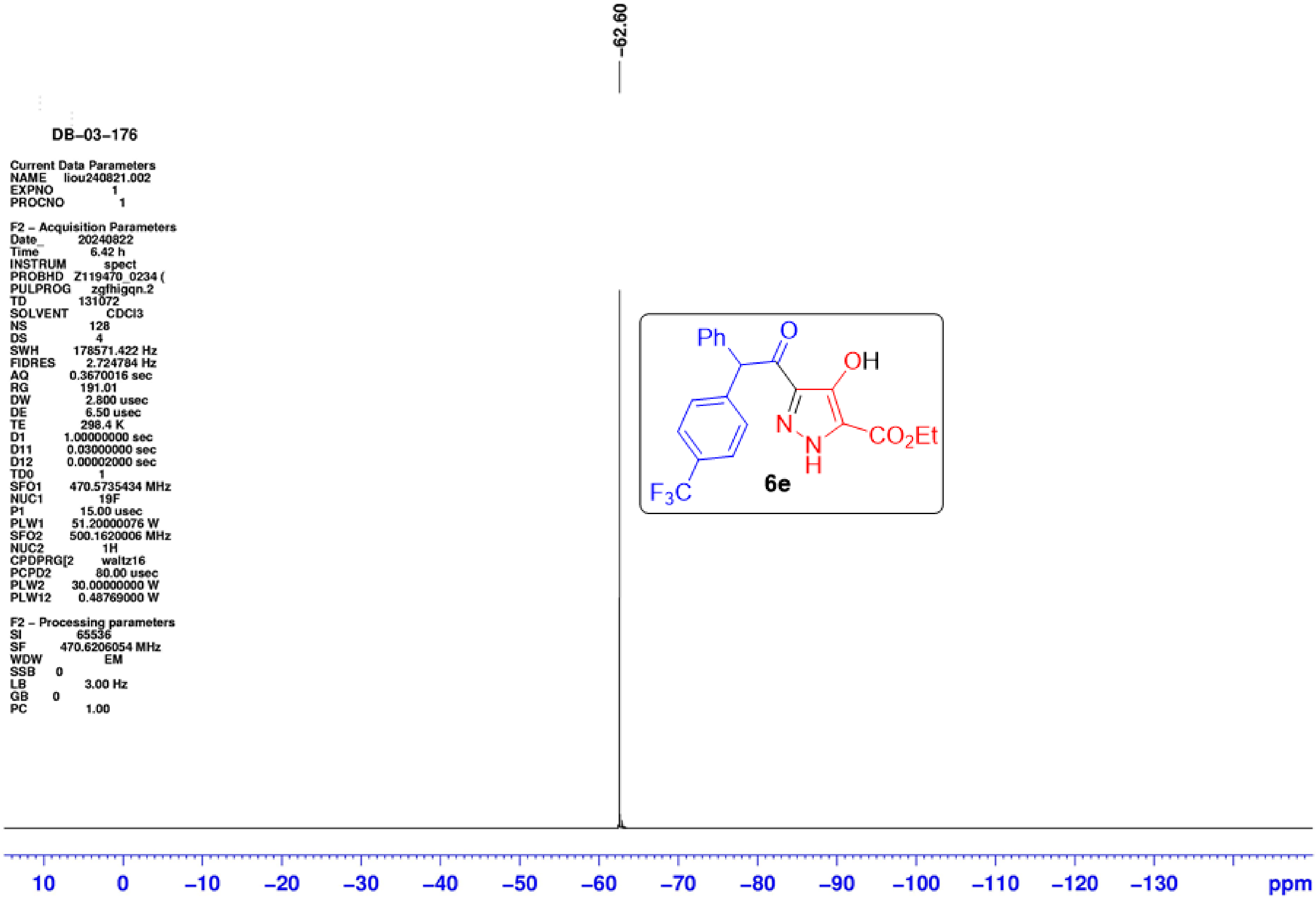

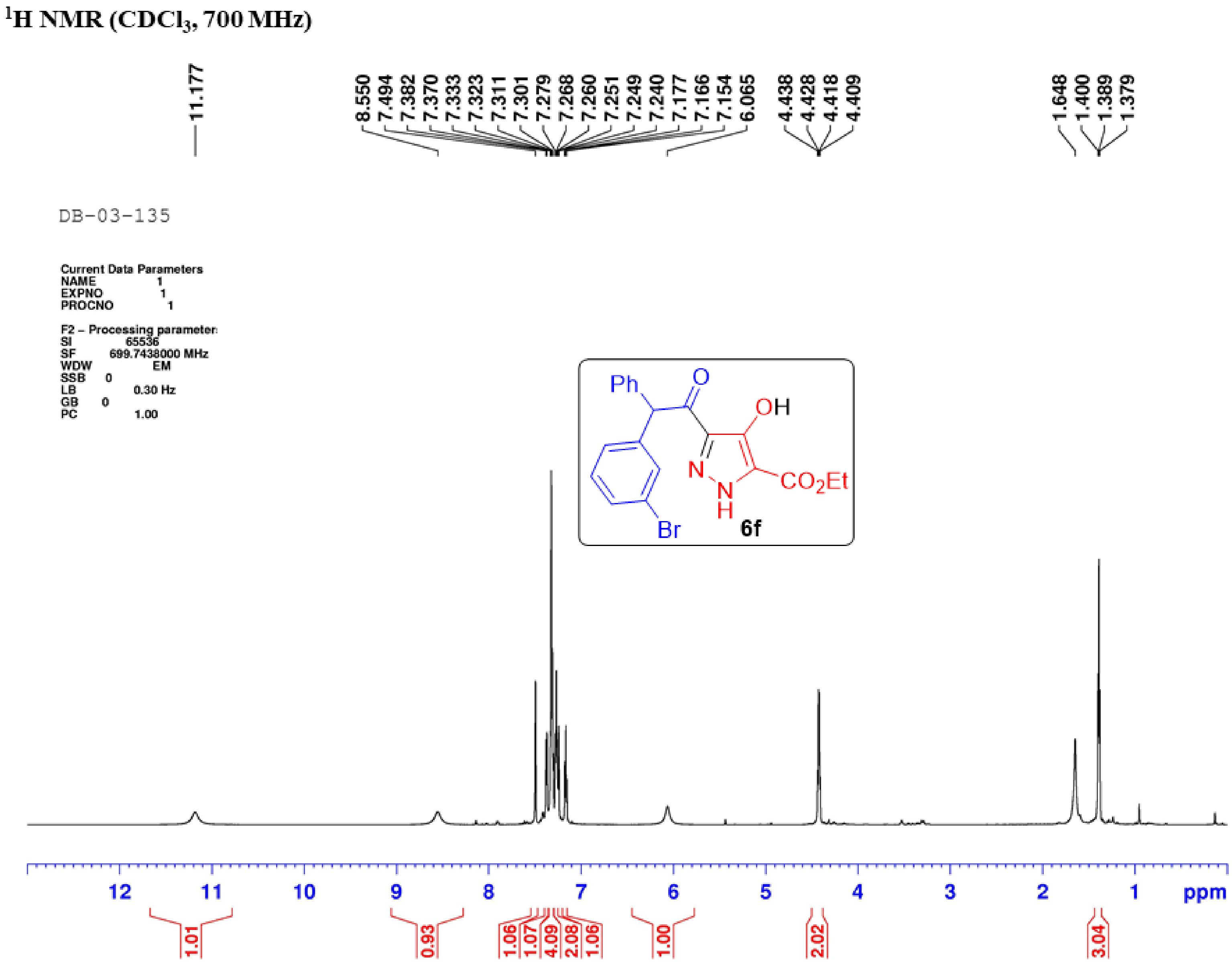

¹H NMR (CDCl₃, 700 MHz)
11.177
8.550
7.494
7.382
7.370
7.333
7.323
7.311
7.301
7.279
7.268
7.260
7.251
7.249
7.240
7.177
7.166
7.154
6.065
4.438
4.428
4.418
4.409
1.648
1.400
1.389
1.379
DB-03-135
Current Data Parameters
NAME 1
EXPNO 1
PROCNO 1
F2 – Processing parameter:
SI 65536
SF 699.7438000 MHz
WDW EM
SSB 0
LB 0.30 Hz
GB 0
PC 1.00
Ph
O
OH
N
N
H
CO2Et
Br
6f
12
11
10
9
8
7
6
5
4
3
2
1
ppm
1.01
0.93
1.06
1.07
4.09
2.08
1.06
1.00
2.02
3.04

^{13}C NMR ($CDCl_3$, 175 MHz)

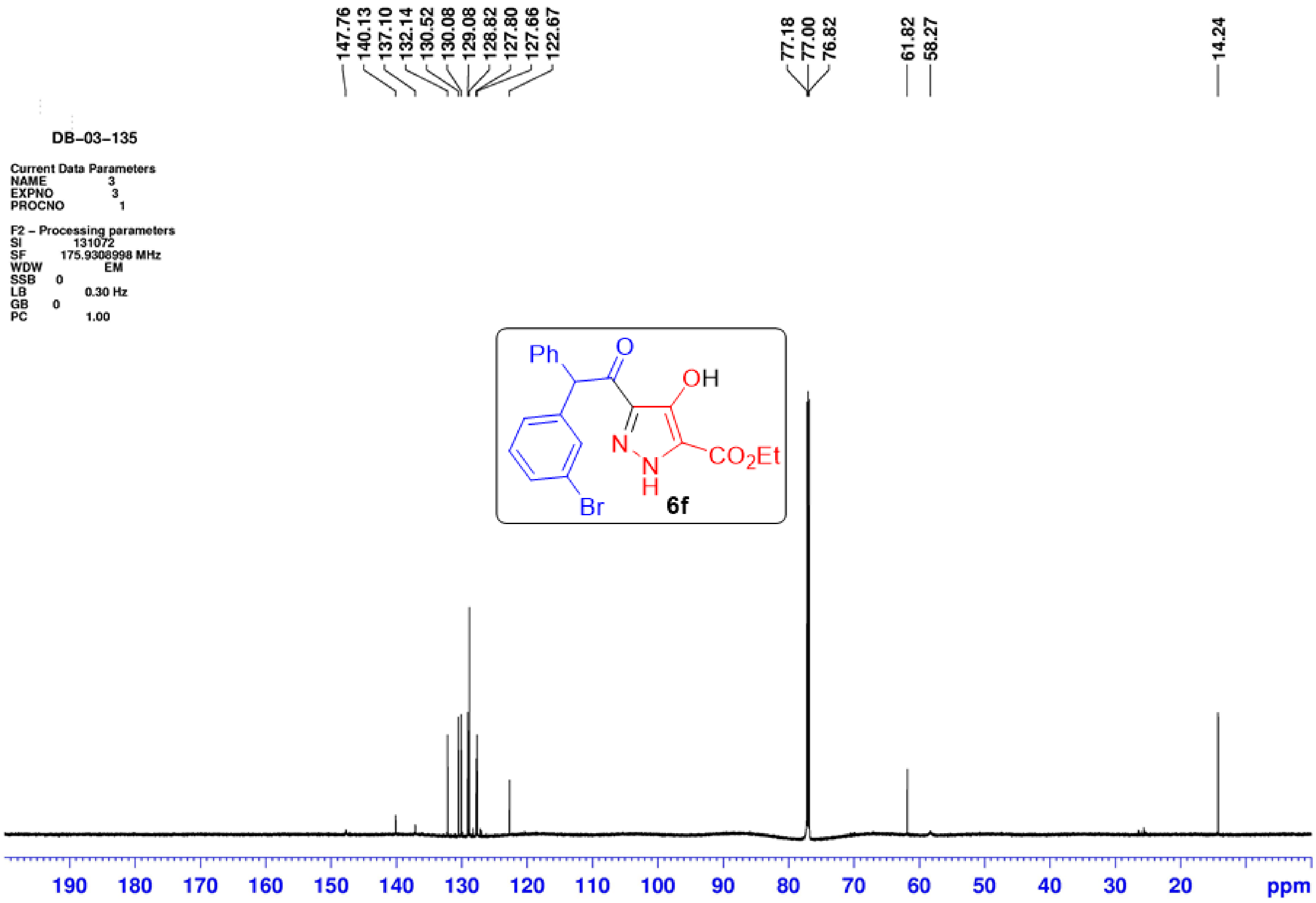

^{1}H NMR ($CDCl_3$, 700 MHz)

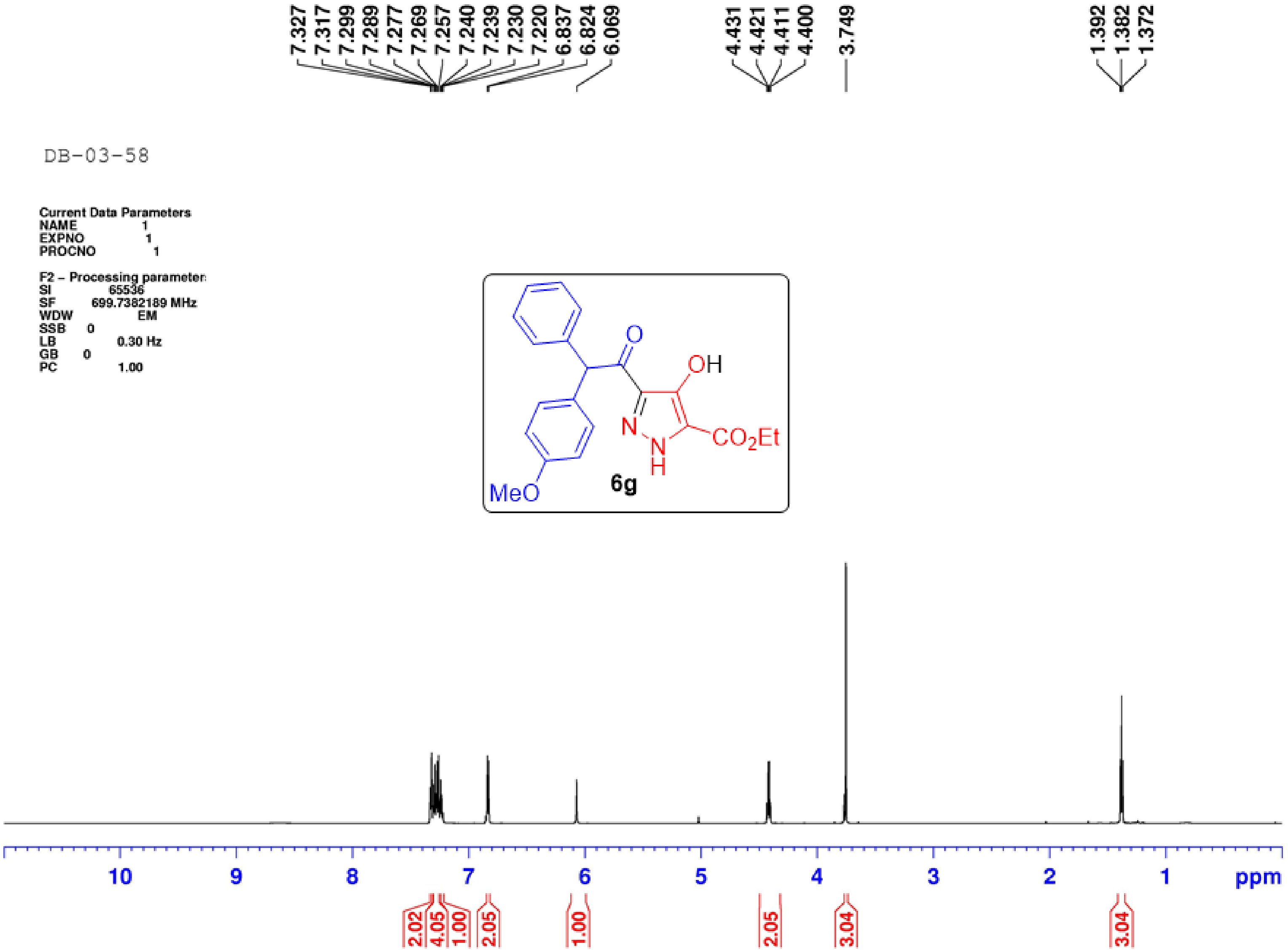

^{13}C NMR ($CDCl_3$, 175 MHz)

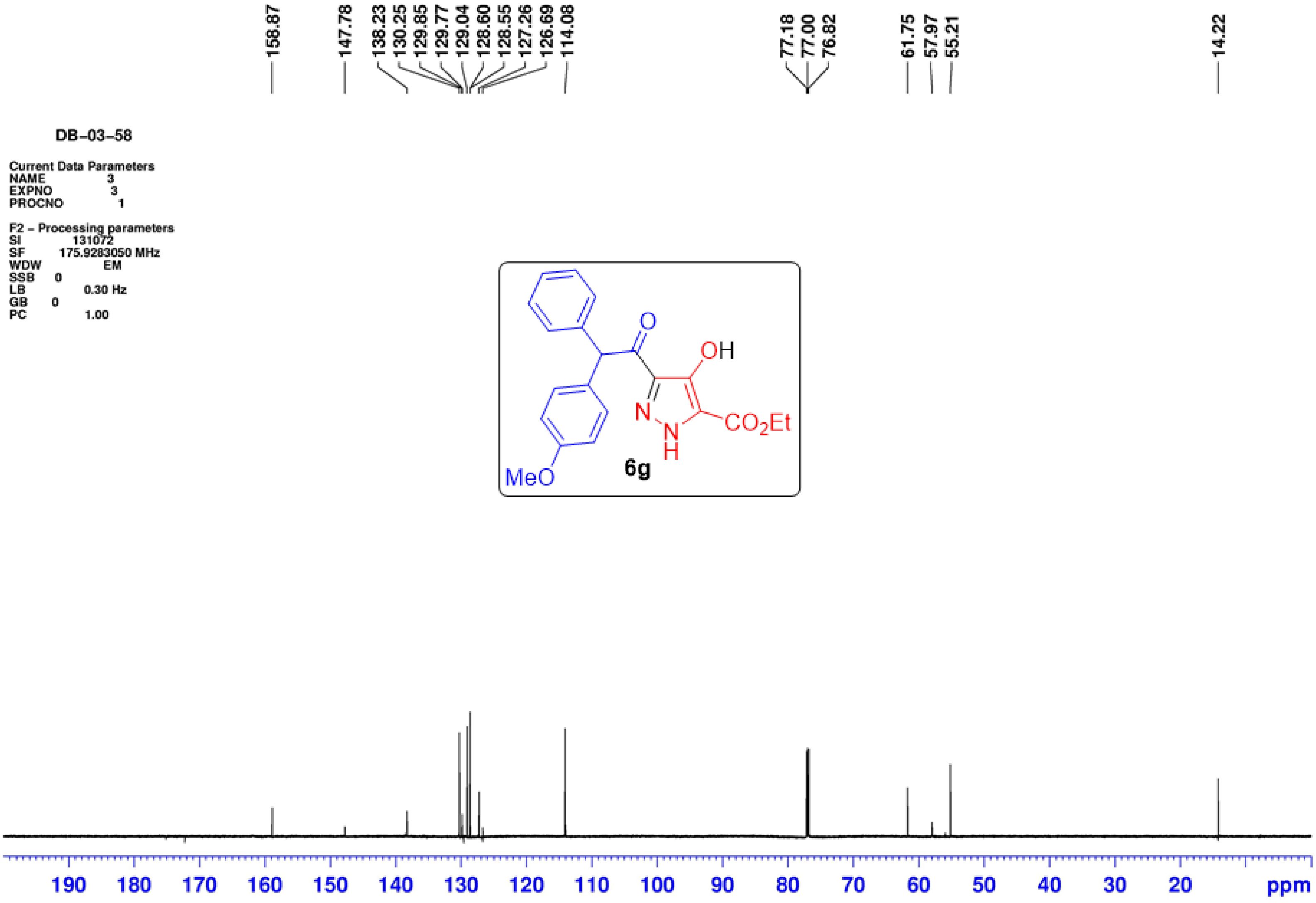

^{1}H NMR ($CDCl_3$, 400 MHz)

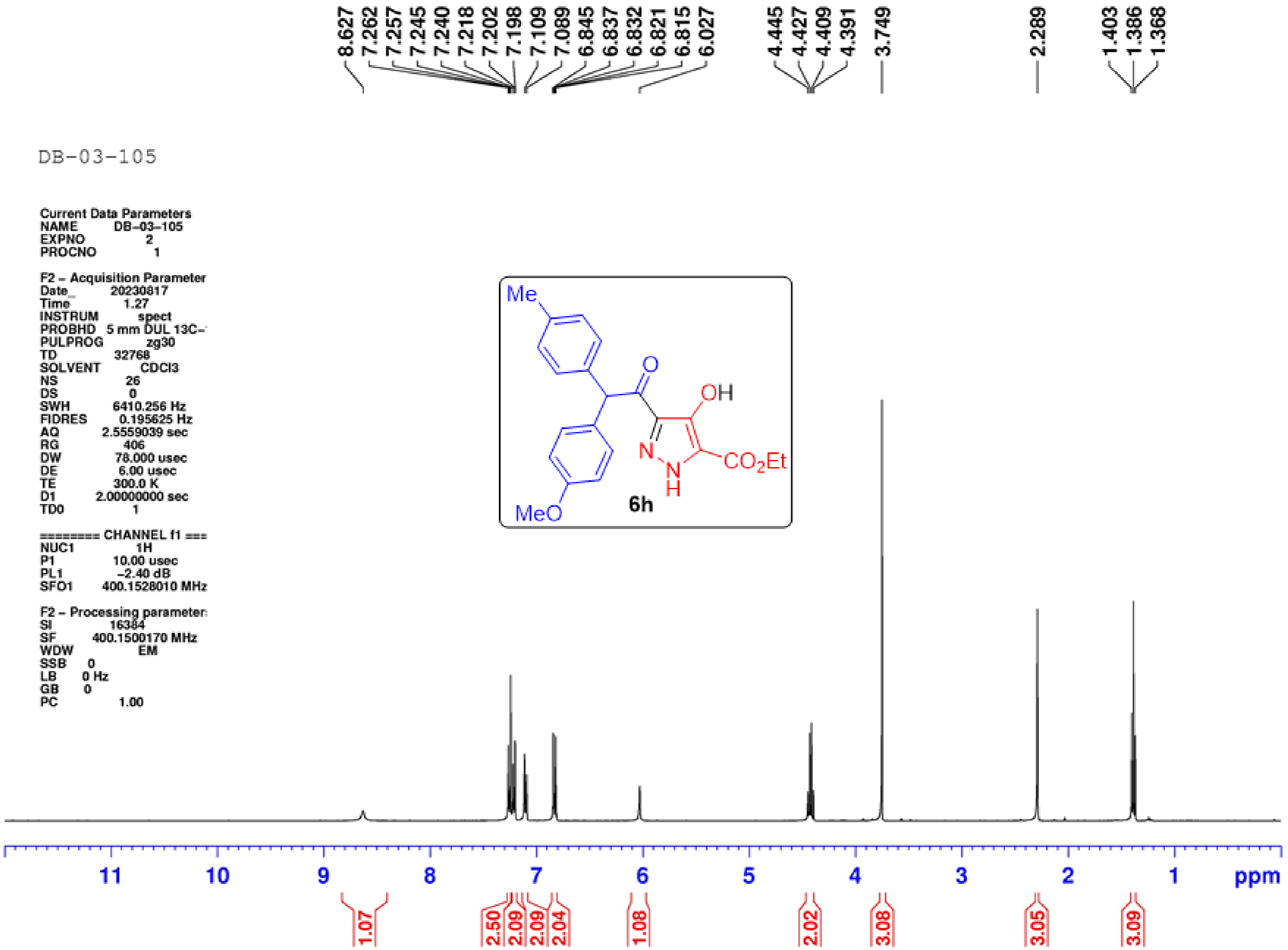

^{13}C NMR ($CDCl_3$, 100 MHz)

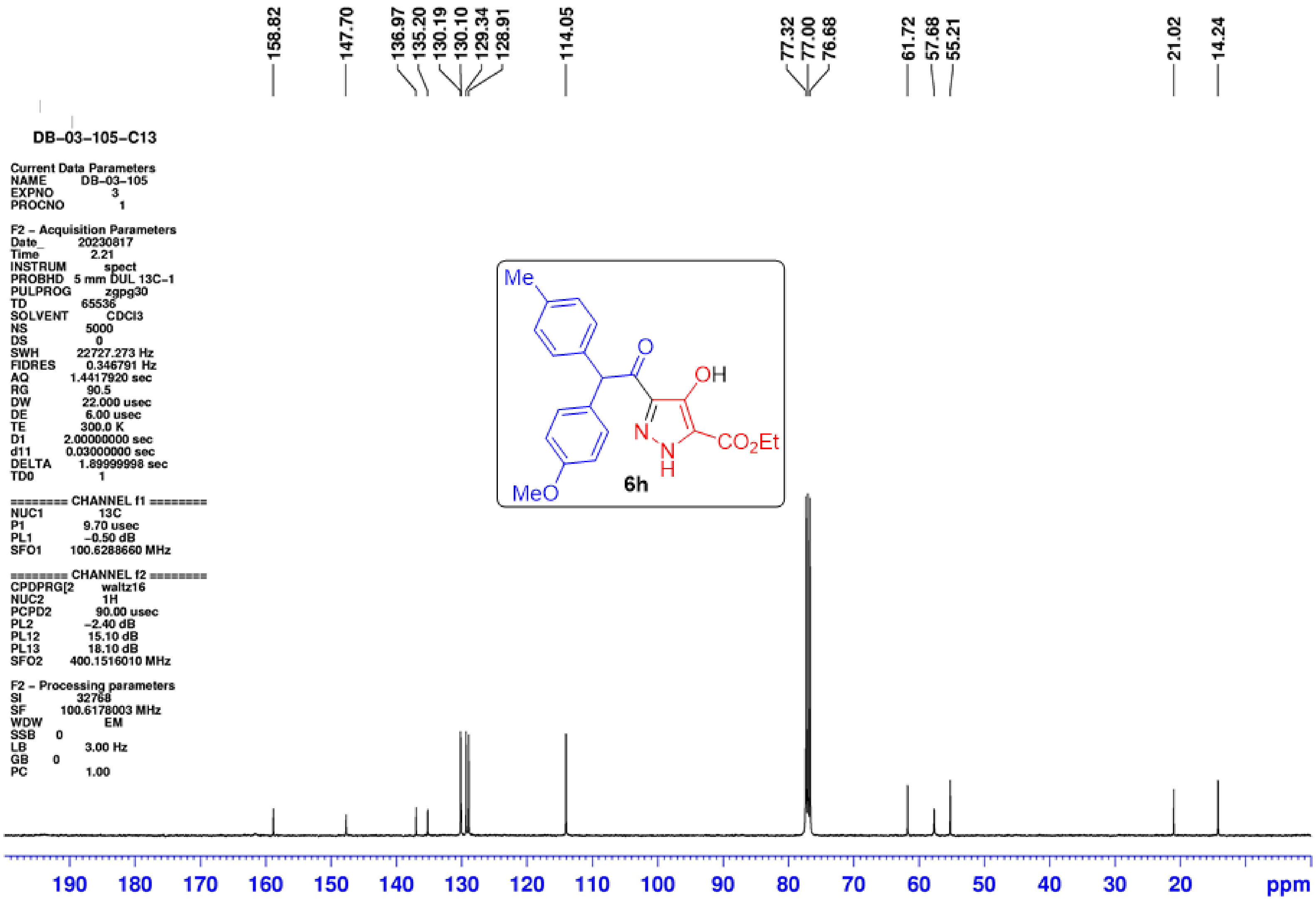

^{1}H NMR ($CDCl_3$, 700 MHz)

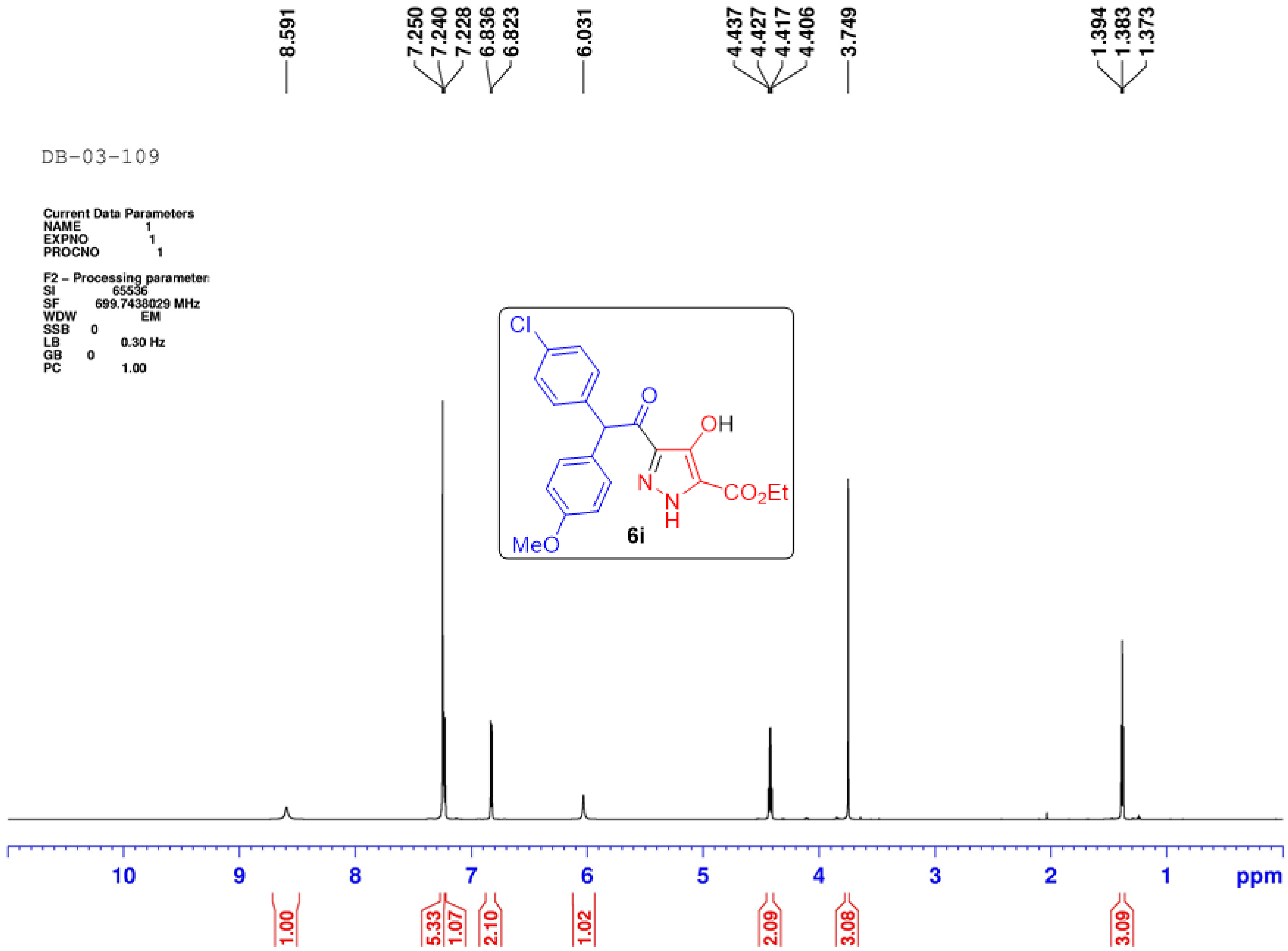

^{13}C NMR ($CDCl_3$, 175 MHz)

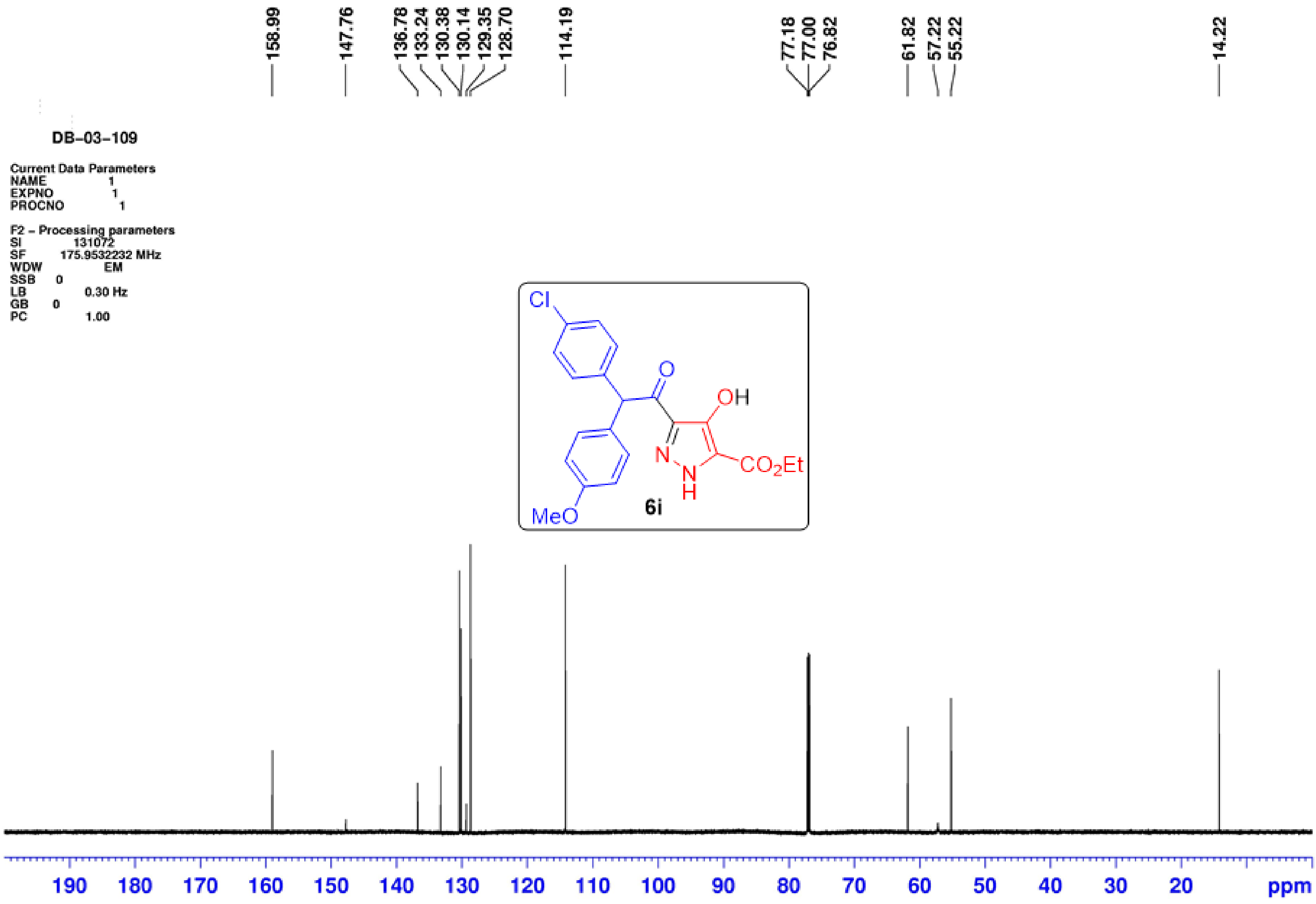

^{1}H NMR ($CDCl_3$, 700 MHz)

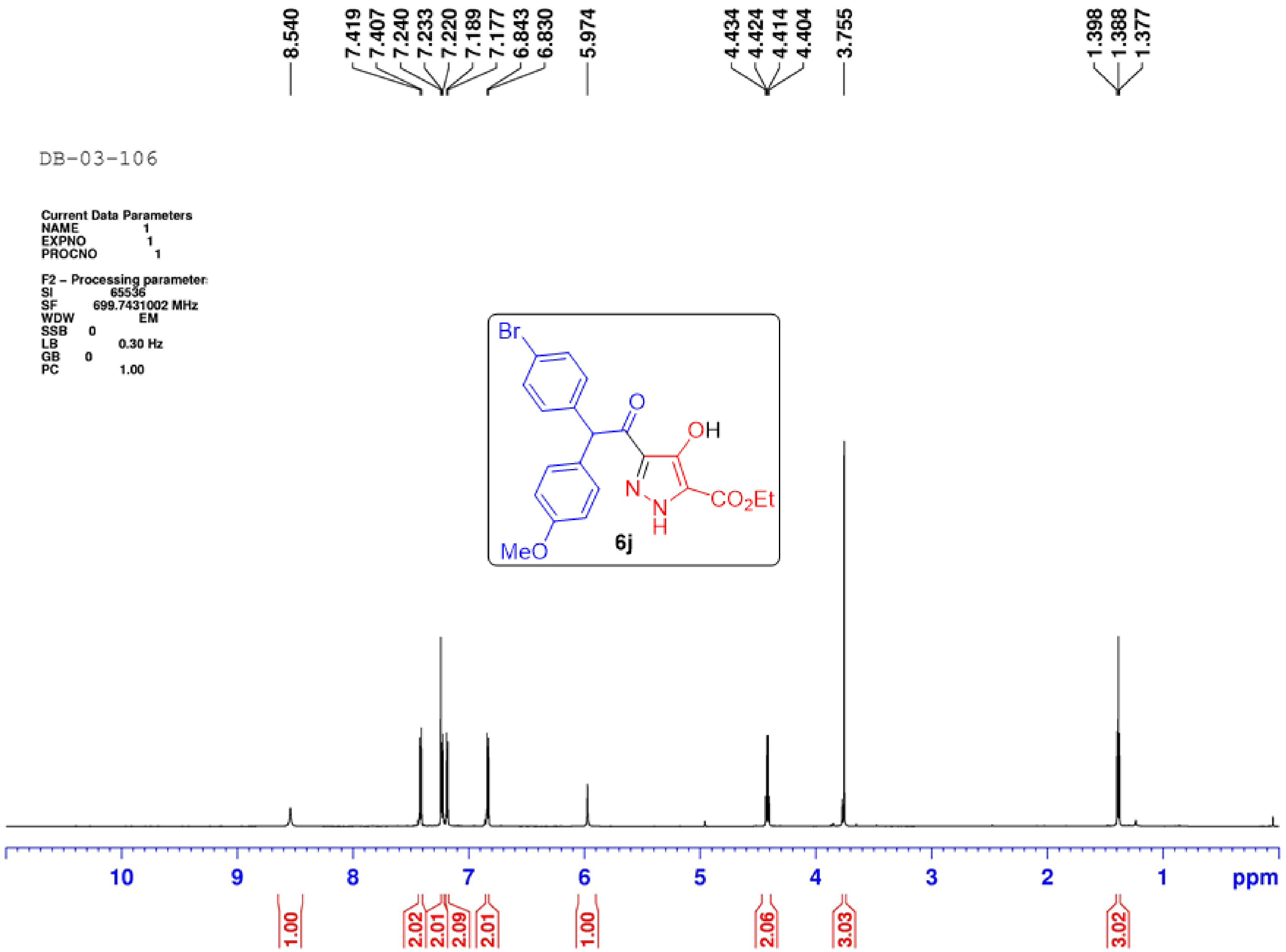

^{13}C NMR ($CDCl_3$, 175 MHz)

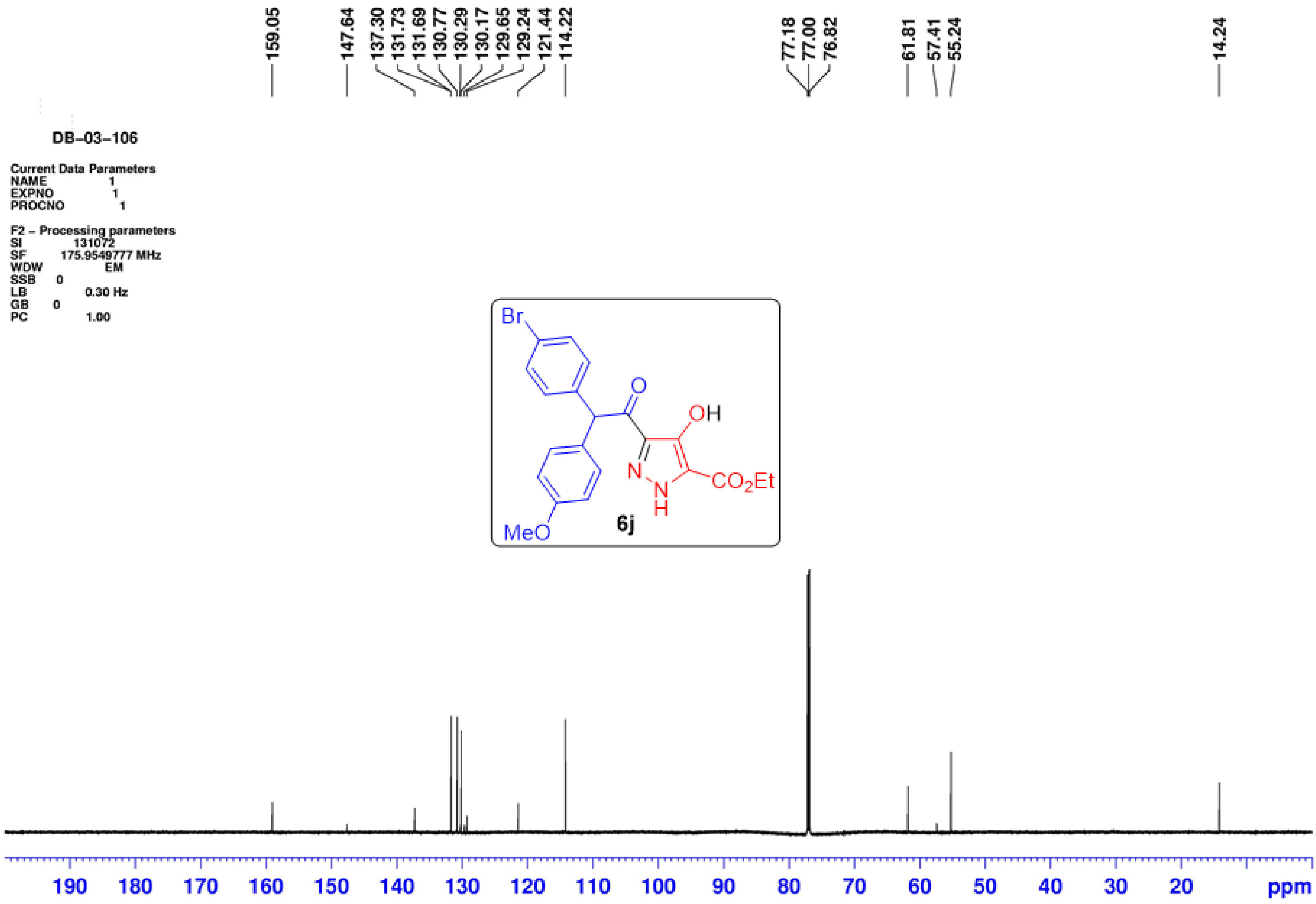

^{1}H NMR ($CDCl_3$, 700 MHz)

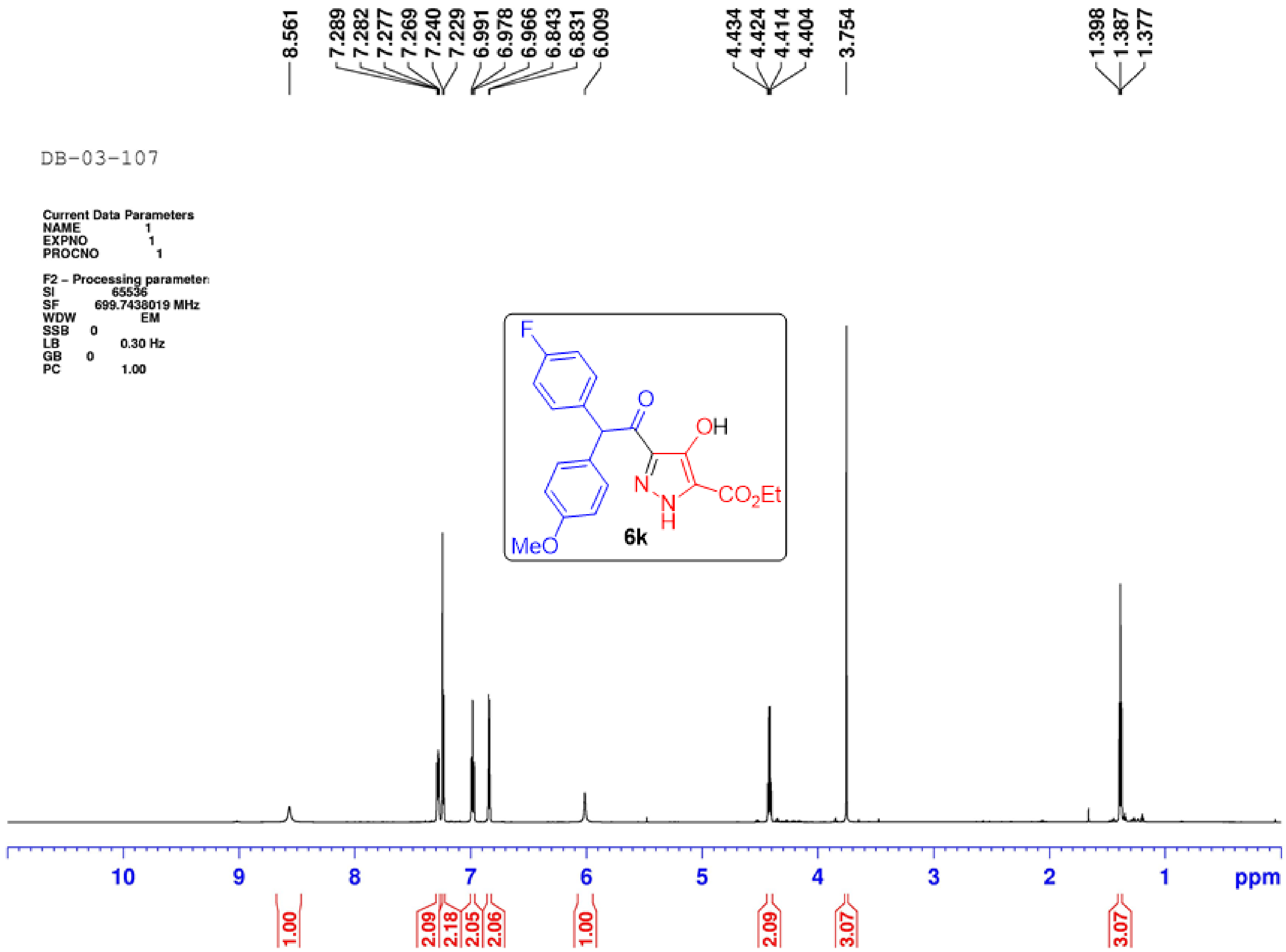

^{13}C NMR ($CDCl_3$, 175 MHz)

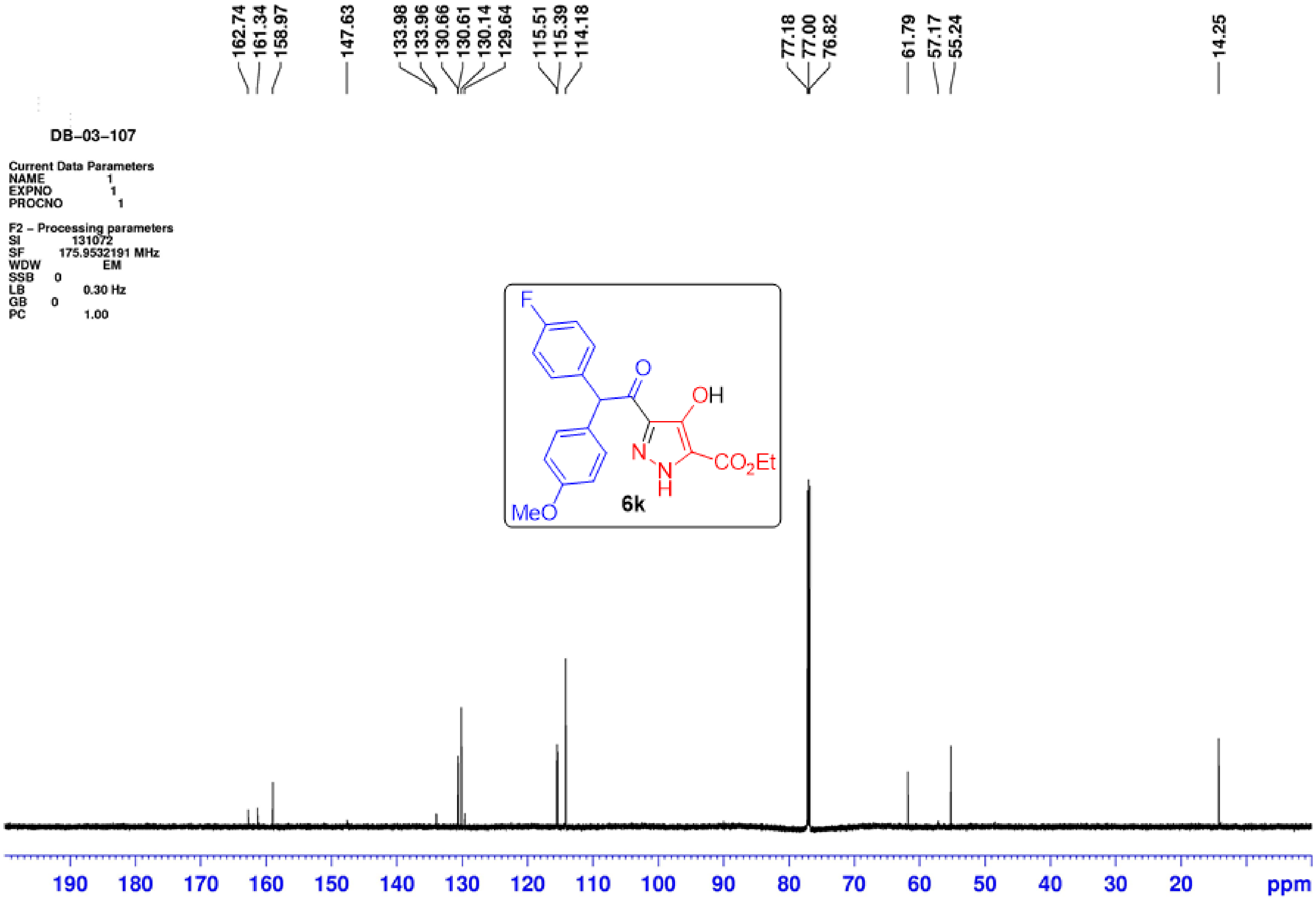

^{19}F NMR ($CDCl_3$, 471 MHz)

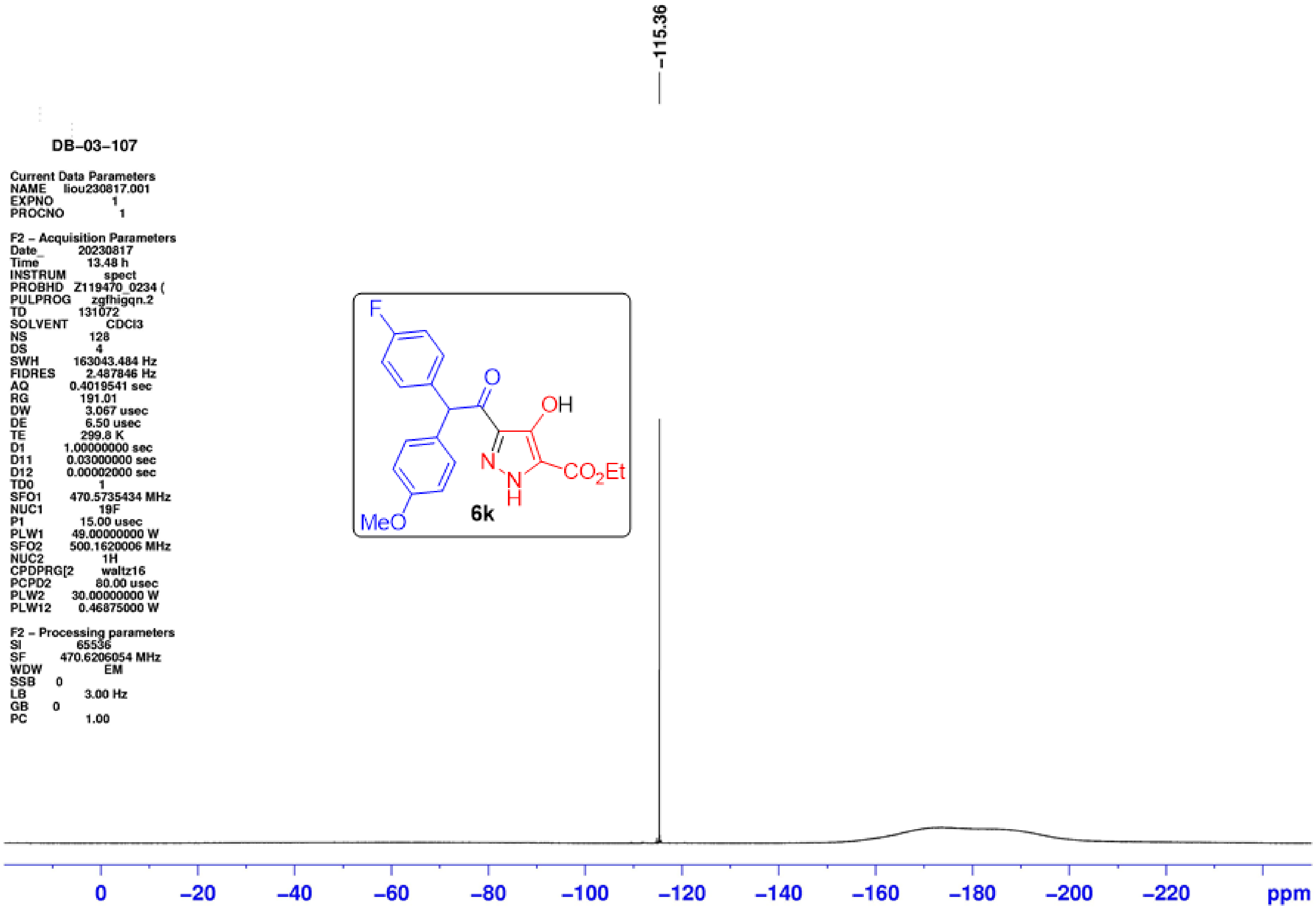

^{1}H NMR ($CDCl_3$, 400 MHz)

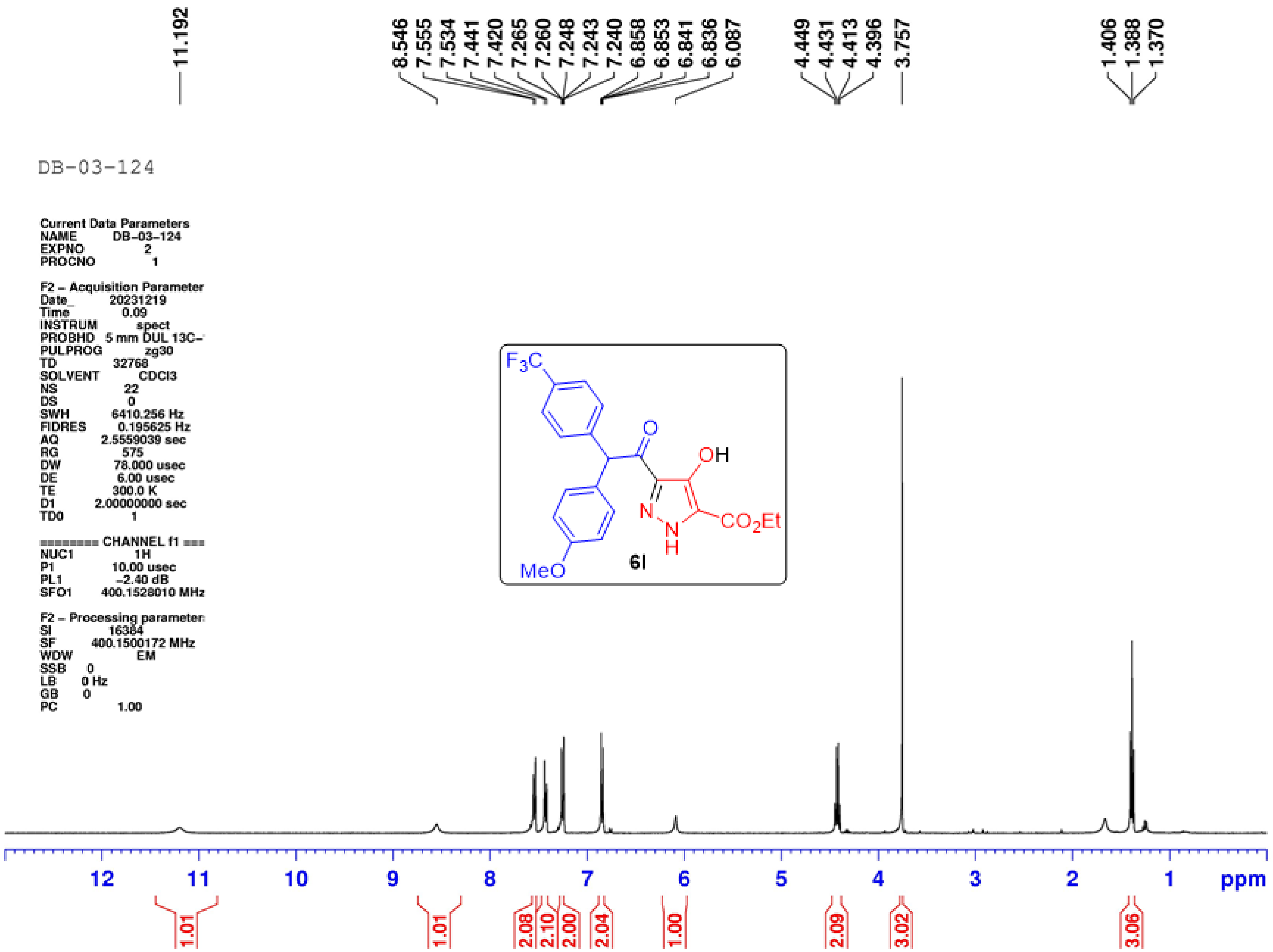

^{13}C NMR ($CDCl_3$, 100 MHz)

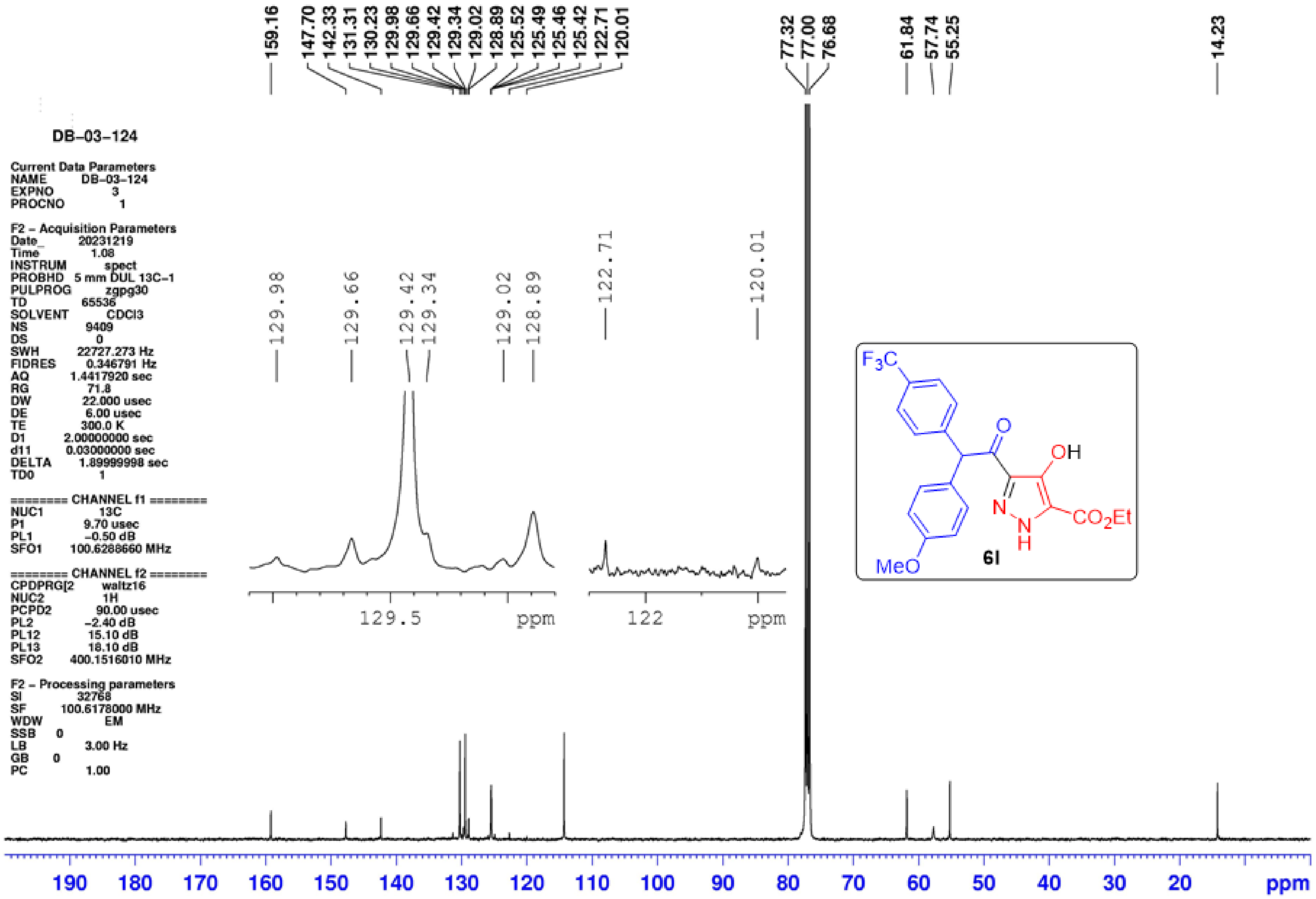

^{19}F NMR ($CDCl_3$, 471 MHz)

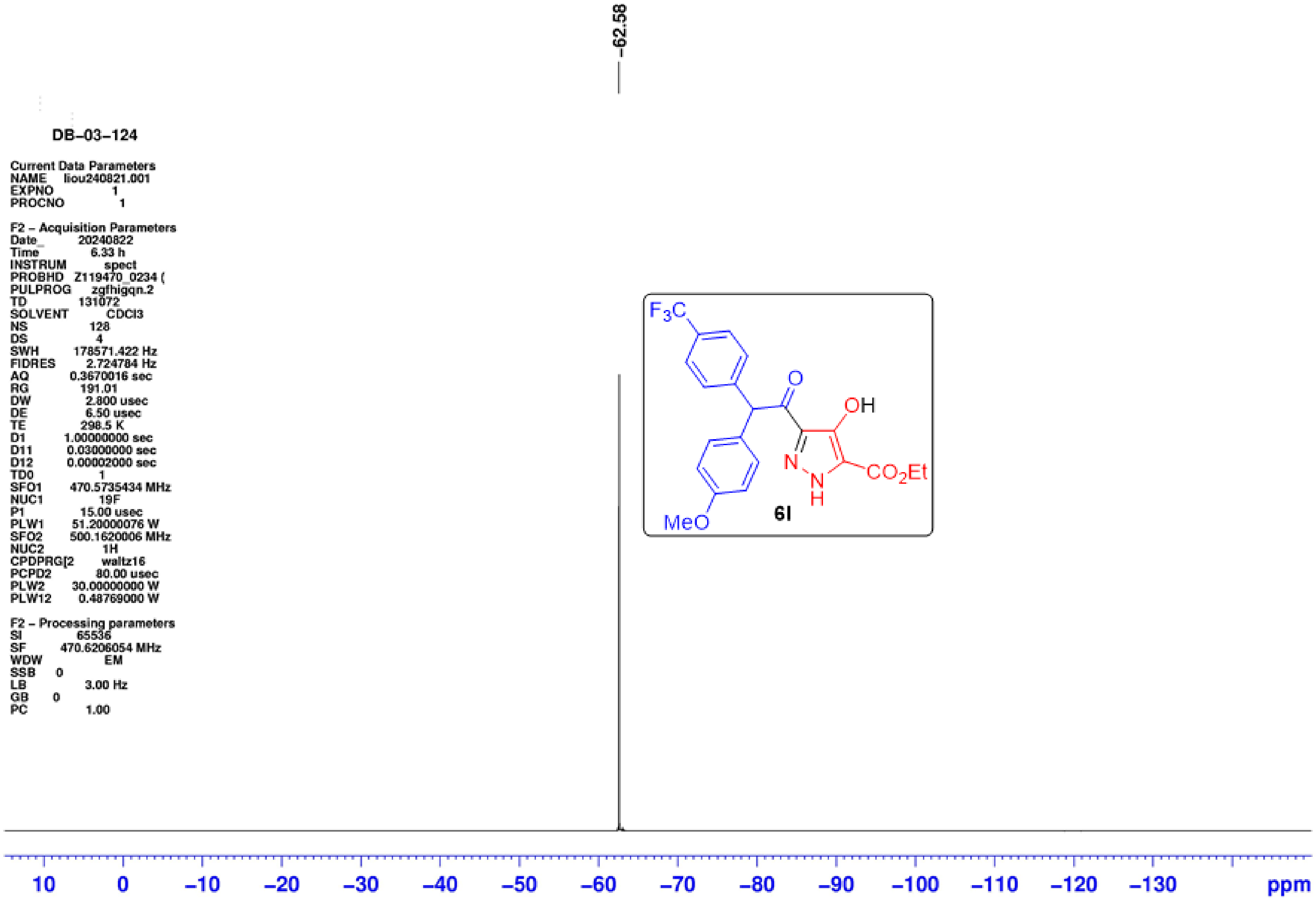

6l

^{1}H NMR ($CDCl_3$, 400 MHz)

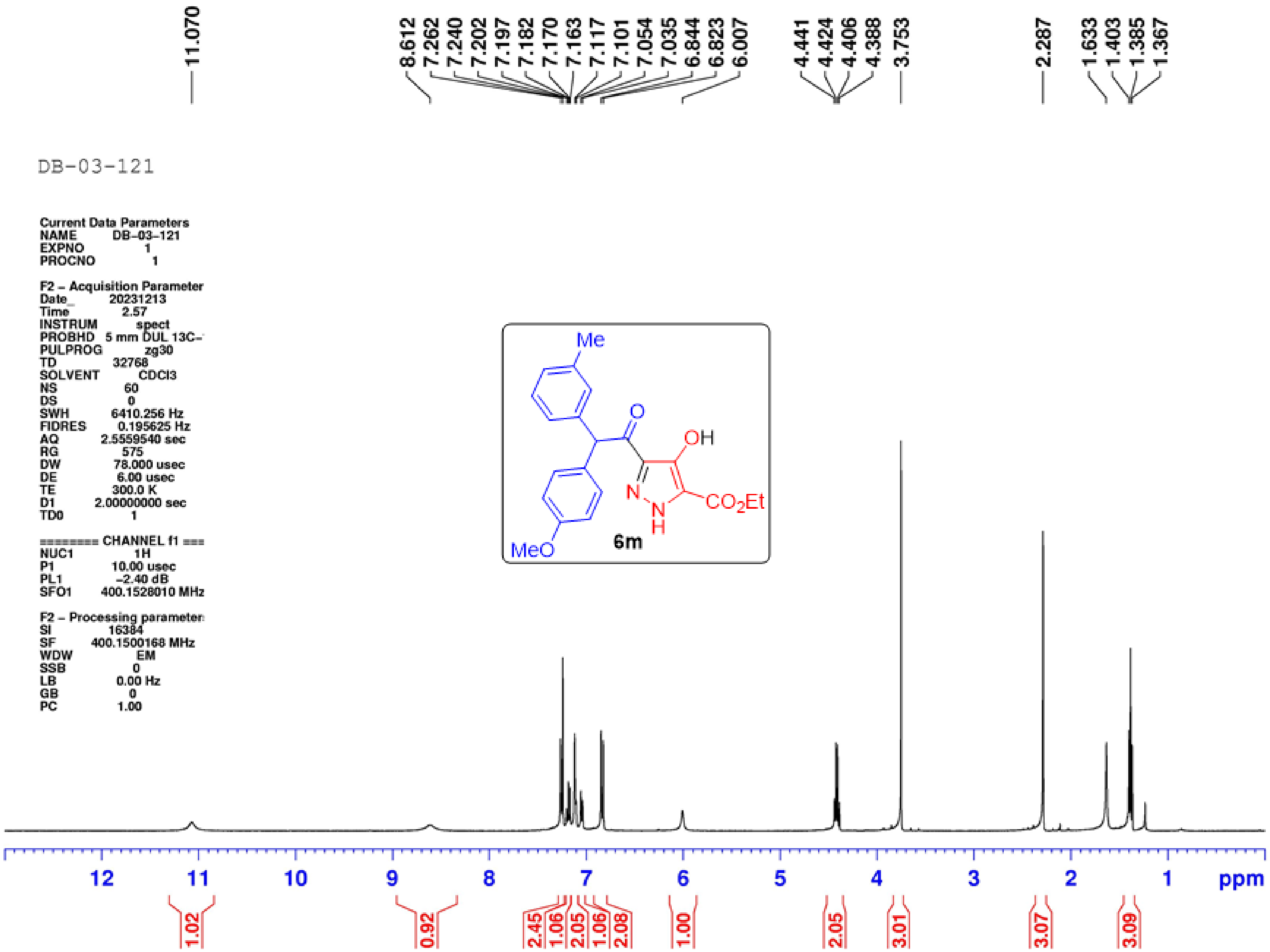

^{13}C NMR ($CDCl_3$, 100 MHz)

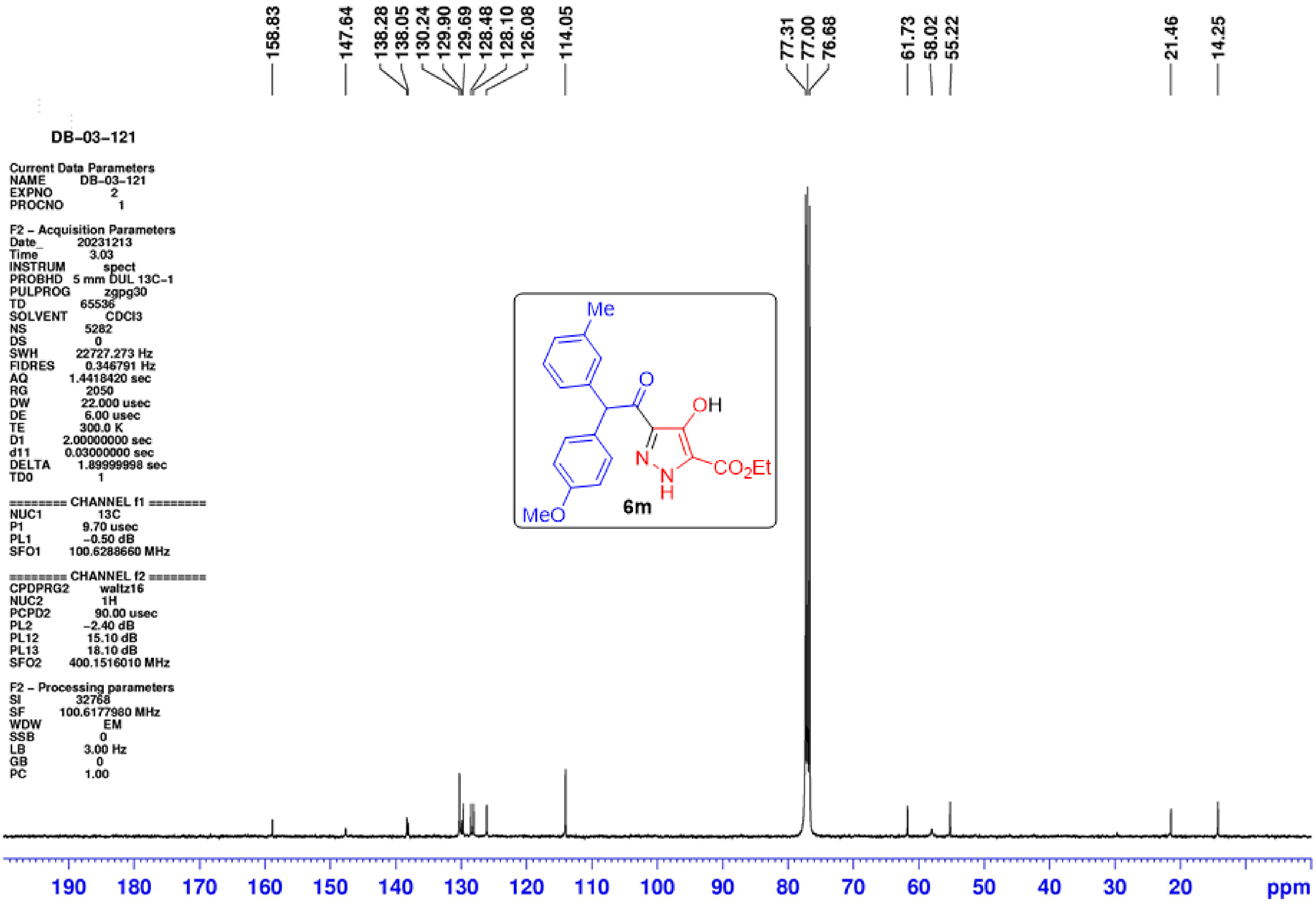

^{1}H NMR ($CDCl_3$, 400 MHz)

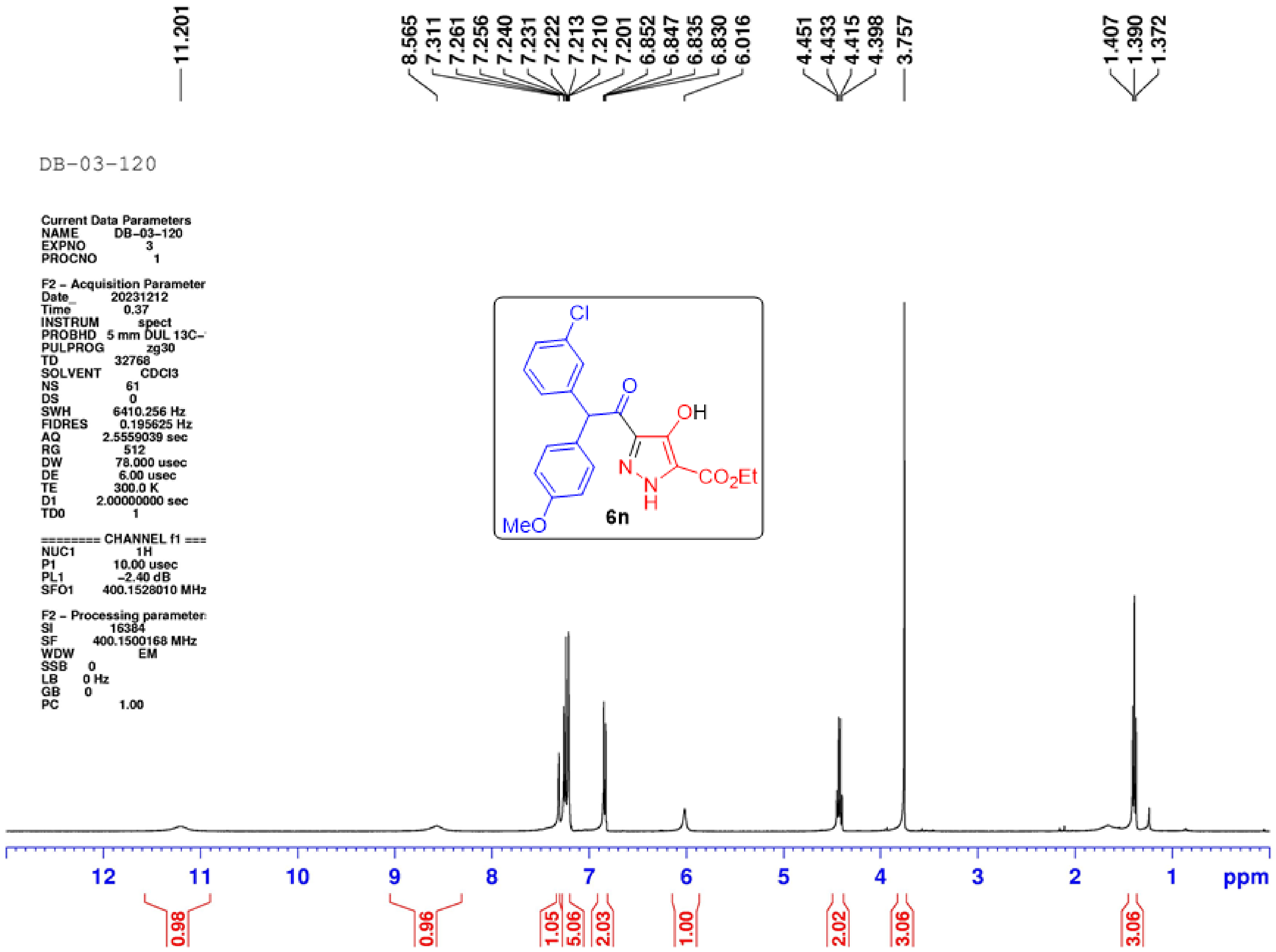

^{13}C NMR ($CDCl_3$, 100 MHz)

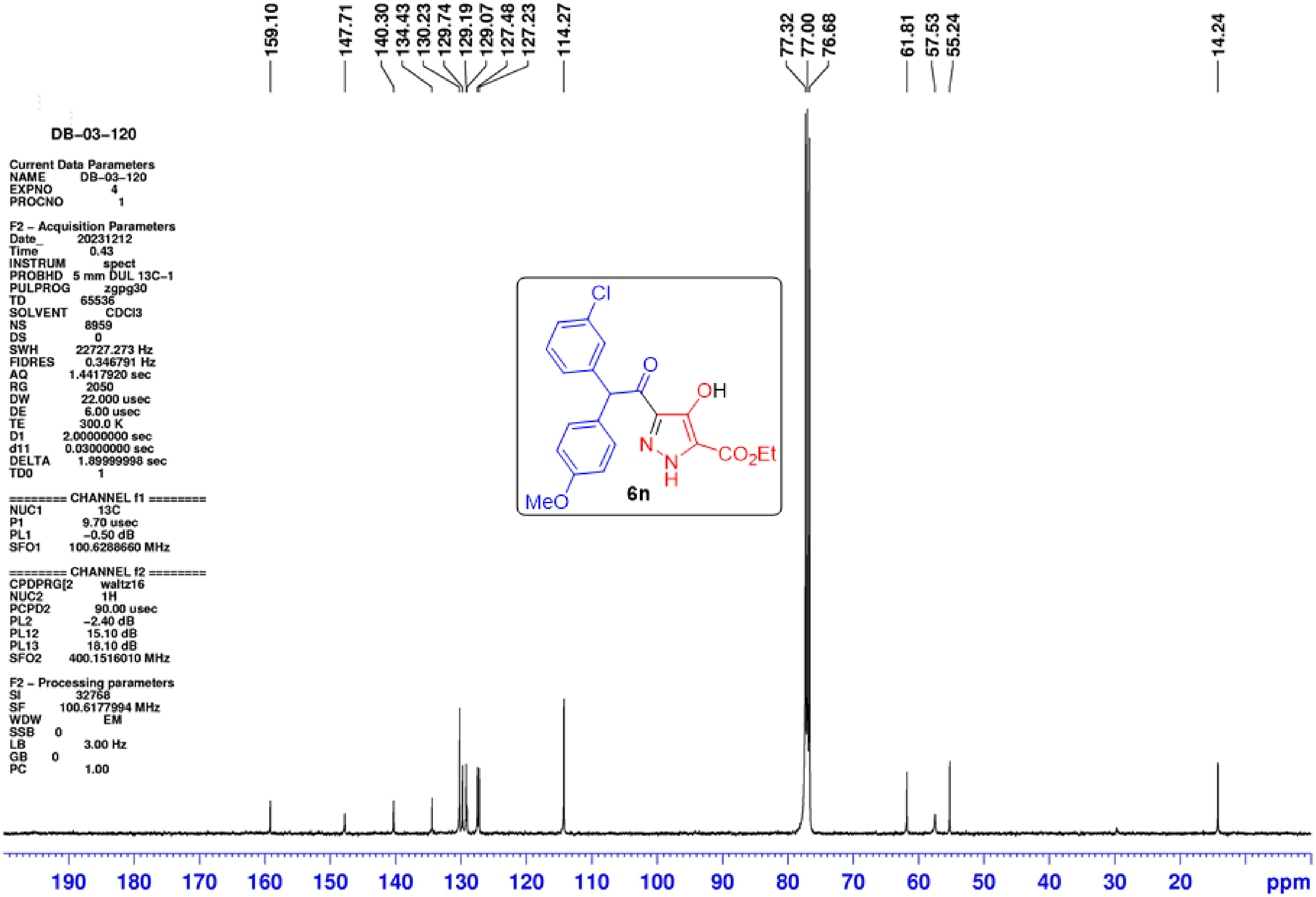

^{1}H NMR ($CDCl_3$, 400 MHz)

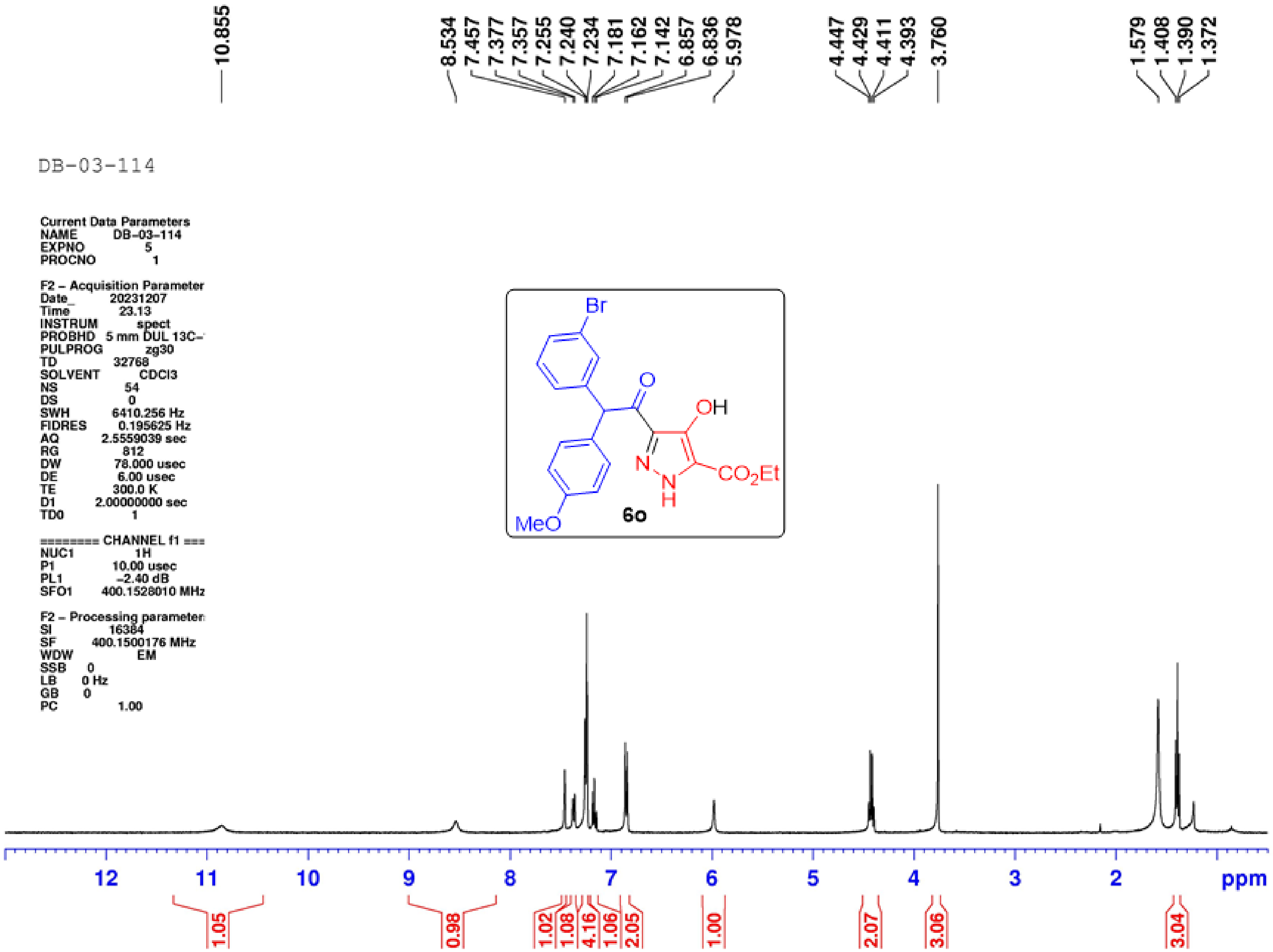

^{13}C NMR ($CDCl_3$, 100 MHz)

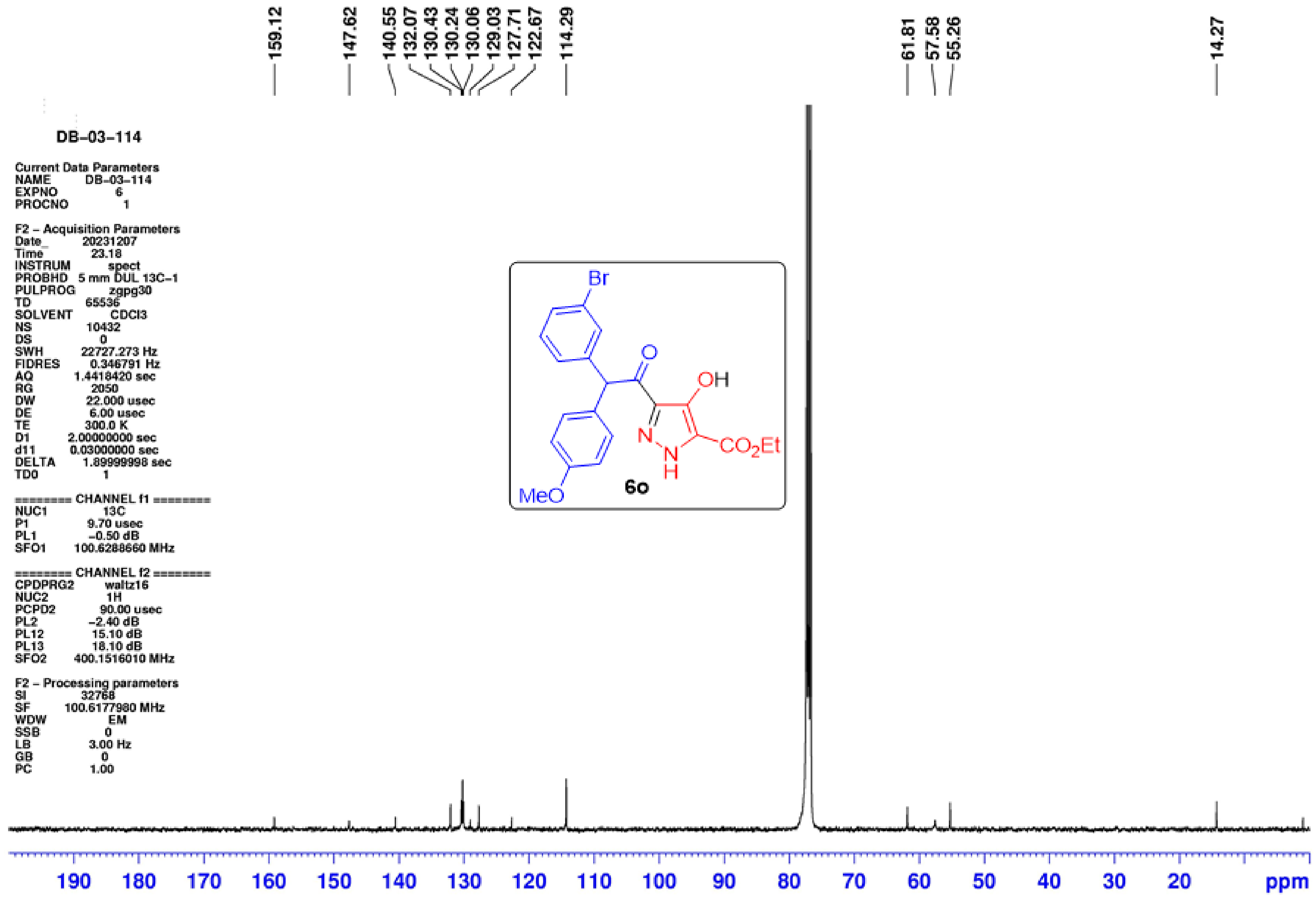

^{1}H NMR ($CDCl_3$, 700 MHz)

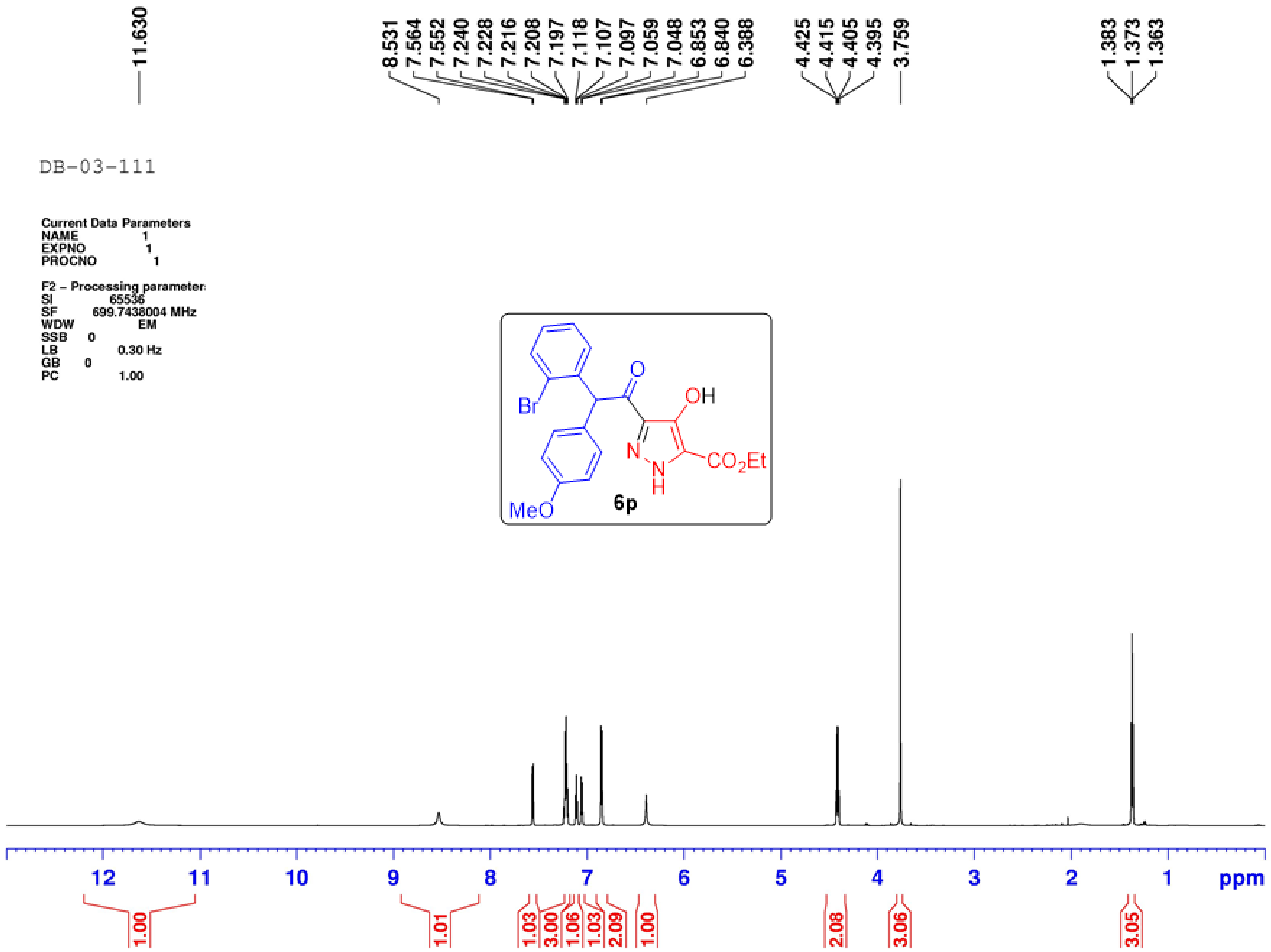

^{13}C NMR ($CDCl_3$, 175 MHz)

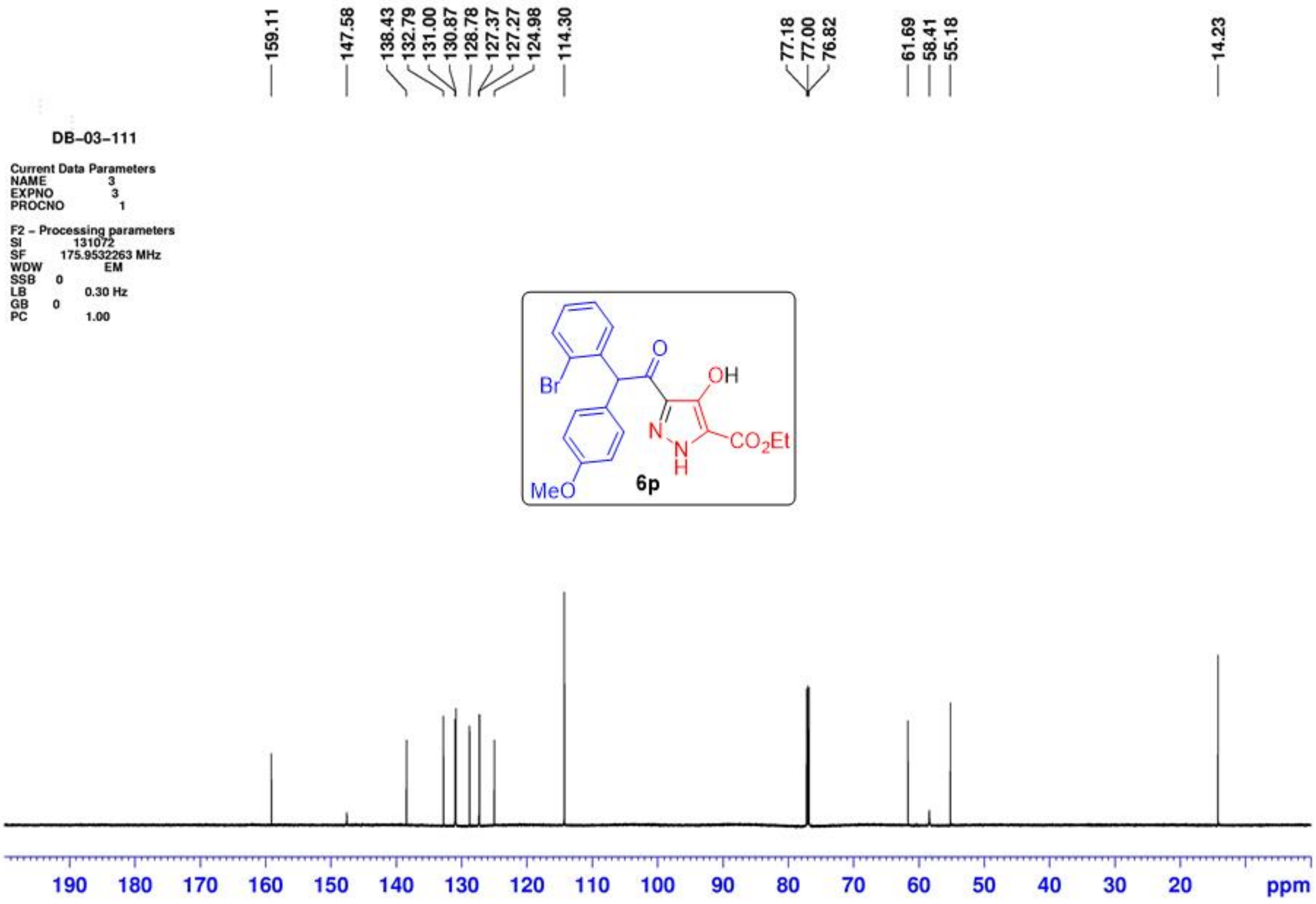

^{1}H NMR ($CDCl_3$, 700 MHz)

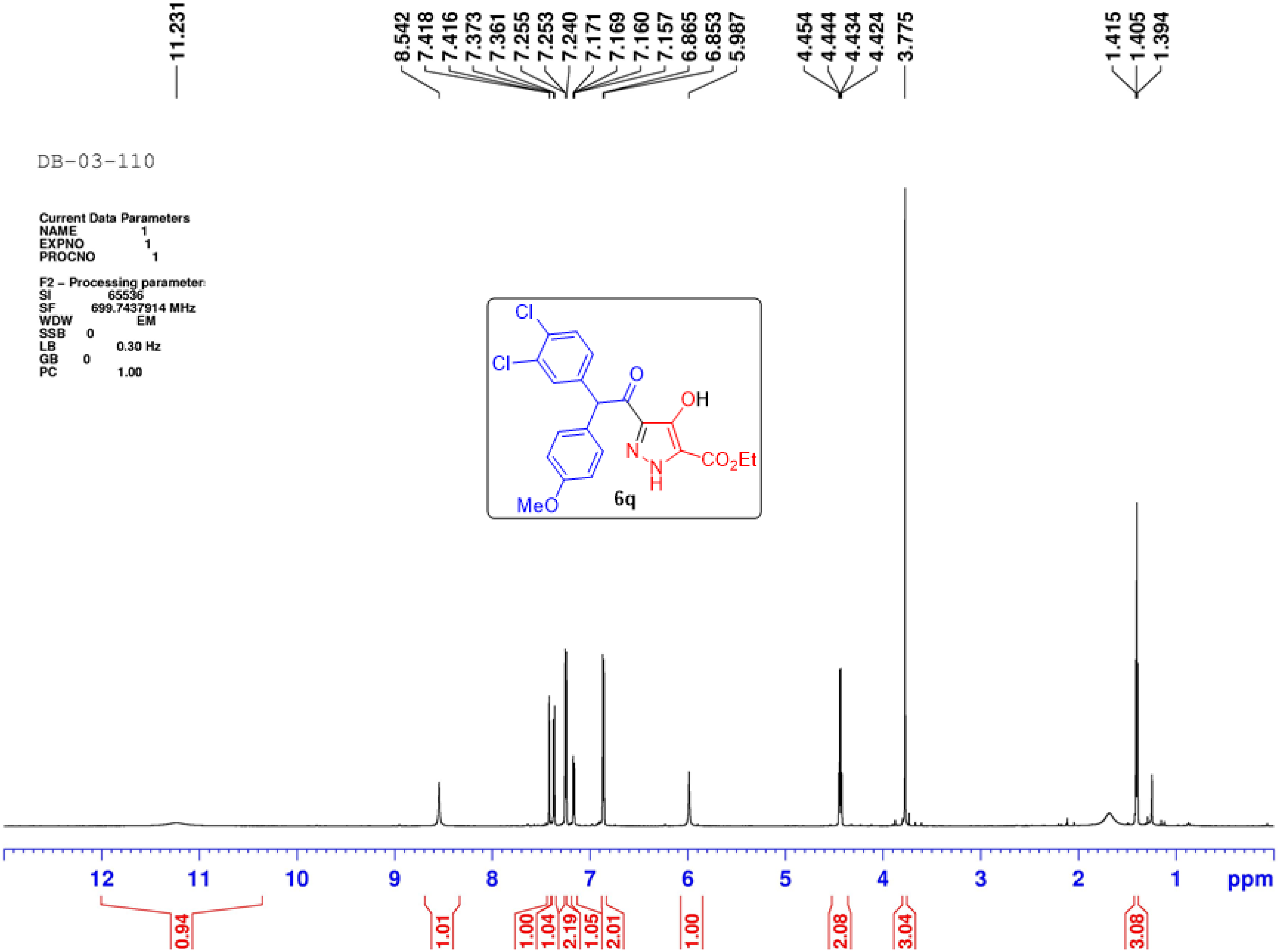

^{13}C NMR ($CDCl_3$, 175 MHz)

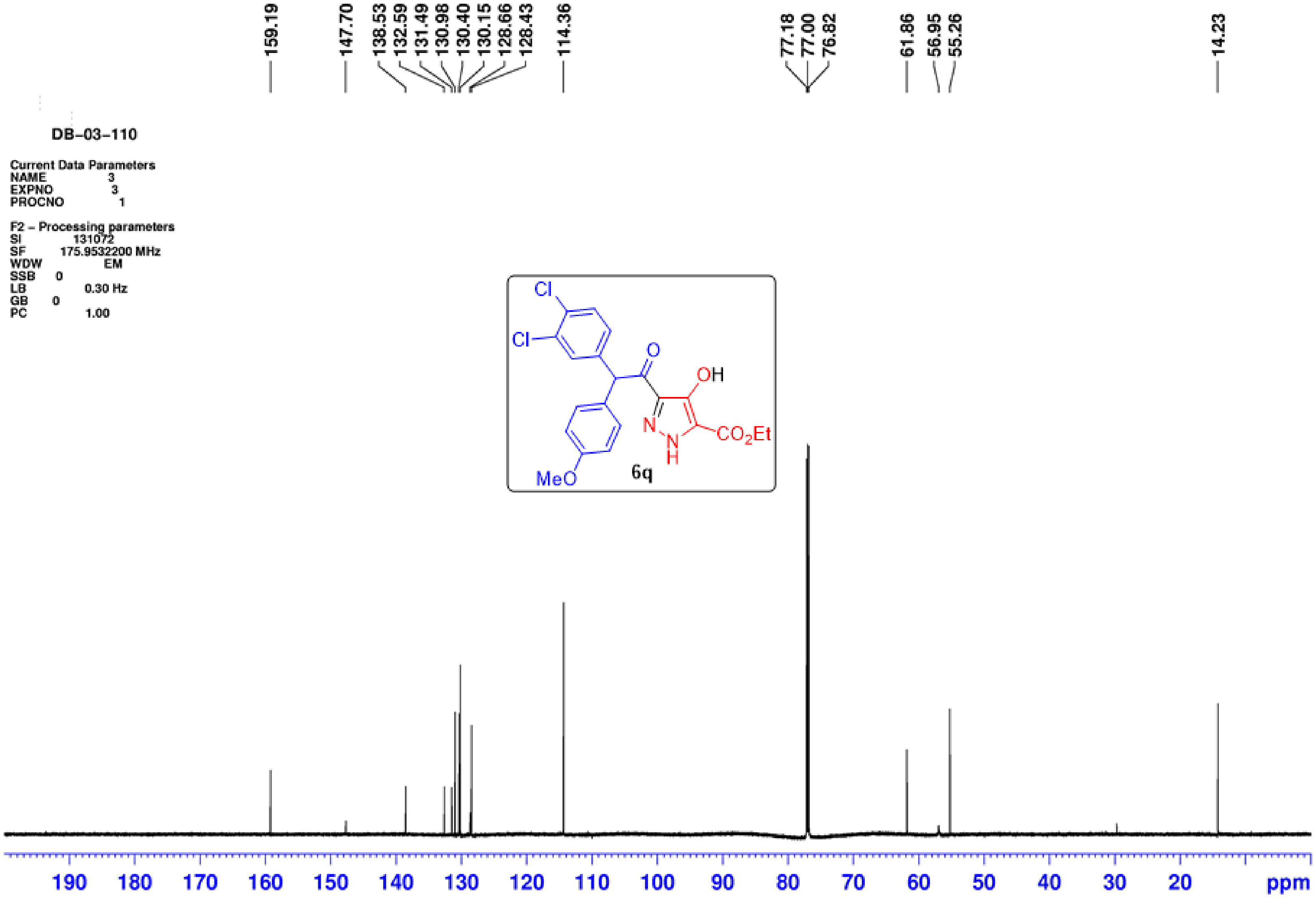

^{1}H NMR (CD_2Cl_2, 700 MHz)

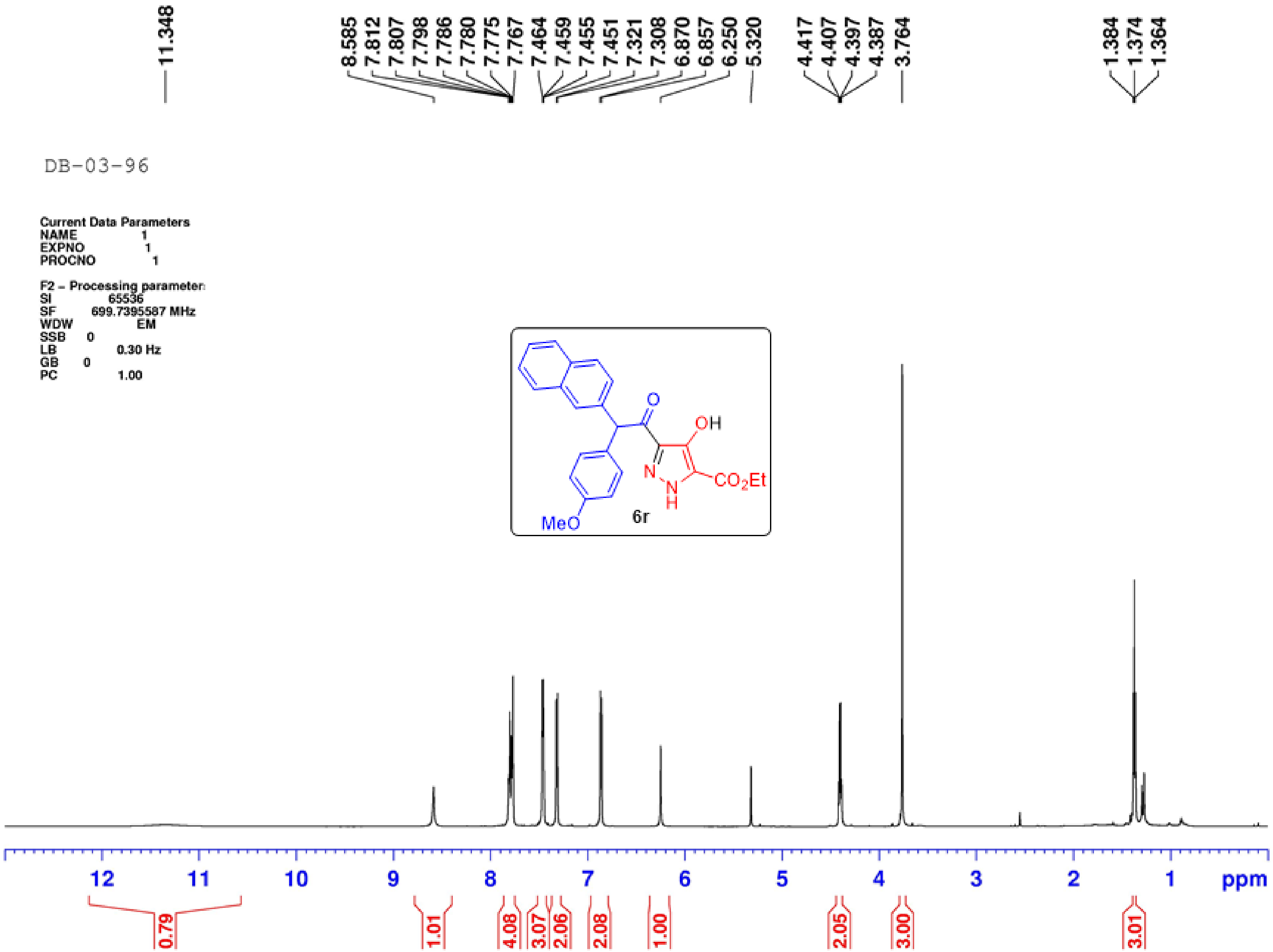

^{13}C NMR (CD_2Cl_2, 175 MHz)

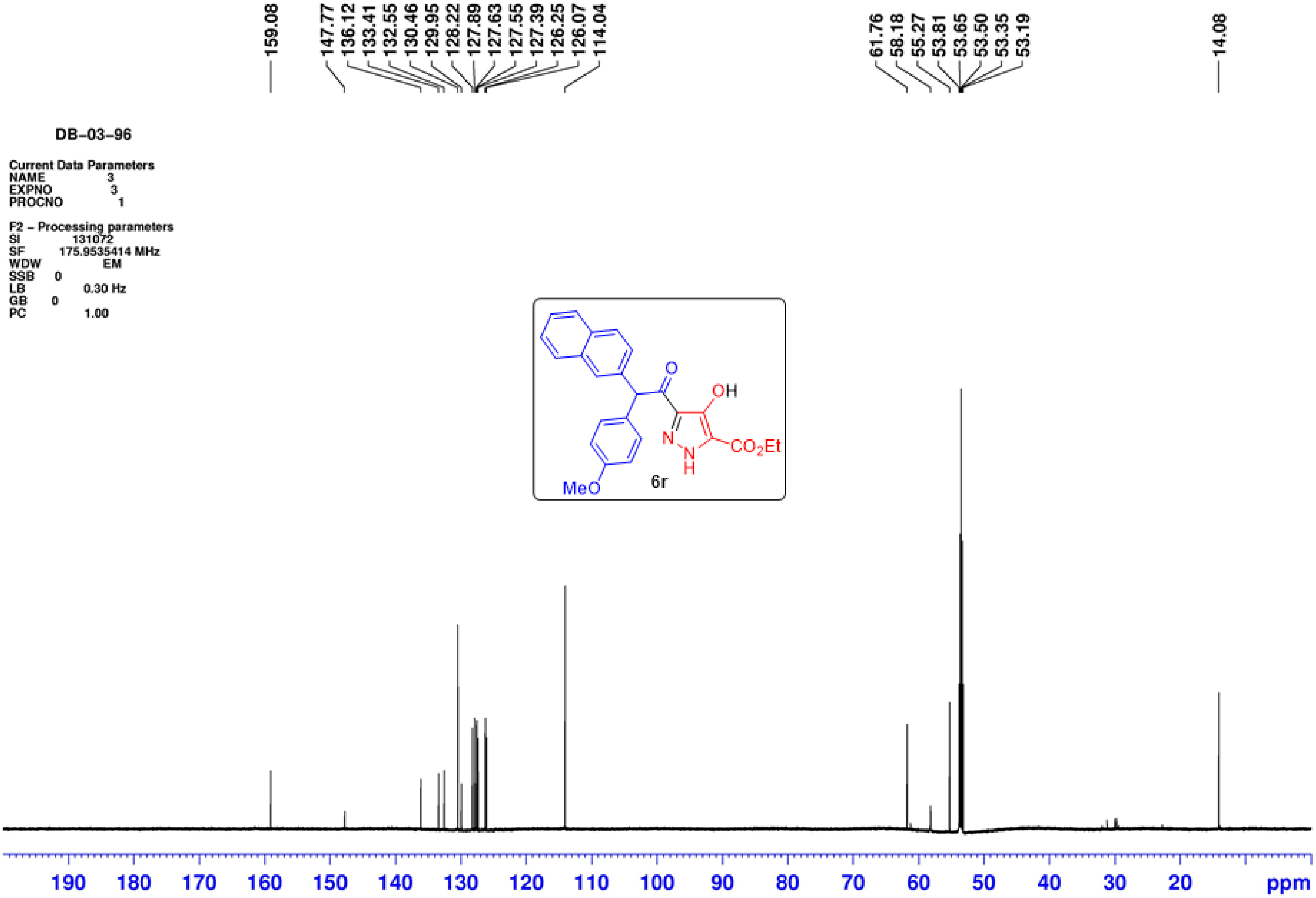

^{1}H NMR ($CDCl_3$, 700 MHz)

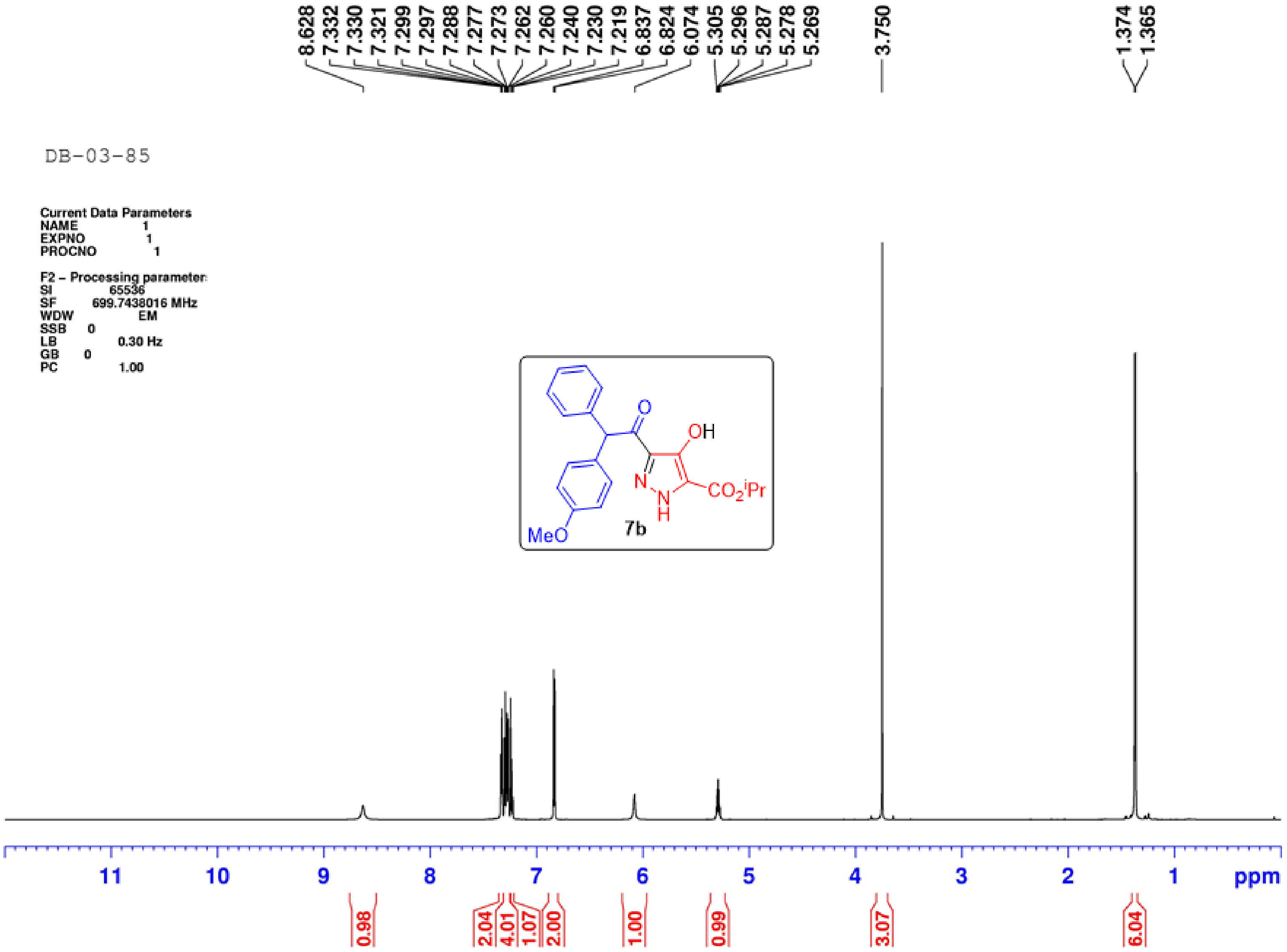

^{13}C NMR ($CDCl_3$, 175 MHz)

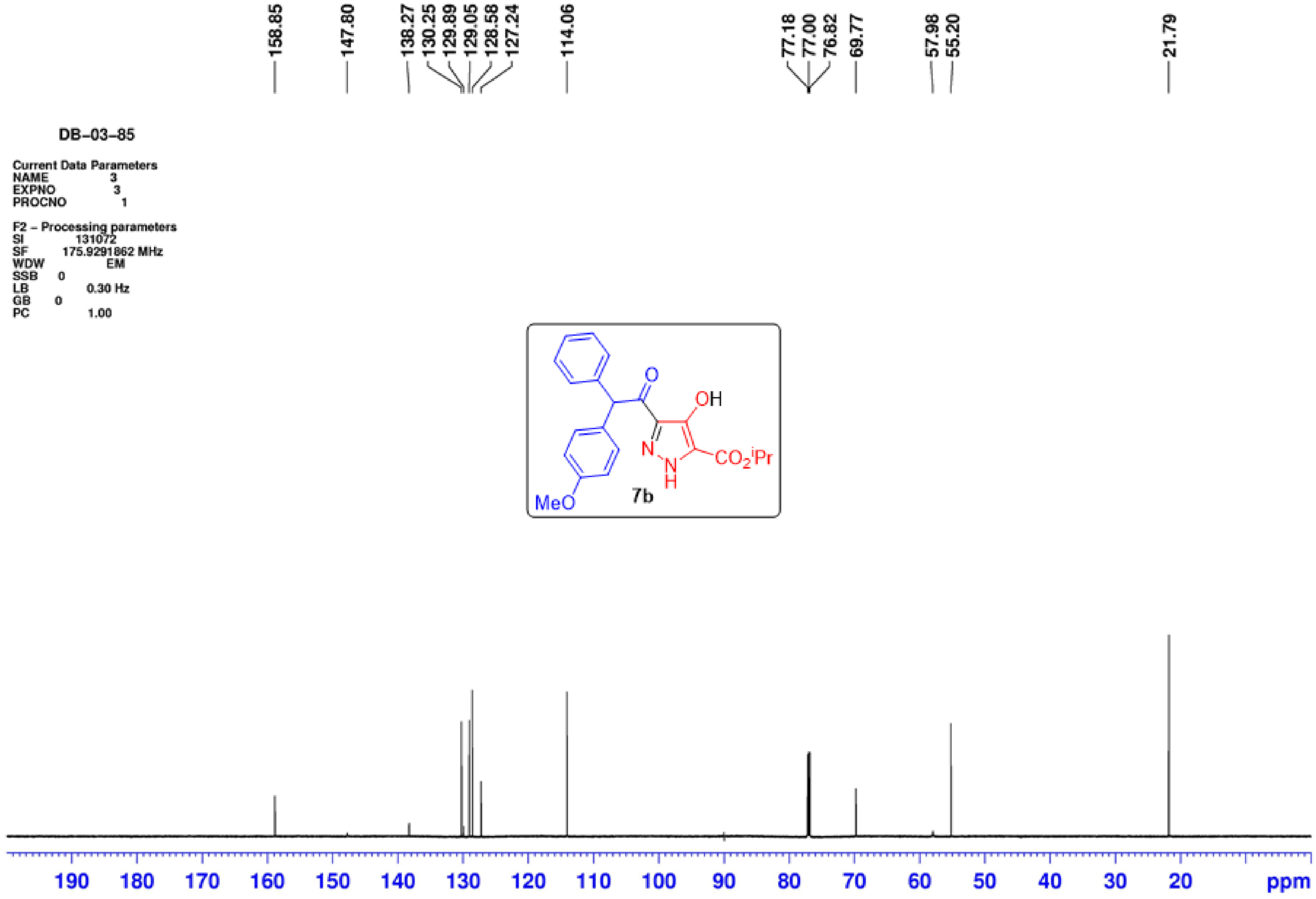

^{1}H NMR ($CDCl_3$, 700 MHz)

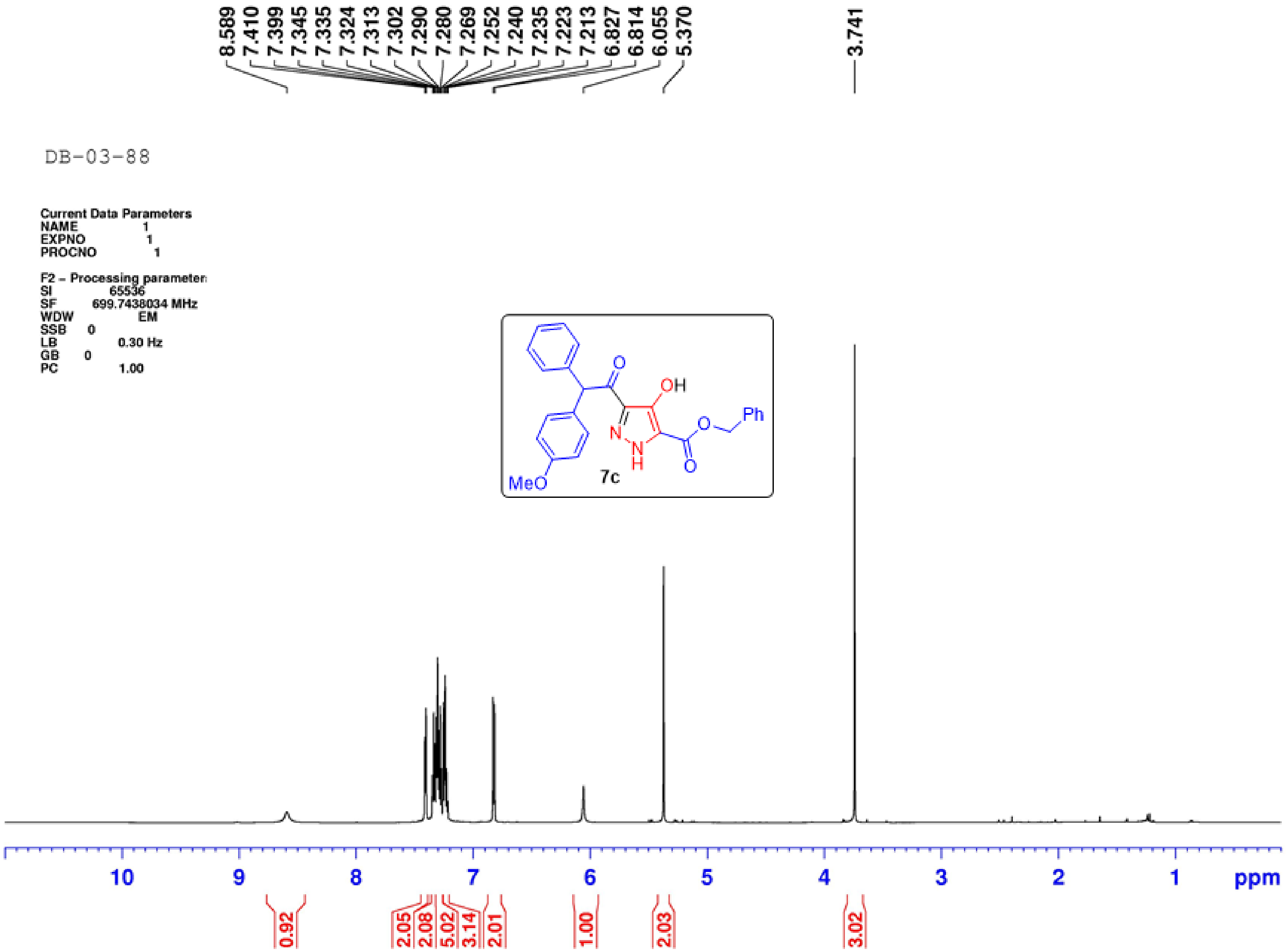

^{13}C NMR (CDCl$_3$, 175 MHz)

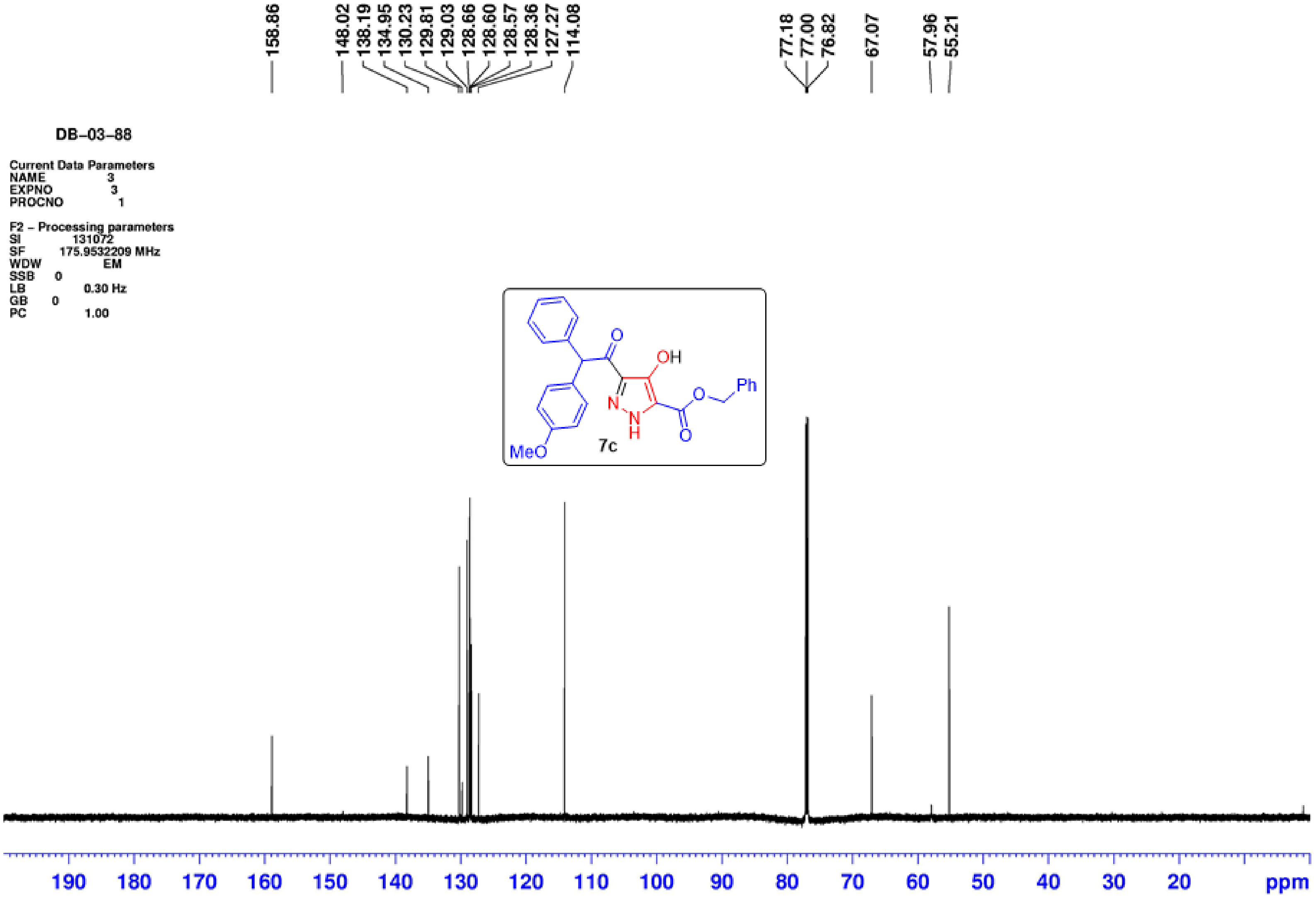

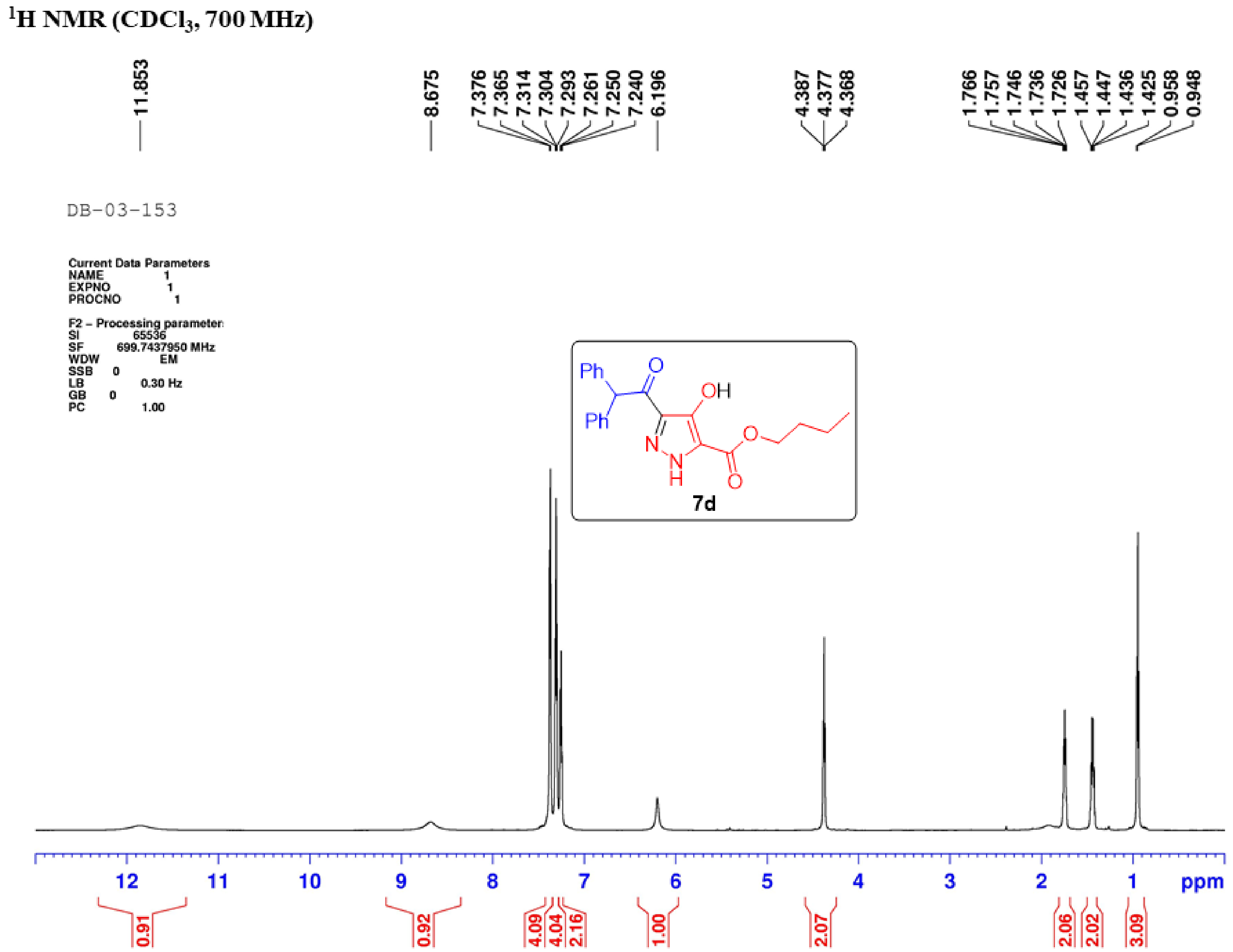

^{1}H NMR (CDCl3, 700 MHz)
11.853
8.675
7.376
7.365
7.314
7.304
7.293
7.261
7.250
7.240
6.196
4.387
4.377
4.368
1.766
1.757
1.746
1.736
1.726
1.457
1.447
1.436
1.425
0.958
0.948
DB-03-153
Current Data Parameters
NAME 1
EXPNO 1
PROCNO 1
F2 - Processing parameter
SI 65536
SF 699.7437950 MHz
WDW EM
SSB 0
LB 0.30 Hz
GB 0
PC 1.00
Ph
O
OH
Ph
N
N
H
O
O
7d
12
11
10
9
8
7
6
5
4
3
2
1
ppm
0.91
0.92
4.09
4.04
2.16
1.00
2.07
2.06
2.02
3.09

^{13}C NMR ($CDCl_3$, 175 MHz)

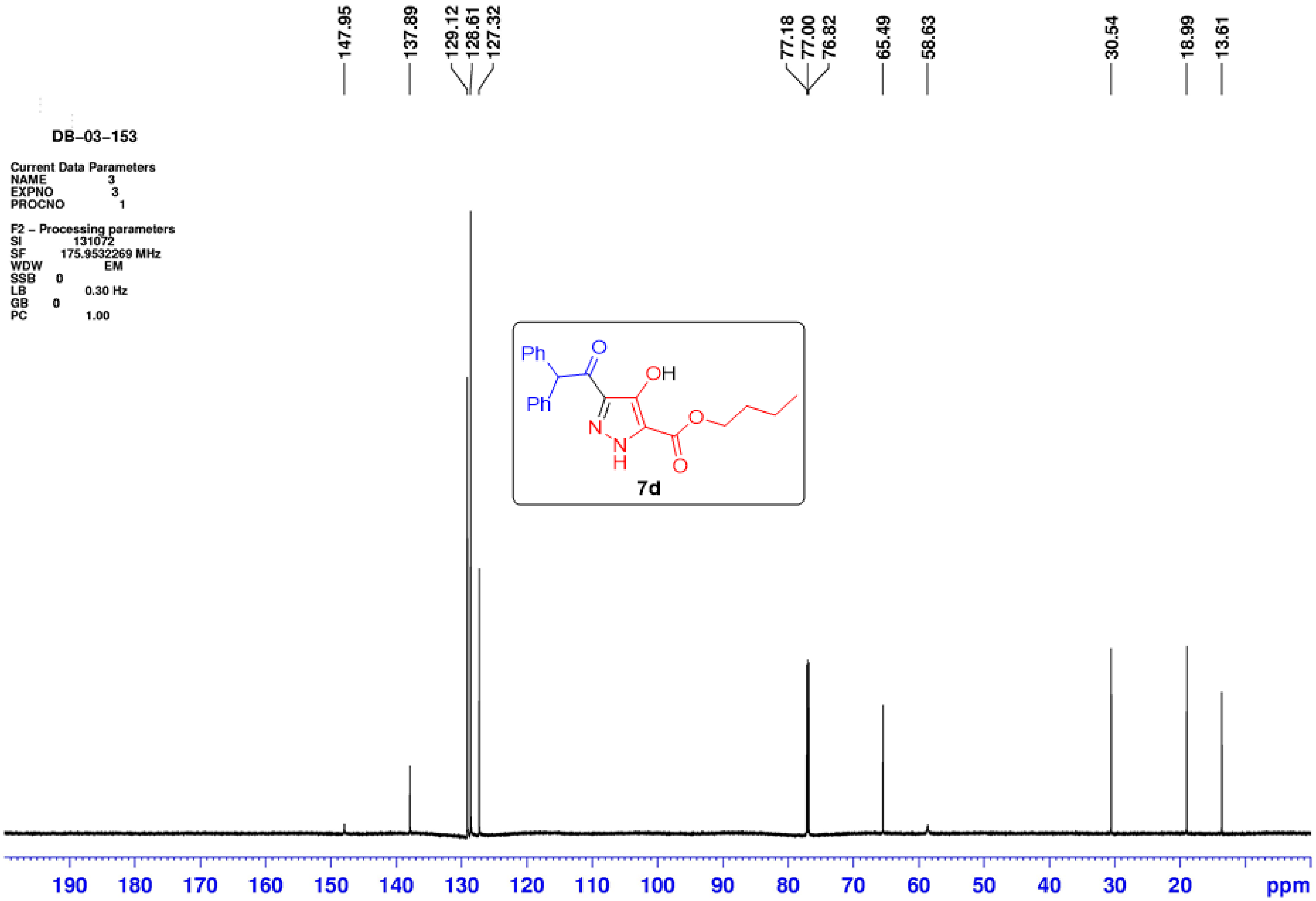

^{1}H NMR ($CDCl_3$, 700 MHz)

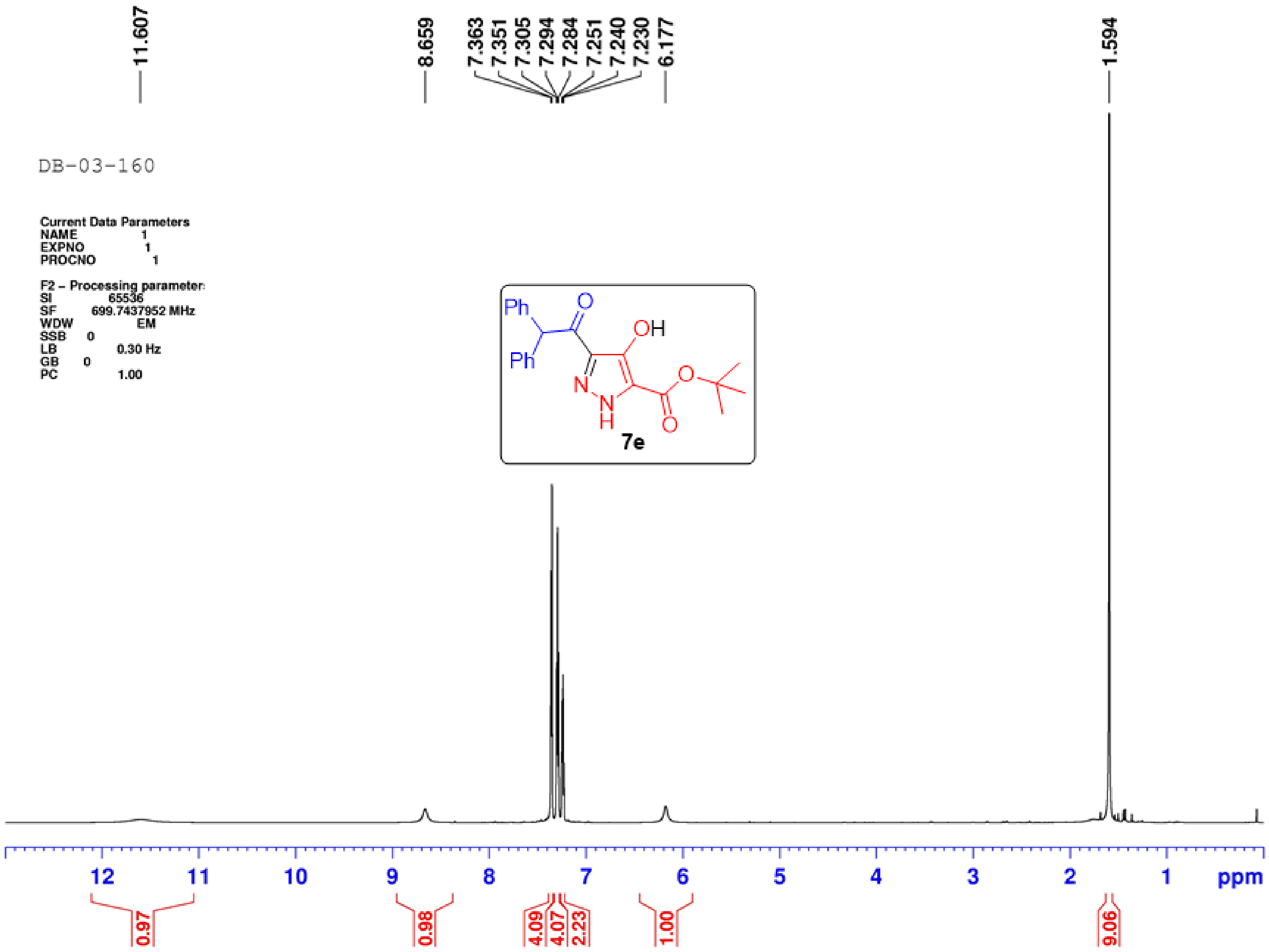

^{13}C NMR (CDCl$_3$, 175 MHz)

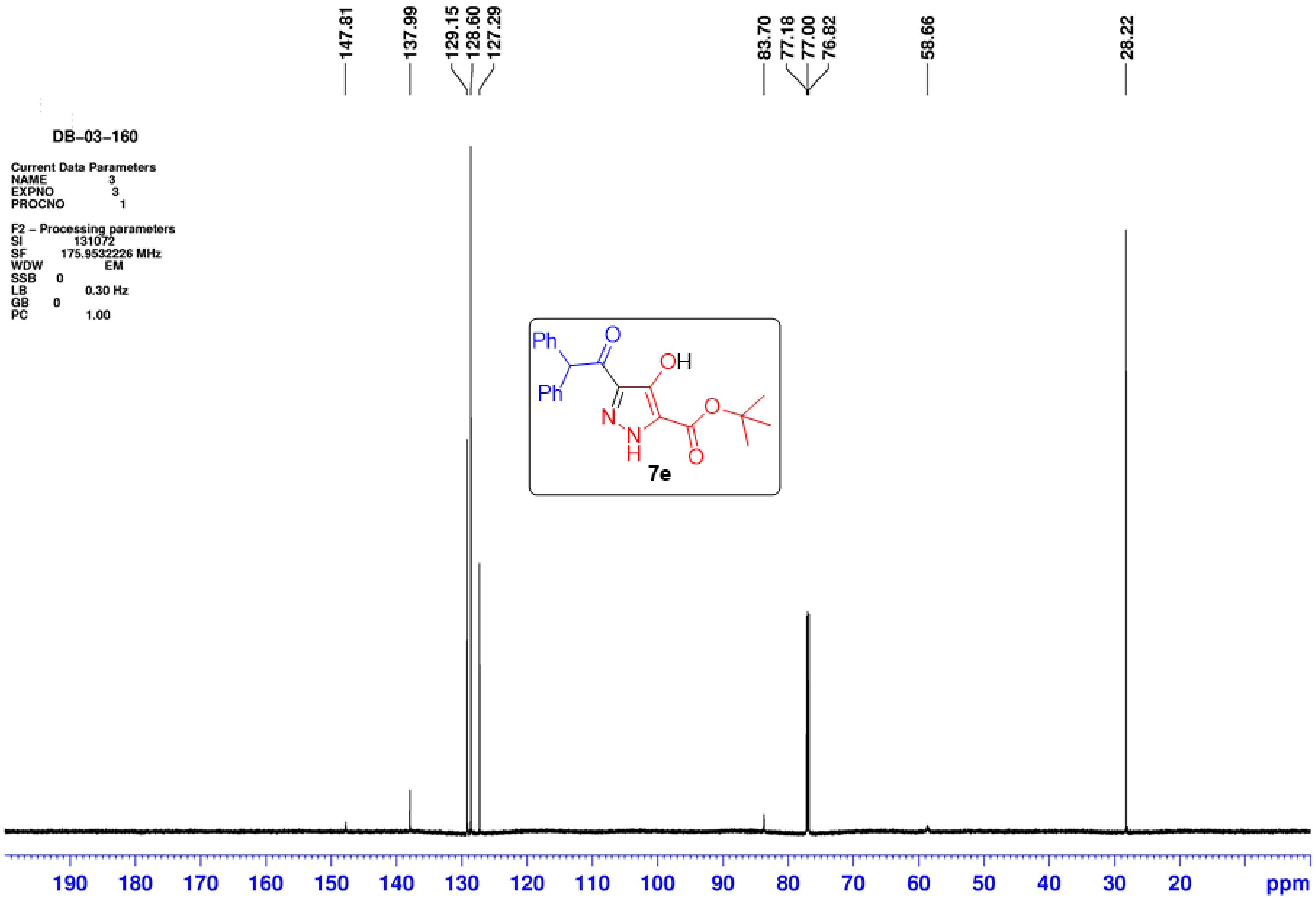

^{1}H NMR ($CDCl_3$, 700 MHz)

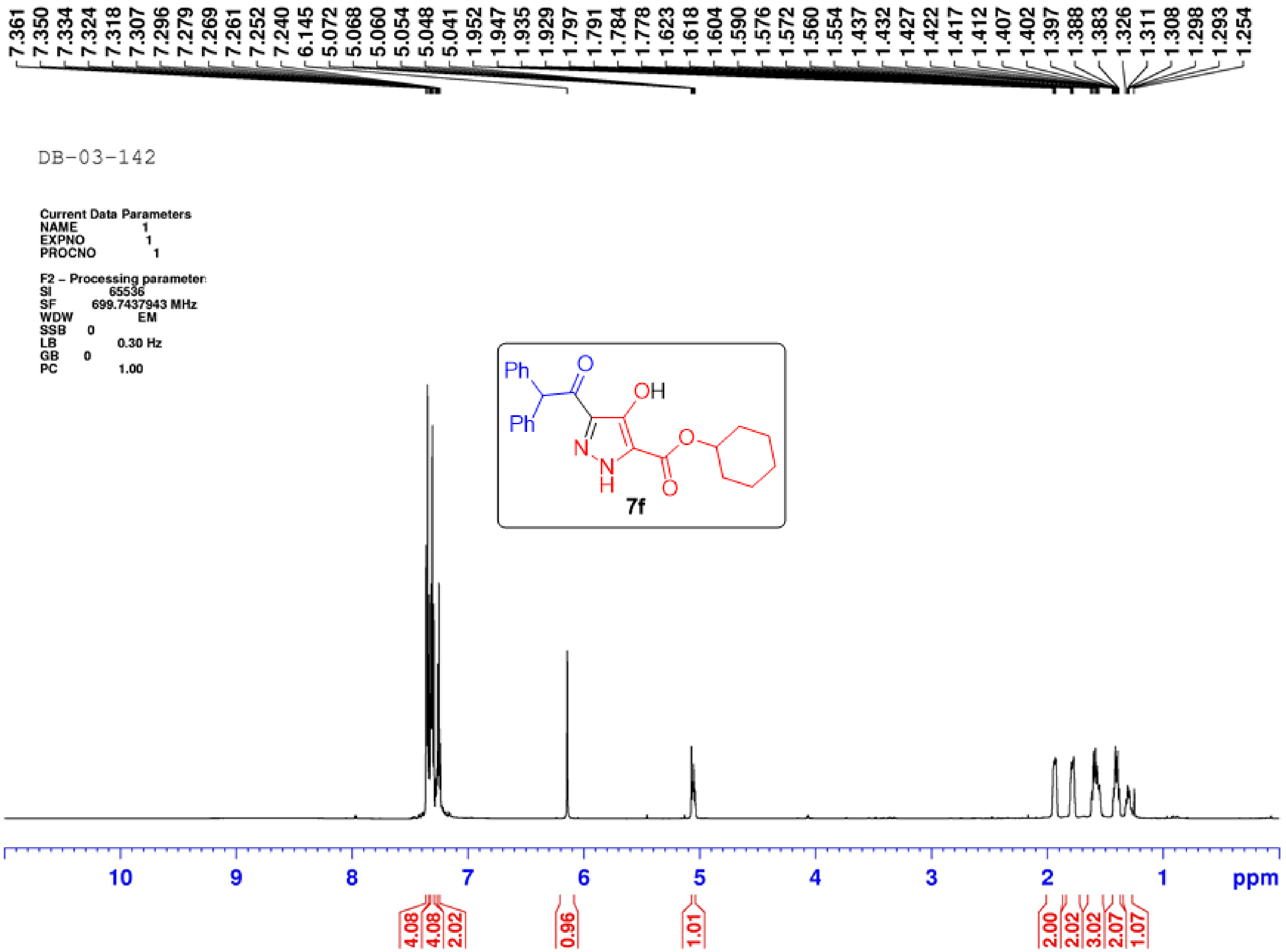

^{13}C NMR (CDCl$_3$, 175 MHz)

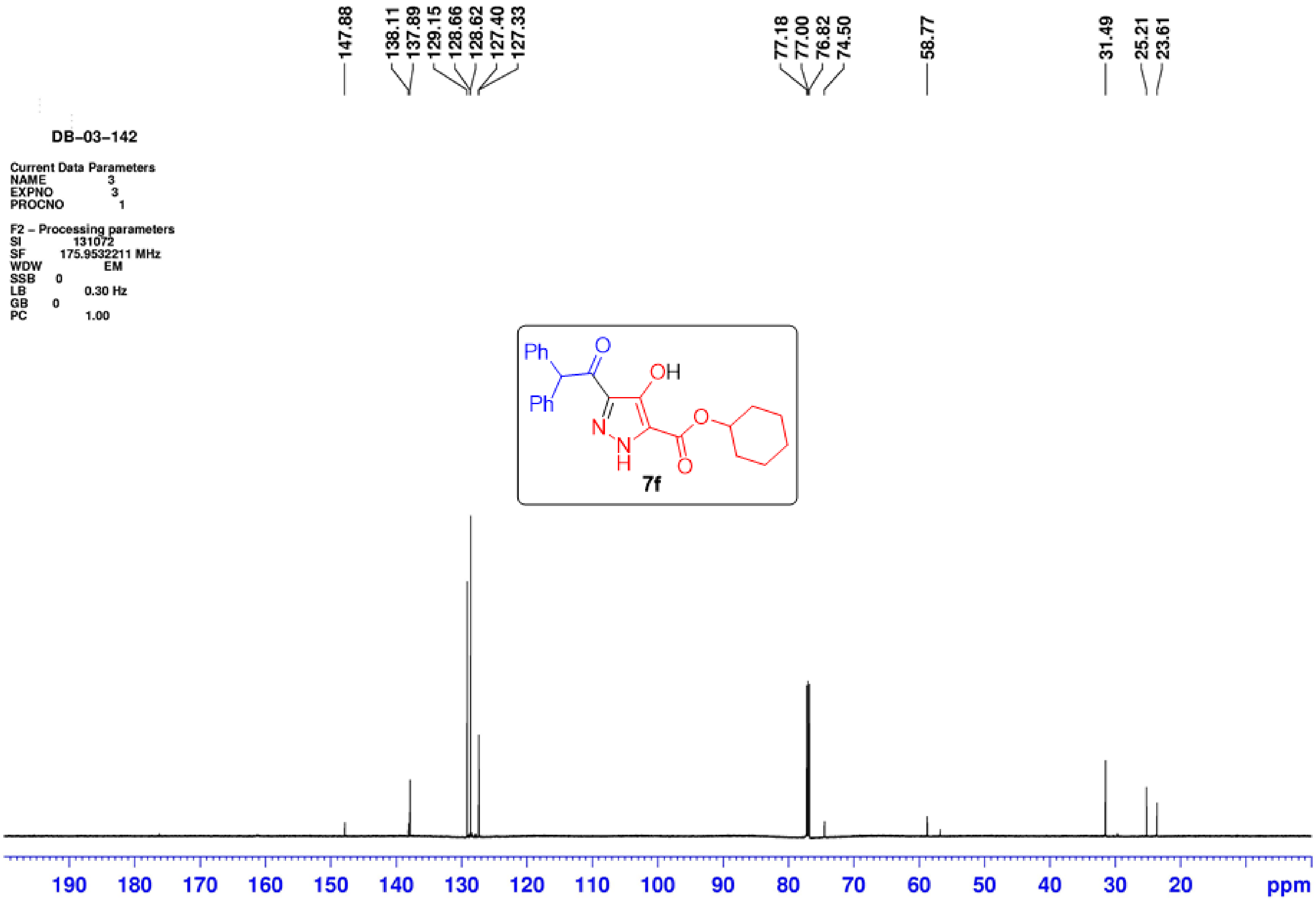

^{1}H NMR ($CDCl_3$, 700 MHz)

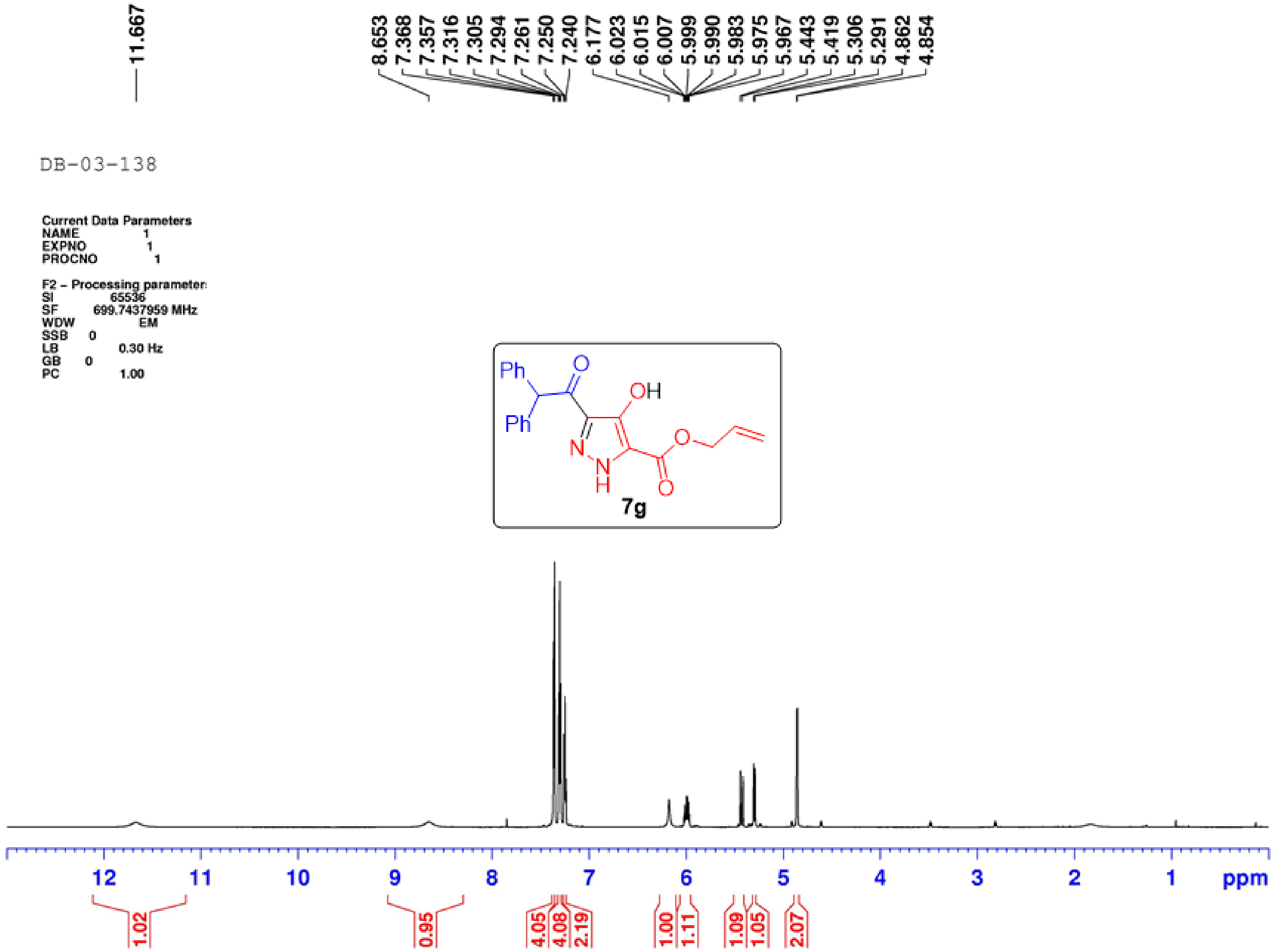

^{13}C NMR ($CDCl_3$, 175 MHz)

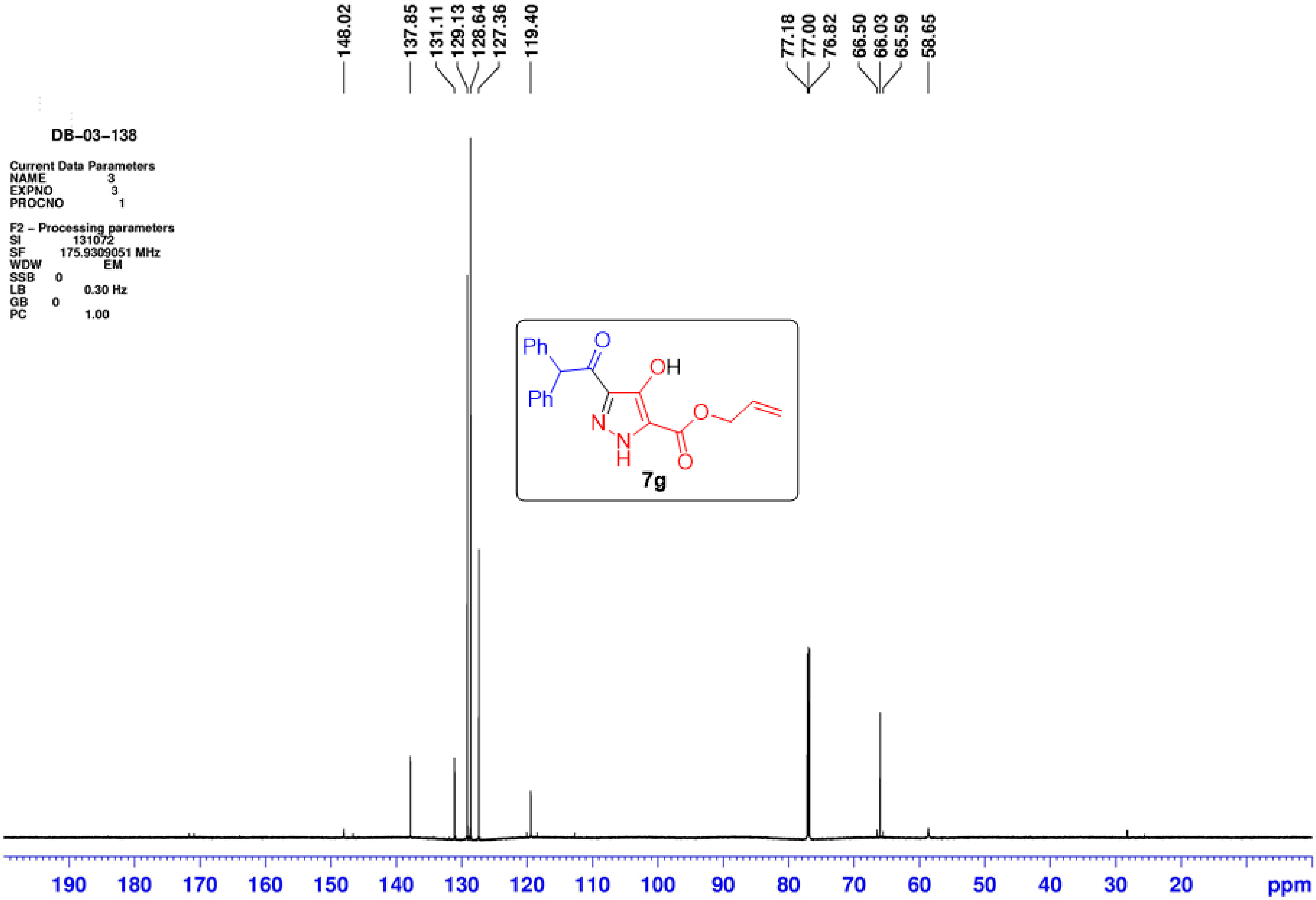

^{1}H NMR ($CDCl_3$, 700 MHz)

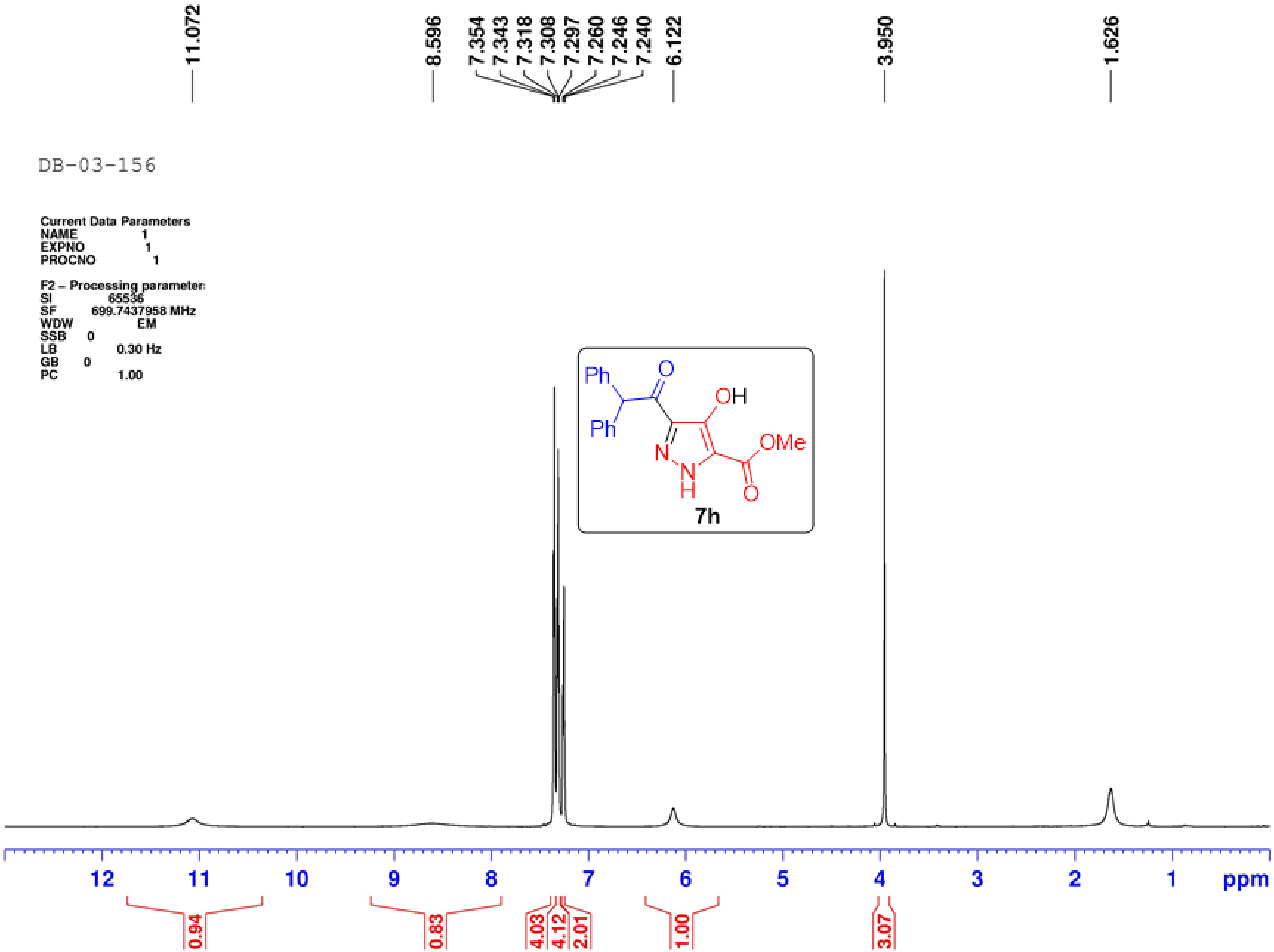

^{13}C NMR ($CDCl_3$, 175 MHz)

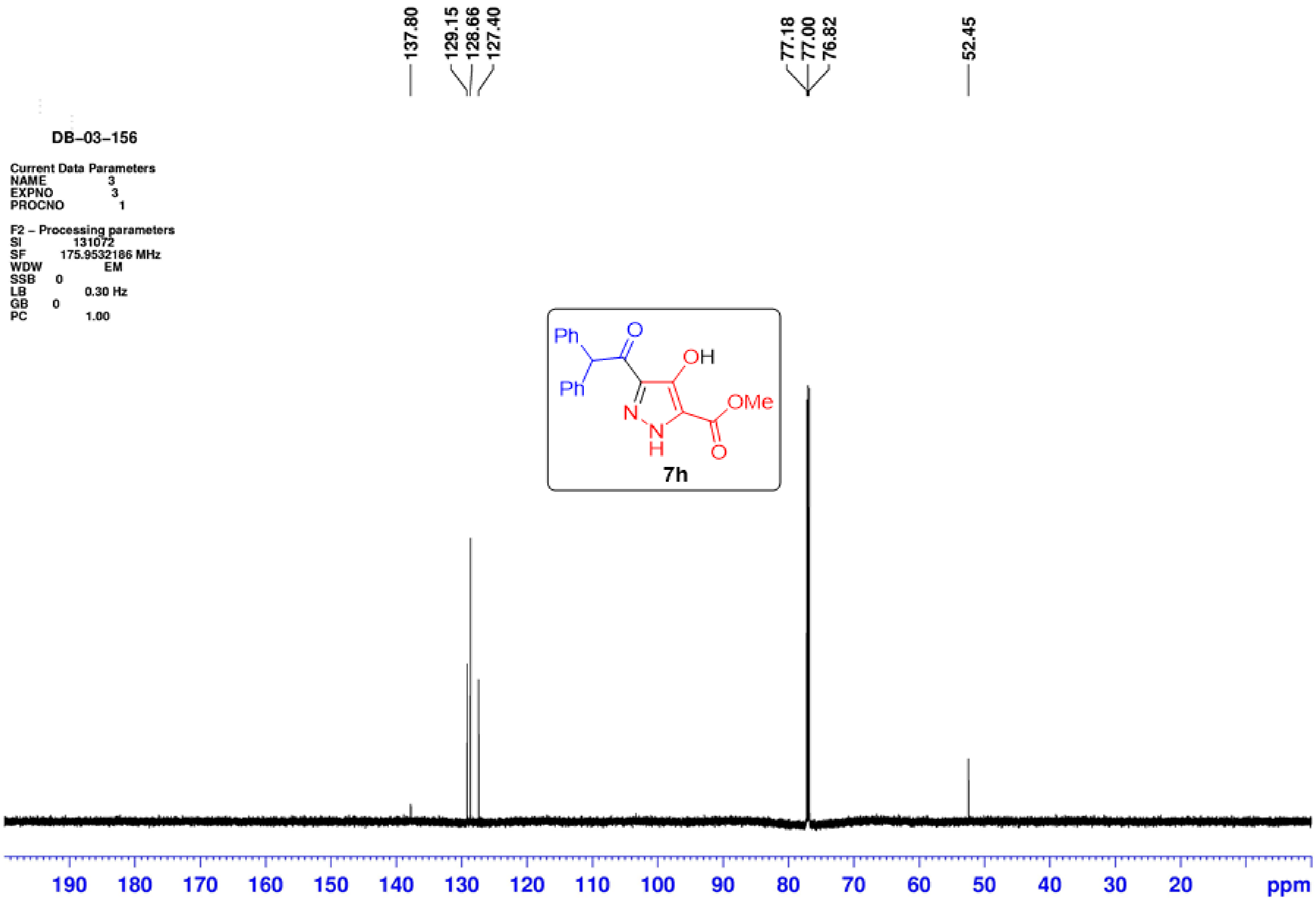

^{1}H NMR ($CDCl_3$, 700 MHz)

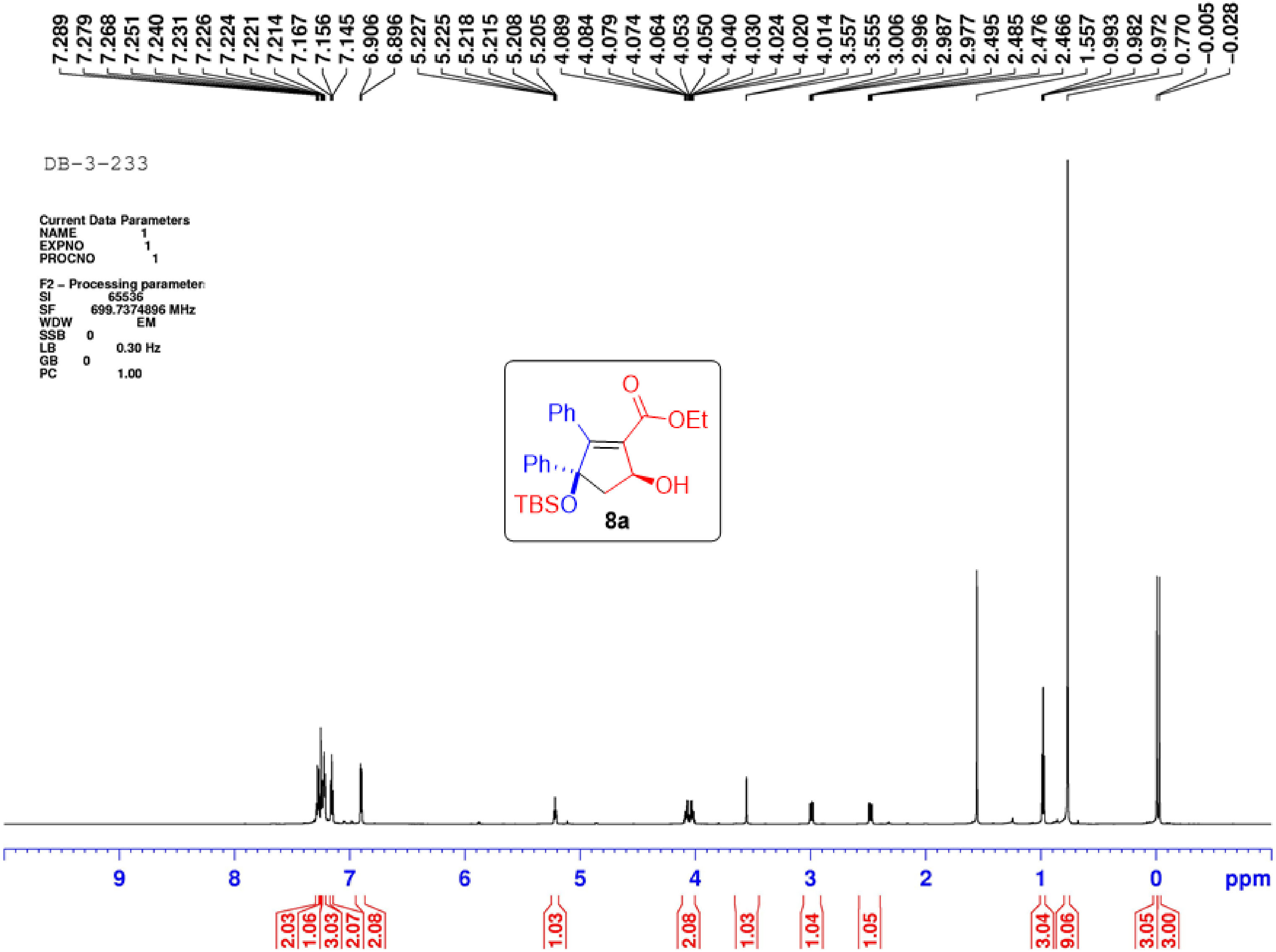

DEPT NMR ($CDCl_3$, 175 MHz)

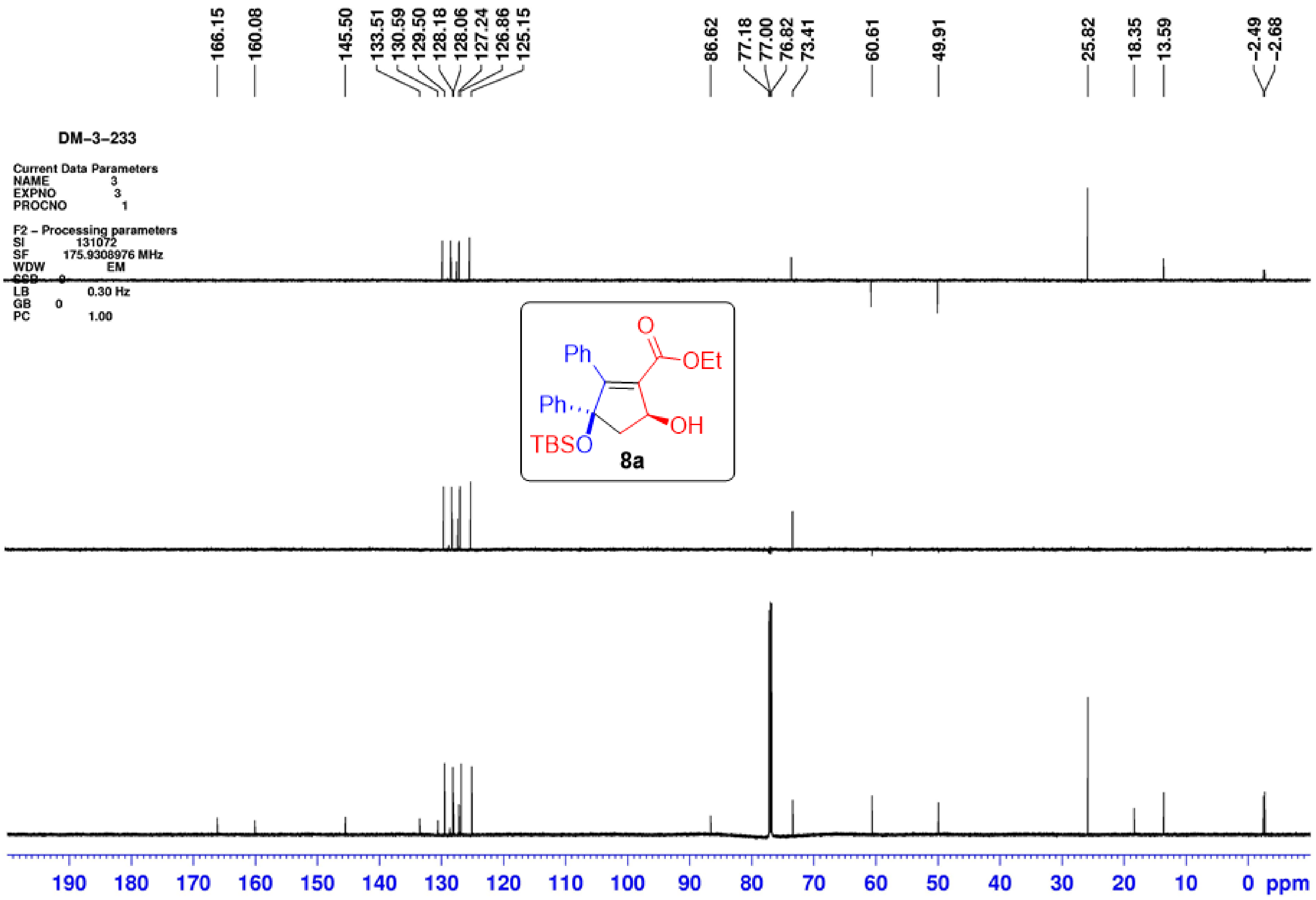

^{1}H NOE NMR ($CDCl_3$, 700 MHz)

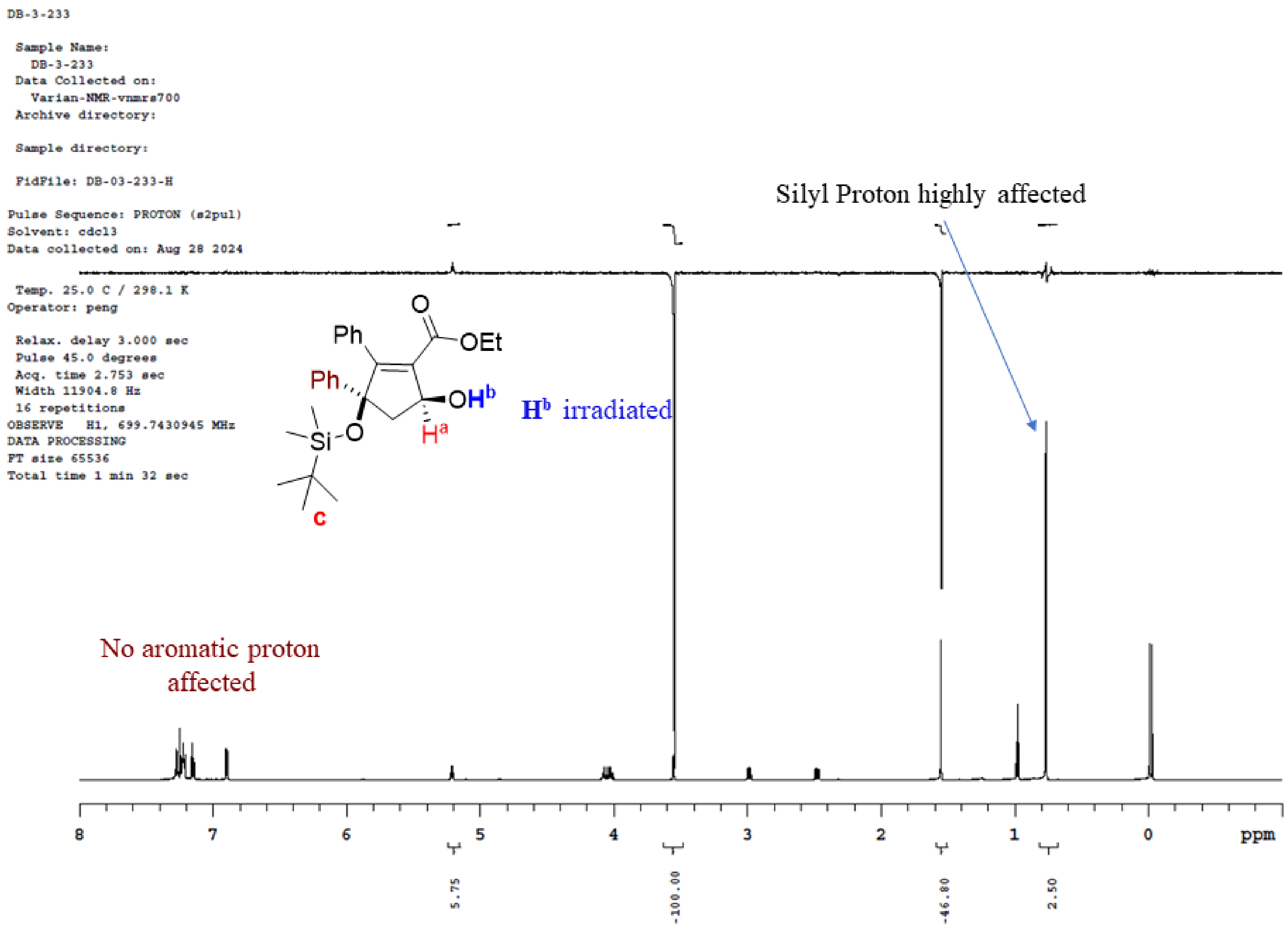

^{1}H NOE NMR ($CDCl_3$, 700 MHz)

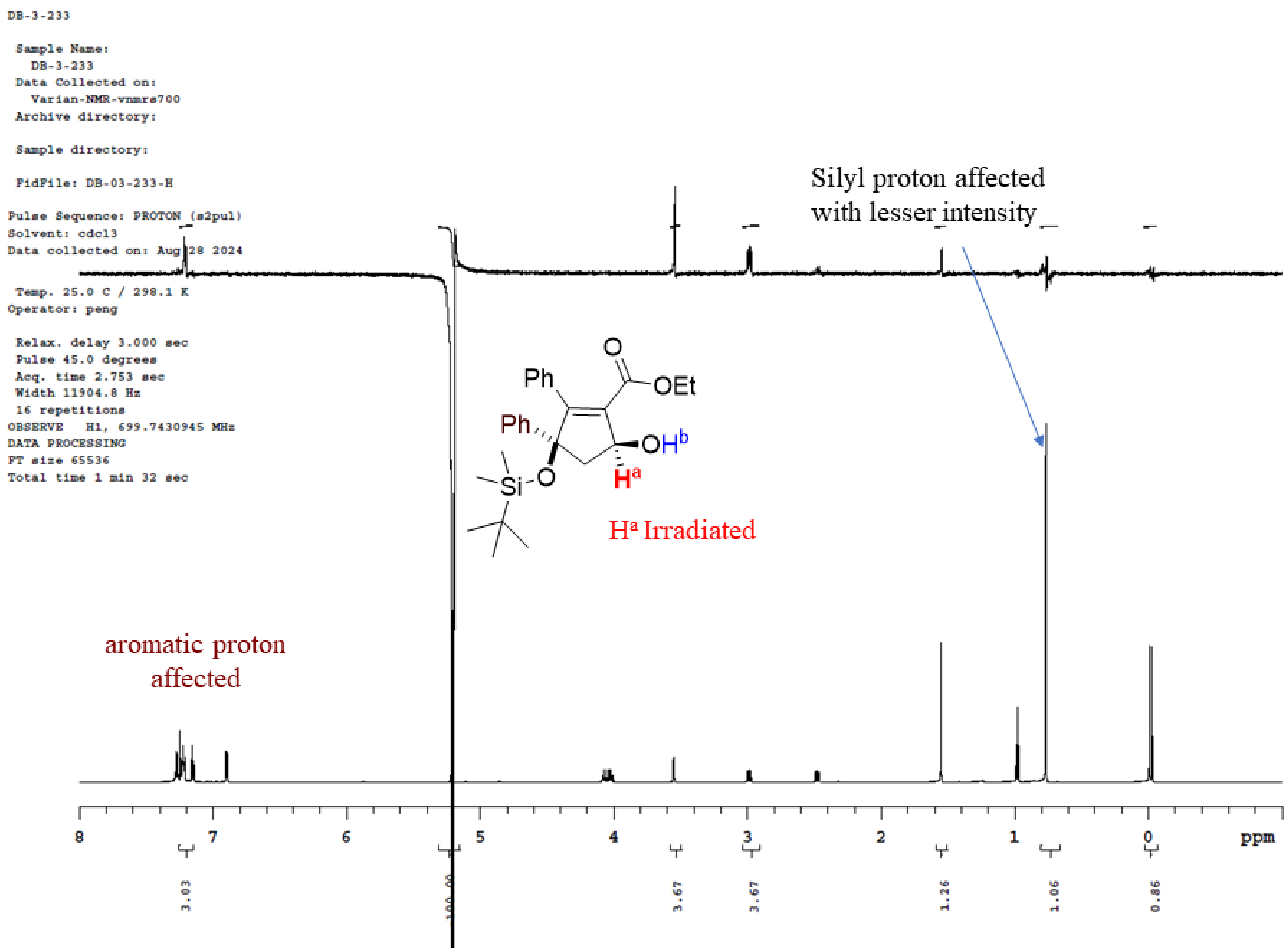

^{1}H NMR ($CDCl_3$, 400 MHz)

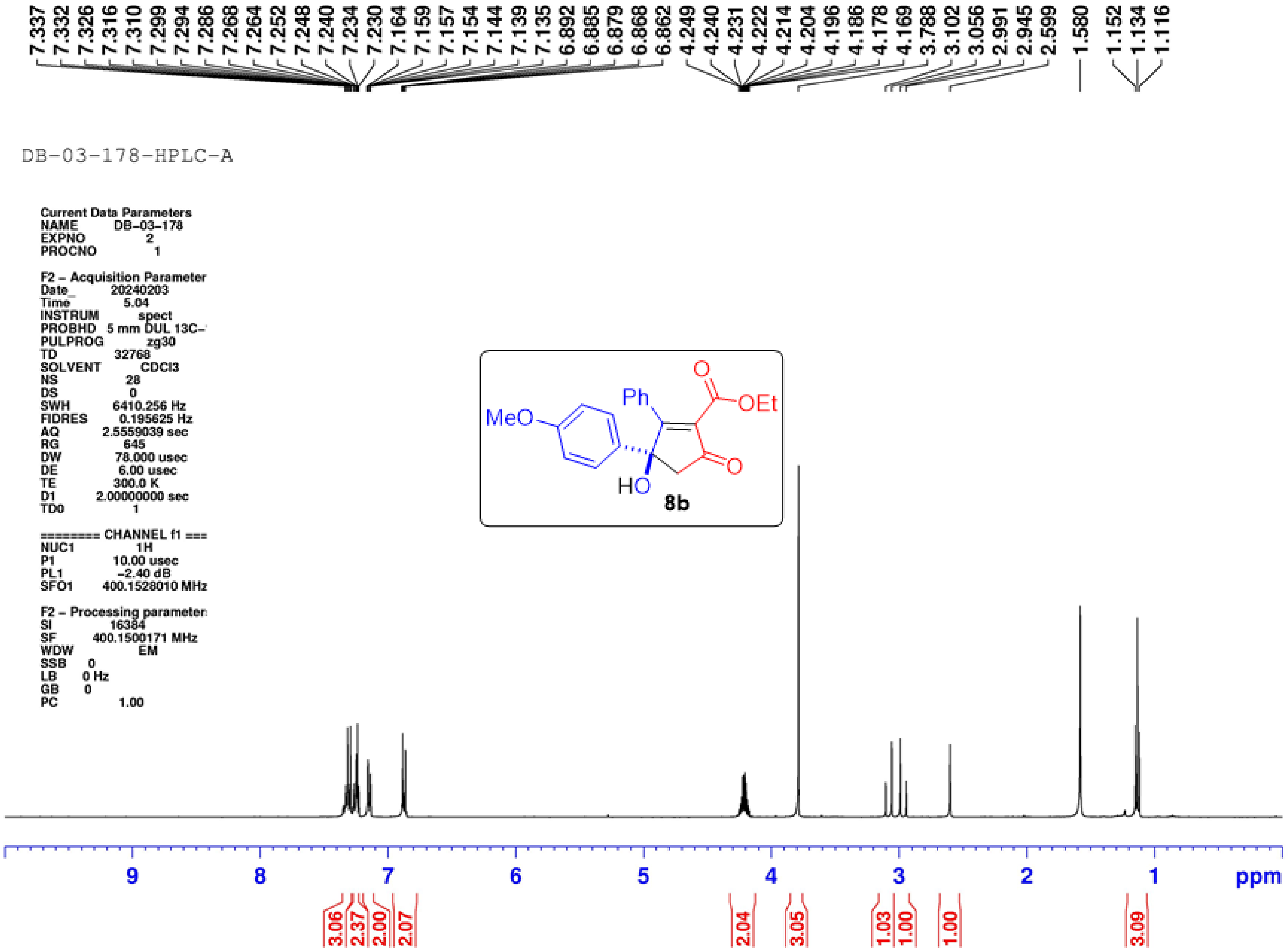

^{13}C NMR ($CDCl_3$, 100 MHz)

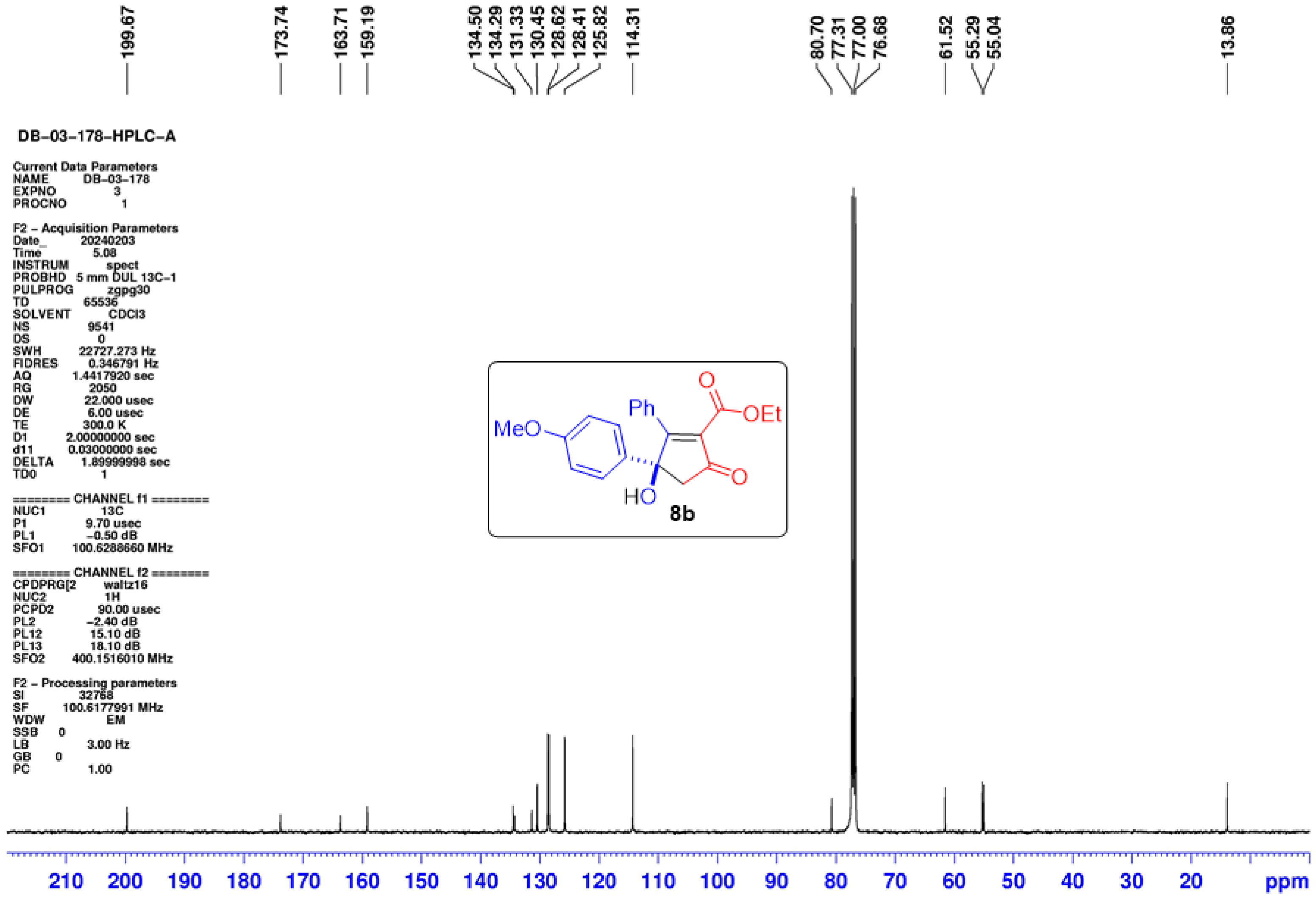

^{1}H NMR ($CDCl_3$, 400 MHz)

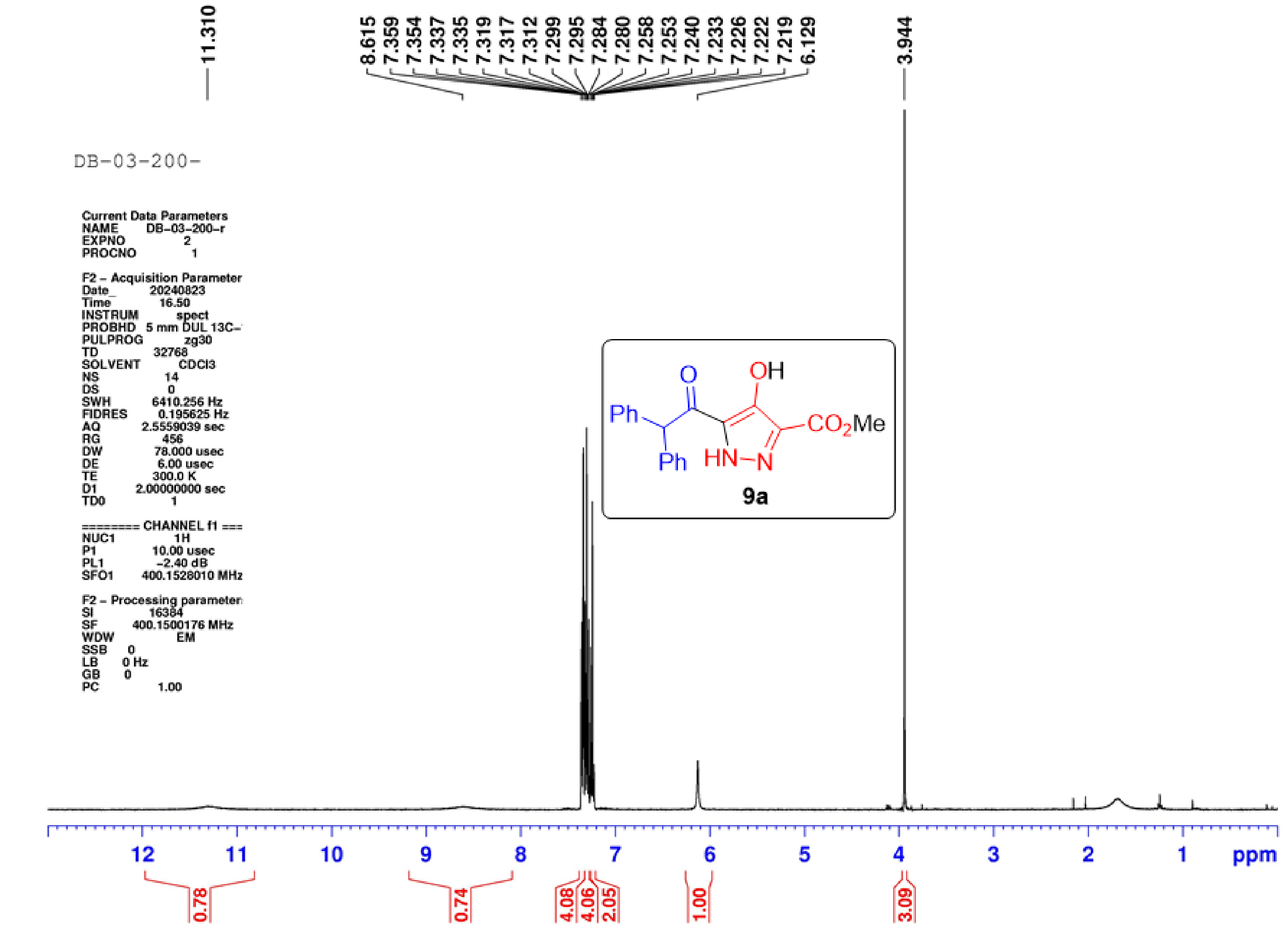

^{13}C NMR ($CDCl_3$, 100 MHz)

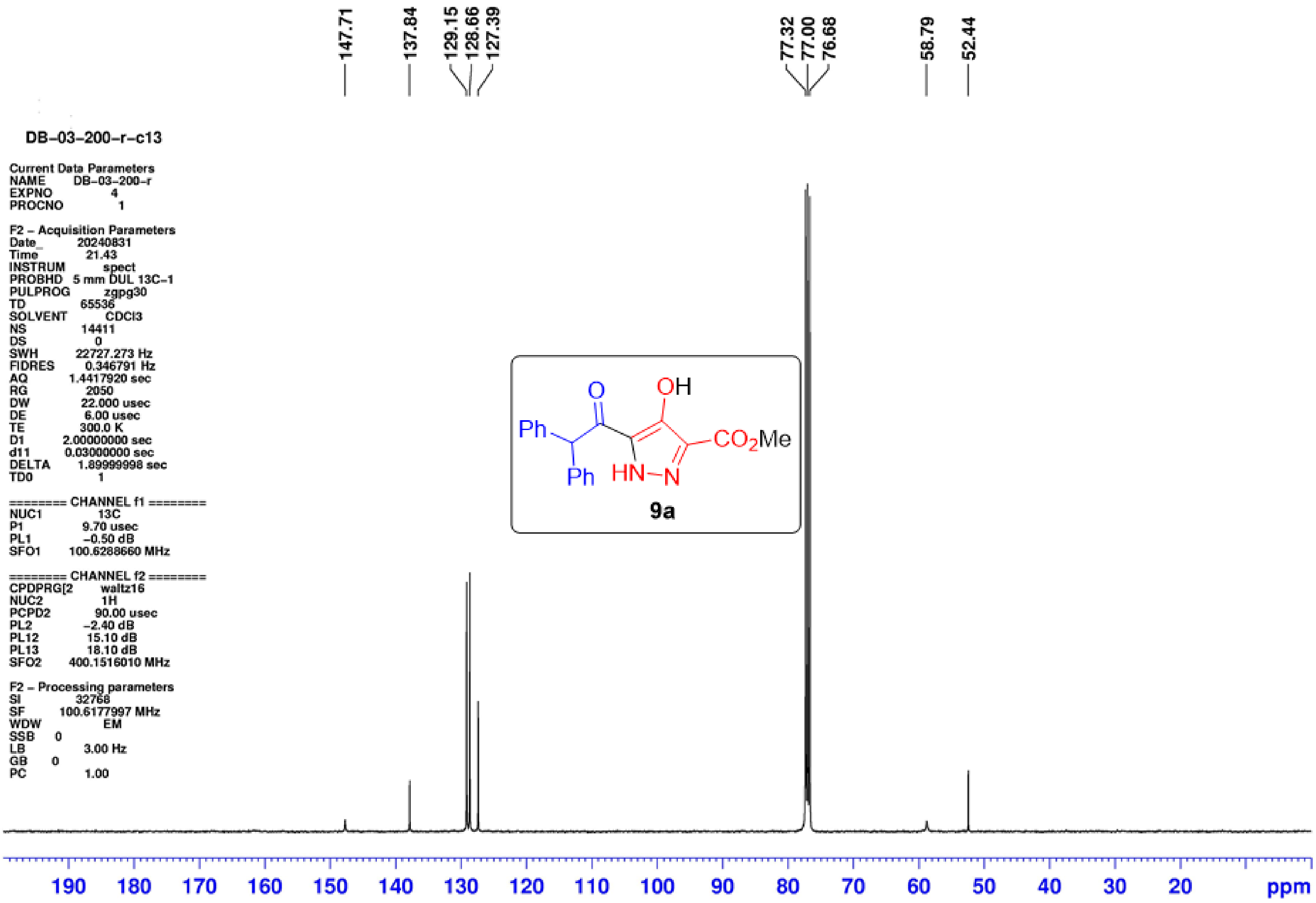

^{1}H NMR ($CDCl_3$, 700 MHz)

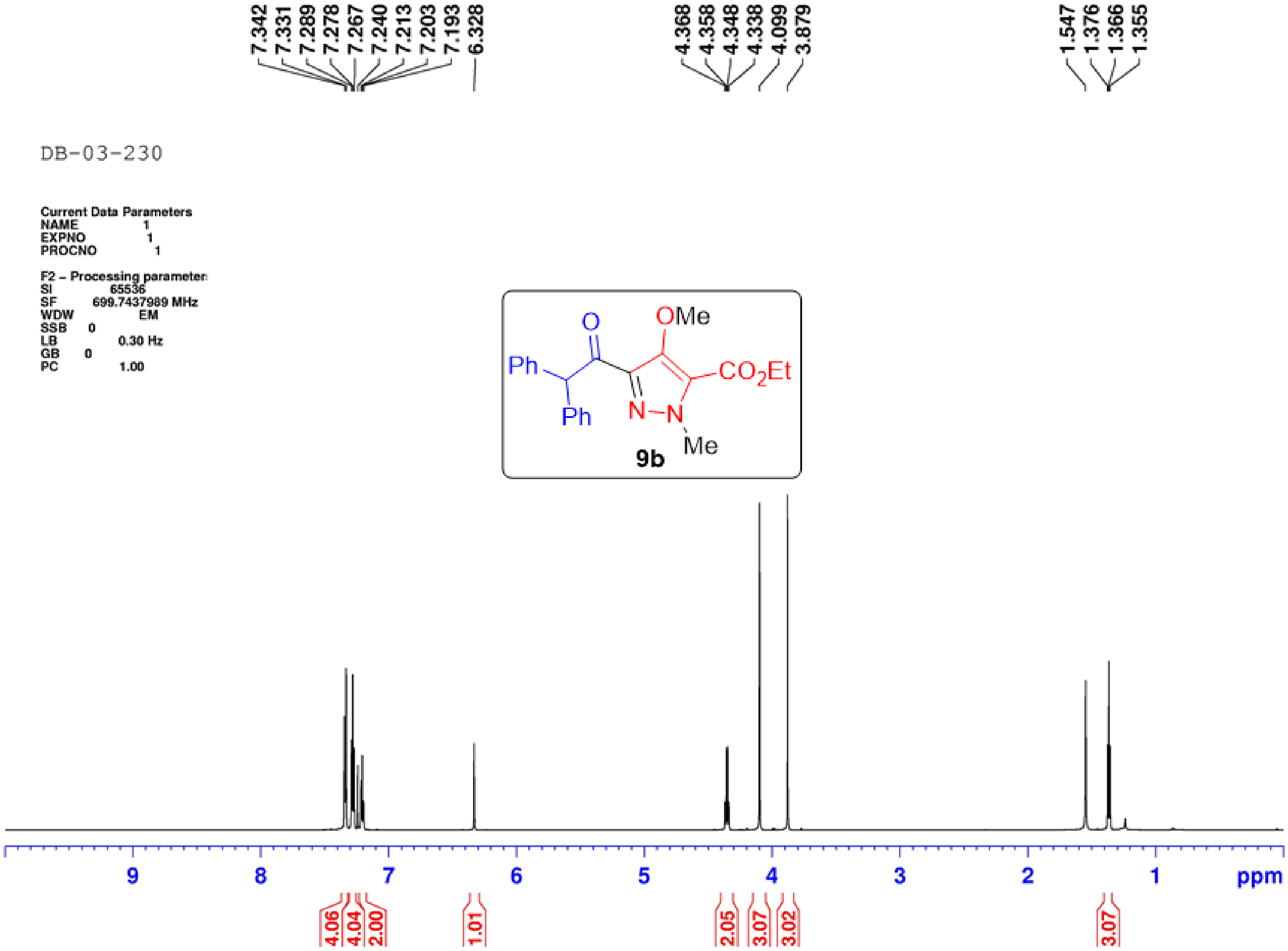

^{13}C NMR ($CDCl_3$, 175 MHz)

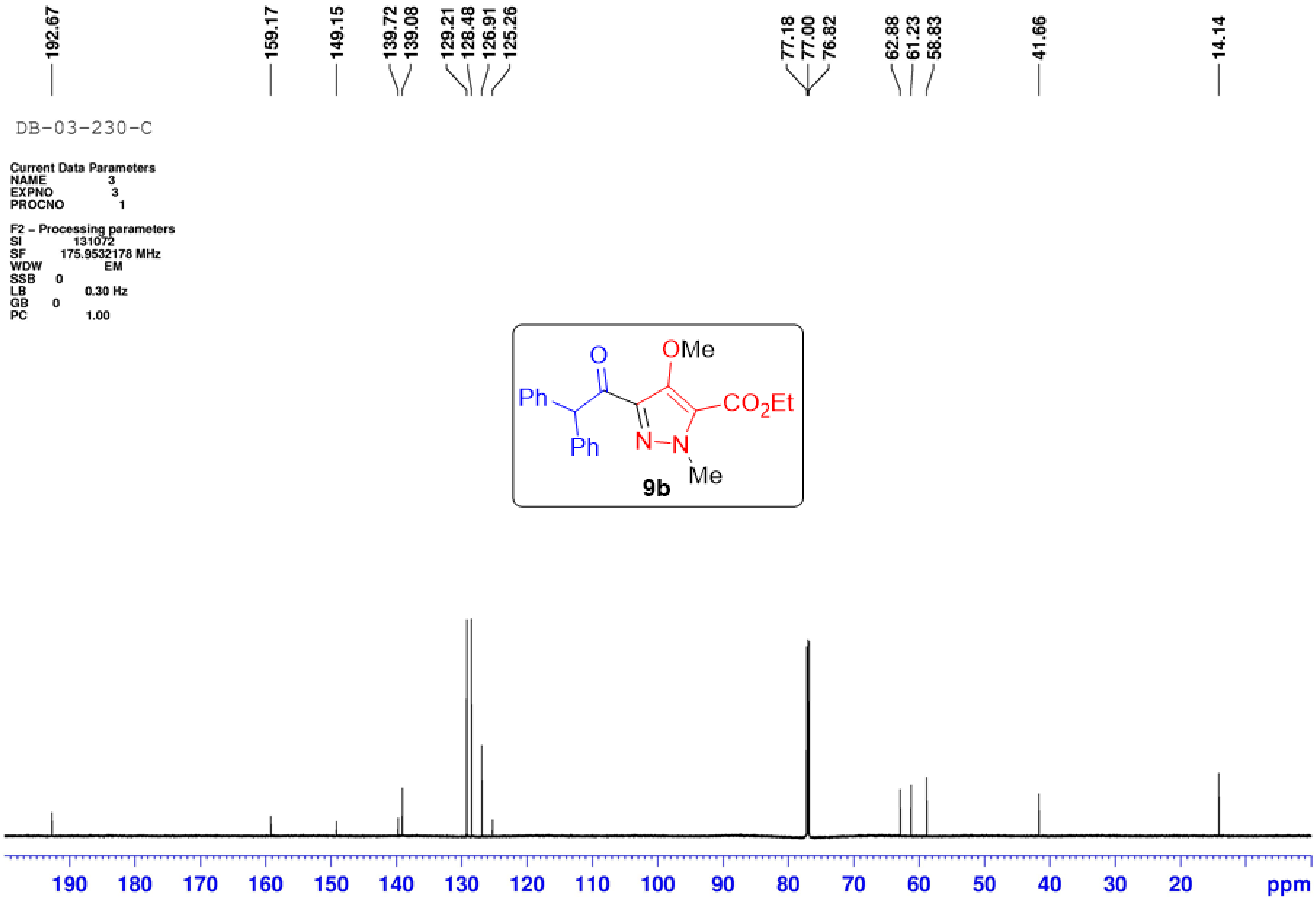

^1H NMR ($CDCl_3$, 700 MHz)

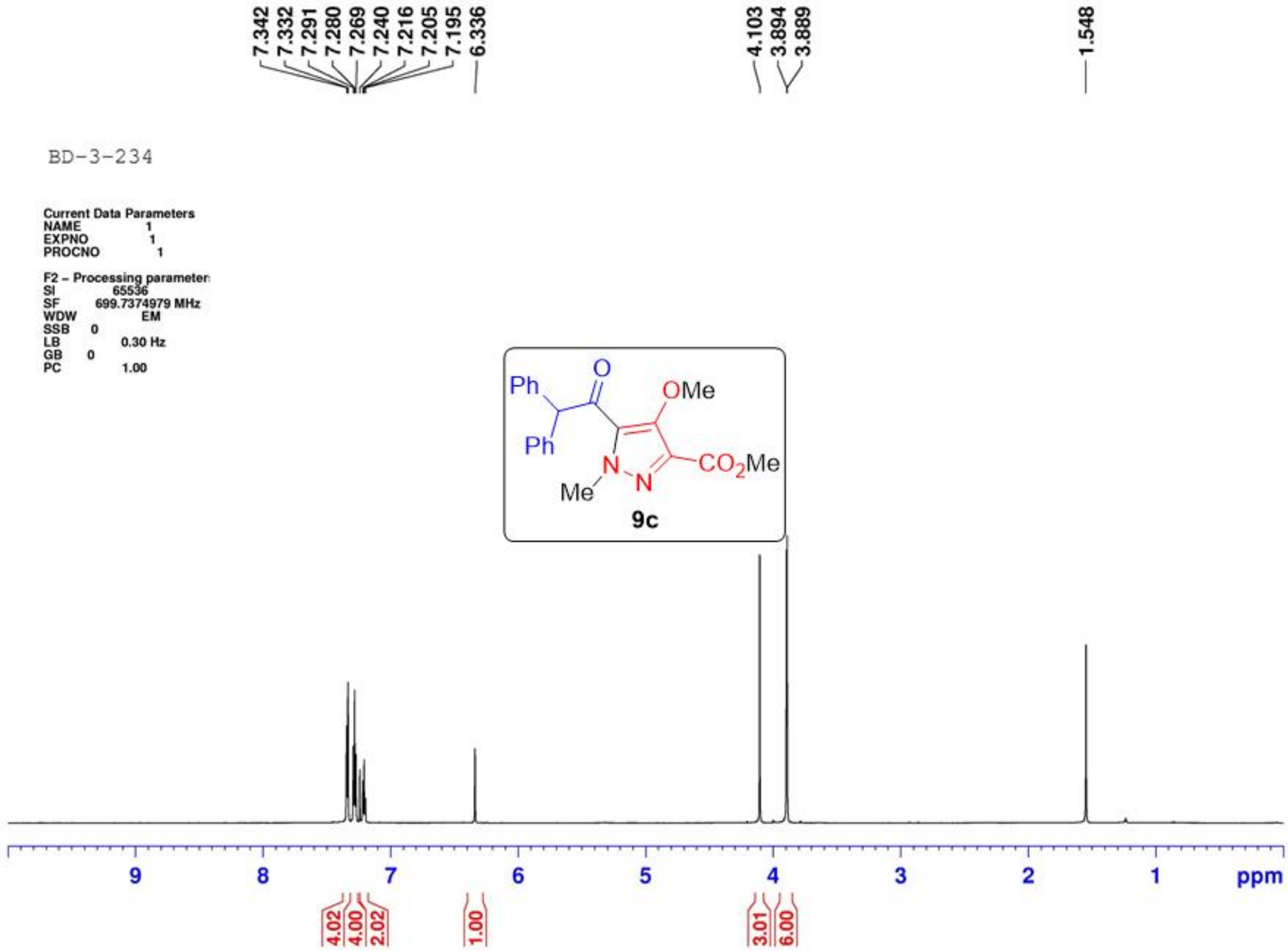

^{13}C NMR ($CDCl_3$, 175 MHz)

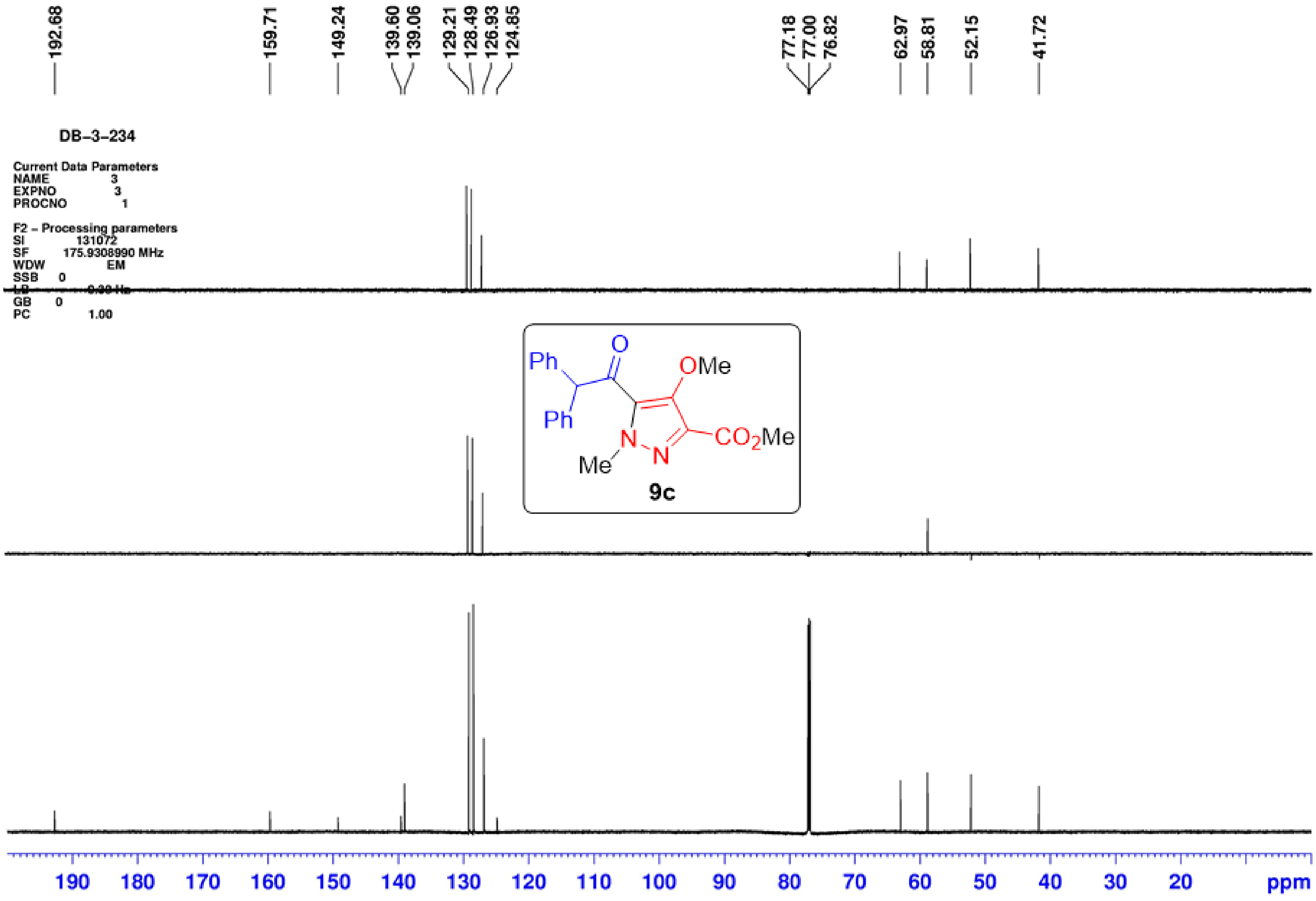

Supplement: Supplementary file 1 — ol5c00068_si_001.pdf [file ol5c00068_si_001.pdf]
